# Palladium-catalyzed regioselective and stereo-invertive ring-opening borylation of 2-arylaziridines with bis(pinacolato)diboron: experimental and computational studies†
†Electronic supplementary information (ESI) available: Experimental procedures, spectroscopic data of new compounds, copies of NMR and HPLC charts, and calculation results. See DOI: 10.1039/c6sc01120a


**DOI:** 10.1039/c6sc01120a

**Published:** 2016-06-09

**Authors:** Youhei Takeda, Akinobu Kuroda, W. M. C. Sameera, Keiji Morokuma, Satoshi Minakata

**Affiliations:** a Department of Applied Chemistry , Graduate School of Engineering , Osaka University , Yamadaoka 2-1 , Suita , Osaka 565-0871 , Japan . Email: takeda@chem.eng.osaka-u.ac.jp ; Email: minakata@chem.eng.osaka-u.ac.jp; b Fukui Institute for Fundamental Chemistry , Kyoto University , Takano-Nishihiraki-cho 34-4, Sakyo-ku , Kyoto 606-8103 , Japan . Email: morokuma.keiji.3a@kyoto-u.ac.jp

## Abstract

A palladium catalyzed regioselective borylative ring opening reaction of 2-arylaziridines to give β-amino-β-arylethylborates was developed. The reaction reported herein represents the first example of ring-opening borylation of non-vinylic aziridines and direct borylative C(sp^3^)–N bond cleavage of neutral organic substrates. NMR studies and density functional theory (DFT) calculations suggested that the active intermediate for the reaction is a PdL_2_ complex [L = P(*t*-Bu)_2_Me]. The multi-component artificial force-induced reaction method (MC-AFIR) located the transition states for the regioselectivity-determining aziridine ring opening that proceeds in an S_N_2 fashion, and explained the selectivity of the reaction. The full catalytic cycle consists of a selectivity-determining aziridine ring opening (oxidative addition), a proton transfer, phosphine ligand dissociation from the catalyst, boron–boron bond cleavage, and reductive elimination. Water is important to the drive the transmetalation step. The calculated overall mechanism and selectivity are consistent with the experimental results.

## Abstract 2

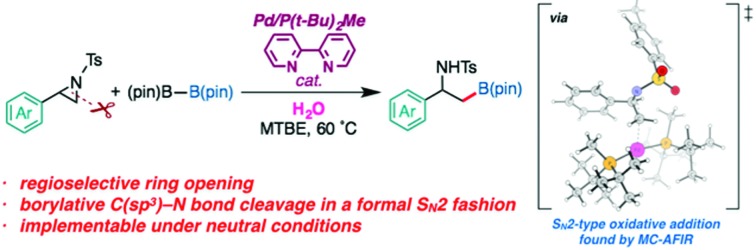
A palladium catalyzed regioselective borylative ring-opening reaction of 2-arylaziridines to give β-amino-β-arylethylborates was developed.

## Introduction

Aziridines, three-membered strained azaheterocycles, serve as versatile building blocks in modern organic synthesis.^1^ Regioselective ring opening of aziridines with nucleophiles, which is driven by release of its ring strain, is one of the most useful transformations of aziridines into ubiquitous β-amino-functionalized motifs.^2^ Aziridines undergo oxidative addition to low-valent late transition metal complexes at the terminal C–N bond in an S_N_2 fashion, generating the corresponding oxidative adducts, azametallacyclobutanes.^3^–^5^ In light of this reactivity, diverse transition metal-catalyzed regio-, stereo-, and/or chemoselective transformations of aziridines are feasible,^6^–^9^ where the oxidative addition is smoothly coupled with subsequent elementary processes, such as transmetalation, migratory insertion, and reductive elimination; regioselective Rh- and Co-catalyzed carbonylative ring expansion of aziridines, pioneered by Alper and co-workers, represent the embodiment of this idea.^6^

Over the past few years, numerous advances have been made in transition metal-catalyzed ring-opening cross coupling of aziridines, where aziridines can be utilized as a non-classical alkyl electrophilic partner [eqn (1)].^10^,^11^ In 2012, Doyle reported the first Ni-catalyzed regioselective Negishi alkylation of 2-aryl-*N*-tosylaziridines that leads to branch-type products *via* the regioselective cleavage of a benzylic C–N bond.10*a* Since this report, several groups including us11*b* have developed Ni-10 and Pd-catalyzed^11^ regioselective ring-opening cross coupling reactions of aziridines to form C(sp^3^)–C(sp^2^)/C(sp^3^) bonds, with the regioselectivity seemingly governed by the aziridine substrate rather than the catalyst [eqn (1)]. Despite the above-mentioned successes in C–C cross couplings, other catalytic ring-opening C–E (E ≠ C) bond forming reactions of aziridines based on a cross-coupling mechanism are also unexploited.^12^
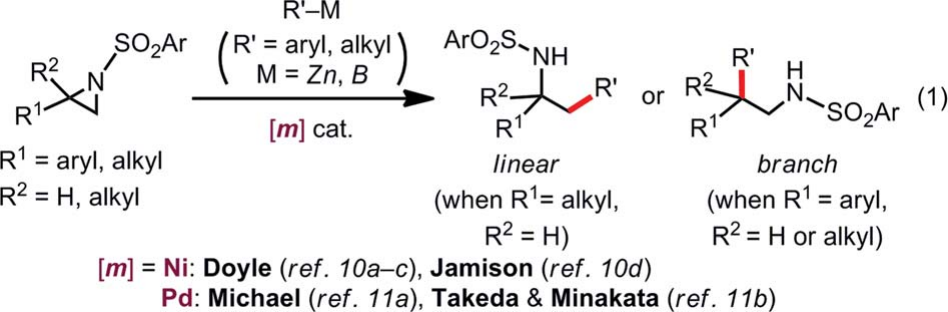


Our latest findings11*b* led us to seek new catalytic ring-opening C–E coupling systems of aziridines. Moreover, we became intrigued with the C–B coupling of aziridines to give β-amino-functionalized alkylboronates; alkylboronic acid derivatives serve not only as useful building blocks in organic synthesis, but are also biologically interesting motifs in medicinal chemistry.^13^ Only a few precedents in transition-metal-catalyzed borylative substitution of vinylaziridines *via* C(sp^3^)–N bond cleavage have been reported. Szabó and Pineschi disclosed a Pd(ii) pincer complex^14^ and a Ni(0)/BINAP^12^ system as catalysts for the borylative ring opening of vinylaziridines with diboron reagents *via* the cleavage of an allylic C–N bond to give γ-amino alkylboronic acid derivatives (formal S_N_2′-type reaction),^15^ respectively [eqn (2)]. Nevertheless, to the best of our knowledge, the catalytic direct displacement of the C(sp^3^)–N bond of aziridines with a C(sp^3^)–B bond on the same carbon (formal S_N_2-type reaction) has never been described to date.

We herein report a regioselective ring-opening C–B cross-coupling reaction of 2-arylaziridines that is realized using a Pd/P(*t*-Bu)_2_Me/bpy catalytic system to give β-amino-alkylboronates [eqn (3)], which can serve as versatile building blocks to synthesize amino-functionalized compounds and biologically relevant β-amino acid surrogates.^16^,^17^ Notably, our reaction features the opposite regioselectivity in C–N bond cleavage (at the 3-position) to those previously reported for C–C cross couplings using the same type of aziridine substrate (at the 2-position).10a,c,11b Also, our C–B coupling system represents the first example of a formal S_N_2-type borylative C(sp^3^)–N bond cleavage of neutral compounds.^18^ It should be noted that the present C–B coupling proceeds smoothly under neutral conditions as in the cases of borylative substitution of allyl carboxylates^19^ and carbonates.19a,^20^ In these cases, the leaving group-derived oxy anions (RCO_2_^–^ and RO^–^) serve as the internal bases, while the metal catalyzed borylative substitution of alkyl halides usually requires the addition of stoichiometric external base or activator to promote transmetalation.^21^ In addition to experimental elaboration, we have performed theoretical calculations, applying density functional theory (DFT) and the multi-component artificial force-induced reaction (MC-AFIR) method to determine the mechanism of the reaction and explain the origin of the selectivity, and to clarify why external base is not required in this reaction system.
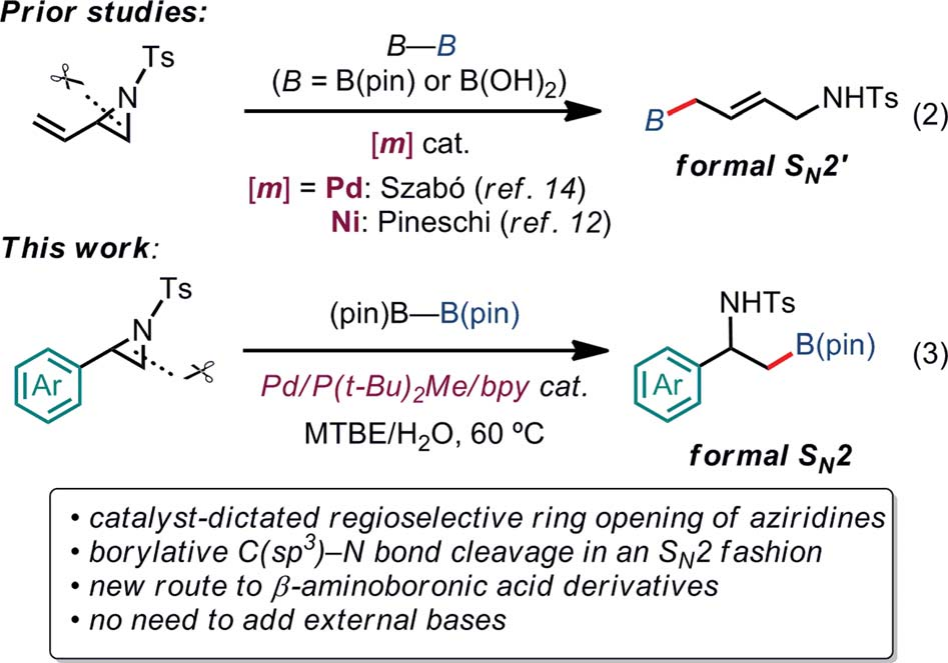


## Results and discussion

### Experimental part

#### Development of Pd-catalyzed borylative ring-opening reaction of 2-arylaziridines

To identify the reaction conditions for the borylative ring-opening reaction, optimization studies applying racemic 2-phenyl-*N*-tosyl-aziridines (**1a**) as a model substrate with bis(pinacolato)diboron B_2_(pin)_2_ (**2**) were performed (Tables S1–S13 in the ESI†).^22^ The executive summary of the optimization study is shown in Table 1.

**Table 1:** Evaluation of reaction parameters

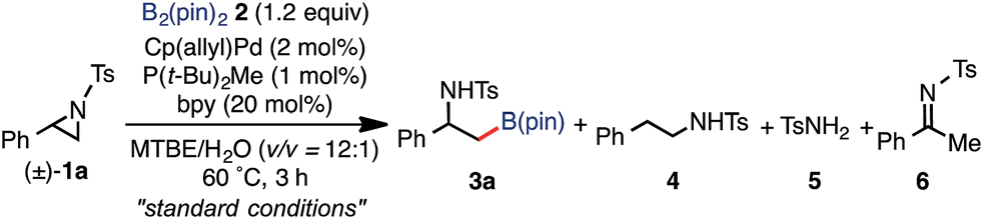						
Entry	Difference from the “standard conditions”	Yield^*a*^ (%)				Recovery of **1a**^*a*^ (%)
		**3a**	**4**	**5**	**6**	
1	None	81	0	0	13	0
2	Cp(allyl)Pd (1 mol%)/P(*t*-Bu)_2_Me (2 mol%)	74	0	11	12	3
3	Cp(allyl)Pd (1 mol%)/P(*t*-Bu)_2_Me (3 mol%)	45	0	10	14	0
4^*b*^	Without bpy	52	0	14	25	0
5^*b*^	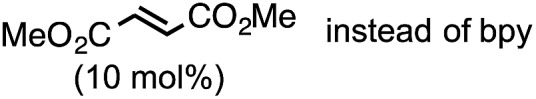	0	0	10	0	90
6^*b*^	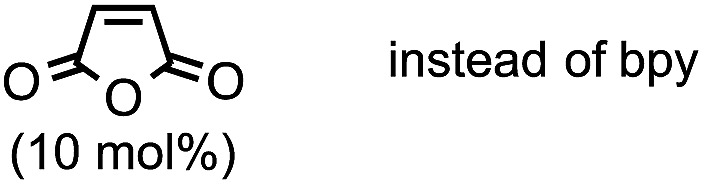	Trace	0	3	0	96
7^*b*^	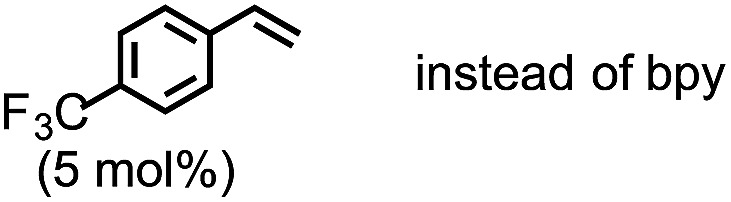	64	0	8	16	0
8	Pd_2_(dba)_3_/SIPr was used as catalyst	0	5	37	0	48
9	Pd_2_(dba)_3_/PCy_3_ was used as catalyst	5	10	0	0	85
10	Pd[P(*t*-Bu)_3_]_2_ was used as catalyst	0	0	0	0	97
11^*c*^	Without H_2_O	0	0	0	7	84

^*a*^Determined with GC or ^1^H NMR.

^*b*^1 mol% of Cp(allyl)Pd and 2 mol% of ligand were used.

^*c*^1 mol% of Cp(allyl)Pd and 0.5 mol% of ligand were used.

The highest yield (81%, isolated yield: 71%) of borylated product **3a** was obtained when **1a** was treated with 1.2 equiv. of B_2_(pin)_2_ at 60 °C in the presence of catalytic amounts of Cp(allyl)Pd (2 mol%), P(*t*-Bu)_2_Me^23^ (1 mol%), and 2,2′-bipyridine (bpy, 20 mol%) in a mixed solvent of methyl-*tert*-butylether (MTBE) and H_2_O (entry 1, “standard conditions”). Most importantly, the regioselectivity of this ring opening and C–B coupling was opposite to those observed with the C–C coupling of the same substrate,10a,c,11b implying that the oxidative addition occurred at the C–N bond on the terminal carbon (the 3-position of the aziridine). The regioisomer of **3a** was not detected in the crude ^1^H NMR spectra, indicating that the regioselectivity in the ring-opening of **1a** should be almost perfect. In fact, the regioselective oxidative addition dictated by the interactions between the substrate and the Pd(0) catalyst was supported by theoretical calculations (*vide infra*). The L : Pd ratio [L = P(*t*-Bu)_2_Me] was found to have a significant impact on the product distribution (entries 2 and 3). As the L : Pd ratio was increased from 0.5 (entry 1) to 2 to 3, the yields of **3a** decreased to 74% and 45%, respectively (entries 2 and 3). This irregular ratio of L : Pd implies that there might be a complex equilibrium of PdL_*n*_ species generated *in situ*, some of which are specifically active in the catalytic cycle. In fact, this speculation was partly supported with ^31^P NMR experiments and theoretical calculations (*vide infra*). Addition of electron deficient unsaturated compounds, which can coordinate to metal complexes, is an effective strategy to suppress undesired β-hydride elimination or to accelerate reductive elimination, thereby often leading to better results in alkyl cross coupling reactions.^24^ Indeed, the effect of adding bpy was significantly effective for suppressing the formation of byproduct **6** as well as **5**, which was presumably derived from the hydrolysis of **6** with H_2_O (entries 4–7). The effect of the ligands was also significant (entries 8–10). N-heterocyclic carbene (NHC) ligands, SIPr for instance,11*b* promoted the consumption of **1a** and produced undesired products **4** and **5** (entry 8). The results obtained from sterically demanding trialkylphosphine/Pd catalysts (entries 9 and 10) suggest a sluggish oxidative addition step, which is in good agreement with Wolfe's report.^9^ Furthermore, as mentioned in the introduction section, the ring opening borylation disclosed herein does not require the addition of any external base, which is usually used to activate the transmetalation step for the borylative substitution of alkyl halides. In connection with this, addition of water is crucial. Moreover, in the absence of H_2_O, no conversion of **1a** was observed (entry 11), while at least 1 equiv. of H_2_O allowed the borylation (Table S11 in the ESI†). Theoretical calculations implied that H_2_O serves as a proton source (H^+^) as well as a source of internal base (Pd–OH) to promote the four-membered transmetalation between B_2_(pin)_2_ and Pd (*vide infra*). To investigate the fate of the other B(pin) moiety of B_2_(pin)_2_ in the reaction, the ^11^B NMR spectrum of the crude product was acquired in benzene-*d*_6_. The only peak other than the remaining B_2_(pin)_2_ was detected at *δ* = 22.6 ppm as a slightly broad singlet. The comparison with the reported ^11^B resonances of (pin)B–OH (*δ* = 22.5 ppm in benzene-*d*_6_)25*a* and (pin)B–O–B(pin) (*δ* = 21.6 ppm in benzene-*d*_6_)25*b* would suggest that one of the B(pin) moieties of the starting diboron reagent was converted to (pin)B–OH during the reaction.

#### Substrate scope and synthetic utility

After establishing the optimized conditions, we explored the substrate scope of the borylative substitution reaction (Table 2). In terms of 2-arylaziridines, *p*-, *m*-, and *o*-tolyl-substituted aziridines **2b**, **2c**, and **2d** smoothly underwent borylative ring opening in a regioselective manner to give the corresponding β-amide-β-tolylethylboronates **3b**, **3c**, and **3d** in good yields. Aziridine bearing a *p*-fluorophenyl substituent at the 2-position was efficiently borylated, giving the corresponding alkylboronate **3e** in a high yield. It is important to note that the *p*-Cl functionality on the aryl ring of aziridine **2f** survived the reaction conditions to provide the borylative product **3f** in a high yield. Nevertheless, the reaction with *p*-bromophenyl-substituted aziridine did not proceed at all. An invaluable functional group in medicinal chemistry, a CF_3_-installed aziridine **2g** gave the corresponding CF_3_-bearing alkylboronate **3g**. Notably, this ring opening borylation was applicable to the arylaziridines bearing a strong electron withdrawing group (NO_2_, OAc, and CO_2_Me), and kept these functionalities intact. This highlights an advantage of using neutral conditions.

**Table 2:** Scope and limitation of the borylation^*a*^ ^,^^*b*^

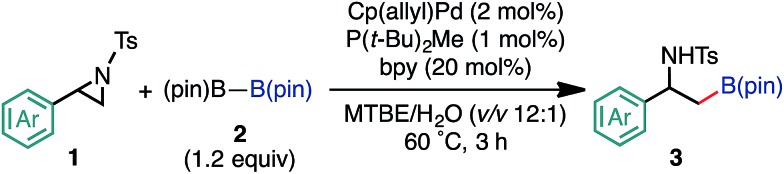
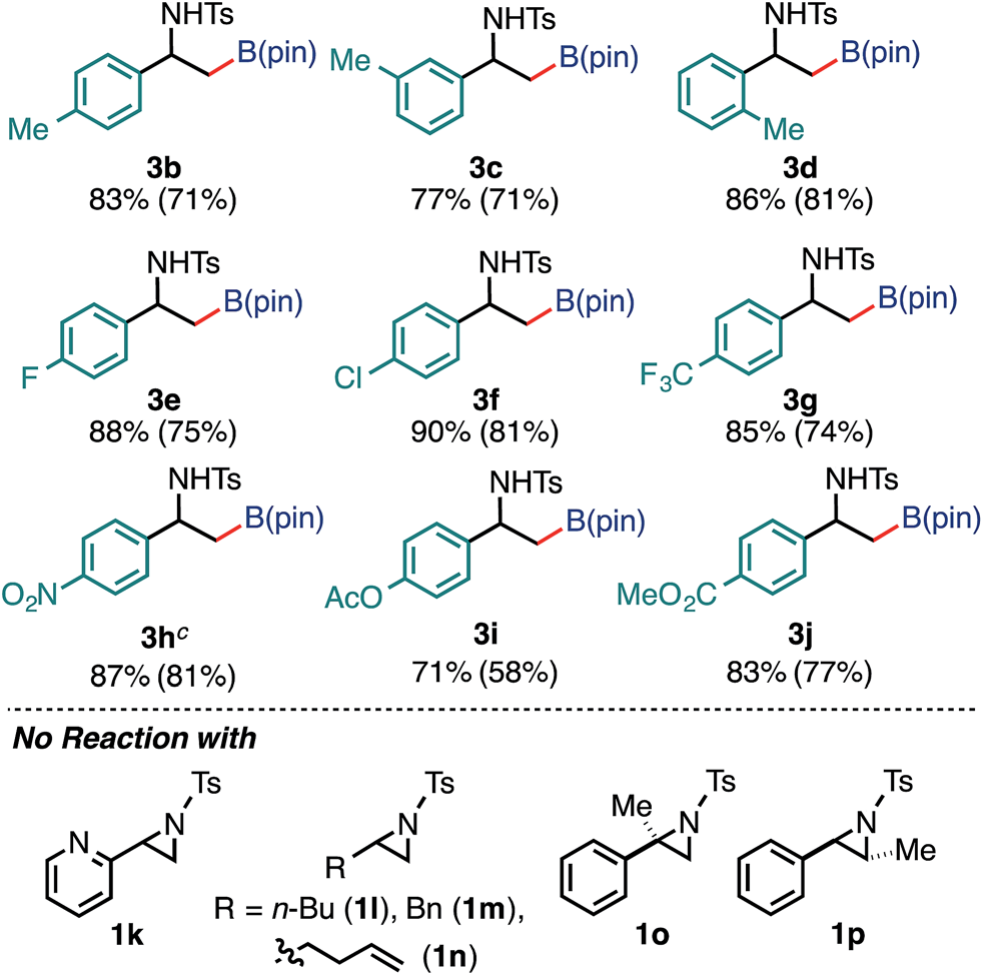

^*a*^Reaction conditions: **1** (0.50 mmol), **2** (0.60 mmol), Cp(allyl)Pd (10 μmol), P(*t*-Bu)_2_Me (5 μmol), bpy (10 μmol) in MTBE/H_2_O (1.5 mL, v/v 12 : 1) at 60 °C under N_2_ atmosphere for 3 h.

^*b*^The values outside and inside parentheses indicate ^1^H NMR and isolated yields, respectively.

^*c*^The reaction was conducted at 80 °C.

Furthermore, the borylation was scalable, and **3a** was prepared on a gram scale (2.89 g, 72% yield) from 10 mmol of **1a** (pS17 of the ESI†). With respect to the limitation of substrate, 2-pyridyl- (**1k**) and 2-alkylaziridines (**1l**, **1m**, and **1n**) were not consumed under the reaction conditions. Furthermore, this reaction is very sensitive to the steric hindrance around the 3-positioned carbon, where oxidative addition to Pd(0) has occurred. For example, 2-methyl-2-phenylaziridine (**1o**) and *trans*-2-phenyl-3-methylaziridine (**1p**) were not consumed at all under the standard conditions. As for the scope of the *N*-functional groups of aziridines, *p*-MeOC_6_H_4_SO_2_ and *t*-BuSO_2_-substituted 2-phenylaziridines smoothly underwent borylation in a regioselective manner (at the 3-position) to produce the corresponding β-amino-β-phenylethylboronates **3r** (65%) and **3s** (52%), respectively. On the other hand, *N-p*-nosyl-2-phenylaziridine did not give borylated product (Table S12 in the ESI†). The use of an *N*-acylated aziridine, *N*-Boc-2-phenylaziridine for instance, did not give borylated products at a noticeable level, highlighting the efficacy of the *N*-tosyl group for the borylation. Regarding the diboron reagents, the use of bis(neopentyl glycolato)diboron gave the corresponding aminoboronate product (**3a′**) in 39% yield, while the borylation using bis(hexylene glycolato)diboron produced the corresponding borylated products in a low yield as an inseparable diastereomeric mixture, presumably due to the existence of two chiral centers on the benzylic and glycolate carbons (Table S13 in the ESI†).

Borylated product **3a** was successfully transformed into β-amino-β-aryl-substituted alkyltrifluoroborate **7** in excellent yield, which can serve as a versatile building block to synthesize β-amino-β-arylethanes through Pd-catalyzed cross coupling reactions [eqn (4)].^26^
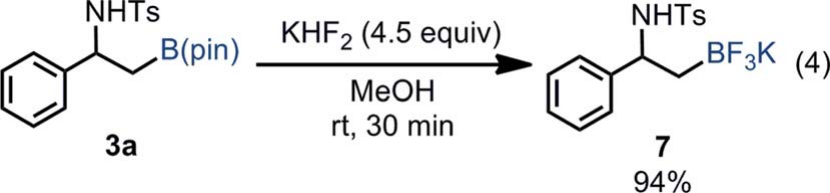


**Scheme 1:**
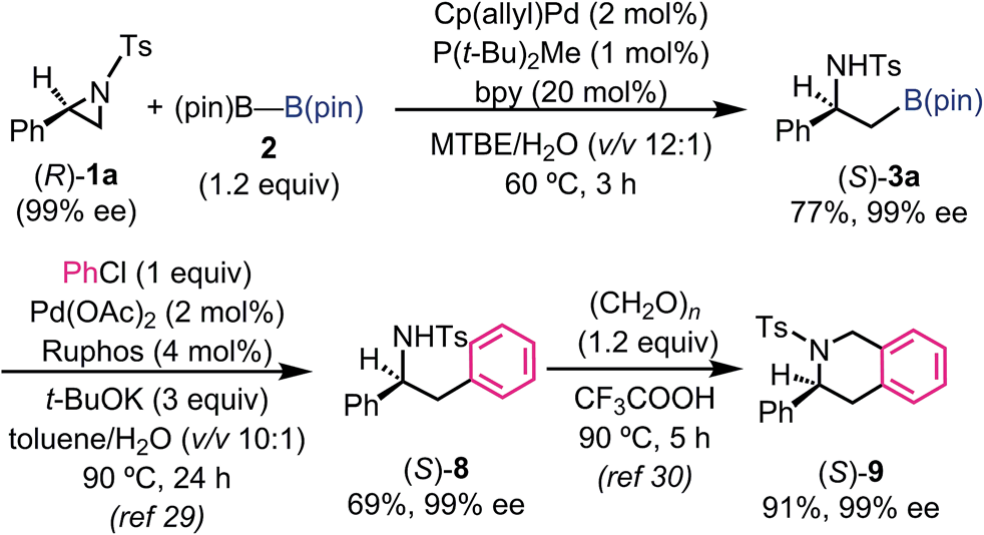
Preparation of enantiopure β-aminoboronate and its synthetic application.

In order to understand the stereochemical outcome of the secondary stereogenic center of the borylated product and to demonstrate the utility of the alkylboronate, an enantiopure aziridine (*R*)-**1a** (99% ee from chiral HPLC analysis) was subjected to the standard reaction conditions (Scheme 1). HPLC analysis of the borylated product **3a** revealed that the stereochemical information was completely retained (99% ee, *S*) through the reaction. This result excludes the possibility of the reaction pathway of oxidative addition/β-hydride elimination/re-insertion of [Pd–H] species leading to **3a**, although it cannot perfectly exclude such a pathway.^27^ To demonstrate the synthetic utility of the enantiopure β-amino-alkylboronate (*S*)-**3a**, the asymmetric synthesis of 3-phenyl-1,2,3,4-tetrahydroisoquinoline derivative was conducted (Scheme 1). The tetrahydroisoquinoline skeleton is a ubiquitous motif in alkaloid natural products and constitutes a biologically important azaheterocycle.^28^ Enantiopure alkylboronate (*S*)-**3a** was subjected to the Pd-catalyzed Suzuki–Miyaura cross coupling^29^ with chlorobenzene to give the optically active 1,2-diphenylamine derivative (*S*)-**8** in 69% yield, while keeping the enantiopurity intact (99% ee, chiral HPLC). The following Pictet–Spengler reaction^30^ successfully gave the enantiopure tetrahydroisoquinoline product (*S*)-**9** in excellent yield.

#### Mechanistic aspect

**Scheme 2:**
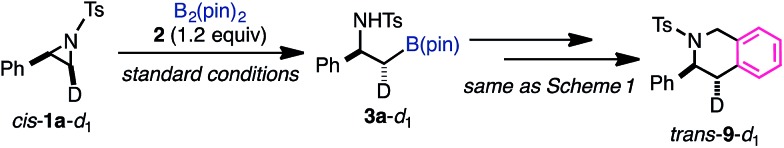
Borylation of deuterated aziridine.

To obtain the stereochemical course in the oxidative addition step, a similar synthetic sequence using deuterated aziridine *cis*-**1a**-*d*_1_ as a substrate was conducted (Scheme 2). The stereochemistry on the terminal carbon (the 3-position) was completely inverted (for details, see the ESI†). This result agrees with the S_N_2 nature of the oxidative addition process of aziridine toward Pd(0) complexes reported by our group11*b* and others.^5^,11a Moreover, this stereo-invertive process is supported with computational studies (*vide infra*).

**Fig. 1:**
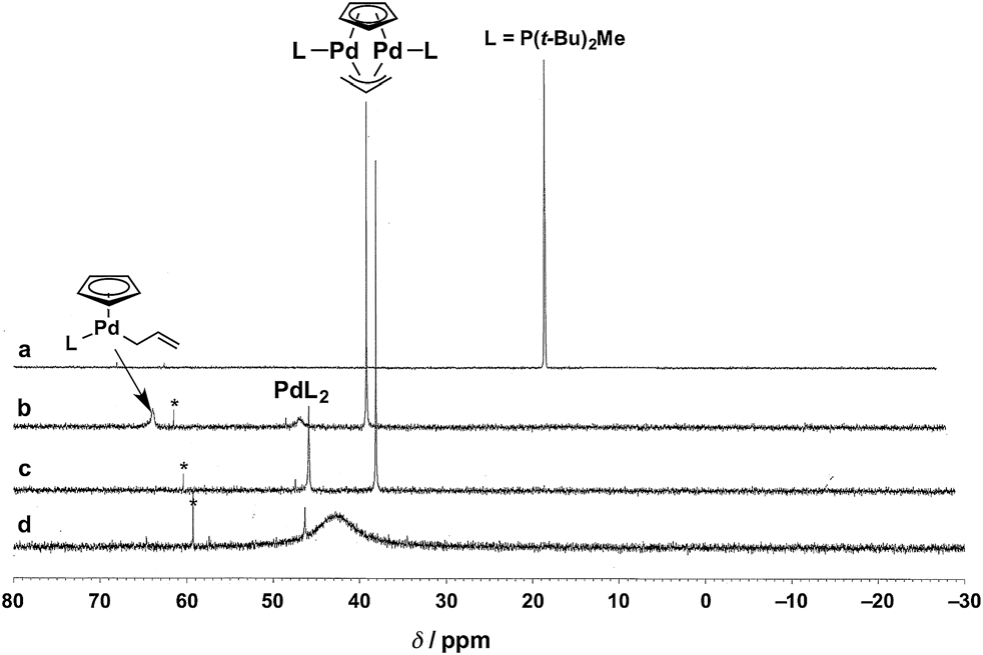
Stacked ^31^P{^1^H} NMR spectra acquired at 60 °C in THF-*d*_8_. (a) Free L [L = P(*t*-Bu)_2_Me]; (b, c) the reaction of Cp(allyl)Pd with 0.5 equiv. of L ((b) upon mixing, (c) 1 h); (d) the reaction of Cp(allyl)Pd with 1 equiv. of L after 1 h. The peaks with asterisks are attributed to phosphine oxide.

To identify the Pd species generated by mixing P(*t*-Bu)_2_Me and Cp(allyl)Pd, we monitored the ^31^P{^1^H} NMR spectra of these mixtures in THF-*d*_8_ at 60 °C (Fig. 1). Upon addition of 2 equiv. of Cp(allyl)Pd to L (*i.e.*, L : Pd = 0.5), the sharp resonance corresponding to free L at *δ* 15.3 ppm (Fig. 1a, lit. *δ* 12.5 ppm at room temperature in toluene-*d*_8_),^31^ completely disappeared and three new peaks appeared at *δ* 37.0 (sharp, strong), 44.7 (broad, weak), and 62.0 (broad, weak) ppm (Fig. 1b). According to the data reported by the Baird group,^32^ these peaks are assignable to a dinuclear Pd(i) complex [Pd_2_L_2_(μ-Cp)(μ-allyl)] (lit. *δ* 35.4 ppm at 65 °C in toluene-*d*_8_), a bisphosphine Pd(0) complex PdL_2_ (lit. *δ* 41.0 ppm at 65 °C in toluene-*d*_8_), and an [(η^5^-Cp)Pd(η^1^-allyl)L] species ([(η^5^-Cp)Pd(η^1^-PhC_3_H_4_)L] lit. *δ* 61.7 ppm at room temperature in toluene-*d*_8_), respectively, as illustrated in Fig. 1. After stirring the mixture at 60 °C for 1 h, the broadened weak peak at *δ* 62.0 ppm corresponding to [(η^5^-Cp)Pd(η^1^-allyl)L] disappeared, and the intensity of the peak at *δ* 44.7 ppm (PdL_2_) increased as well (the integration ratio of [Pd_2_L_2_(μ-Cp)(μ-allyl)] : PdL_2_ = 3 : 2, Fig. 1c). These peaks were not changed when bpy (10 equiv.) was added (Fig. S1b in the ESI†). This result would indicate that bpy does not coordinate to these Pd species, which was also supported by our computational results (*vide infra*). Moreover, upon addition of another equivalent of L (*i.e.*, L : Pd = 1), the strongest sharp peak at *δ* 37.0 ppm (dinuclear Pd complex) disappeared, and a broad peak around *δ* 42.6 ppm appeared (Fig. 1d). Furthermore, addition of one more equivalent of L (L : Pd = 2) gave a significantly broadened peak (*δ* ∼ 30 ppm, Fig. S1a in the ESI†), indicating significant exchange among several PdL_*n*_ species. Our observations could indicate that PdL_2_ undergoes rapid exchange with PdL_*n*_ in THF-*d*_8_ when the L : Pd ratio is greater than 0.5. Therefore, the same would be the case with the real reaction system.

**Scheme 3:**
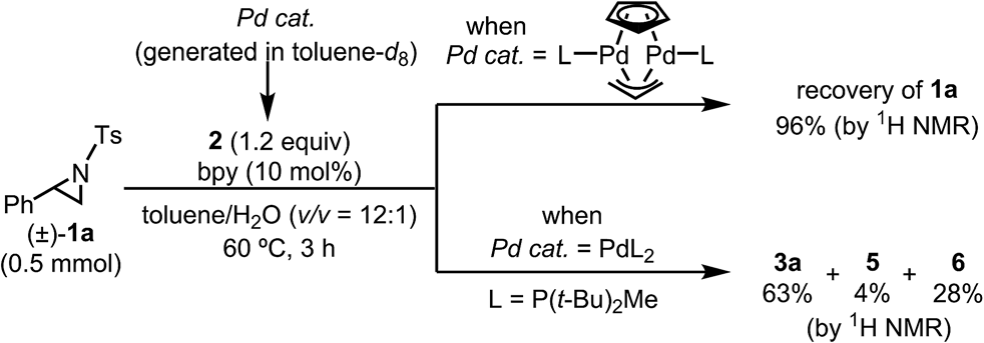
Verification test of the catalytically active species.

Based on the NMR experiments, [Pd_2_L_2_(μ-Cp)(μ-allyl)] or PdL_2_ would be the catalytically active Pd species (*i.e.*, the starting point of the catalytic cycle). To determine which is the active species in the catalytic reaction, both complexes were generated separately as the sole components according to Baird's conditions (*i.e.*, 1 : 1 and 2 : 1 mixtures of L and Cp(allyl)Pd were mixed in toluene-*d*_8_ and stirred at 77 °C for 1 h),^32^ and the resulting Pd complexes were used as a catalyst in the borylation reaction in toluene (Schemes 3). As a result, while the dinuclear Pd(i) complex was found to be totally inert for the borylation reaction, the bisphosphine complex Pd(0)L_2_ gave almost the same result as the real reaction system. Therefore, we conclude that Pd(0)L_2_ would be the starting active species in the catalytic cycle, and this conclusion was strongly supported by theoretical calculations (*vide infra*).

### Computational part

#### Computational methods

Geometry optimizations were performed with the B3LYP-D3 33–36 functional as implemented in the Gaussian09 program.^37^ The polarizable continuum model (PCM) was used as the implicit solvent model with a dielectric constant (*ε*) of 2.3714.^38^ The SDD basis set and associated effective core potential were applied for Pd,^39^,^40^ and 6-31G(d) basis sets were used for the other atoms (BS1).^41^–^44^ All stationary points, local minima (LMs) or transition states (TSs), were optimized without any constraint. Vibrational frequency calculations were performed to confirm the nature of the stationary points. Pseudo-IRC calculations were performed to confirm the connectivity between the TSs and LMs. The final potential energies of the optimized structures were calculated as single-point energies on the optimized structures, where a SDD basis set was used for Pd, and cc-pVTZ basis sets were used for the other atoms (BS2).^45^–^47^ In the results section, we report both the relative Gibbs free energy (Δ*G*) at 298.15 K and 1 atm and the relative electronic energy with the zero point energy correction (Δ*E*).

**Fig. 2:**
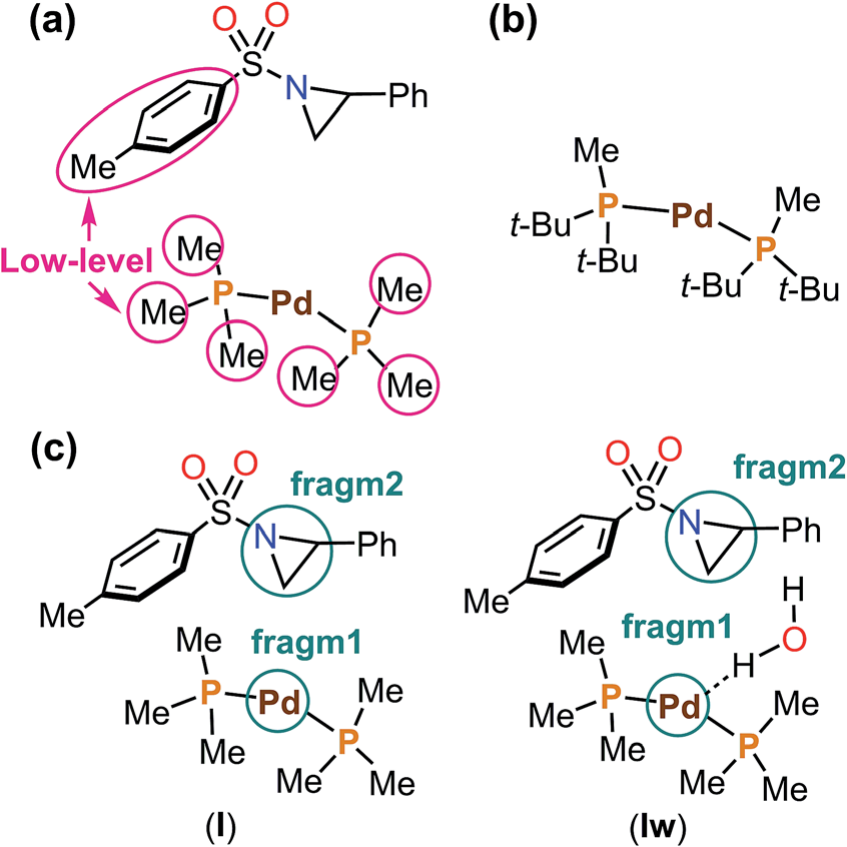
(a) ONIOM partitioning of the model complex into high- and low-levels, (b) the real complex, (c) the artificial force (*γ* = 300 kJ mol^–1^) was applied between **fragm1** (catalyst) and **fragm2** (substrate) without or with an explicit water molecule.

The aziridine ring opening step of the mechanism takes place *via* many different TSs, and therefore a proper sampling is very important. An automatic exploration of all important reaction pathways was accomplished using the multi-component artificial force induced reaction (MC-AFIR) method, as implemented in the global reaction route mapping (GRRM) strategy.^48^–^50^ An artificial force parameter (*γ*) of 300 kJ mol^–1^ was applied, and this is suitable for finding TSs within 300 kJ mol^–1^. The MC-AFIR calculations were terminated when no new AFIR LM was found for 10 consecutive AFIR paths (NFault = 10). In AFIR calculations, the energy and derivatives were obtained using the ONIOM(B3LYP-D3:PM6-D3) method.^51^–^57^ Partitioning of the molecular system is shown in Fig. 2a. A model catalyst was used for MC-AFIR calculations (Fig. 2). SDD basis sets and the associated effective core potentials were used for palladium, and 3-21G basis sets (BS3) were applied for the high-level region of ONIOM calculations.^58^–^60^ All AFIR paths were inspected and approximate TSs were identified. Then, the real phosphine ligand was introduced to all approximate TSs, and the TS structures were fully optimized (without artificial force) with B3LYP-D3/BS1 method. A Boltzmann distribution of the transition states was used to calculate the regioselectivity.

**Fig. 3:**
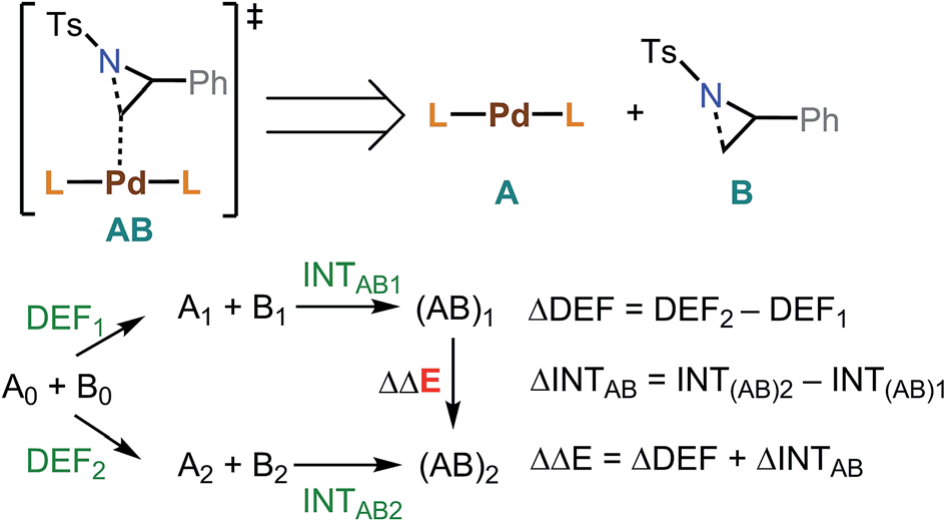
EDA between two optimized transition states leading to the desired and undesired products.

Energy decomposition analysis (EDA)^61^,^62^ was performed for the key TSs leading to the desired and the undesired products. B3LYP-D3/BS2 level and PCM were used for the EDA. In this analysis, a TS structure was divided into **A** (catalyst) and **B** (substrate) (Fig. 3). INT_AB_ is the interaction energy between **A** and **B** at their optimized TS structure. The deformation energy (DEF) is the energy of **A** and **B** at the optimized TS, relative to the optimized structures of isolated **A** and **B** (denoted as A_0_ and B_0_). The energy difference (ΔΔ*E*) between the optimized transition states, (**AB**)_1_ and (**AB**)_2_, is the sum of the internal energy difference (ΔINT_AB_) and the deformation energy difference (ΔDEF).

#### Thermodynamically stable complexes in solution

Our starting point was to explore thermodynamically stable complexes in solution. First, we checked the relative stability of PdL, PdL_2_, PdL_3_, and PdL_4_ complexes [L = P(*t*-Bu)_2_Me]. Among them, the PdL_2_ complex (**I**) is the most stable complex, and this is in agreement with our NMR studies. The PdL_3_ and PdL complexes are 3.4 and 26.0 kcal mol^–1^ higher in energy, respectively. Despite several attempts, no PdL_4_ complex was found, as one of the four ligands (L) always dissociated due to steric repulsion. We used PdL_2_ (**I**) as a candidate to generate other possible complexes in solution. Moreover, the two vacant sites of the PdL_2_ complex, A and B, can be filled by the potential ligands in solution, specifically H_2_O, solvent (denoted as Sol), and additive (denoted as bpy). Table 3 summarizes the possible complexes and energies relative to the PdL_2_ complex (**I**).

**Table 3:** Possible complexes Pd(L)_2_(A)(B) [L = P(*t*-Bu)_2_Me, Sol = MTBE] and their relative Δ*G* (Δ*E* in parentheses) in kcal mol^–1^^*a*^

A	B				
	Empty	H_2_O	bpy	L	Sol
Empty	0.0, (0.0)	–0.5, (–8.2)	4.0, (–7.4)	3.4, (–10.9)	X, (Sol)
H_2_O	—	4.7, (–14.0)	2.8, (–17.9)	7.9, (–15.1)	X, (Sol)
bpy	—	—	X, (bpy)	X, (L)	X, (Sol)
L	—	—	—	X, (L)	X, (Sol)
Sol	—	—	—	—	X, (Sol)

^*a*^The symbol “X” indicates that the ligand in parentheses dissociates upon structure optimization.

Starting from the PdL_2_ complex (**I**), coordination of one H_2_O, L, and bpy on **I** gives rise to PdL_2_(H_2_O) (**Iw**, –0.5 kcal mol^–1^), PdL_3_ (3.4 kcal mol^–1^), and PdL_2_(bpy) (4.0 kcal mol^–1^) complexes, respectively. Among the three-coordinate complexes, **Iw** is the thermodynamically most stable complex, and is only 0.5 kcal mol^–1^ more stable than **I**. Therefore, both **I** and **Iw** can be formed in solution. In **Iw**, an H_2_O molecule coordinates to the metal with one of the two hydrogen atoms. It is important to note that the additive (bpy) can coordinate to the metal, and the resulting complex, PdL_2_(bpy), is, however, 4.5 kcal mol^–1^ higher than the most stable complex, **Iw**. All four-coordinate complexes found in the calculations, PdL_2_(bpy)(H_2_O) (2.8 kcal mol^–1^), PdL_2_(H_2_O)_2_ (4.7 kcal mol^–1^), and PdL_3_(H_2_O) (7.9 kcal mol^–1^) are relatively higher in energy. Coordination of solvent (Sol) is not possible due to steric repulsion between the bulky groups of the solvent molecules (MTBE) and **I**. We concluded that the thermodynamically most stable complexes in solution are **Iw** and **I**. The general rules that lead to the formation of the thermodynamically stable complexes in solution are: (1) coordination of two ligands on PdL_2_, which gives rise to four-coordinate complexes, is not favorable; (2) in terms of making three-coordinate complexes, the binding preference of the third ligand follows the order H_2_O > L > bpy; (3) solvent MTBE would not coordinate to the PdL_2_ complex due to steric repulsion; (4) coordination of the additive (bpy) is not favorable. According to Table 3, additive coordination on PdL_2_ is not favorable as the subsequent complex PdL_2_(bpy) is 4.0 kcal mol^–1^ higher than PdL_2_, while the PdL_2_(bpy)_2_ complex is not stable. At the same time, it is important to note that aziridine binding on PdL_2_ is relatively easier (*vide infra*), and the corresponding adduct is only 2.6 kcal mol^–1^ higher than PdL_2_. Therefore, the additive would not coordinate to the PdL_2_ complex before the aziridine substrate binding. In our present mechanistic study, we do not consider the role of the additive in the mechanism because the reaction works even in the absence of additive (Table 1, entry 4).

#### Aziridine ring-opening step

**Fig. 4:**
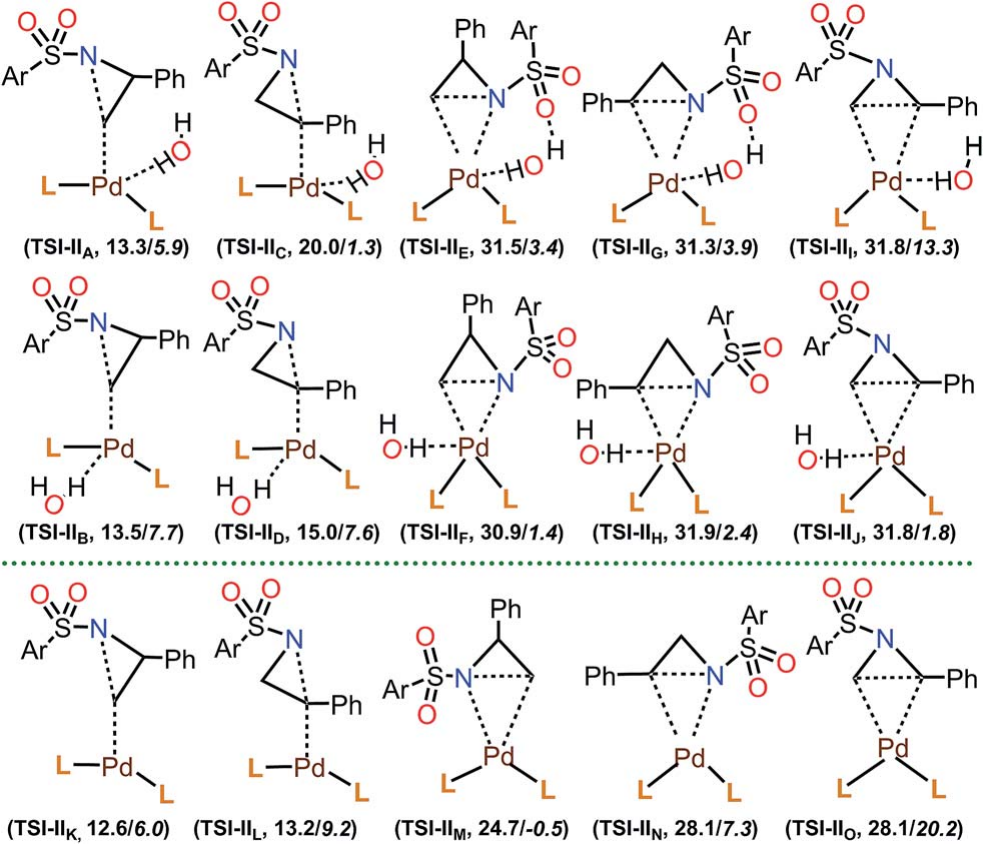
Groups of transition states for the aziridine ring opening, starting from **Iw** (**TSI-II_A_** through **TSI-II_J_**) and **I** (**TSI-II_K_** through **TSI-II_O_**), and energies of the lowest energy TS of each group relative to the PdL_2_(H_2_O) (**Iw**) complex (Ar = *p*-tolyl; L = P(*t*-Bu)_2_Me). Δ*G* values are in plain text, and Δ*E* values are in italics.

Our next task was the exploration of the aziridine ring-opening step. For this purpose, we used the most stable **I** and **Iw** complexes as the active intermediates for the reaction, and 2-phenyl-*N*-tosyl-aziridine (**1**) was used as the substrate. Therefore, two independent MC-AFIR calculations, with and without an explicit water molecule, were performed as shown in Fig. 2c. The fully optimized TS structures, 73 TSs from **Iw** and 33 TSs from **I**, were categorized into 15 groups: **TSI-II_A_** through **TSI-II_J_** from **Iw**, and **TSI-II_K_** through **TSI-II_O_** from **I**, based on structural similarities (Fig. 4). The lowest energy TS of each group, their relative energies, and their existence probability are depicted in Table 4 (see Table S14 in the ESI† for a full description of all the TSs).

**Table 4:** Low energy TSs for the aziridine ring opening starting from **I** and **Iw**^*a*^

TS	Group/regioselectivity	ΔΔ*G*	Δ*G* (Δ*E*)	Existence probability (%)
**TS1-t-lw**	K/terminal	0.0	12.6 (6.0)	27.6
**TS2-t-lw**	K/terminal	0.3	12.9 (5.4)	17.9
**TS3-b-lw**	L/benzylic	0.6	13.2 (9.2)	10.0
**TS4-t-l**	A/terminal	0.7	13.3 (5.9)	9.6
**TS5-t-lw**	K/terminal	0.8	13.4 (6.9)	7.1
**TS6-t-l**	B/terminal	0.9	13.5 (7.7)	6.1
**TS7-t-l**	B/terminal	1.0	13.6 (7.7)	4.8
**TS8-t-l**	A/terminal	1.1	13.7 (6.8)	4.6
**TS9-t-lw**	K/terminal	1.3	13.9 (7.6)	2.9
**TS10-t-lw**	K/terminal	2.3	14.9 (7.6)	0.6
**TS11-b-l**	D/benzylic	2.4	15.0 (7.6)	0.5
**TS12-t-lw**	K/terminal	2.6	15.2 (–1.2)	0.4
**TS13-t-lw**	K/terminal	2.6	15.2 (8.3)	0.3
**TS14-t-lw**	K/terminal	2.7	15.3 (7.5)	0.3
**TS15-t-lw**	K/terminal	3.3	15.9 (9.0)	0.1
**TS16-t-lw**	K/terminal	3.5	16.1 (8.3)	0.1
**TS17-t-lw**	K/terminal	3.6	16.2 (7.9)	0.1
**TS18-t-l**	B/terminal	3.7	16.3 (9.2)	0.1
**TS19-t-l**	A/terminal	3.7	16.3 (–0.8)	0.1

^*a*^Δ*G* and Δ*E* values are indicated in kcal mol^–1^, relative to **Iw**. “t” and “b” indicate the terminal (3-position) and benzylic carbons (2-position), respectively.

Among the calculated TSs (Table 4), **TS1-t-lw** (12.6 kcal mol^–1^) which belongs to the **TSI-II_K_** group is the lowest energy TS of this step. In this TS, the aziridine ring opening takes place at the less hindered carbon in an S_N_2 fashion, leading to the desired product forming. The same product can be obtained through **TS2-t-lw** (12.9 kcal mol^–1^), **TS4-t-l** (13.3 kcal mol^–1^), **TS5-t-lw** (13.4 kcal mol^–1^), **TS6-t-l** (13.5 kcal mol^–1^), **TS7-t-l** (13.6 kcal mol^–1^), **TS8-t-l** (13.7 kcal mol^–1^), and **TS9-t-lw** (13.9 kcal mol^–1^). On the other hand, at **TS3-b-lw** (13.2 kcal mol^–1^) of the group **TSI-II_L_**, aziridine ring opening occurs at the hindered carbon (the 2-position), and this is the lowest TS leading to the undesired product. Based on a Boltzmann distribution over the calculated TSs, the regioselectivity is calculated to be 89 : 11, which is qualitatively in agreement with the experimental results (99 : 1).

In the calculated TSs for **TSI-II_E_** (31.5 kcal mol^–1^), **TSI-II_F_** (30.9 kcal mol^–1^), and **TSI-II_M_** (24.7 kcal mol^–1^), aziridine ring opening occurs at the less hindered carbon (Fig. 4). However, these TSs are relatively higher in energy due to the fact that the *N-p*-toluenesulfonyl and 2-phenyl substituents of the substrate are much closer to the bulky groups of **Iw** or **I**. Similarly, TSs in the group of **TSI-II_G_** (31.3 kcal mol^–1^), **TSI-II_H_** (31.9 kcal mol^–1^), and **TSI-II_N_** (28.1 kcal mol^–1^) show high energy barriers, where aziridine ring opening occurs at the hindered carbon. TSs in the group of **TSI-II_I_** (31.8 kcal mol^–1^), **TSI-II_J_** (31.8 kcal mol^–1^), and **TSI-II_O_** (28.1 kcal mol^–1^) represent the aziridine ring opening through the carbon–carbon bond, which is, however, not possible due to very large reaction barriers. Furthermore, we checked the effect of H_2_O molecules on aziridine ring opening. Starting from the lowest energy TS for this step (**TS1-t-lw**, 12.6 kcal mol^–1^), up to four H_2_O molecules were introduced, and the corresponding TSs were calculated. The calculated Δ*G* of **TS1-t-lw** with one H_2_O molecule (**TS1-t-lw-w**, 16.1 kcal mol^–1^), two H_2_O molecules (**TS1-t-lw-w_2_**, 14.3 kcal mol^–1^), three H_2_O molecules (**TS1-t-lw-w_3_**, 15.6 kcal mol^–1^), and four H_2_O molecules (**TS1-t-lw-w_4_**, 16.4 kcal mol^–1^) suggested that explicit H_2_O molecules in the system would not stabilize the **TS1-t-lw**.

**Fig. 5:**
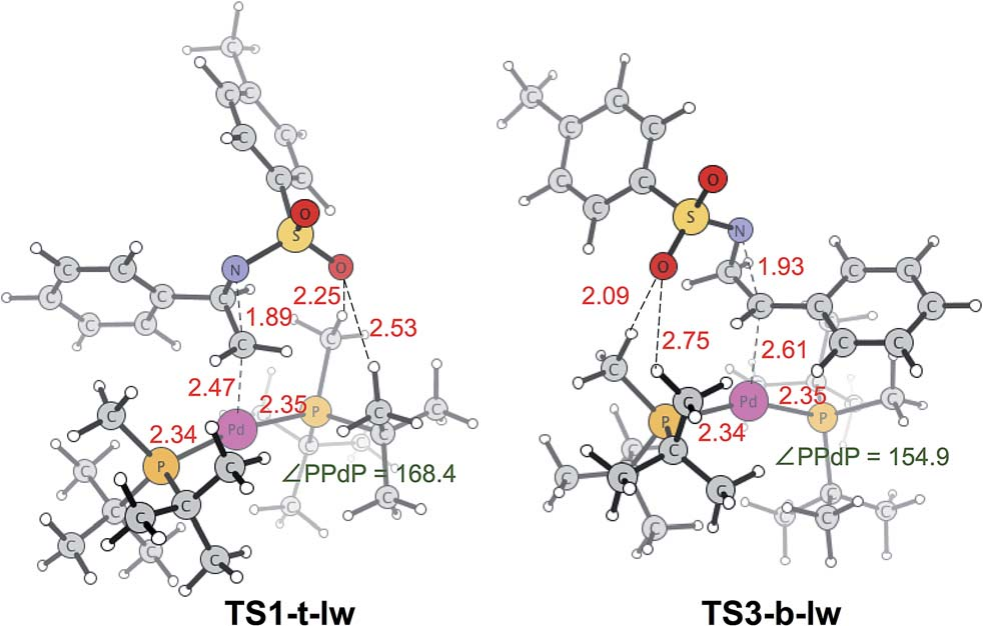
Lowest energy TSs leading to the desired product (**TS1-t-lw**) and undesired product (**TS3-b-lw**). Bond lengths (red) are in Å and bond angles (green) are in degrees.

**TS1-t-lw** is a major contributor to the aziridine ring opening at the less hindered carbon, while **TS3-b-lw** is the lowest TS for aziridine ring opening at the hindered carbon (Fig. 5). The calculated Gibbs free energy difference between **TS1-t-lw** and **TS3-b-lw** is 0.6 kcal mol^–1^, while the potential energy difference is 3.2 kcal mol^–1^. When we do not consider the zero point energy corrections, the potential energy difference is 3.5 kcal mol^–1^, and we have used this value for the EDA. According to the EDA (Table 5), the origin of this difference comes from ΔINT_AB_ (3.9 kcal mol^–1^), indicating better interactions at **TS1-t-lw**. This is due to the fact that the aziridine substrate can approach closer to the metal in **TS1-t-lw** (Pd–C = 2.47 Å) (*vs.* Pd–C = 2.61 Å at **TS3-b-lw**) (Fig. 5) than in **TS3-b-lw** due to the lower steric repulsion.

**Table 5:** EDA for the potential energy difference (kcal mol^–1^) between the key transition states, **TS1-t-lw** and **TS3-b-lw**

TS	DEF (DEF_A_, DEF_B_)	INT_AB_	Δ*E*
**TS1-t-lw**	23.2 (1.2, 22.0)	–26.5	–3.3
**TS3-b-lw**	22.8 (2.7, 20.1)	–22.6	0.2
	**ΔDEF**	**ΔINT_AB_**	**ΔΔ*E***
	–0.4 (–1.5, 1.1)	3.9	3.5

**Fig. 6:**
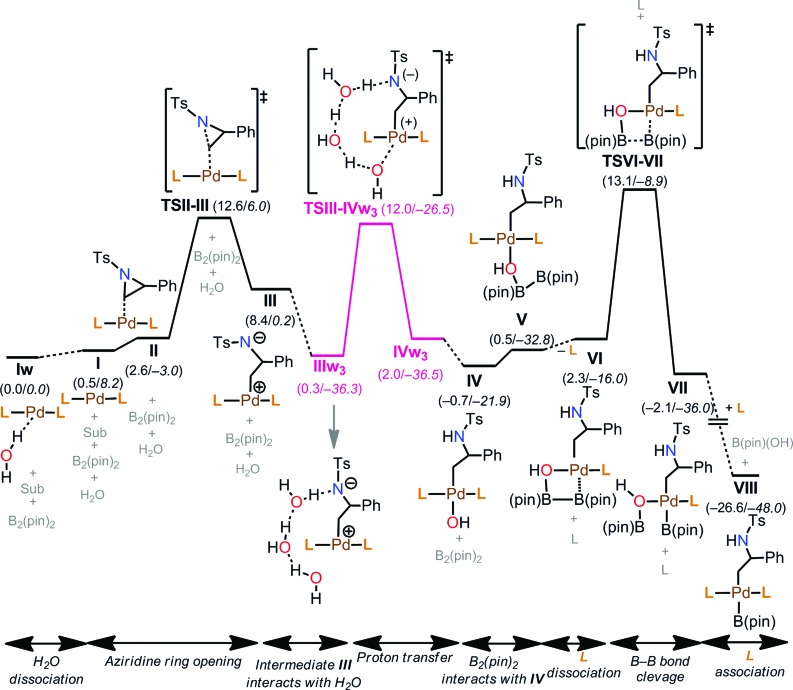
Free energy profile for the reaction mechanism (aziridine ring opening, proton transfer, and B–B bond cleavage processes). Energies are indicated in kcal mol^–1^. Δ*G* values are in plain text, and Δ*E* values are in italics.

The free energy profile for the early stages of the mechanism is shown in Fig. 6. The reaction starts from the thermodynamically most stable complex, **Iw**. Therefore, we report energies relative to **Iw**. Then, aziridine coordination on **I** leads to a complex **II** (2.6 kcal mol^–1^), and the subsequent aziridine ring opening occurs through **TS1-t-lw** (**TSII–III**, 12.6 kcal mol^–1^). Aziridine ring opening is also possible from **Iw** (not shown in Fig. 6) with an overall barrier of 13.3 kcal mol^–1^ (**TS4-t-lw**), which is, however, 0.7 kcal mol^–1^ higher than **TS1-t-lw**. Beyond **TS1-t-lw**, an intermediate, **III** (8.4 kcal mol^–1^) is formed.

#### Proton transfer to anionic amine

The next step of the mechanism would be a proton transfer from H_2_O molecules in solution to the anionic nitrogen atom in the water-coordinated intermediate **III**. This proton transfer process takes place smoothly from **IIIw_3_** (0.3 kcal mol^–1^), which is 8.1 kcal mol^–1^ more stable than **III**. Furthermore, three H_2_O molecules make a hydrogen bonding network starting from the anionic amine that is connected to the metal through a water oxygen coordination. The TS for this proton transfer process is 12.0 kcal mol^–1^ (**TSIII–IVw_3_**). Proton transfer is also possible from the analogous intermediate with four H_2_O molecules, **IIIw_4_** (11.3 kcal mol^–1^), and the subsequent transition state is, however, 1.3 kcal mol^–1^ higher than **TSIII–IVw_3_** (not shown in Fig. 6). We were unable to locate TSs starting from the analogous intermediates with two H_2_O molecules (**IIIw_2_**, 1.1 kcal mol^–1^) and one H_2_O molecule (**IIIw**, 2.3 kcal mol^–1^), where the potential energy surface for the proton transfer processes was found to be repulsive (Fig. S2 and S3†). In **IIIw_3_**, three water molecules form a complete head-to-tail chain between the anionic amines and Pd centres. As a result, the synchronous three proton transfer *via***TSIII–IVw_3_** takes place and gives a protonated N, two H_2_O molecules, and a hydroxyl group bound to the metal center. Based on our analysis, we conclude that three H_2_O molecules are required for the proton transfer process, leading to an intermediate **IVw_3_** (2.0 kcal mol^–1^). Once the proton is transferred, the chain of water molecules is no longer required, and the corresponding intermediate without H_2_O, **IV**, is 2.7 kcal mol^–1^ more stable than **IVw_3_**.

#### B–B bond coordination and cleavage

Now, the B(pin)–B(pin) species binds to **IV** and the subsequent intermediate **V** (0.5 kcal mol^–1^) is formed. Starting from **V**, inner-sphere B–B bond cleavage does not take place due to steric repulsion between the bulky alkyl groups in L and B(pin)–B(pin). One of the two phosphine ligands of the catalyst must dissociate, and the resulting intermediate, **VI**, is only 1.8 kcal mol^–1^ higher than **V**. Then, B–B bond cleavage takes place in an inner sphere fashion with a barrier of 10.8 kcal mol^–1^ (**TSVI–VII**), giving rise to **VII** (–2.1 kcal mol^–1^). We have checked the possibility of ligand exchange between the (pin)B–OH and a stronger phosphine ligand L on intermediate **VII**, and the resulting complex, **VIII**, is 24.5 kcal mol^–1^ more stable than **VII**.

#### *cis–trans* isomerization and reductive elimination

**Fig. 7:**
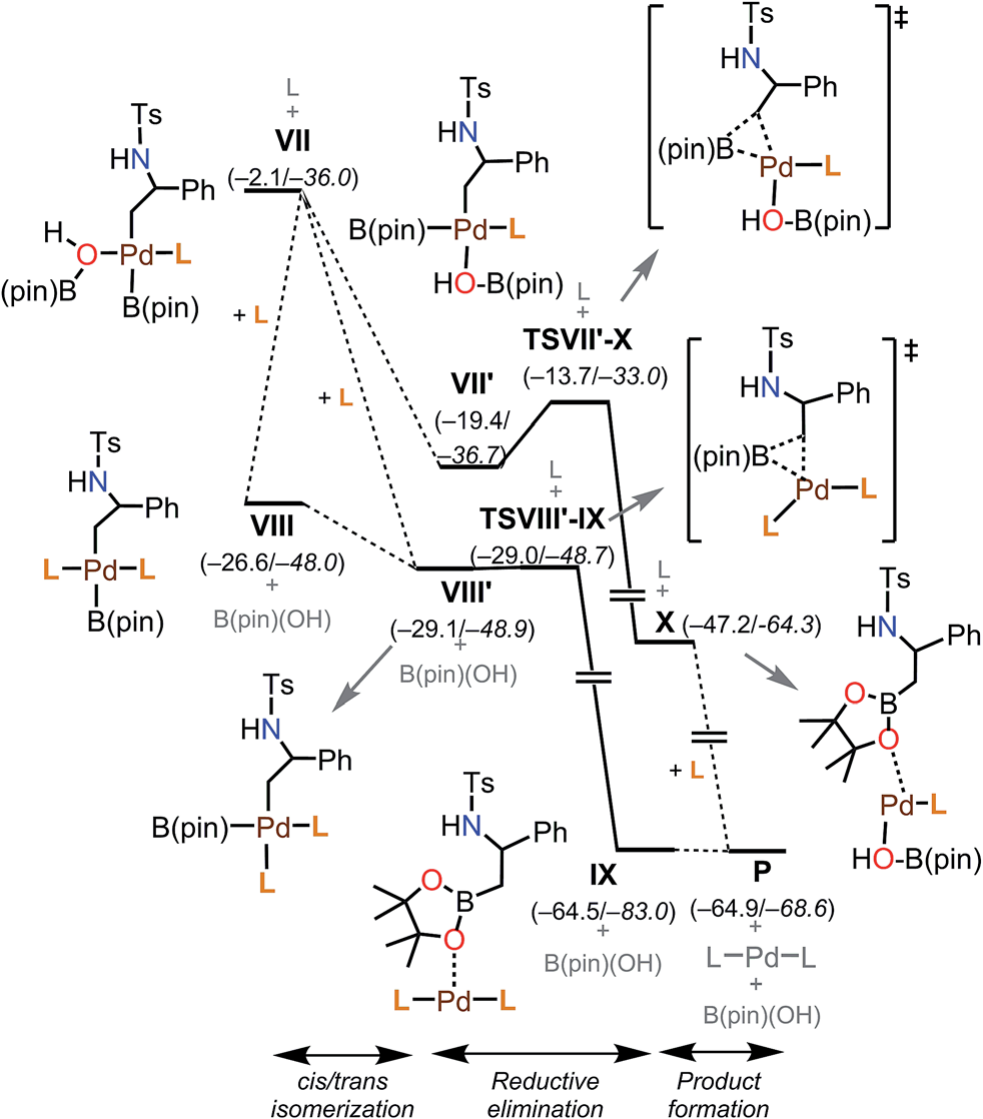
Free energy profile for the later steps of the mechanism. Energies are indicated in kcal mol^–1^, Δ*G* values are in plain text, and Δ*E* values are in italics.

The energy profiles for the final stages of the reaction are shown in Fig. 7. In both the intermediates **VII** and **VIII**, B(pin) is *trans* to the alkyl group derived from the aziridine substrate, and therefore *cis*/*trans* isomerization must take place before the reductive elimination. The *cis* isomer **VIII′** is 2.5 kcal mol^–1^ more stable than the *trans* isomer **VIII**. This step may occur through ligand dissociation processes. Since we expect this isomerization to be a low energy process, we did not study this in depth. The subsequent reductive elimination from **VIII′** occurs through a barrier of 0.1 kcal mol^–1^ (**TSVIII′–IX**). In the resulting intermediate **IX**, the product (**P**) is still at the metal coordination sphere, and **P** can be removed easily to recover the active form of the catalyst, PdL_2_ (**I**), for the next catalytic cycle. Similarly, the formation of the *cis* isomer of **VII**, the less stable intermediate **VII′** (–19.4 kcal mol^–1^), and the subsequent reductive elimination can occur easily with a barrier of 5.7 kcal mol^–1^ (**TSVII′–X**). Then, a phosphine ligand (L) can coordinate to the resulting intermediate **X**, which ultimately yields the product, **P**.

### Side product formation

Under the optimized reaction conditions, imine **6** is formed as a side product (Table 1). We have explored the mechanism for the side reaction as shown in Fig. S4 in the ESI.†

### Catalytic cycle

**Fig. 8:**
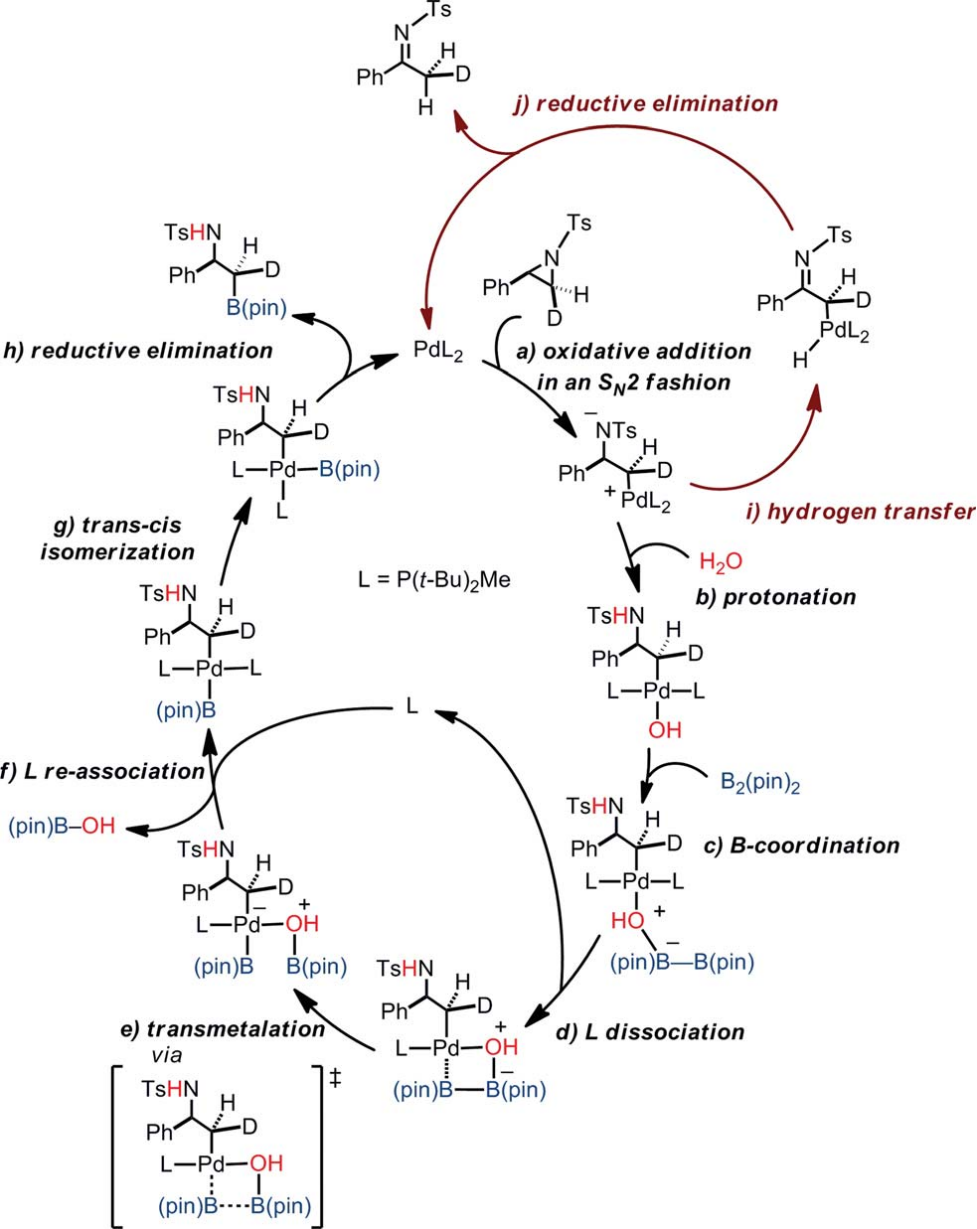
A proposed catalytic cycle of the borylation.

Putting together the present results from the experimental and theoretical studies, the proposed catalytic cycle for the borylation is shown in Fig. 8 (black cycle). Pd(0)L_2_ is generated through the sequential reduction of Cp(allyl)Pd. Then, Pd(0)L_2_ attacks the less hindered carbon of the aziridine in an S_N_2 fashion (a) to give a stereo-inverted oxidative adduct. A hydrogen bonded chain of H_2_O molecules plays two roles in the following steps: (i) as a proton source to quench TsN^–^ (b) and (ii) as an internal base to form the [Pd(OH)] species. This intermediate activates the B–B bond of B_2_(pin)_2_ (c). The dissociation of a phosphine ligand (d) facilitates the transmetalation (e). The free phosphine ligand participates in the catalytic cycle again to form the (alkyl)PdL_2_B(pin) complex (f). The *trans*–*cis* isomerization (g) followed by reductive elimination (h) leads to the C–B cross-coupled product and the completion of the catalytic cycle. A side reaction may occur after the oxidation addition (brown cycle): the oxidative adduct of aziridine undergoes hydrogen transfer (i) followed by reductive elimination (j) to produce an imine byproduct. Our computational study suggests that the aziridine ring-opening step for substrate **1a** has barriers of 12.6 kcal mol^–1^ (terminal position) and 13.2 kcal mol^–1^ (benzylic position) (Table 4). For an alkyl aziridine, in particular **1l**, the aziridine ring-opening step showed barriers of 15.4 kcal mol^–1^ (2-position) and 16.3 kcal mol^–1^ (3-position). In the absence of the aryl group, the aziridine ring opening of **1l** is 2.8 kcal mol^–1^ higher than **1**, where the reaction is difficult with **1l**. This is qualitatively in agreement with our experimental results, where the reaction does not proceed with **1l**.

## Conclusions

In conclusion, we have developed a Pd-catalyzed regioselective borylative ring opening reaction of 2-arylaziridines to give β-aminoalkylboronates that are otherwise difficult to synthesize using existing methodologies. Importantly, the regioselectivity of the ring opening is controlled by the interactions between the catalyst and the substrate. The S_N_2 nature of the oxidative addition of aziridine was verified using deuterated aziridine and computational studies. Furthermore, the borylative reaction is applicable under neutral conditions that allow for high functional compatibility. The mechanism of the full catalytic cycle was proposed using DFT and MC-AFIR methods. The aziridine ring opening is initiated by the active species PdL_2_. TSs were systematically determined for the selectivity-determining aziridine ring opening step, and the calculations reasonably reproduced the experimental regioselectivity. The next step of the mechanism is a proton transfer that is facilitated by a H_2_O hydrogen bond chain. The resulting Pd-hydroxo species activates the transmetalation step, where inner sphere boron–boron bond cleavage occurs, and leads to the final reductive elimination step. These experimental and theoretical findings open up an avenue to the further development of transition metal-catalyzed ring-opening C–E bond forming cross couplings of aziridines.

## Supplementary Material

Supplementary information
