# Supplementary material for: Palladium-catalyzed regioselective and stereo-invertive ring-opening borylation of 2-arylaziridines with bis(pinacolato)diboron: experimental and computational studies
Source: Chem Sci. 2016 Jun 9;7(9):6141–52. doi: 10.1039/c6sc01120a (PMC6024180; doi:10.1039/c6sc01120a)
Supplement: Supplementary file 1 [file SC-007-C6SC01120A-s001.pdf]

Electronic Supplementary Material (ESI) for Chemical Science.
This journal is © The Royal Society of Chemistry 2016

Electronic Supplementary Material (ESI) for

Palladium-Catalyzed Regioselective Ring-Opening Borylation of 2-Arylaziridines with Bis(pinacolato)diboron: Experimental and Computational Studies

Youhei Takeda,*,† Akinobu Kuroda,† W. M. C. Sameera,‡ Keiji Morokuma,*,‡ and Satoshi Minakata*,†

† Department of Applied Chemistry, Graduate School of Engineering, Osaka University, Yamadaoka 2-1, Suita, Osaka 565-0871, Japan

‡ Fukui Institute for Fundamental Chemistry, Kyoto University, Takano-Nishiraki-cho, 34-4, Sakyo-ku, Kyoto 606-8103, Japan

E-mail: takeda@chem.eng.osaka-u.ac.jp; morokuma.keiji.3a@kyoto-u.ac.jp; minakata@chem.eng.osaka-u.ac.jp

Table of Contents

Experimental Part

General Remarks S2
Preparation of 2-Arylaziridines S2–S5
Optimization Studies of Reaction Conditions S5–S16
A Typical Procedure for Pd-catalyzed Borylation S16
A Procedure for Gram-scale Preparation of **3a** S17
Spectroscopic Data of Borylated Products S17–S20
Derivatization of Aminoboronates S21–23
Deuterium-Labeling Experiments S23–S24
NMR experiments S25
Procedures for the borylation of **1a** using the Pd catalyst generated in toluene S26
^{1}H and ^{13}C NMR Spectra of New Compounds S27–S46
Chiral HPLC Charts S47–S49
References S50

Computational Part

TSs for the Aziridine Ring Opening Starting from **I** and **Iw** S51–S53
Relaxed Potential Energy Surfaces for the Protonation of Intermediate **IIIw** and **IIIw$_2$** S53–S54
Side Reaction S54–S55
Energies of Structures S55–S59
Cartesian Coordinates of the Optimized Structures S60–S113

Experimental Part

General Remarks

All reactions were carried out under an atmosphere of nitrogen using standard Schlenk technique or glove box. Melting points were determined on a Stanford Research Systems MPA100 OptiMelt Automated Melting Point System. Infrared spectra were acquired on a SHIMADZU IRAffinity-1 FT-IR Spectrometer. All ^{1}H, ^{13}C and ^{31}P NMR spectra were recorded on a JEOL JMTC-400/54/ss NMR Spectrometer (^{1}H NMR, 400 MHz; ^{13}C NMR, 100 MHz; ^{31}P NMR, 162 MHz), and chemical shifts were referenced to the signal of an internal standard (tetramethylsilane, $\delta = 0$ ppm for ^{1}H and ^{13}C NMR measurements) or an external standard (85% H_3PO_4 qa. for ^{31}P NMR measurement). Chiral-phase high-performance liquid chromatography (HPLC) was performed on a SHIMADZU prominence series instruments equipped with chiral columns. Low- and high-resolution mass spectra were obtained on a JEOL JMS-DX303HF mass spectrometer. Analytical thin-layer chromatography (TLC) was performed on pre-coated silica gel glass plates (Merck silica gel 60 F_{254}, 0.25 mm thickness), and compounds were visualized with UV lamp or treatment with an ethanolic solution of phosphomolybdic acid followed by heating. Products were purified by flash column chromatography on a silica gel BW-300 (Fuji Silysia Chemical Ltd).

Materials. All solvent was purchased from commercial sources and used after distillation. Styrene derivatives, bis(pinacolate)diborone, P(*t*-Bu)$_2$Me and other commercial reagents were purchased from Sigma Aldrich or TCI and used as received. Cp(allyl)Pd[S1] and Cp(cinnamyl)Pd[S2] were prepared according to previously published procedures.

Preparation of 2-Arylaziridines. All aziridines **1a**–**1u** were prepared according to the procedures reported in literature, and their spectroscopic data were in agreement with those previously reported as follows:

2-Phenyl-1-tosylaziridine (1a) [CAS No. 24395-14-0]

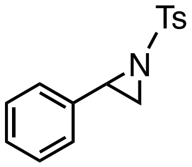

Prepared through aziridination of styrene according to the procedure described in literature;[S3a] Spectroscopic data were in agreement with those previously reported;[S4a] Purified by recrystallization from MeOH; 74% yield.

(*R*)-2-Phenyl-1-tosylaziridine [(*R*)-1a] [CAS No. 62596-62-7]

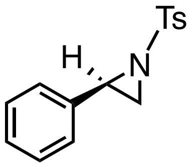

Prepared from (*R*)-phenylglycinol through cyclization according to the procedure described in literature;[S3b] Spectroscopic data were in agreement with those previously reported;[S4a] Purified by silica gel column chromatography (hexane/EtOAc 99:1 to 7:3); 90% yield, 99% ee (HPLC); HPLC (Chiralcel OJ; 1.0 mL/min; *i*-PrOH/*n*-hexane 30:70; $\lambda = 254$ nm): t_R 19.1 min.

***cis*-2-Deuterium-2-phenyl-1-tosylaziridine (*cis*-1a-*d*$_1$)** [CAS No. 320750-88-7]

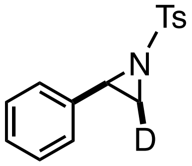

Prepared according to the procedure described in literature;[S3c] Spectroscopic data were in agreement with those previously reported;[S3c] Purified by silica gel column chromatography (hexane/EtOac, 99:1 to 8:2); 90% yield.

2-(*p*-Tolyl)-1-tosylaziridine (1b) [CAS No. 97401-87-1]

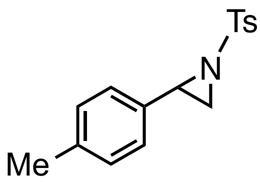

Prepared through aziridination of 1-methyl-4-vinylbenzene according to the procedure described in literature;[S3d] Spectroscopic data were in agreement with those previously reported;[S4b] Purified by silica gel column chromatography (hexane/EtOAc 99:1 to 7:3) followed by recrystallization from EtOAc; 61% yield.

2-(*m*-Tolyl)-1-tosylaziridine (1c) [CAS No. 403518-39-8]

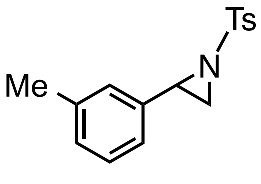

Prepared through aziridination of 1-methyl-3-vinylbenzene according to the procedure described in literature;[S3d] Spectroscopic data were in agreement with those previously reported;[S4b] Purified by silica gel column chromatography (hexane/EtOAc 99:1 to 7:3) followed by recrystallization from EtOAc; 42% yield.

2-(*o*-Tolyl)-1-tosylaziridine (1d) [CAS No. 1111321-35-7]

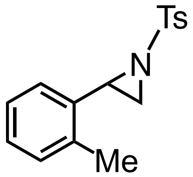

Prepared through aziridination of 1-methyl-2-vinylbenzene according to the procedure described in literature;[S3d] Spectroscopic data were in agreement with those previously reported;[S4b] Purified by silica gel column chromatography (hexane/EtOAc 99:1 to 7:3) followed by recrystallization from EtOAc; 36% yield.

2-(4-Fluorophenyl)-1-tosylaziridine (1e) [CAS No. 250260-25-4]

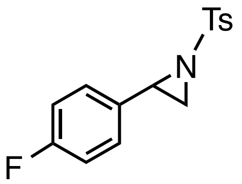

Prepared through aziridination of 1-fluoro-4-vinylbenzene according to the procedure described in literature;[S3d] Spectroscopic data were in agreement with those previously reported;[S4b] Purified by silica gel column chromatography (hexane/EtOAc 99:1 to 7:3) followed by recrystallization from EtOAc; 61% yield.

2-(4-Chlorophenyl)-1-tosylaziridine (1f) [CAS No. 97401-93-9]

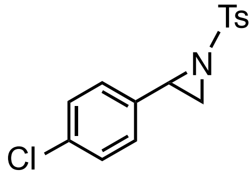

Prepared through aziridination of 1-fluoro-4-vinylbenzene according to the procedure described in literature;[S3d] Spectroscopic data were in agreement with those previously reported;[S4a] Purified by silica gel column chromatography (hexane/EtOAc 99:1 to 7:3) followed by recrystallization from EtOAc; 53% yield.

1-Tosyl-2-(4-(trifluoromethyl)phenyl)aziridine (1g) [CAS No. 250260-27-6]

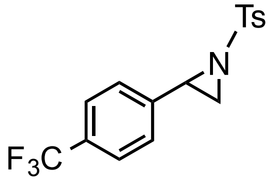

Prepared through aziridination of 1-(trifluoromethyl)-4-vinylbenzene according to the procedure described in literature;[S3d] Spectroscopic data were in agreement with those previously reported;[S4b] Purified by silica gel column chromatography (hexane/EtOAc 99:1 to 7:3) followed by recrystallization from EtOAc; 65% yield.

2-(4-Nitrophenyl)-1-tosylaziridine (1h) [CAS No. 155721-37-2]

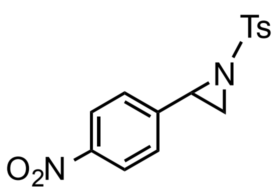

Prepared through aziridination of 1-nitro-4-vinylbenzene according to the procedure described in literature;[S3d] Spectroscopic data were in agreement with those previously reported;[S4a] Purified by silica gel column chromatography (hexane/EtOAc 99:1 to 7:3) followed by recrystallization from EtOAc; 67% yield.

4-(1-Tosylaziridin-2-yl)phenyl acetate (1i) [CAS No. 250260-26-5]

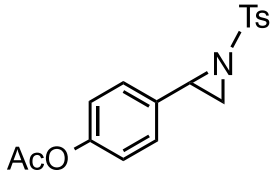

Prepared through aziridination of 4-vinylphenyl acetate according to the procedure described in literature;[S3d] Spectroscopic data were in agreement with those previously reported;[S4b] Purified by silica gel column chromatography (hexane/EtOAc 99:1 to 7:3) followed by recrystallization from EtOAc; 48% yield.

Methyl 4-(1-tosylaziridin-2-yl)benzoate (1j) [CAS No. 1365842-12-1]

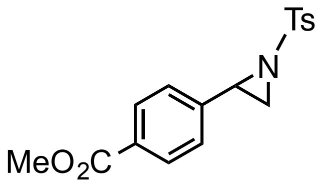

Prepared through aziridination of methyl 4-vinylbenzoate according to the procedure described in literature;[S3d] Spectroscopic data were in agreement with those previously reported;[S4c] Purified by silica gel column chromatography (hexane/EtOAc 99:1 to 7:3) followed by recrystallization from EtOAc; 72% yield.

2-(1-Tosylaziridin-2-yl)pyridine (1k) [CAS No. 796975-18-3]

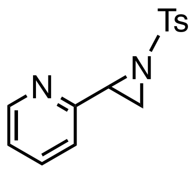

Prepared through aziridination of 2-vinylpyridine according to the procedure described in literature;[S3e] Spectroscopic data were in agreement with those previously reported;[S3d] Purified by silica gel column chromatography (hexane/EtOAc 99:1 to 7:3) followed by recrystallization from EtOAc; 18% yield.

2-Butyl-1-tosylaziridine (1l) [CAS No. 116905-61-4]

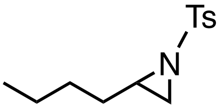

Prepared through aziridination of 1-hexene according to the procedure described in literature;[S3g] Spectroscopic data were in agreement with those previously reported;[S4d] Purified by silica gel column chromatography (hexane/EtOAc 99:1 to 7:3); 42% yield.

2-Benzyl-1-tosylaziridine (1m) [CAS No. 71535-50-7]

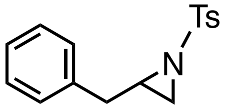

Prepared through aziridination of allylbenzene according to the procedure described in literature;[S3h] Spectroscopic data were in agreement with those previously reported;[S3g] Purified by silica gel column chromatography (hexane/EtOAc 99:1 to 7:3) followed by recrystallization from EtOAc; 86% yield.

2-(But-3-en-1-yl)-1-tosylaziridine (1n) [CAS No. 176516-06-6]

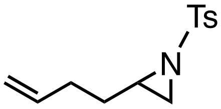

Prepared through aziridination of 1,5-hexadiene according to the procedure described in literature;[S3i] Spectroscopic data were in agreement with those previously reported;[S3i] Purified by silica gel column chromatography (hexane/EtOAc 99:1 to 7:3); 51% yield.

2-Methyl-2-phenyl-1-tosylaziridine (1o) [CAS No. 137595-22-3]

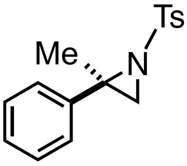

Prepared through aziridination of α-methylstyrene according to the procedure described in literature;[S3e] Spectroscopic data were in agreement with those previously reported;[S3e] Purified by silica gel column chromatography (hexane/EtOAc 99:1 to 7:3) followed by recrystallization from EtOAc; 12% yield.

***trans*-2-Methyl-3-phenyl-1-tosylaziridine (1p)** [CAS No. 137595-21-2]

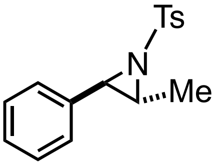

Prepared through aziridination of (*E*)-propenylbenzene according to the procedure described in literature;[S3f] Spectroscopic data were in agreement with those previously reported;[S3f] Purified by silica gel column chromatography (hexane/EtOAc 99:1 to 7:3); 51% yield.

1-Tosyl-1a,2,3,7b-tetrahydro-1*H*-naphtho[1,2-*b*]azirine (1q) [CAS No. 137595-23-4]

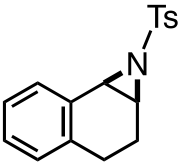

Prepared through aziridination of 1,2-dihydronaphthalene according to the procedure described in literature;[S3c] Spectroscopic data were in agreement with those previously reported;[S4d] Purified by silica gel column chromatography (hexane/EtOAc 99:1 to 7:3) followed by recrystallization from EtOAc; 68% yield.

1-((4-Methoxyphenyl)sulfonyl)-2-phenylaziridine (1r) [CAS No. 181306-57-0]

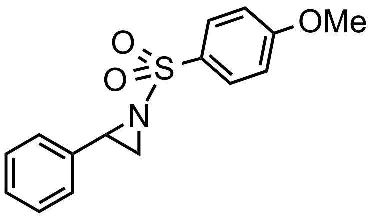

Prepared through aziridination of styrene according to the procedure described in literature;[S3j] Spectroscopic data were in agreement with those previously reported;[S4e] Purified by silica gel column chromatography (hexane/EtOAc 99:1 to 8:2) followed by recrystallization from EtOAc; 48% yield.

1-(*tert*-Butylsulfonyl)-2-phenylaziridine (1s) [CAS No. 238747-35-8]

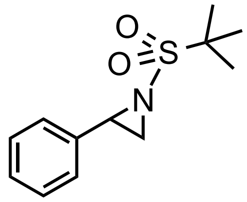

Prepared through aziridination of styrene according to the procedure described in literature;[S4c] Spectroscopic data were in agreement with those previously reported;[S4c] Purified by silica gel column chromatography (hexane/EtOAc 99:1 to 8:2) followed by recrystallization from EtOAc; 32% yield.

1-((4-Nitrophenyl)sulfonyl)-2-phenylaziridine (1t) [CAS No. 175222-83-0]

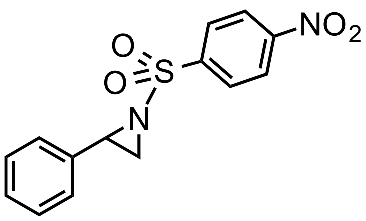

Prepared through aziridination of styrene according to the procedure described in literature;[S3k] Spectroscopic data were in agreement with those previously reported;[S4e] Purified by silica gel column chromatography (hexane/EtOAc 99:1 to 8:2) followed by recrystallization from EtOAc; 61% yield.

5-Methyl-2-((2-phenylaziridin-1-yl)sulfonyl)pyridine (1u) [CAS No. 796034-71-4]

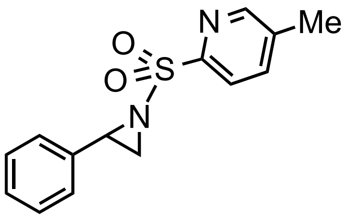

Prepared through aziridination of styrene according to the procedure described in literature;[S3l] Spectroscopic data were in agreement with those previously reported;[S4e] Purified by silica gel column chromatography (hexane/EtOAc 99:1 to 8:2) followed by recrystallization from EtOAc; 45% yield.

Optimization Studies of Reaction Conditions

1. Effect of Pd catalyst and ligand

A typical procedure for the screening of Pd catalyst and ligand. In a glove box, to a 3 mL vial with a magnetic stir (10 mm) bar were added Pd catalyst (1 mol% of Pd), Ligand (2 mol%) and MTBE (550 μL), and the resulting solution was stirred at 60 °C for 5 min on an aluminum heating block. After allowing the vial cool to room temperature, *N*-tosyl-2-phenylaziridine (54.6 mg, 0.20 mmol) and $B_2(pin)_2$ (60.9 mg, 0.24 mmol, 1.2 equiv) were added. The vial was capped with a hole cap and a Teflon®/rubber septum and removed from the glove box. Deionized H_2O (50 μL) was added through the septum under a stream of N_2 gas, and the resulting mixture was stirred at 60 °C on an aluminum heating block for 3 h. *n*-Dodecane (34.1 mg, 0.20 mmol, 1.0 equiv, as an internal standard for GC analysis) was added to the vial through the septum, and the mixture was diluted with acetone and subjected to GC analysis. Yields of products were estimated based on a calibration curve method.

Table S1. Effect of Pd catalyst and ligand

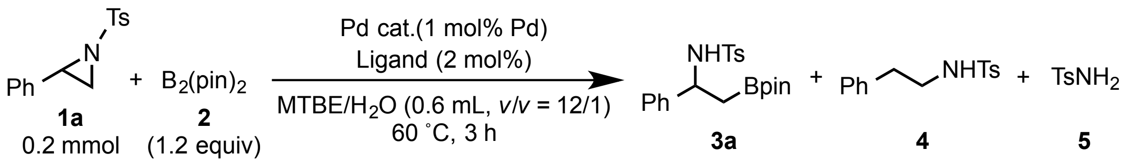

entry	Pd cat.	Ligand	yield (%)[a] 3a	4	5	recovery of 1a (%)[a]
1	–	$Pd(PPh_3)_4$	5	17	73	0
2	–	$Pd(P(o\text{-tol})_3)_2$	4	4	0	90
3	$Pd_2(dba)_3$	PCy_3	5	10	0	85
4	$Pd_2(dba)_3$	$PCyp_3$	7	0	29	37
5	$Pd_2(dba)_3$	$P(t\text{-Bu})_2Me$	69	16	8	0
6	$Pd(P(t\text{-Bu})_3)_2$	–	0	0	0	97
7	$Pd_2(dba)_3$	PMe_3	0	0	22[b]	77[b]
8	$Pd_2(dba)_3$	PBn_3	0	0	0	95
9	$Pd_2(dba)_3$	PPh_2Et	10	2	0	87
10	$Pd_2(dba)_3$	$PPhMe_2$	0	2	0	73
11	$Pd_2(dba)_3$	JohnPhos	0	0	6	60
12	$Pd_2(dba)_3$	*t*BuXPhos	0	0	37	18
13	$Pd_2(dba)_3$	*t*BuDavePhos	0	0	10	58
14	$Pd_2(dba)_3$	QPhos	0	0	18	62
15	$Pd_2(dba)_3$	D*t*BPF	0	0	0	92
16	$Pd_2(dba)_3$	APhos	0	0	0	100
17	$Pd_2(dba)_3$	BippyPhos	0	0	84	0
18	$Pd_2(dba)_3$	cataCXium® PtB	0	0	23	28
19	$Pd_2(dba)_3$	XPhos	3	19	0	77
20	$Pd_2(dba)_3$	$P(NMe_2)_3$	0	0	0	100
21	$Pd_2(dba)_3$	$P(NEt_2)_3$	0	0	0	98
22	$Pd_2(dba)_3$	PTA	0	23	0	68
23	$Pd_2(dba)_3$	dppe	0	0	0	97
24	$Pd_2(dba)_3$	dppp	0	0	0	97
25	$Pd_2(dba)_3$	dppb	0	0	0	96
26	$Pd_2(dba)_3$	dppbenz	0	0	0	98
27	$Pd_2(dba)_3$	DPEphos	4	0	0	95
28	$Pd_2(dba)_3$	Xantphos	4	12	0	82
29	$Pd_2(dba)_3$	BINAP	0	6	0	85
30	$Pd_2(dba)_3$	phen	0	1	0	94
31	$Pd_2(dba)_3$	SIPr	0	5	37	48
32	$Pd_2(dba)_3$	IPr	0	4	28	42

[a] GC yields. [b] NMR yields.

structure of ligands

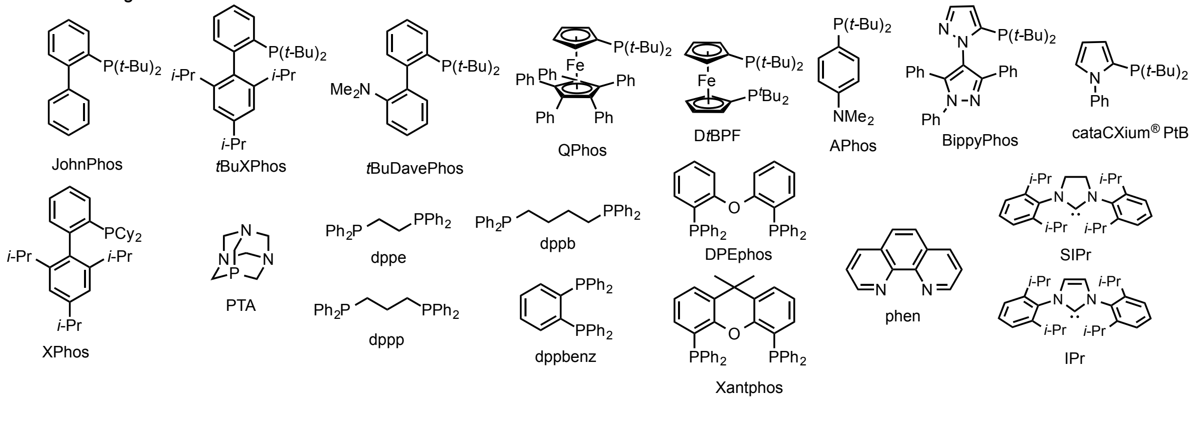

2. Effect of Solvent

A typical procedure for the screening of solvent. In a glove box, to a 3 mL vial with a magnetic stir (10 mm) bar were added $Pd_2(dba)_3$ (1.0 mg, 1.0 μmol, 0.5 mol%), P(*t*-Bu)$_2$Me (0.05 M solution of the solvent used, 80 μL, 4.0 μmol, 2 mol%), and solvent (530 μL), and the resulting solution was stirred at 60 °C for 5 min on an aluminum heating block. After allowing the vial cool to room temperature, *N*-tosyl-2-phenylaziridine (54.6 mg, 0.20 mmol) and $B_2(pin)_2$ (60.9 mg, 0.24 mmol, 1.2 equiv) were added. The vial was capped with a hole cap and a Teflon®/rubber septum and removed from the glove box. Deionized H_2O (50 μL) was added through the septum under a stream of N_2 gas, and the resulting mixture was stirred at 60 °C on an aluminum heating block for 3 h. *n*-Dodecane (34.1 mg, 0.20 mmol, 1.0 equiv, as an internal standard for GC analysis) was added to the vial through the septum, and the mixture was diluted with acetone and subjected to GC analysis. Yields of products were estimated based on a calibration curve method.

Table S2. Effect of Solvent

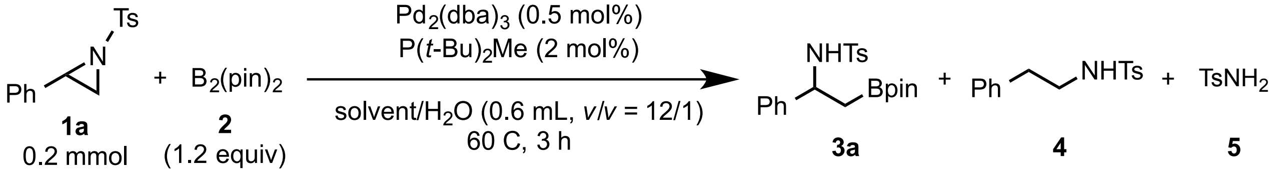

entry	solvent	yield (%)^a			recovery of
		**3a**	**4**	**5**	**1a** (%)^a
1	Et_2O	29	4	35	31
2	THF	19	4	27	41
3	1,4-dioxane	28	5	45	2
4	MeCN	8	2	0	89
5	DMF	21	12	42	16
6	CH_2Cl_2	5	2	4	80
7	toluene	10	3	3	72
8	MTBE	69	0	8	0

^{a} GC yields

3. Effect of Pd source

A typical procedure for the screening of Pd source. In a glove box, to a 3 mL vial with a magnetic stir (10 mm) bar were added Pd source (2.0 μmol, 1 mol%), P(*t*-Bu)$_2$Me (0.05 M solution of the MTBE, 80 μL, 4.0 μmol, 2 mol%), and MTBE (530 μL), and the resulting solution was stirred at 60 °C for 5 min on an aluminum heating block. After allowing the vial cool to room temperature, *N*-tosyl-2-phenylaziridine (54.6 mg, 0.20 mmol) and $B_2(pin)_2$ (60.9 mg, 0.24 mmol, 1.2 equiv) were added. The vial was capped with a hole cap and a Teflon®/rubber septum and removed from the glove box. Deionized H_2O (50 μL) was added through the septum under a stream of N_2 gas, and the resulting mixture was stirred at 60 °C on an aluminum heating block for 3 h. The reaction mixture was diluted with CH_2Cl_2 (3 mL), and the organic layer was washed with brine (3 mL). The aqueous layer was extracted with CH_2Cl_2 (5 mL × 3), and the combined organic layer was dried over Na_2SO_4. Yields of products were estimated based on ^{1}H NMR integration using 1,1,2,2,-tetrachloroethane as an internal standard.

Table S3. Effect of Pd source

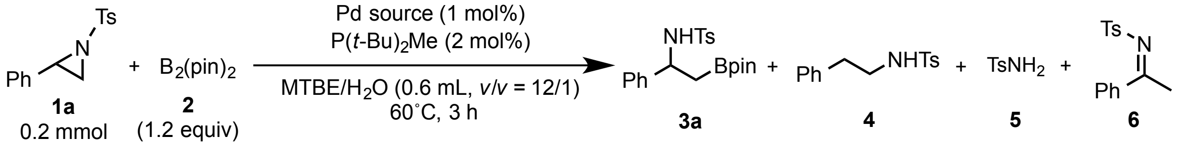

entry	Pd source	yield (%)[a]				recovery of 1a (%)[a]
		3a	4	5	6	
1	Cp(allyl)Pd	52	0	14	25	0
2	Cp(cinnamyl)Pd	47	0	12	25	0
3	$Pd_2(dba)_3$	69	26	8	0	0

[a] ^{1}H NMR yield.

structure of Pd source

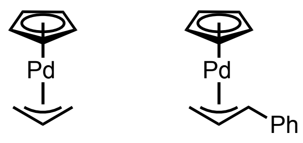

Cp(allyl)Pd   Cp(cinnamyl)Pd

4. Effect of Additive

A typical procedure for the screening of additive. In a glove box, to a 3 mL vial with a magnetic stir (10 mm) bar were added Cp(allyl)Pd (0.1 M solution of MTBE, 20 μL, 2.0 μmol, 1 mol%), P(*t*-Bu)$_2$Me (0.05 M solution of MTBE, 80 μL, 4.0 μmol, 2 mol%), and MTBE (510 μL), and the resulting solution was stirred at 60 °C for 5 min on an aluminum heating block. After allowing the vial cool to room temperature, *N*-tosyl-2-phenylaziridine (54.6 mg, 0.20 mmol), B$_2$(pin)$_2$ (60.9 mg, 0.24 mmol, 1.2 equiv), and additive (2–20 mol%) were added. The vial was capped with a hole cap and a Teflon®/rubber septum and removed from the glove box. Deionized H_2O (50 μL) was added through the septum under a stream of N_2 gas, and the resulting mixture was stirred at 60 °C on an aluminum heating block for 3 h. The reaction mixture was diluted with CH_2Cl_2 (3 mL), and the organic layer was washed with brine (3 mL). The aqueous layer was extracted with CH_2Cl_2 (5 mL × 3), and the combined organic layer was dried over Na_2SO_4. Yields of products were estimated based on ^{1}H NMR integration using 1,1,2,2,-tetrachloroethane as an internal standard.

Table S4. Effect of additive 1 (Benzoquinone Derivatives)

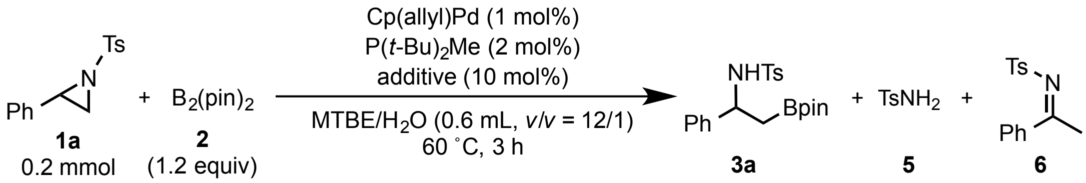

entry	additive	yield (%)[a]			recovery of 1a (%)[a]
		3a	5	6	
1	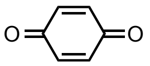	65	18	15	0
2	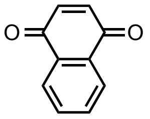	63	10	19	0
3	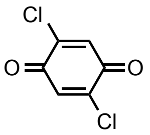	0	4	0	95
4	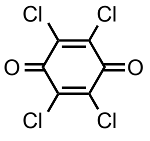	0	0	0	91
5	none	52	14	25	0

[a] ^{1}H NMR yield.

Table S5. Effect of additive 2 (Styrene Derivatives)

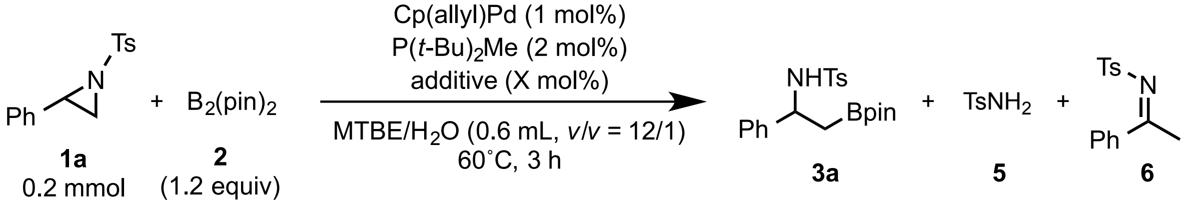

entry	additive (X mol%)		yield (%)[a] 3a	5	6	recovery of 1a (%)[a]
1	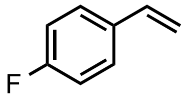	(5)	46	7	12	0
2		(10)	69	21	12	0
3		(20)	48	7	15	0
4	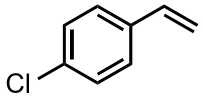	(5)	53	7	12	0
5		(10)	73	20	11	0
6		(20)	66	9	19	0
7	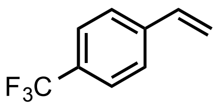	(5)	64	8	16	0
8		(10)	69	20	8	0
9		(20)	55	7	12	0
10	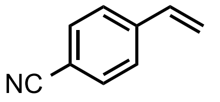	(10)	60	9	17	0
11	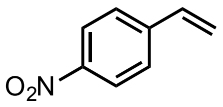	(10)	61	9	16	0
12	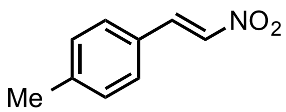	(5)	65	11	14	0
13	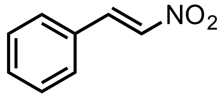	(5)	55	14	16	0
14	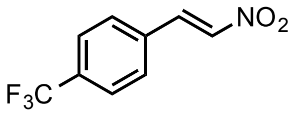	(5)	38	8	18	34
15	none		52	14	25	0

[a] ^1H NMR yield.

Table S6. Effect of additive 3 (other types of alkenes and alkynes)

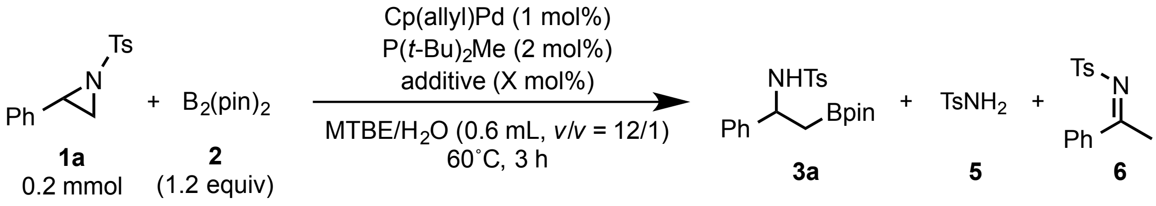

entry	additive (X mol%)		yield (%)[a] 3a	5	6	recovery of 1a (%)[a]
1	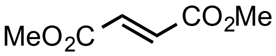	(10)	0	10	0	90
2	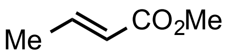	(10)	0	trace	0	95
3	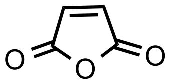	(10)	trace	3	0	96
4	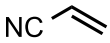	(5)	52	7	16	14
5	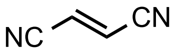	(5)	0	0	3	83
6	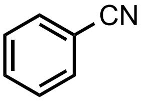	(5)	52	9	17	0
7	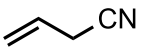	(5)	64	9	15	0
8	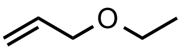	(5)	64	8	17	0
9	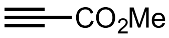	(5)	trace	0	0	92
10	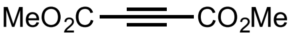	(5)	0	0	0	88
11	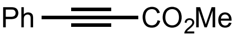	(5)	51	8	15	8
12	none		52	14	25	0

[a] ^{1}H NMR yield.

Table S7. Effect of additive 4 (bipyridine derivatives)

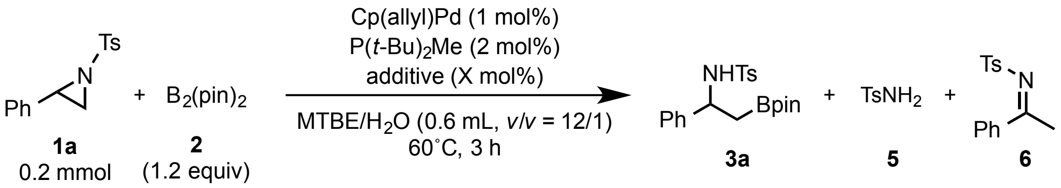

entry	additive (X mol%)		yield (%)[a] 3a	5	6	recovery of 1a (%)[a]
1	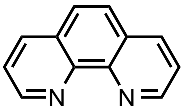	(5)	trace	trace	0	84
2	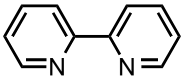	(2)	70	12	17	0
3		(5)	74	11	12	3
4		(10)	64	15	19	0
5[b]		(5)	41	trace	8	37
6[c]		(5)	50	15	23	0
7	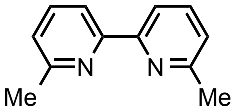	(5)	61	9	19	0
8	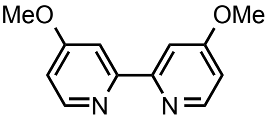	(5)	57	8	18	0
9	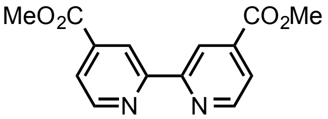	(5)	52	10	25	0
10	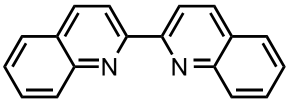	(5)	55	13	16	0
11	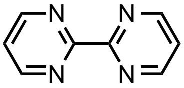	(5)	67	13	22	0
12	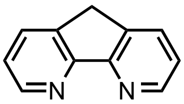	(5)	65	11	19	0
13	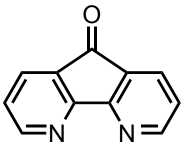	(5)	63	9	15	0
14	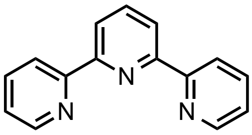	(5)	63	10	19	0
15	none		52	14	25	0

[a] ^{1}H NMR yields. [b] 50 ˚C. [c] Cp(allyl)Pd, ^{t}Bu$_2$MeP and bipyridine were pre-stired at 50 ˚C for 5 min.

Table S8. Effect of additive 5 (pyridine derivatives)

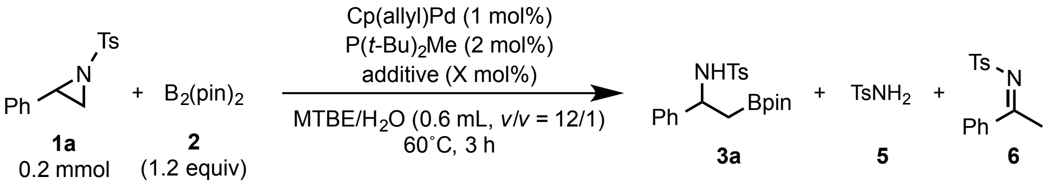

entry	additive (X mol%)		yield (%)[a] 3a	5	6	recovery of 1a (%)[a]
1	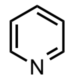	(5)	61	10	21	0
2	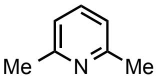	(5)	62	10	18	0
3	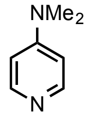	(5)	66	13	20	0
4	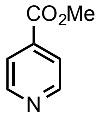	(5)	0	55	0	21
5	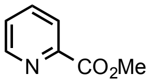	(5)	52	10	23	0
6	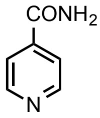	(5)	46	28	0	0
7	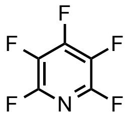	(5)	39	5	30	0
8	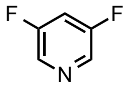	(5)	55	8	15	0
9	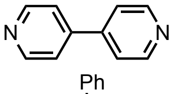	(5)	33	62	0	0
10	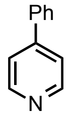	(5)	57	18	16	0
11	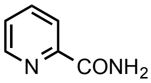	(5)	51	12	21	0
12	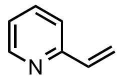	(5)	58	9	15	0
13	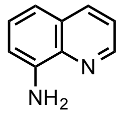	(5)	42	9	14	0
14	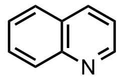	(5)	53	14	16	0
15	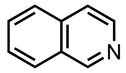	(5)	46	21	18	0
16	none		52	14	25	0

[a] ^{1}H NMR yield.

5. Effect of the Pd/P(t-Bu)$_2$Me/bpy ratio

A typical procedure for the screening of the catalyst ratio. In a glove box, to a 3 mL vial with a magnetic stir (10 mm) bar were added Cp(allyl)Pd (0.1 M solution of MTBE, 1 or 2 mol%), P(t-Bu)$_2$Me (0.05 M solution of MTBE, 0.1–3 mol%), and MTBE (1.0 mL), and the resulting solution was stirred at 60 °C for 5 min on an aluminum heating block. After allowing the vial cool to room temperature, *N*-tosyl-2-phenylaziridine (136.7 mg, 0.50 mmol), B_2(pin)$_2$ (152.7 mg, 0.60 mmol, 1.2 equiv), and bipyridine (2–20 mol%) were added. The vial was capped with a hole cap and a Teflon®/rubber septum and removed from the glove box. Deionized H_2O (125 μL) was added through the septum under a stream of N_2 gas, and the resulting mixture was stirred at 60 °C on an aluminum heating block for 3 h. The reaction mixture was diluted with CH_2Cl_2 (10 mL), and the organic layer was washed with brine (5 mL). The aqueous layer was extracted with CH_2Cl_2 (10 mL × 3), and the combined organic layer was dried over Na_2SO_4. Yields of products were estimated based on ^{1}H NMR integration using 1,1,2,2,-tetrachloroethane as an internal standard.

Table S9. Effect of catalyst ratio

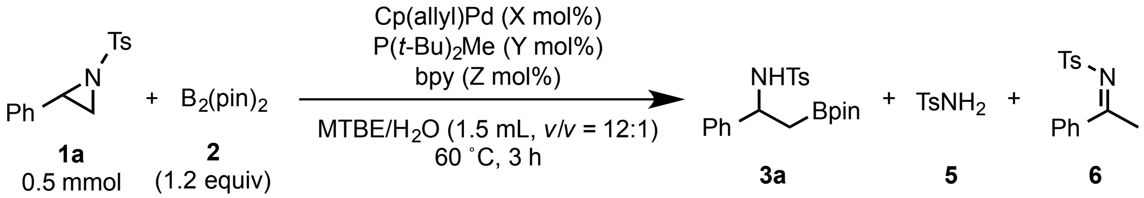

entry	Cp(allyl)Pd (X mol%)	P(t-Bu)$_2$Me (Y mol%)	*P*/*Pd*	bpy (Z mol%)	yield (%)[a] 3a	5	6	recovery of **1a** (%)[a]
1	1	2	2	5	66	12	14	0
2	1	3	3	5	45	10	14	0
3	1	1	1	5	72	11	13	0
4	1	0.5	0.5	5	75	9	10	0
5	1	0.1	0.1	5	4	0	2	78
6	1	0.5	0.5	2	58	10	10	0
7	1	0.5	0.5	20	77	4	10	0
8	2	1	0.5	20	81	0	13	0

[a] ^{1}H NMR yields.

6. Effect of reaction temperature

A typical procedure for the screening of reaction temperature. In a glove box, to a 3 mL vial with a magnetic stir (10 mm) bar were added Cp(allyl)Pd (0.1 M solution of MTBE, 100 μL, 10 μmol, 2 mol%), P(t-Bu)$_2$Me (0.05 M solution of MTBE, 100 μL, 5 μmol, 1 mol%), and MTBE (1.3 mL), and the resulting solution was stirred at 60 °C for 5 min on an aluminum heating block. After allowing the vial cool to room temperature, *N*-tosyl-2-phenylaziridine (136.7 mg, 0.50 mmol), B_2(pin)$_2$ (152.7 mg, 0.60 mmol, 1.2 equiv), and bipyridine (16.0 mg, 0.1 mmol, 20 mol%) were added. The vial was capped with a hole cap and a Teflon®/rubber septum and removed from the glove box. Deionized H_2O (125 μL) was added through the septum under a stream of N_2 gas, and the resulting mixture was stirred at 60 °C on an aluminum heating block for 3 h. The reaction mixture was diluted with CH_2Cl_2 (10 mL), and the organic layer was washed with brine (5 mL). The aqueous layer was extracted with CH_2Cl_2 (10 mL × 3), and the combined organic layer was dried over Na_2SO_4. Yields of products were estimated based on ^{1}H NMR integration using 1,1,2,2,-tetrachloroethane as an internal standard.

Table S10. Effect of reaction temperature

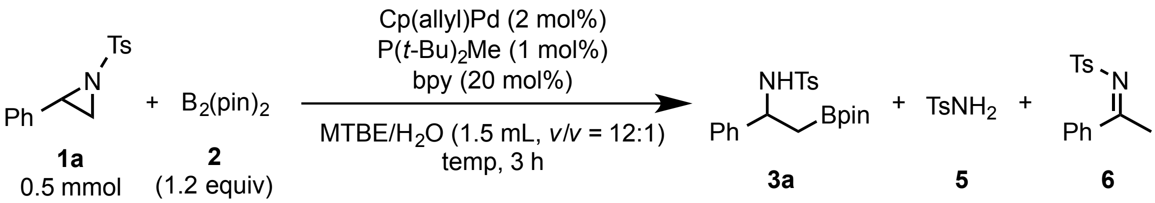

entry	temp. (˚C)	yield (%)[a]			recovery of
		3a	5	6	1a (%)[a]
1[b]	rt	11	0	2	86
2	40	16	0	2	64
3	50	33	5	7	34
4	60	81	0	13	0

[a] ^{1}H NMR yields. [b] 6 h.

7. Effect of equivalent of water

A typical procedure for the screening of the equivalent of water. In a glove box, to a 3 mL vial with a magnetic stir (10 mm) bar were added Cp(allyl)Pd (0.1 M solution of MTBE, 50 μL, 5 μmol, 1 mol%), P(*t*-Bu)$_2$Me (0.05 M solution of MTBE, 50 μL, 2.5 μmol, 0.5 mol%), and MTBE (1.3 mL), and the resulting solution was stirred at 60 °C for 5 min on an aluminum heating block. After allowing the vial cool to room temperature, *N*-tosyl-2-phenylaziridine (136.7 mg, 0.50 mmol), B$_2$(pin)$_2$ (152.7 mg, 0.60 mmol, 1.2 equiv), and bipyridine (16.0 mg, 0.1 mmol, 20 mol%) were added. The vial was capped with a hole cap and a Teflon®/rubber septum and removed from the glove box. Deionized H_2O (0–20 equiv) was added through the septum under a stream of N_2 gas, and the resulting mixture was stirred at 60 °C on an aluminum heating block for 3 h. The reaction mixture was diluted with CH_2Cl_2 (10 mL), and the organic layer was washed with brine (5 mL). The aqueous layer was extracted with CH_2Cl_2 (10 mL × 3), anTad the combined organic layer was dried over Na_2SO_4. Yields of products were estimated based on ^{1}H NMR integration using 1,1,2,2,-tetrachloroethane as an internal standard.

Table S11. Effect of equivalent of water

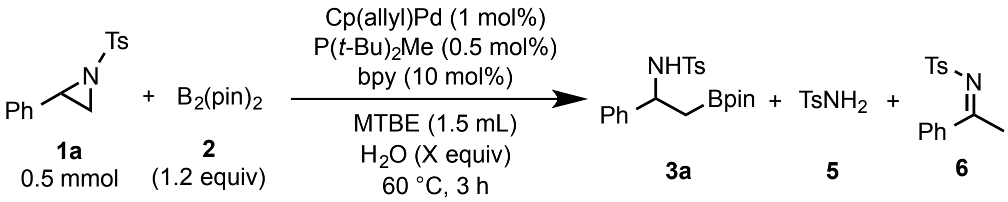

entry	H_2O (X equiv)	yield (%)[a]			recovery of
		3a	5	6	1a (%)[a]
1	20	79	4	11	0
2	14	81	5	10	0
3	10	78	3	9	0
4	5	79	4	10	0
5	1	52	0	9	33
6	0	0	0	7	84

[a] ^{1}H NMR yields.

8. Effect of N-sulfonyl group

A typical procedure for the screening of N-functionalized 2-phenylaziridines 1r–1u. In a glove box, to a 3 mL vial with a magnetic stir (10 mm) bar were added Cp(allyl)Pd (0.1 M solution of MTBE, 100 μL, 10 μmol, 2 mol%), P(*t*-Bu)$_2$Me (0.05 M solution of MTBE, 100 μL, 5.0 μmol, 1 mol%), and MTBE (0.3 mL), and the resulting solution was stirred at 50 °C for 5 min on an aluminum heating block. After allowing the vial cool to room temperature, MTBE (1.0 mL), *N*-sulfonyl-2-phenylaziridine **1** (0.50 mmol), B_2(pin)$_2$ (152.7 mg, 0.60 mmol, 1.2 equiv), and bipyridine (16.0 mg, 0.1 mmol, 20 mol%) were added. The vial was capped with a hole cap and a Teflon®/rubber septum and removed from the glove box. Deionized H_2O (125 μL) was added through the septum under a stream of N_2 gas, and the resulting mixture was stirred at 60 °C on an aluminum heating block for 3 h. The reaction mixture was diluted with CH_2Cl_2 (10 mL), and the organic layer was washed with brine (5 mL). The aqueous layer was extracted with CH_2Cl_2 (10 mL × 3), and the combined organic layer was dried over Na_2SO_4. Solvent was removed under reduced pressure to give crude products, which product was then purified by flash column chromatography on silica gel followed by gel permeation chromatography ($CHCl_3$). ^{1}H Yields of products were estimated based on ^{1}H NMR integration using 1,1,2,2,-tetrachloroethane as an internal standard.

Table S12. Effect of N-functional group

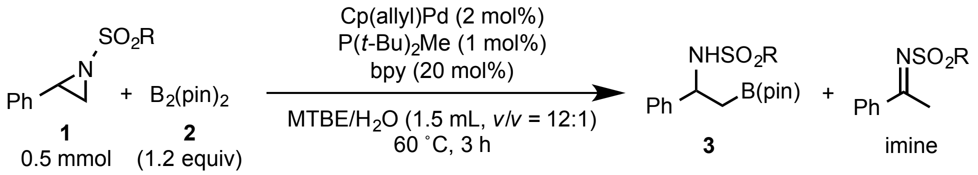

entry	R (**1**)		yield (%)[a] **3**	yield (%)[a] imine	recovery of **1** (%)[a]
1	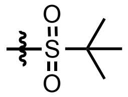	(**1r**)	**3r** 68 (52)[b]	12 (2)[b]	0
2	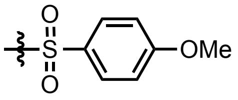	(**1s**)	**3s** 75 (65)[b]	12	0
3	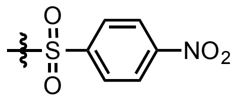	(**1t**)	0	0	75
4	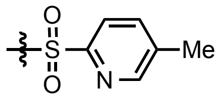	(**1u**)	<22[c]	0	64

[a] ^{1}H NMR yields. [b] isolated yield. [c] Mixtures with some impurities, which were difficult to separate.

9. Effect of boron source

A typical procedure for the screening of boron source. In a glove box, to a 3 mL vial with a magnetic stir (10 mm) bar were added Cp(allyl)Pd (0.1 M solution of MTBE, 100 μL, 10 μmol, 2 mol%), P(*t*-Bu)$_2$Me (0.05 M solution of MTBE, 100 μL, 5.0 μmol, 1 mol%), and MTBE (0.3 mL), and the resulting solution was stirred at 50 °C for 5 min on an aluminum heating block. After allowing the vial cool to room temperature, MTBE (1.0 mL), *N*-tosyl-2-phenylaziridine **1a** (136.7 mg, 0.50 mmol), boron source (0.60 mmol, 1.2 equiv), and bipyridine (16.0 mg, 0.1 mmol, 20 mol%) were added. The vial was capped with a hole cap and a Teflon®/rubber septum and removed from the glove box. Deionized H_2O (125 μL) was added through the septum under a stream of N_2 gas, and the resulting mixture was stirred at 60 °C on an aluminum heating block for 3 h. The reaction mixture was diluted with CH_2Cl_2 (10 mL), and the organic layer was washed with

brine (5 mL). The aqueous layer was extracted with CH_2Cl_2 (10 mL × 3), and the combined organic layer was dried over Na_2SO_4. Solvent was removed under reduced pressure to give crude products, which product was then purified by gel permeation chromatography ($CHCl_3$). ^{1}H Yields of products were estimated based on ^{1}H NMR integration using 1,1,2,2,-tetrachloroethane as an internal standard.

Table S13. Effect of boron source

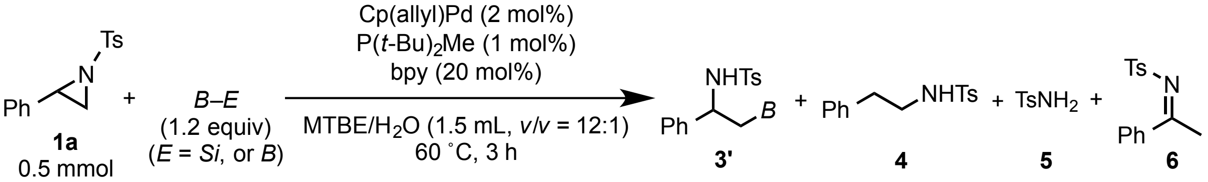

entry	B–E	yield (%)[a] 3'	4	5	6	recovery of **1a** (%)[a]
1	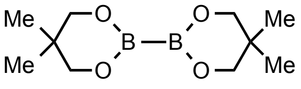	**3'a** 45 (39)[b]	29	0	10	0
2	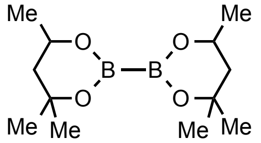	**3'b** 12 (10)[b, c]	7	0	5	50
3	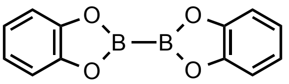	0	70[b]	0	2	18
4	$(HO)_2B$—$B(OH)_2$	0	79[b]	0	0	8
5[d]	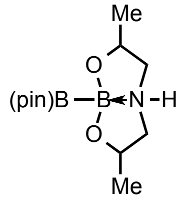	0	0	10	7	77
6	(pin)B—$SiMe_2Ph$	0	0	0	0	100

[a] ^{1}H NMR yields. [b] isolated yield. [c] *d.r.* = 1:1. [d] Boron reagent was prepared according to literature (ref. S5).

A general procedure for the Pd-catalyzed borylative ring-opening of 2-arylaziridines. In a glove box, to a 3 mL vial with a magnetic stir (10 mm) bar were added Cp(allyl)Pd (0.1 M solution of MTBE, 100 μL, 10 μmol, 2 mol%), P(*t*-Bu)$_2$Me (0.05 M solution of MTBE, 100 μL, 5 μmol, 1 mol%), and MTBE (1.3 mL), and the resulting solution was stirred at 60 °C for 5 min on an aluminum heating block. After allowing the vial cool to room temperature, aziridine **1** (0.50 mmol), $B_2(pin)_2$ (152.7 mg, 0.60 mmol, 1.2 equiv), and bipyridine (16.0 mg, 10 μmol, 20 mol%) were added. The vial was capped with a hole cap and a Teflon®/rubber septum and removed from the glove box. Deionized H_2O (125 μL) was added through the septum under a stream of N_2 gas, and the resulting mixture was stirred at 60 °C on an aluminum heating block for 3 h. The reaction mixture was diluted with CH_2Cl_2 (10 mL), and the organic layer was washed with brine (5 mL). The aqueous layer was extracted with CH_2Cl_2 (10 mL × 3), and the combined organic layer was dried over Na_2SO_4. The NMR yields of products were estimated based on ^{1}H NMR integration using 1,1,2,2,-tetrachloroethane as an internal standard. The crude product was purified by flash column chromatography on silica gel to give borylated products. Since the complete separation of borylated products **3** from the remaining $B_2(pin)_2$ was very difficult by silica gel chromatography in

most cases due to their similar polarities, further purification of products was conducted by gel permutation chromatography (GPC, eluent: $CHCl_3$).

***A procedure for gram-scale borylation of 1a**.* In a glove box, to a 100 mL test tube with a magnetic stir bar were added Cp(allyl)Pd (0.1 M solution of MTBE, 2.0 mL, 0.2 mmol, 2 mol%), P(*t*-Bu)$_2$Me (0.05 M solution of MTBE, 2.0 mL, 0.1 mmol, 1 mol%), and MTBE (3.0 mL). The tube was removed from glove box, and the solution was stirred at 50 °C for 5 min. After allowing the vial cool to room temperature, MTBE (23 mL), bipyridine (320 mg, 2.0 mmol, 20 mol%), B_2(pin)$_2$ (3.05 g, 12 mmol, 1.2 equiv), *N*-tosyl-2phenyaziridine (**1a**) (2.73 g, 10 mmol), and deionized H_2O (2.5 mL) were added under a stream of N_2 gas. The resulting mixture was stirred at 60 °C for 3 h. To the reaction mixture, brine (25 mL) was added, and the organic layer was extracted with CH_2Cl_2 (25 mL × 3), and the combined organic layer was dried over Na_2SO_4. Solvent was removed under reduced pressure to give the crude product, which was purified by flash column chromatography on silica gel followed by gel permeation chromatography ($CHCl_3$), giving borylated product **3a** in 72% (2.89 g).

Spectroscopic data of Borylated Products

4-Methyl-*N*-(1-phenyl-2-(4,4,5,5-tetramethyl-1,3,2-dioxaborolan-2-yl)ethyl)-benzensulfon-amide (3a)

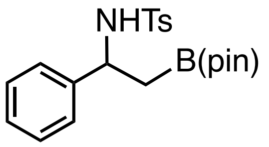

Purified by silica gel column chromatography (hexane/EtOAc 99:1 to 7:3) followed by GPC (eluent: $CHCl_3$); 71% yield (^{1}H NMR yield: 81%); Colorless solid; Mp 112.7 °C (dec.); R_f 0.31 (hexane/EtOAc 7:3); ^{1}H NMR (400 MHz, $CDCl_3$) δ 7.60 (2H, d, J = 8.4 Hz), 7.18–7.12 (7H, m), 5.47 (1H, d, J = 8.4 Hz), 4.66–4.61 (1H, m), 2.37 (3H, s), 1.35 (1H, dd, J = 15.6, 5.6 Hz), 1.23 (1H, dd, J = 15.6, 7.0 Hz), 1.11 (6H, s), 1.10 (6H, s); ^{13}C NMR (100 MHz, $CDCl_3$) δ 142.8, 142.2, 137.9, 129.2, 128.1, 127.0, 126.9, 126.2, 83.6, 54.2, 24.6, 24.5, 21.4, 20.3 (br); IR (ATR, cm^{-1}): ν 3256, 2984, 1458, 1444, 1413, 1361, 1338, 1319, 1155, 1095, 1049, 850, 808 704; MS (CI, isobutane) *m/z* (relative intensity, %): 402 ([M+H]$^+$, 3), 298 (25), 231 ([M–NHTs]$^+$, 100); HRMS (CI, isobutane): calcd for $C_{21}H_{29}NO_4SB$ (M+H) 402.1910, found 402.1911.

4-Methyl-*N*-(2-(4,4,5,5-tetramethyl-1,3,2-dioxaborolan-2-yl)-1-(*p*-tolyl)ethyl)benzenesulfon-amide (3b)

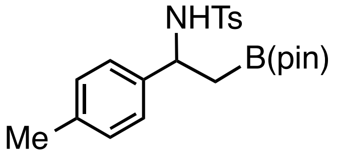

Purified by silica gel column chromatography (hexane/EtOAc 99:1 to 7:3) followed by GPC ($CHCl_3$); 71% yield (^{1}H NMR yield: 83%); Colorless solid; Mp 78.3 °C (dec.); R_f 0.38 (hexane/EtOAc 3:1); ^{1}H NMR (400 MHz, $CDCl_3$): δ 7.60 (2H, d, J = 8.4 Hz), 7.15 (2H, d, J = 8.0 Hz), 7.02 (2H, d, J = 8.4 Hz), 6.95 (2H, d, J = 8.0 Hz), 5.47 (1H, d, J = 8.0 Hz), 4.60–4.55 (1H, m), 2.37 (3H, s), 2.25 (3H, s), 1.33 (1H, dd, J = 15.8, 5.2 Hz), 1.20 (1H, dd, J = 15.8, 6.8 Hz), 1.11 (6H, s), 1.10 (6H, s); ^{13}C NMR (100 MHz, $CDCl_3$): δ 142.7, 139.2, 137.9, 136.5, 129.2, 128.7, 127.0, 126.1, 83.5, 54.0, 24.6, 24.5, 21.4, 20.9, 20.1 (br); IR (ATR, cm^{-1}): ν 3265, 2973, 1370, 1327, 1317, 1303, 1299, 1143, 1094, 1038, 924, 844, 809; MS (FAB$^-$) *m/z* (relative intensity, %): 414 ([M–H]$^+$, 100), 296 (17); HRMS (FAB$^-$): calcd for $C_{22}H_{29}NO_4SB$ ([M–H]$^+$) 414.1910, found 414.1907.

4-Methyl-*N*-(2-(4,4,5,5-tetramethyl-1,3,2-dioxaborolan-2-yl)-1-(*m*-tolyl)ethyl)benzenesulfon-amide (3c)

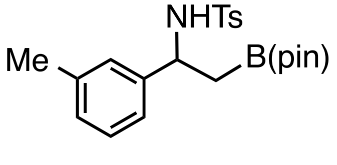

Purified by silica gel column chromatography (hexane/EtOAc 99:1 to 7:3) followed by GPC ($CHCl_3$); 71% yield (^{1}H NMR yield: 77%); Colorless solid; Mp 78.5 °C (dec.); R_f 0.40 (hexane/EtOAc 7:3); ^{1}H NMR (400 MHz, $CDCl_3$): δ 7.59 (2H, d, J = 8.4 Hz), 7.13 (2H, d, J = 8.4 Hz), 7.04 (1H, t, J = 8.0 Hz), 6.94–6.90 (2H, m), 6.87 (1H, s), 5.53 (1H, d, J = 8.4 Hz), 4.62–4.57 (1H, m), 2.35 (3H, s), 2.18 (3H, s), 1.33 (1H, dd, J = 16.0, 5.6 Hz),

1.21 (1H, dd, J = 16.0, 7.2 Hz), 1.11 (6H, s), 1.10 (6H, s); ^{13}C NMR (100 MHz, $CDCl_3$) δ 142.6, 142.0, 137.9, 137.4, 129.1, 127.9, 127.6, 126.97, 126.93, 123.3, 83.5, 54.3, 24.6, 24.5, 21.3, 21.1, 20.2 (br); IR (ATR, cm^{-1}): ν 3256, 2980, 1363, 1326, 1303, 1142, 1093, 1033, 945, 844, 814, 731; MS (FAB$^-$) m/z (relative intensity, %): 414 ([M–H]$^+$, 78), 296 (11); HRMS (FAB$^-$): calcd for $C_{22}H_{29}NO_4SB$ ([M–H]$^+$) 414.1910, found 414.1916.

4-Methyl-*N*-(2-(4,4,5,5-tetramethyl-1,3,2-dioxaborolan-2-yl)-1-(*o*-tolyl)ethyl)benzenesulfonamide (3d)

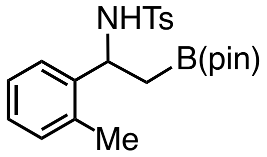

Purified by silica gel column chromatography (hexane/EtOAc, 99:1 to 7:3) followed by GPC ($CHCl_3$); 81% yield (^{1}H NMR yield: 86%); Colorless solid; Mp 82.5 °C (dec.); R_f 0.38 (hexane/EtOAc 7:3); ^{1}H NMR (400 MHz, $CDCl_3$): δ 7.54 (2H, d, J = 8.4 Hz), 7.10–7.07 (3H, m), 6.98–6.89 (3H, m), 5.58 (1H, d, J = 7.6 Hz), 4.88–4.83 (1H, m), 2.32 (3H, s), 2.24 (3H, s), 1.30 (2H, d, J = 7.2 Hz), 1.08 (6H, s), 1.06 (6H, s); ^{13}C NMR (100 MHz, $CDCl_3$): δ 142.7, 140.3, 137.8, 134.6, 130.1, 129.1, 126.88, 126.87, 125.9, 125.5, 83.5, 50.6, 24.6, 24.5, 21.3, 19.8 (br), 19.0; IR (ATR, cm^{-1}): ν 3271, 2976, 1381, 1369, 1328, 1313, 1153, 1143, 1092, 1049, 846, 820, 751; MS (FAB$^-$) m/z (relative intensity, %): 414 ([M–H]$^+$, 94), 296 (13); HRMS (FAB$^-$): calcd for $C_{22}H_{29}NO_4SB$ ([M–H]$^+$) 414.1910, found 414.1911.

***N*-(1-(4-Fluorophenyl)-2-(4,4,5,5-tetramethyl-1,3,2-dioxaborolan-2-yl)ethyl)-4-methylbenzenesulfonamide (3e)**

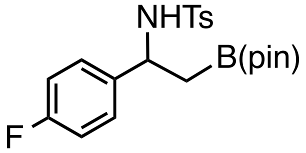

Purified by silica gel column chromatography (hexane/EtOAc, 99:1 to 7:3) followed by GPC ($CHCl_3$); 75% yield (^{1}H NMR yield: 88%); Colorless solid; Mp 106.0 °C (dec.); R_f 0.35 (hexane/EtOAc 7:3); ^{1}H NMR (400 MHz, $CDCl_3$): δ 7.58 (2H, d, J = 8.4 Hz), 7.16–7.09 (4H, m), 6.82 (2H, dd, J_{HH} = 8.4 Hz, J_{HF} = 8.4 Hz), 5.62 (1H, brd, J = 7.2 Hz), 4.63–4.58 (1H, m), 2.36 (3H, s), 1.32 (1H, dd, J = 15.8, 5.6 Hz), 1.21 (1H, dd, J = 15.8, 8.0 Hz), 1.098 (6H, s), 1.093 (6H, s); ^{13}C NMR (100 MHz, $CDCl_3$): δ 161.7 (d, $J_{C–F}$ = 243.6 Hz), 142.9, 138.0, 137.7, 129.2, 128.0 (d, $J_{C–F}$ = 8.3 Hz), 126.9, 114.7 (d, $J_{C–F}$ = 21.4 Hz), 83.6, 53.7, 24.5, 21.37, 21.32, 20.3 (br); IR (ATR, cm^{-1}): ν 3255, 2871, 1603, 1508, 1371, 1327, 1291, 1222, 1155, 1141, 1093, 1056, 966, 890, 846, 808; MS (FAB$^-$) m/z (relative intensity, %): 418 ([M–H]$^+$, 100), 296 (13); HRMS (FAB$^-$): calcd for $C_{21}H_{26}FNO_4SB$ ([M–H]$^+$) 418.1660, found 418.1659.

***N*-(1-(4-Chlorophenyl)-2-(4,4,5,5-tetramethyl-1,3,2-dioxaborolan-2-yl)ethyl)-4-methylbenzenesulfonamide (3f)**

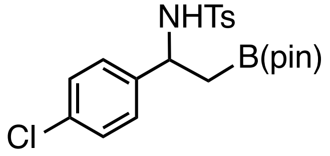

Purified by silica gel column chromatography (hexane/EtOAc 99:1 to 7:3) followed by GPC ($CHCl_3$); 81% yield (^{1}H NMR yield: 90%); Colorless solid; Mp 129.4 °C (dec.); R_f 0.35 (hexane/EtOAc 3:1); ^{1}H NMR (400 MHz, $CDCl_3$): δ 7.57 (2H, d, J = 8.4 Hz), 7.17–7.03 (6H, m), 5.49 (1H, brd, J = 7.6 Hz), 4.61–4.56 (1H, m), 2.38 (3H, s), 1.31 (1H, dd, J = 15.8, 5.6 Hz), 1.20 (1H, dd, J = 15.8, 6.8 Hz), 1.12 (6H, s), 1.11 (6H, s); ^{13}C NMR (100 MHz, $CDCl_3$): δ 143.1, 140.8, 137.7, 132.7, 129.3, 128.1, 127.7, 127.0, 83.8, 53.7, 24.6, 24.5, 21.4, 20.0 (br); IR (ATR, cm^{-1}): ν 3259, 2975, 1370, 1325, 1299, 1142, 1092, 1085, 1044, 1010, 969, 889, 845, 808, 723, 705; MS (FAB$^-$) m/z (relative intensity, %): 436 ([M+2–H]$^+$, 23), 434 ([M–H]$^+$, 86), 296 (13); HRMS (FAB$^-$): calcd for $C_{21}H_{26}ClNO_4SB$ ([M–H]$^+$) 434.1364, found 434.1366.

4-Methyl-*N*-(2-(4,4,5,5-tetramethyl-1,3,2-dioxaborolan-2-yl)-1-(4-(trifluoromethyl)phenyl)-ethyl)benzenesulfonamide (3g)

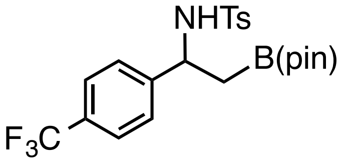

Purified by silica gel column chromatography (hexane/EtOAc 99:1 to 7:3) followed by GPC ($CHCl_3$); 74% yield (^{1}H NMR yield: 85%); Colorless solid; Mp 164.3 °C (dec.); R_f 0.35 (hexane/EtOAc 3:1); ^{1}H NMR (400 MHz, $CDCl_3$): δ 7.53 (2H, d, J = 8.4 Hz), 7.35 (2H, d, J =

8.0 Hz), 7.23 (2H, d, *J* = 8.4 Hz), 7.08 (2H, d, *J* = 8.0 Hz), 5.81 (1H, d, *J* = 8.0 Hz), 4.70–4.64 (1H, m), 2.33 (3H, s), 1.35 (1H, dd, *J* = 16.0, 6.0 Hz), 1.26 (1H, dd, *J* = 16.0, 7.2 Hz), 1.10 (12H, s); ^{13}C NMR (100 MHz, $CDCl_3$): δ 146.2, 143.0, 137.5, 129.2, 129.1 (q, $J_{C\text{–}F}$ = 31.3 Hz), 126.9, 126.7, 124.9 (q, $J_{C\text{–}F}$ = 3.3 Hz), 124.0 (q, $J_{C\text{–}F}$ = 270.9 Hz), 83.8, 54.1, 24.59 24.52, 21.2, 20.3 (br); IR (ATR, cm^{-1}): ν 3273, 2899, 1372, 1325, 1143, 1122, 1066, 1047, 967, 890, 846, 810, 704; MS (FAB^-) *m*/*z* (relative intensity, %): 468 ($[M\text{–}H]^+$, 100), 296 (13); HRMS (FAB^-): calcd for $C_{22}H_{26}F_3NO_4SB$ ($[M\text{–}H]^+$) 468.1628, found 468.1624.

4-Methyl-*N*-(1-(4-nitrophenyl)-2-(4,4,5,5-tetramethyl-1,3,2-dioxaborolan-2-yl)ethyl)benzene-sulfonamide (3h)

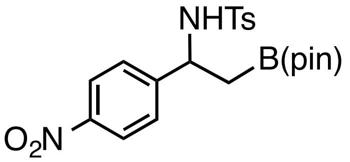

Purified by silica gel column chromatography (hexane/EtOAc, 99:1 to 7:3) followed by GPC ($CHCl_3$); 81% yield (^{1}H NMR yield: 87%); Yellow solid; Mp 175.4 °C (dec.); R_f 0.25 (hexane/EtOAc 7:3); ^{1}H NMR (400 MHz, $CDCl_3$): δ 8.03 (2H, d, *J* = 8.8 Hz), 7.59 (2H, d, *J* = 8.4 Hz), 7.35 (2H, d, *J* = 8.8 Hz), 7.17 (2H, d, *J* = 8.4 Hz), 5.65 (1H, d, *J* = 8.0 Hz), 4.71–4.66 (1H, m), 2.37 (3H, s), 1.32 (1H, dd, *J* = 16.0, 5.6 Hz), 1.24 (1H, dd, *J* = 16.0, 6.8 Hz), 1.13 (6H, s), 1.12 (6H, s); ^{13}C NMR (100 MHz, $CDCl_3$): δ 149.9, 146.8, 143.5, 137.4, 129.4, 127.2, 127.0, 123.3, 84.0, 53.8, 24.6, 24.5, 21.4, 20.0 (br); IR (ATR, cm^{-1}): ν 3295, 2979, 1599, 1518, 1343, 1326, 1151, 1143, 1089, 1008, 963, 859, 846, 808; MS (FAB^-) *m*/*z* (relative intensity, %): 445 ($[M\text{–}H]^+$, 100), 296 (11); HRMS (FAB^-): calcd for $C_{21}H_{26}N_2O_6SB$ ($[M\text{–}H]^+$) 445.1605, found 445.1600.

4-(1-((4-Methylphenyl)sulfonamido)-2-(4,4,5,5-tetramethyl-1,3,2-dioxaborolan-2-yl)ethyl)-phenyl acetate (3i)

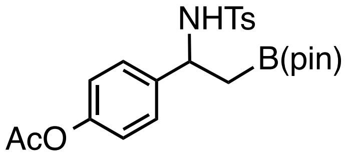

Purified by silica gel column chromatography (hexane/EtOAc 99:1 to 7:3) followed by GPC ($CHCl_3$); 58% yield (^{1}H NMR yield: 71%); Colorless solid; Mp 149.8 °C (dec.); R_f 0.20 (hexane/EtOAc 7:3); ^{1}H NMR (400 MHz, $CDCl_3$): δ 7.59 (2H, d, *J* = 8.4 Hz), 7.18–7.14 (4H, m), 6.88 (2H, d, *J* = 8.4 Hz), 5.47 (1H, d, *J* = 8.0 Hz), 4.66–4.61 (1H, m), 2.37 (3H, s), 2.26 (3H, s), 1.34 (1H, dd, *J* = 15.8, 5.6 Hz), 1.22 (1H, dd, *J* = 15.8, 6.8 Hz), 1.11 (6H, s), 1.10 (6H, s); ^{13}C NMR (100 MHz, $CDCl_3$): δ 169.2, 149.5, 143.0, 139.8, 137.7, 129.3, 127.3, 126.9, 121.1, 83.7, 53.7, 24.6, 24.5, 21.4, 21.0, 20.3 (br); IR (ATR, cm^{-1}): ν 3265, 2991, 1751, 1505, 1365, 1337, 1320, 1291, 1215, 1199, 1155, 1143, 1047, 1015, 946, 912, 888, 846, 816, 706; MS (FAB^-) *m*/*z* (relative intensity, %): 458 ($[M\text{–}H]^+$, 66), 153 (100), 296 (12); HRMS (FAB^-): calcd for $C_{23}H_{29}NO_6SB$ ($[M\text{–}H]^+$) 458.1809, found 458.1806.

Methyl-4-(1-((4-methylphenyl)sulfonamido)-2-(4,4,5,5-tetramethyl-1,3,2-dioxaborolan-2-yl)-ethyl)benzoate (3j)

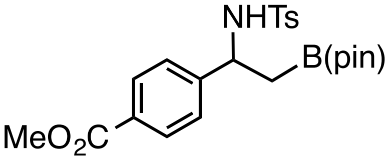

Purified by silica gel column chromatography (hexane/EtOAc 99:1 to 7:3) followed by GPC ($CHCl_3$); 77% yield (^{1}H NMR yield: 83%); Colorless solid; Mp 100.2 °C (dec.); R_f 0.28 (hexane/EtOAc 7:3); ^{1}H NMR (400 MHz, $CDCl_3$): δ 7.82 (2H, d, *J* = 8.4 Hz), 7.59 (2H, d, *J* = 8.4 Hz), 7.22 (2H, d, *J* = 8.4 Hz), 7.13 (2H, d, *J* = 8.4 Hz), 5.72 (1H, d, *J* = 8.0 Hz), 4.69–4.64 (1H, m), 3.89 (3H, s), 2.34 (3H, s), 1.34 (1H, dd, *J* = 16.2, 5.6 Hz), 1.24 (1H, dd, *J* = 16.2, 7.2 Hz), 1.10 (6H, s), 1.09 (6H, s); ^{13}C NMR (100 MHz, $CDCl_3$): δ 166.8, 147.5, 143.1, 137.7, 129.5, 129.4, 128.8, 127.0, 126.3, 83.8, 54.0, 52.0, 24.7, 24.5, 21.4, 20.2 (br); IR (ATR, cm^{-1}): ν 3283, 2980, 1717, 1371, 1326, 1274, 1145, 1102, 1053, 968, 891, 846, 810, 771, 712; MS (FAB^-) *m*/*z* (relative intensity, %): 458 ($[M\text{–}H]^+$, 67), 296 (6); HRMS (FAB^-): calcd for $C_{23}H_{29}NO_6SB$ ($[M+H]^-$) 458.1809, found 458.1812.

4-Methoxy-*N*-(1-phenyl-2-(4,4,5,5-tetramethyl-1,3,2-dioxaborolan-2-yl)ethyl)benzenesulfonamide (3r)

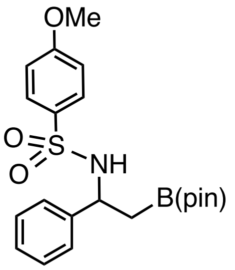

Purified by silica gel column chromatography (hexane/EtOAc 99:1 to 7:3) followed by GPC ($CHCl_3$); 65% yield (^{1}H NMR yield: 75%); Colorless solid; Mp 101.5 °C (dec.); R_f 0.05 (hexane/EtOAc 8:2); ^{1}H NMR (400 MHz, $CDCl_3$): δ 7.63 (2H, d, J = 9.6 Hz), 7.19–7.09 (5H, m), 6.81 (2H, d, J = 9.6 Hz), 5.48 (1H, d, J = 8.4 Hz), 4.64–4.59 (1H, m), 3.81 (3H, s), 1.36 (1H, dd, J = 16.2, 5.6 Hz), 1.24 (1H, dd, J = 16.2, 7.2 Hz), 1.109 (6H, s), 1.102 (6H, s); ^{13}C NMR (100 MHz, $CDCl_3$): δ 162.4, 142.1, 132.6, 129.1, 128.1, 127.0, 126.2, 113.8, 83.6, 55.5, 54.3, 24.6, 24.5, 20.3 (br); IR (ATR, cm^{-1}): ν 3253, 2985, 1597, 1506, 1448, 1363, 1348, 1328, 1298, 1257, 1153, 1095, 1045, 1029, 852, 831, 705; MS (CI, isobutane) m/z (relative intensity, %): 418 ($[M+H]^+$, 1), 314 (17), 231 ($[M–p\text{-}MeOC_6H_4SO_2NH]^+$, 100); HRMS (CI, isobutane): calcd for $C_{21}H_{29}NO_5SB$ ($[M+H]^+$) 418.1859, found 418.1859.

2-Methyl-*N*-(1-phenyl-2-(4,4,5,5-tetramethyl-1,3,2-dioxaborolan-2-yl)ethyl)propane-2-sulfonamide (3s)

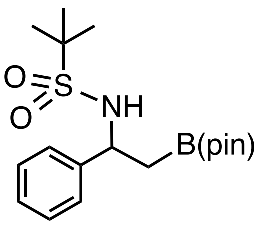

Purified by silica gel column chromatography (hexane/EtOAc 99:1 to 7:3) followed by GPC ($CHCl_3$); 52% yield (^{1}H NMR yield: 68%); Colorless solid; Mp 111.3 °C (dec.); R_f 0.38 (hexane/EtOAc 7:3); ^{1}H NMR (400 MHz, $CDCl_3$): δ 7.34–7.28 (4H, m), 7.21 (1H, t, J = 6.8 Hz), 5.12 (1H, d, J = 9.2 Hz), 4.82-4.77 (1H, m), 1.60–1.50 (2H, m), 1.31 (9H, s), 1.12 (12H, s); ^{13}C NMR (100 MHz, $CDCl_3$): δ 143.7, 128.3, 127.0, 126.0, 83.5, 59.5, 55.1, 24.6, 24.5, 24.1, 22.2 (br); IR (ATR, cm^{-1}): ν 3267, 2980, 1456, 1372, 1326, 1298, 1145, 1125, 1051, 967, 876, 845, 759, 703; MS (FAB^-) m/z (relative intensity, %): 366 ($[M–H]^+$, 70), 262 (7); HRMS (FAB^-): calcd for $C_{18}H_{29}NO_4SB$ ($[M–H]^+$) 366.1910, found 366.1914.

***N*-(2-(5,5-Dimethyl-1,3,2-dioxaborinan-2-yl)-1-phenylethyl)-4-methylbenzenesulfonamide (3'a)**

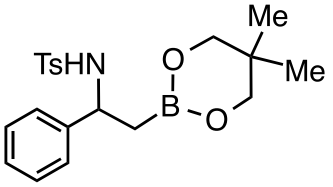

Purified by gel permeation chromatography ($CHCl_3$); 39% yield (^{1}H NMR yield: 45%); Colorless solid; Mp 105.5 °C (dec.); ^{1}H NMR (400 MHz, $CDCl_3$): δ 7.59 (2H, d, J = 8.4 Hz), 7.18–7.07 (7H, m), 5.59 (1H, d, J = 7.2 Hz), 4.61–4.56 (1H, m), 3.48 (4H, s), 2.36 (3H, s), 1.26 (1H, dd, J = 5.6, 15.8 Hz), 1.18 (1H, dd, J = 6.4, 15.8 Hz), 0.78 (6H, s); ^{13}C NMR (100 MHz, $CDCl_3$): δ 142.8, 142.6, 137.9, 129.2, 128.1, 127.0, 126.7, 126.2, 71.8, 54.6, 31.4, 23.7 (br), 21.5, 21.3; IR (ATR, cm^{-1}): ν 3288, 2960, 1477, 1417, 1323, 1294, 1257, 1157, 1076, 1041, 1006, 935, 813, 746, 700; MS (FAB^-) m/z (relative intensity, %): 386 ($[M–H]^+$, 49), 282 (9); HRMS (FAB^-): calcd for $C_{20}H_{25}NO_4SB$ ($[M–H]^+$) 386.1597, found 386.1594.

4-Methyl-*N*-(1-phenyl-2-(4,4,6-trimethyl-1,3,2-dioxaborinan-2-yl)ethyl)benzenesulfonamide (3'b)

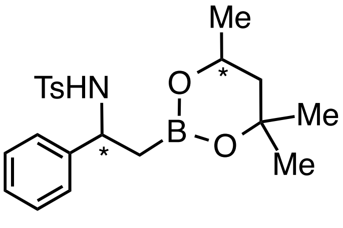

Purified by gel permeation chromatography ($CHCl_3$); 10% yield (obtained as an inseparable 1:1 mixture of diastereomers, ^{1}H NMR yield: 12%); Colorless solid; Mp 108.2 °C (dec.); ^{1}H NMR (400 MHz, $CDCl_3$): δ 7.56 (4H, d, J = 8.4 Hz), 7.14–7.12 (14H, m), 5.64 (1H, d, J = 7.2 Hz), 5.60 (1H, d, J = 7.6 Hz), 4.60–4.54 (2H, m), 4.11–4.03 (2H, m), 2.36 (6H, s), 1.71–1.66 (2H, m), 1.34–1.28 (2H, m), 1.20–1.14 (18H, multiple singlets); ^{13}C NMR (100 MHz, $CDCl_3$): δ 142.9, 142.6, 138.0, 129.2, 127.9, 127.0, 126.6, 126.3, 71.3, 65.0, 54.9, 54.8, 45.5, 30.9, 27.8, 27.7, 24.0 (br), 22.9, 21.4; IR (ATR, cm^{-1}): ν 3297, 2952, 1474, 1427, 1317, 1286, 1228, 1137, 1081, 993, 828,

711; MS (FAB$^-$) *m/z* (relative intensity, %): 400 ([M–H]$^+$, 65), 296 (10); HRMS (FAB$^-$): calcd for $C_{21}H_{27}NO_4SB$ ([M–H]$^+$) 400.1754, found 400.1756.

Derivatization of Aminoboronates:

1. Preparation of β-Aminotrifluoroborate

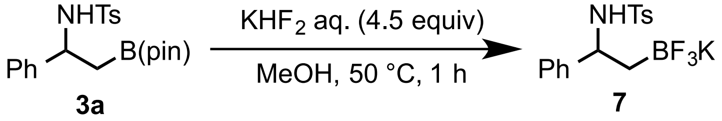

Procedure for the transformation of 3a to 4-Methyl-N-(1-phenyl-2-(trifluoro-λ^4-boranyl)-ethyl)benzenesulfonamide potassium salt (7)

According to the procedure described in literature with some modifications;[S6] To a 20 mL Schlenk tube equipped with a magnetic stir bar, was added **3a** (401.3 mg, 1.0 mmol) under air. The tube was evacuated and refilled with N_2 gas three times. MeOH (5.0 mL) was added, and KHF_2 4.5 M aqueous solution prepared from 351. 5 mg of KHF_2 (4.5 mmol) and 1.0 mL of H_2O was subsequently dropwise added to the solution. The resulting mixture was stirred at 50 °C for 1 h. The solvent was then removed under reduced pressure, and the solid residue was triturated with acetone (5 mL). The solution was filtrated and washed with additional acetone (2 mL × 2). The combined solution was concentrated under reduced pressure to give colorless solid, which was washed with diethyl ether (5 mL × 3) and dried under vacuum to give the title product as an 0.5 hydrate (see the elementary analysis) in 94% yield; Colorless solid; Mp 207.4 °C (dec.); ^{1}H NMR (400 MHz, DMSO-d_6): δ 7.50 (2H, d, J = 8.4 Hz), 7.26 (2H, d, J = 8.4 Hz), 7.13–7.03 (5H, m), 6.90 (1H, brs), 4.11–4.06 (1H, m), 2.37 (3H, s), 0.53–0.38 (2H, m); ^{13}C NMR (100 MHz, DMSO): δ 146.6, 141.7, 138.1, 129.0, 127.2, 126.6, 126.5, 125.3, 57.2, 30.0 (br), 20.9; IR (ATR, cm^{-1}): ν 3249, 2920, 1411, 1330, 1317, 1286, 1159, 1085, 1068, 1053, 1018, 941, 910, 898, 825, 806, 765; Anal. Calcd for $C_{15}H_{16}BF_3KNO_2S\cdot 0.5H_2O$: C, 46.16; H, 4.39; N, 3.59. Found: C, 46.50; H, 4.30; N, 3.60.

2. Synthesis of Enantiopure Tetrahydroisoquinoline Derivative

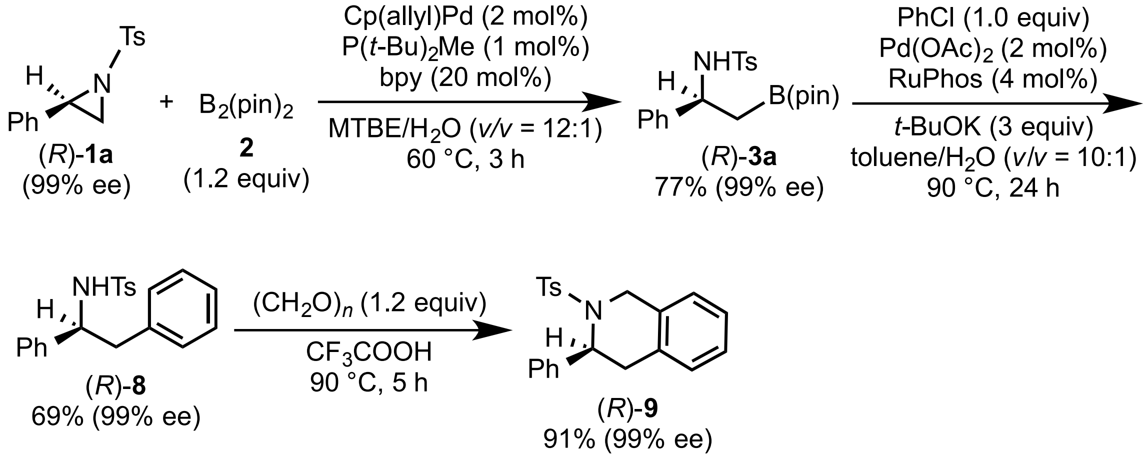

Scheme S1. Synthetic route to tetrahydroisoquinoline **9**

***Step 1: preparation of enantiopure β*-amino alkylboronate[(*S*)-3a]**

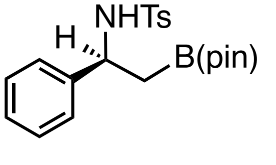

Enantiopure boronate (*S*)-**3a** was prepared according to the general procedure for Pd-catalyzed ring-opening borylation using 1.37 g of enantiopure (*R*)-2-phenyl-1-tosylaziridine [(*R*)-**1a**] as the substrate; 71% yield; Spectroscopic data were in agreement with racemic product **3a**; HPLC (Chiralcel OJ, 1.0 mL/min; *i*-PrOH/*n*-hexane 20:80, λ = 254 nm): t_R 5.6 min;

$[\alpha]_D^{20}$ –35.0 (*c* 1.05, $CHCl_3$). The absolute chemistry of this compound was deduced from that of (*S*)-**8** (shown below).

***Step 2: Pd-catalyzed Suzuki-Miyaura cross-coupling of (S)-3a with chlorobenzene leading to (S)-N-(1,2-diphenylethyl)-4-methylbenzenesulfonamide* [(*S*)-8]**

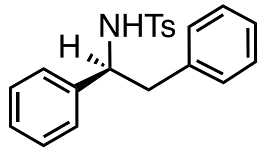

The Suzuki-Miyaura cross-coupling of (*S*)-**3a** with PhCl was conducted according to the procedure described in literature with some modifications;[S6] To a 10 mL Schlenk tube equipped with a magnetic stir bar (20 mm), were added $Pd(OAc)_2$ (4.5 mg, 20 μmol, 2 mol%), RuPhos (19.0 mg, 40 μmol, 4 mol%), and *t*-BuOK (336.6 mg, 3.0 mmol, 3 equiv) under air. The tube was evacuated and refilled with N_2 gas three times. Toluene (3.0 mL) was added to the tube, and the resulting mixture was stirred at room temperature for 10 min. Chlorobenzene (112.0 mg, 1.0 mmol, 1 equiv), (*S*)-**3a** (401.3 mg, 1.0 mmol, 1 equiv), and deionized H_2O (0.3 mL) were added, and the resulting solution was stirred vigorously at 90 °C for 24 h. The reaction mixture was filtered through a pad of Celite and washed with CH_2Cl_2 (25 mL). The filtrate was concentrated in vacuo, and the resulting residue was purified by flash column chromatography on silica gel (hexane/EtOAc 99:1 to 7:3) to give product (*S*)-**8**, which was then subjected to chiral HPLC analysis; 69% yield; 99% ee (HPLC); Colorless solid; Mp 126.5 °C dec.; R_f 0.45 (hexane/EtOAc = 7/3); Spectroscopic data were in agreement with those of previously reported racemic **8**;[S7] ^{1}H NMR (400 MHz, $CDCl_3$): δ 7.43 (2H, d, *J* = 8.0 Hz), 7.14–7.12 (6H, m), 7.05–7.02 (4H, m), 6.91–6.89 (2H, m), 5.39 (1H, brs), 4.51–4.46 (1H, m), 2.96 (2H, d, *J* = 7.2 Hz), 2.33 (3H, s); ^{13}C NMR (100 MHz, $CDCl_3$): δ 142.8, 140.3, 137.0, 136.4, 129.24, 129.21, 128.3, 128.1, 127.2, 126.9, 126.6, 59.2, 44.0, 21.3 (one sp^2 carbon corresponding to a Ph group seems to be overlapped with some peaks); IR (ATR, cm^{-1}): ν 3360, 3062, 1494, 1454, 1141, 1313, 1304, 1286, 1152, 1087, 1060, 951, 921, 808, 746, 704; MS (CI, isobutane) *m/z* (relative intensity, %): 352 ($[M+H]^+$, 5), 181 ($[M–NHTs]^+$, 100); HRMS (CI, isobutane): calcd for $C_{21}H_{22}NO_2S$ ($[M+H]^+$) 352.1371, found 352.1367; HPLC (Chiralcel OJ, 1.0 mL/min; *i*-PrOH/*n*-hexane 20:80; λ = 254 nm): t_R 10.6 min; $[\alpha]_D^{20}$ –34.8 (*c* 1.05, $CHCl_3$). Racemic sample of **8** [CAS No. 93172-47-5] for chiral HPLC analysis was synthesized by the same procedure using racemic **3**. The absolute stereochemistry of this compound was confirmed by comparing the specific optical rotations of this compound and the authentic sample prepared thorough the N-tosylation of enantiopure (*S*)-1,2-diphenylethan-1-amine.[S8]

***Step 3: Pictet-Spengler cyclization of (S)-8 leading to (S)-3-phenyl-2-tosyl-1,2,3,4-tetrahydroisoquinoline* [(*S*)-9]**

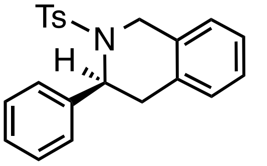

The title compound was synthesized from (*S*)-**8** according to the procedure described in literature with some modifications;[S9] To a 10 mL Schlenk tube equipped with a magnetic stir bar, were added (*S*)-**8** (175.5 mg, 0.50 mmol) and paraformaldehyde (18.0 mg, 0.6 mmol based on formaldehyde, 1.2 equiv) under the air. The tube was evacuated and refilled with N_2 gas three times. Trifluoroacetic acid (1.0 mL) was added, and the resulting mixture was stirred at 90 °C for 5 h. The reaction mixture was allowed cool to room temperature and poured into H_2O (10 mL). The organic layer was separated, and the aqueous layer was extracted with $CHCl_3$ (3 mL × 3). Organic layers were combined, washed with sat. $NaHCO_3$ aq. (3 mL), and dried over Na_2SO_4. Solvent was removed under reduced pressure to give crude product, which was purified by flash column chromatography on silica gel (hexane/EtOAc 99:1 to 9:1), giving the title compound (*S*)-**9** as colorless solid in 91% yield. The product (*S*)-**9** was then subjected to chiral HPLC analysis; 99% ee (HPLC); Spectroscopic data were in agreement with those of previously reported racemic **9**;[S10] ^{1}H NMR (400 MHz, $CDCl_3$): δ 7.62 (2H, d, *J* = 8.0 Hz), 7.21–6.97 (11H, m), 5.38 (1H, t, *J* = 4.4 Hz), 4.69 (1H, d, *J* = 16.4 Hz), 4.15 (1H, d, *J* = 16.4 Hz), 3.05 (2H, d, *J* = 4.4 Hz), 2.35 (3H, s); ^{13}C NMR (100 MHz, $CDCl_3$): δ 143.1, 139.7, 137.0, 132.5, 132.3, 129.4, 128.5, 128.3, 127.3, 127.1, 127.06, 127.05, 126.3, 125.8, 54.5, 43.9, 32.1, 21.4; IR (ATR, cm^{-1}): ν 3066, 3028, 2864, 1788, 1598, 1496, 1456, 1350, 1332, 1161, 1089, 1060, 941, 891, 812, 769; HRMS (CI, isobutane): calcd

for $C_{22}H_{22}NO_2S$ ([M+H]$^+$) 364.1371, found 364.1367; HPLC (Chiralcel OD-H, 1.0 mL/min; *i*-PrOH/*n*-hexane 20:80; λ = 254 nm): t_R 18.4 min. Racemic sample of **9** [CAS No. 1182624-36-7] for chiral HPLC analysis was synthesized by the same procedure using racemic **8**. The absolute stereochemistry of this compound was deduced from that of (*S*)-**8**.

Deuterium-Labeling Experiments. In order to determine the stetreochemical information of the borylation, borylated product obtained from deuterated aziridine (relative stereochemistry is *cis*) *cis*-**1a**-***d*₁**[S3c] was derivatized according to the same route and procedures as those in Scheme S1 (Scheme S2).

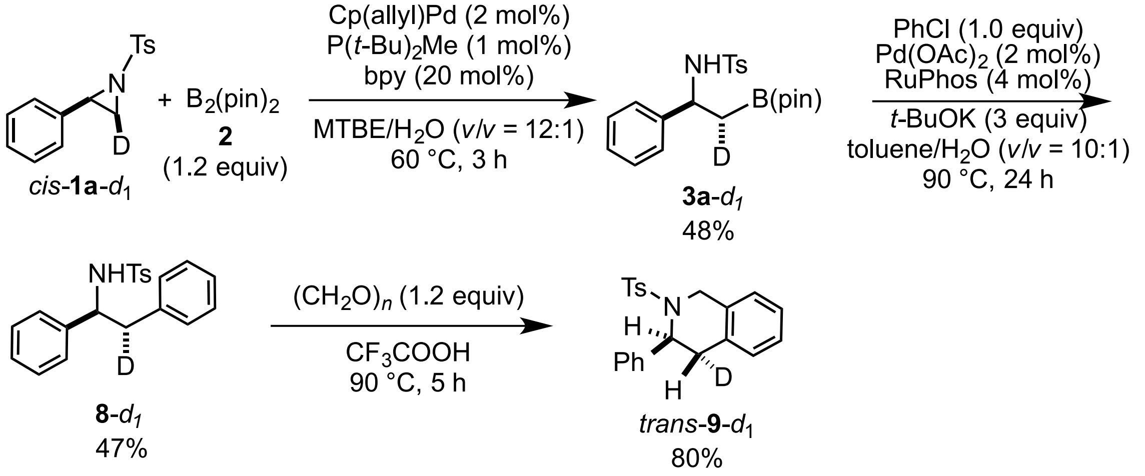

Scheme S2. Derivatization of deutrated aziridine *cis*-**1a-d₁** to tetrahydroisoquinoline *trans*-**9-d₁**

Step 1: Borylation of cis-1a-d₁ leading to 3a-d₁

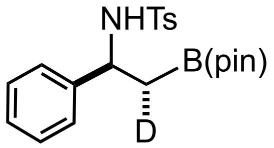

Purified by silica gel column chromatography (hexane/EtOAc 99:1 to 7:3) followed by GPC ($CHCl_3$); 56% yield; Colorless solid; Mp 113.5 °C (dec.); R_f 0.31 (hexane/EtOAc 7:3); ^{1}H NMR (400 MHz, $CDCl_3$): δ 7.60 (2H, d, J = 8.0 Hz), 7.17–7.11 (7H, m), 5.47 (1H, d, J = 7.6 Hz), 4.65–4.61 (1H, m), 2.36 (3H, s), 1.33 (1H, d, J = 5.2 Hz), 1.10 (6H, s), 1.09 (6H, s); ^{13}C NMR (100 MHz, $CDCl_3$): δ 142.8, 142.2, 137.9, 129.3, 128.1, 127.0 (2C), 126.2, 83.6, 54.2, 24.64, 24.56, 21.4 (the signal corresponding to the carbon directly connected to the boron atom was not detected due to the quadrupolar relaxation); IR (ATR, cm^{-1}): ν 3261, 2978, 2927, 2173, 1598, 1442, 1361, 1330, 1319, 1322, 1155, 1139, 1095, 1045, 972, 935, 852, 808, 758, 702; MS (FAB$^-$) *m*/*z* (relative intensity, %): 401 ([M–H]$^+$, 100), 297 (3); HRMS (FAB$^-$): calcd for $C_{21}H_{26}DBNO_4S$ [(M–H)]$^+$ 401.1817, found 401.1816.

***Step 2: Cross-coupling of 3a-d₁ leading to* 8-*d*₁**

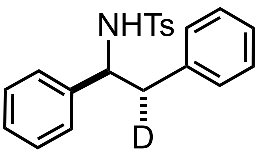

Purified by silica gel column chromatography (hexane/EtOAc 99:1 to 7:3); 47% yield; Colorless solid; Mp 126.3 °C (dec.); R_f 0.45 (hexane/EtOAc 7:3); ^{1}H NMR (400 MHz, $CDCl_3$): δ 7.42 (2H, d, J = 8.4 Hz), 7.18–7.17 (6H, m), 7.10–7.03 (4H, m), 6.91–6.89 (2H, m), 4.75 (1H, d, J = 6.0 Hz), 4.50 (1H, dd, J = 7.2, 6.0 Hz), 2.96 (1H, d, J = 7.2 Hz), 2.37 (3H, s); ^{13}C NMR (100 MHz, $CDCl_3$): δ 143.0, 140.3, 136.9, 136.1, 129.3, 129.2, 128.5, 128.3, 127.4, 127.0, 126.8, 126.6, 58.9, 43.7 (t, J_{CD} = 19.4 Hz), 21.4; IR (ATR, cm^{-1}): ν 3275, 3062, 3028, 2924, 2183, 1598, 1494, 1454, 1321, 1303, 1290, 1091, 1058, 925, 812, 754; MS (CI, isobutane) *m*/*z* (relative intensity, %): 353 ([M+H]$^+$, 3), 248 (100), 182 ([M–NHTs]$^+$, 27); HRMS (CI) calcd for $C_{21}H_{21}DNO_2S$ ([M+H]$^+$) 353.1434, found 353.1431.

Step 3: Pictet-Spengler Cyclization of 8-d_1 leading to trans-9-d_1

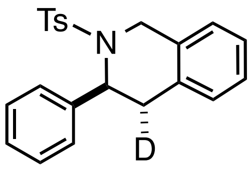

Purified by silica gel column chromatography (hexane/EtOAc 99:1 to 7:3); 80% yield; Colorless solid; Mp 126.3 °C dec.; R_f 0.68 (hexane/EtOAc = 7/3); ^{1}H NMR (400 MHz, $CDCl_3$): δ 7.72 (2H, d, J = 8.4 Hz), 7.23–6.97 (11H, m), 5.38 (1H, d, J = 7.6 Hz), 4.69 (1H, d, J = 16.8 Hz), 4.18 (1H, d, J = 16.8 Hz), 3.04 (1H, d, J = 7.6 Hz), 2.36 (s, 3H); ^{13}C NMR (100 MHz, $CDCl_3$) δ 143.1, 139.8, 137.2, 132.5, 132.4, 129.4, 128.5, 128.3, 127.3, 127.1, 127.0, 126.9, 126.3, 125.8, 54.6, 44.0 (m), 32.0, 21.4; IR (ATR, cm^{-1}): 3062, 3028, 2922, 2852, 2169, 1598, 1494, 1452, 1344, 1114, 1089, 1051, 956, 923, 813, 756, 738; MS (CI$^+$, isobutane) *m*/*z* (relative intensity, %): 365 ([M+H]$^+$, 50), 287 ([M–Ph]$^+$, 12); HRMS (CI$^+$) calcd for $C_{22}H_{21}DNO_2S$ ([M+H]$^+$) 365.1434, found 365.1429. The relative configuration was determined by comparison with known compounds shown below.[S11]

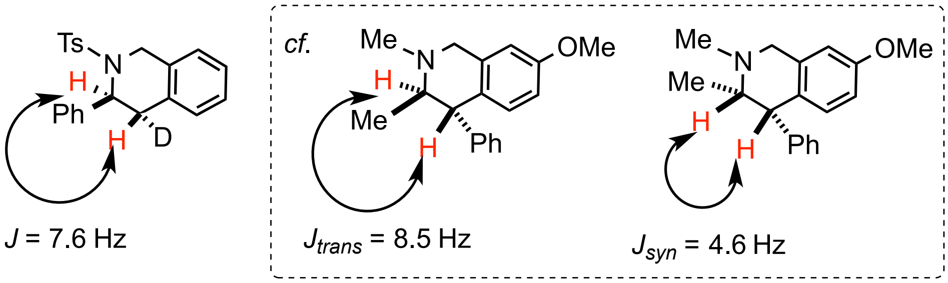

NMR Experiments for monitoring the reaction of Cp(ally)Pd with P(*t*-Bu)$_2$Me.

Experiment 1: In a glove box, to a NMR sample tube fitted with a J. Young Teflon valve were added Cp(allyl)Pd (8.4 mg, 40 μmol), P(*t*-Bu)$_2$Me (0, 20, 40, or 80 μmol), and THF-d_8 (0.5 mL). The NMR tube was removed from glove box and stirred at 60 ˚C for 1 h. $^{31}P\{^1H\}$ NMR spectra were acquired at 60 ˚C (Figure S1a).

Experiment 2: To investigate the interaction between Pd species generated in situ and bpy, 10 equiv of byp (62.4 mg, 0.4 mmol) was added to the mixture of Cp(allyl)Pd (8.4 mg, 40 μmol) and P(*t*-Bu)$_2$Me (6.4 mg, 40 μmol) after stirring at 60 ˚C for 1 h in THF-d_8 (0.5 mL). $^{31}P\{^1H\}$ NMR spectra were acquired at 60 ˚C (Figure S1b).

a)

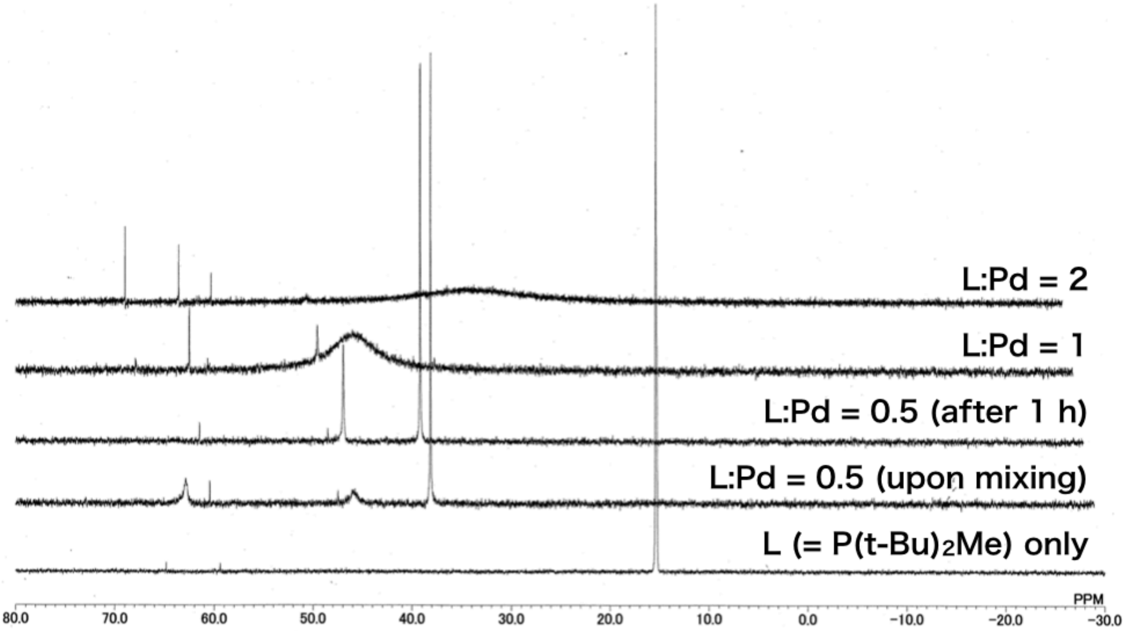

b)

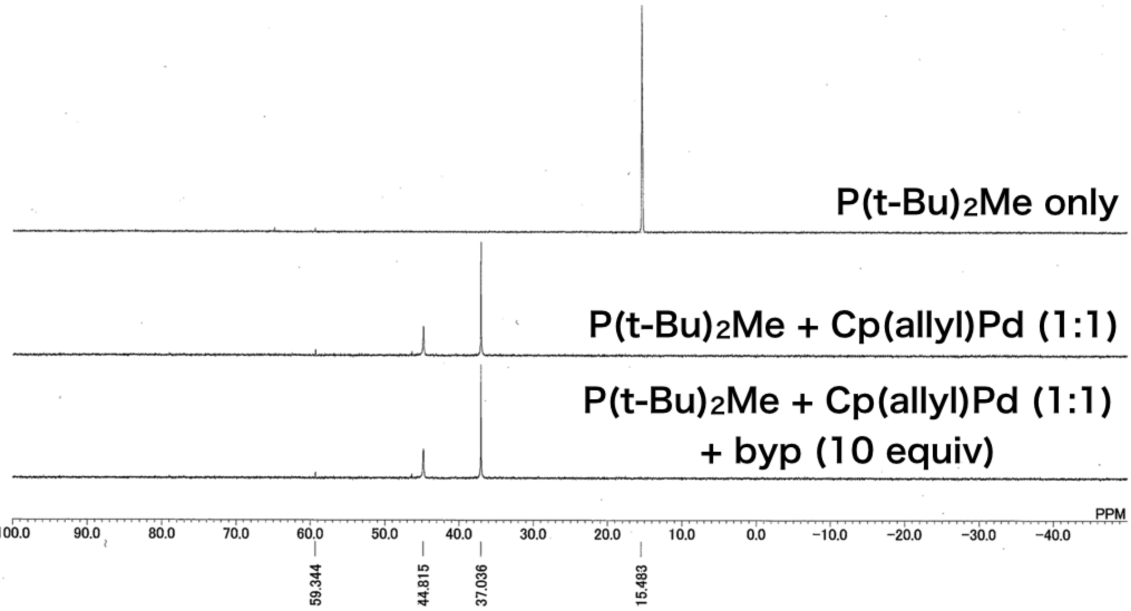

Figure S1. $^{31}P\{^1H\}$ NMR spectra of a) *Experiment 1* and b) *Experiment 2*.

Procedures for the borylation of 1a using the Pd catalyst generated in toluene [Pd_2L_2(μ-Cp)(μ-allyl) or PdL_2]. In a glove box, to a NMR sample tube fitted with a J. Young Teflon valve were added Cp(allyl)Pd (0.1 M toluene-d_8 solution, 100 μL, 10 μmol, 2 mol%) and P(*t*-Bu)$_2$Me (50 mM toluene-d_8 solution, 200 or 400 μL, 10 or 20 μmol, 2 or 4 mol%). The NMR tube was heated at 80 ˚C for 1 h, allowed for cooling to room temperature, and removed from glove box. After the quantitative generation of Pd species was confirmed by $^{31}P\{^1H\}$ NMR [Pd_2L_2(μ-Cp)(μ-allyl)[S12] in the case of L/Pd = 1; PdL_2[S12] in the case of L/Pd = 2] at room temperature, the tube was brought into glove box. The solution was moved into a vial (3 mL) equipped with a stirring bar (10 mm). To the vial, were added toluene (1.0 mL), aziridine **1a** (136.7 mg, 0.5 mmol), $B_2(pin)_2$ (152.7 mg, 0.6 mmol, 1.2 equiv), bpy (16.0 mg, 0.1 mmol, 20 mol%), and the vial was capped with a hole cap and a Teflon®/rubber septum and removed from the glove box. Deionized H_2O (125 μL, 14 equiv) was added through the septum, and the resulting mixture was stirred at 60 ˚C for 3 h. The reaction mixture was diluted with CH_2Cl_2 (10 mL) and washed with brine (5 mL). The organic layer was separated, and the aqueous layer was extracted with CH_2Cl_2 (10 mL × 3). The combined organic layer was dried over Na_2SO_4, and solvent was removed under reduced pressure to give the crude product, which was analyzed by ^1H NMR using 1,1,2,2-tetrachloroethane as an internal standard to determine NMR yields of products.

^{1}H and ^{13}C NMR Charts

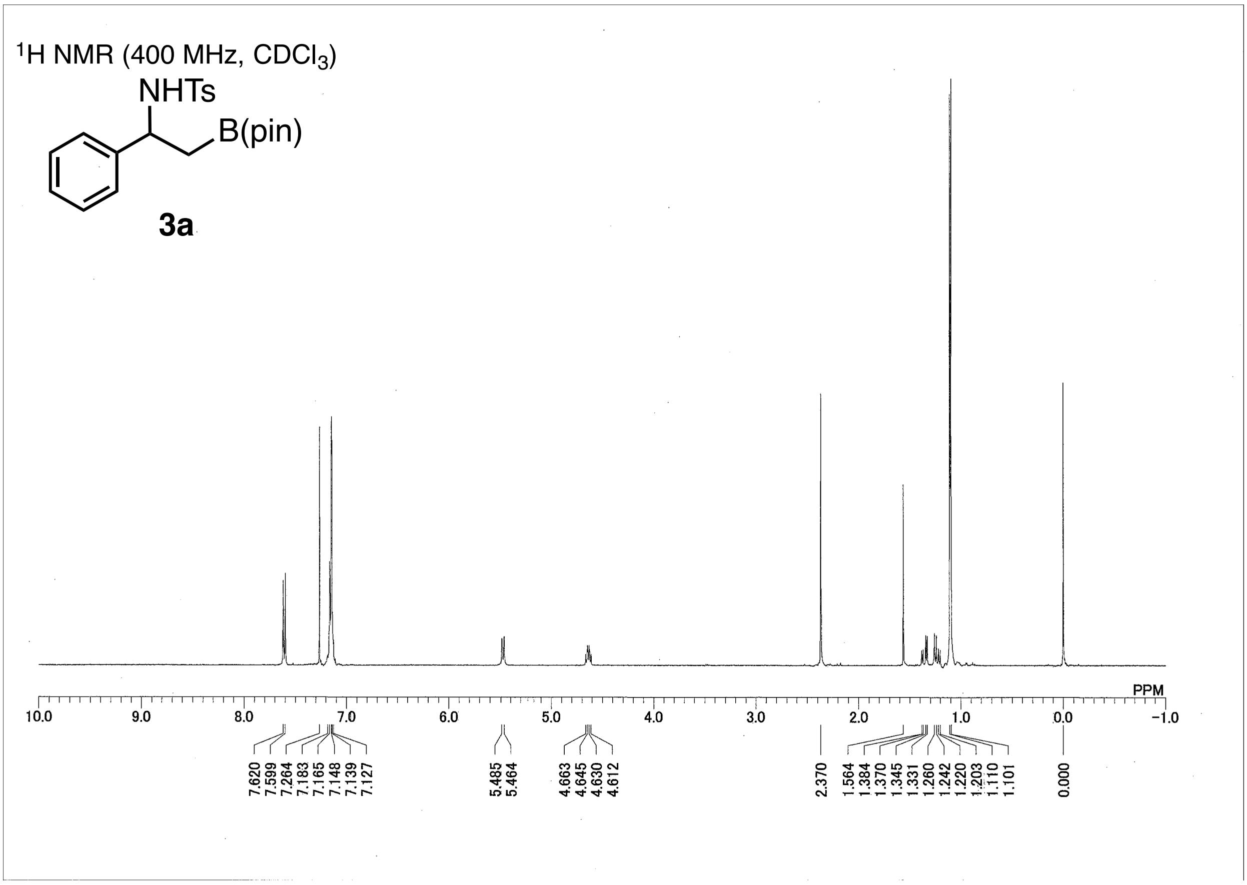

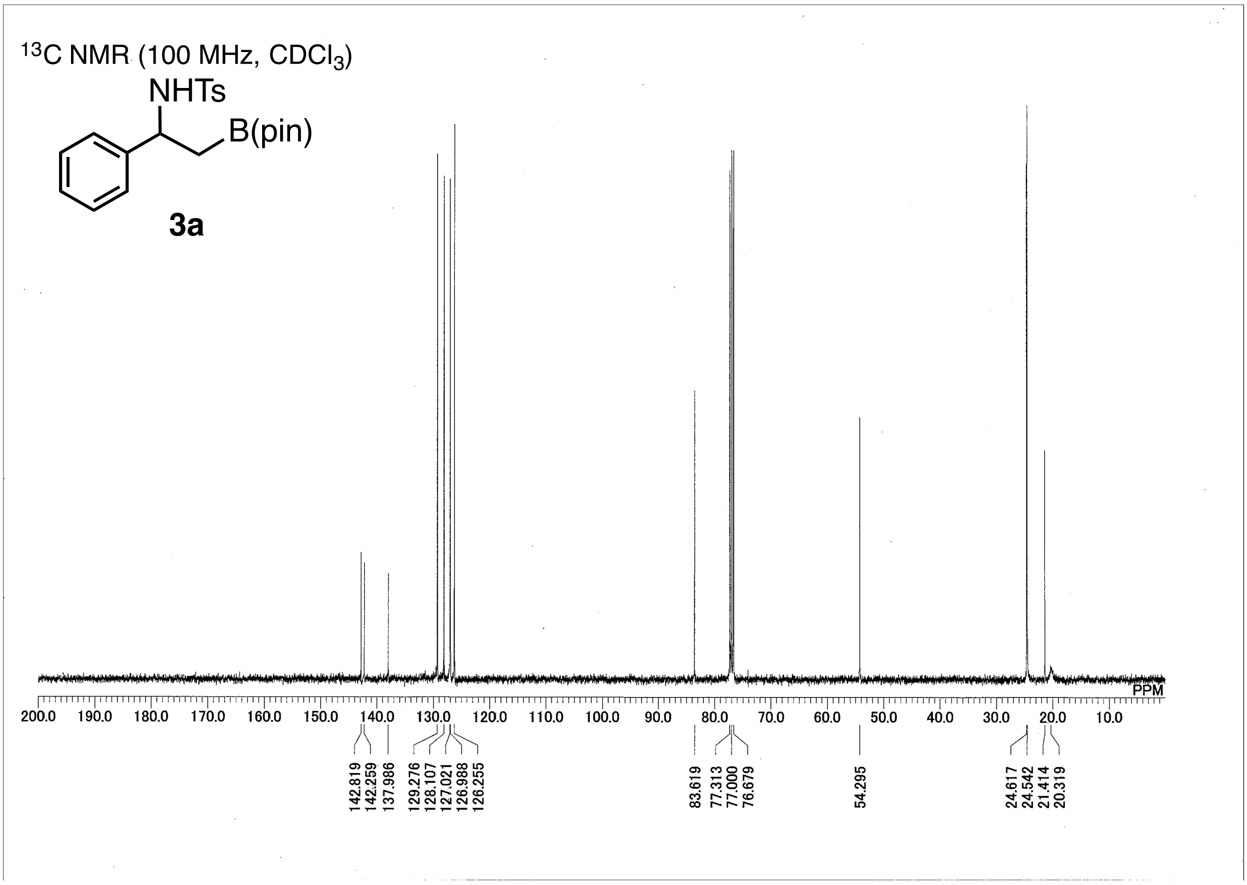

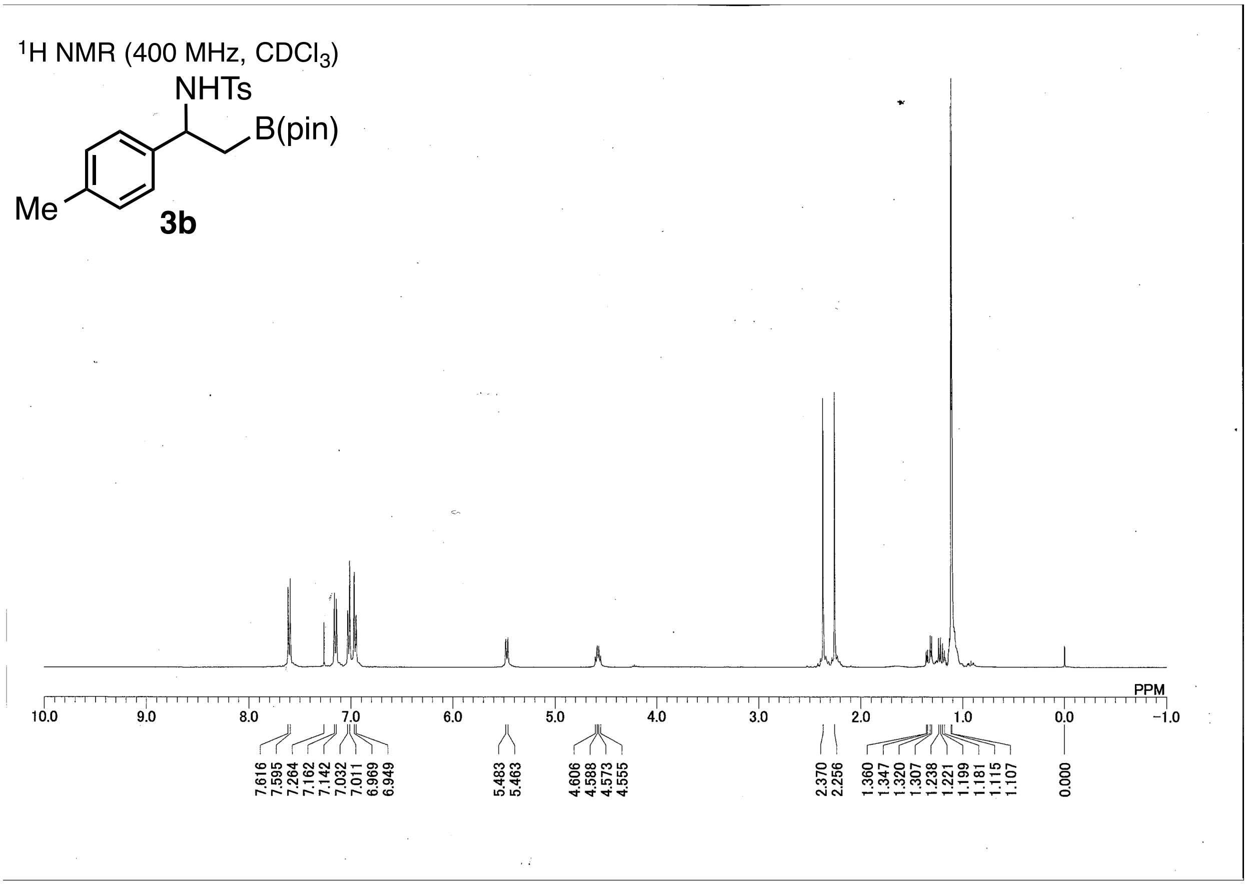

^{1}H NMR (400 MHz, $CDCl_3$)
NHTs
B(pin)
Me
3b
PPM
10.0 9.0 8.0 7.0 6.0 5.0 4.0 3.0 2.0 1.0 0.0 −1.0
7.616 7.595 7.264 7.162 7.142 7.032 7.011 6.969 6.949
5.483 5.463
4.606 4.588 4.573 4.555
2.370 2.256
1.360 1.347 1.320 1.307 1.238 1.221 1.199 1.181 1.115 1.107
0.000

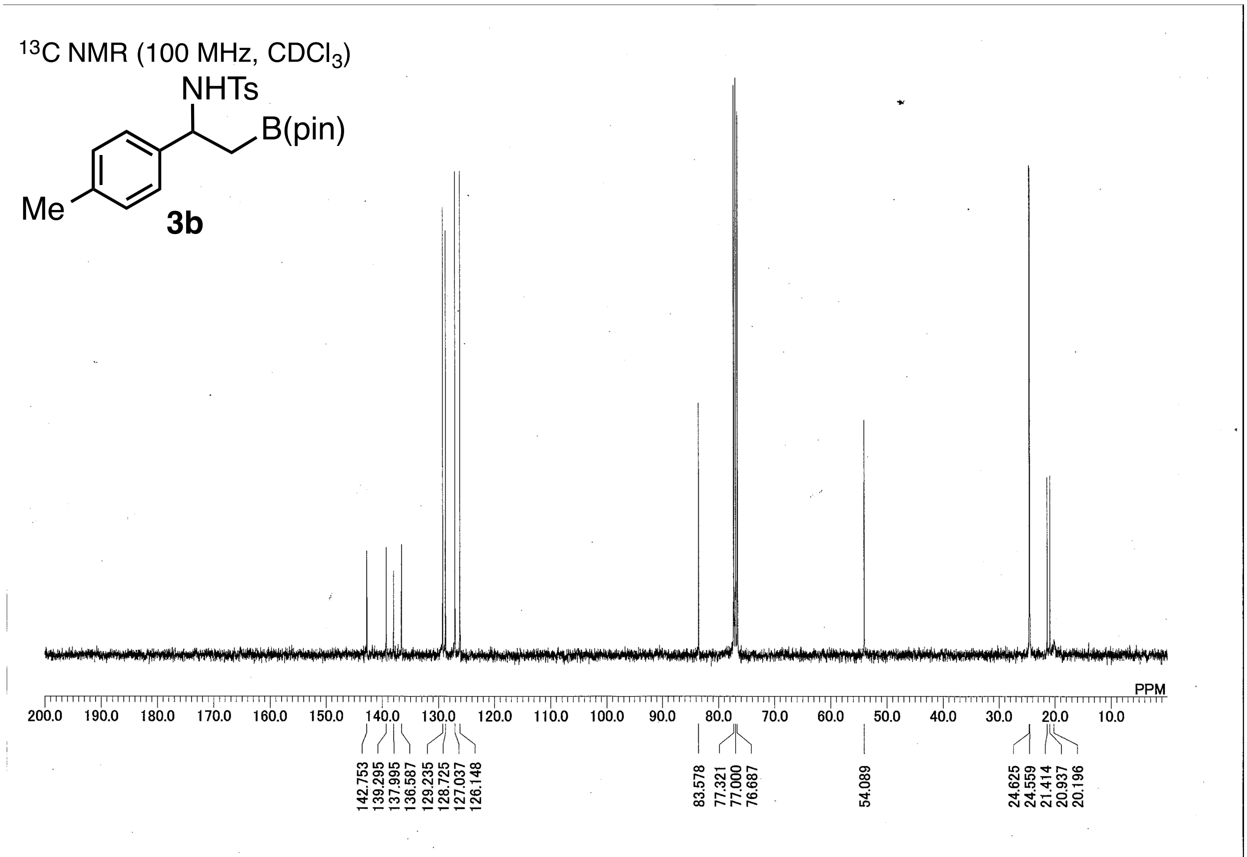

^{13}C NMR (100 MHz, $CDCl_3$)
NHTs
B(pin)
Me
3b
PPM
200.0 190.0 180.0 170.0 160.0 150.0 140.0 130.0 120.0 110.0 100.0 90.0 80.0 70.0 60.0 50.0 40.0 30.0 20.0 10.0
142.753 139.295 137.995 136.587 129.235 128.725 127.037 126.148
83.578 77.321 77.000 76.687
54.089
24.625 24.559 21.414 20.937 20.196

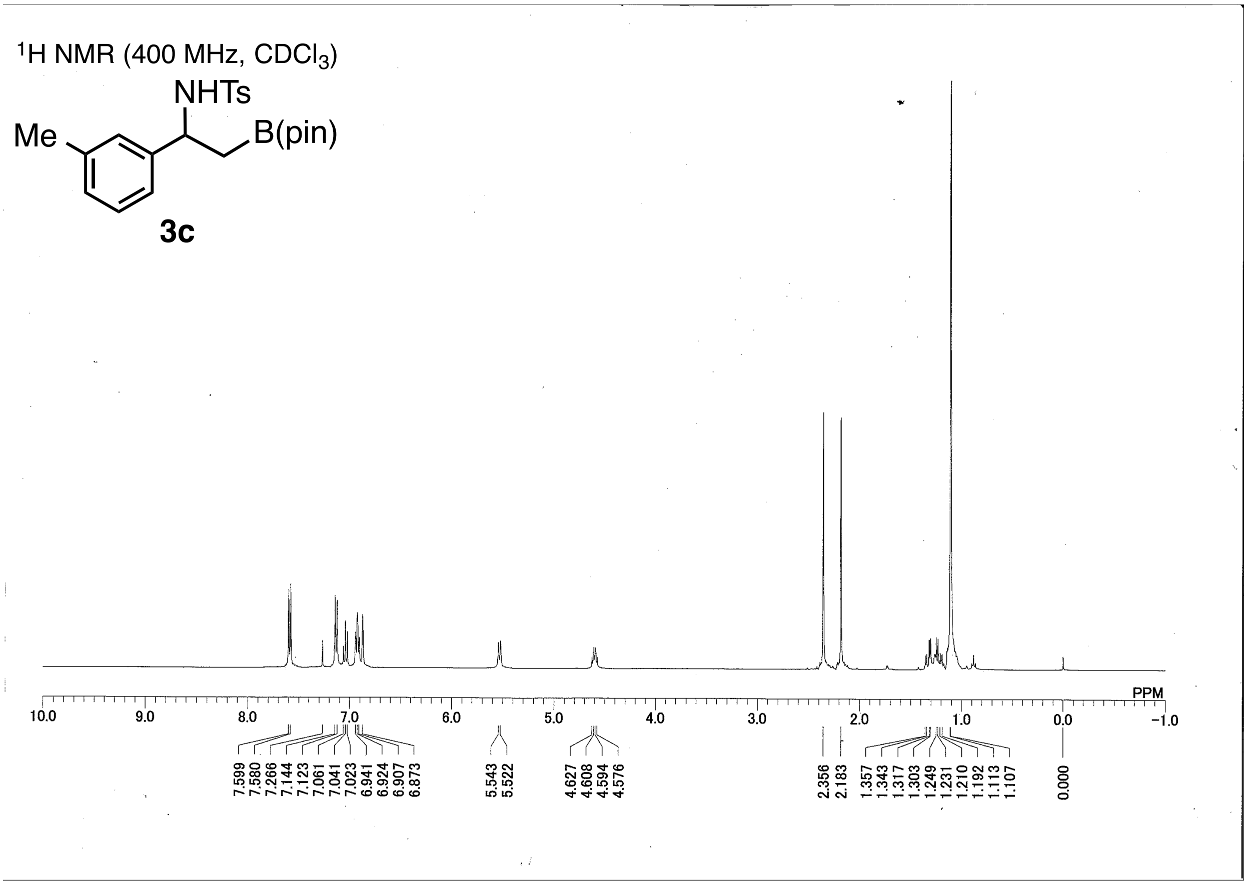

1H NMR (400 MHz, CDCl3)
NHTs
Me
B(pin)
3c
PPM
7.599
7.580
7.266
7.144
7.123
7.061
7.041
7.023
6.941
6.924
6.907
6.873
5.543
5.522
4.627
4.608
4.594
4.576
2.356
2.183
1.357
1.343
1.317
1.303
1.249
1.231
1.210
1.192
1.113
1.107
0.000

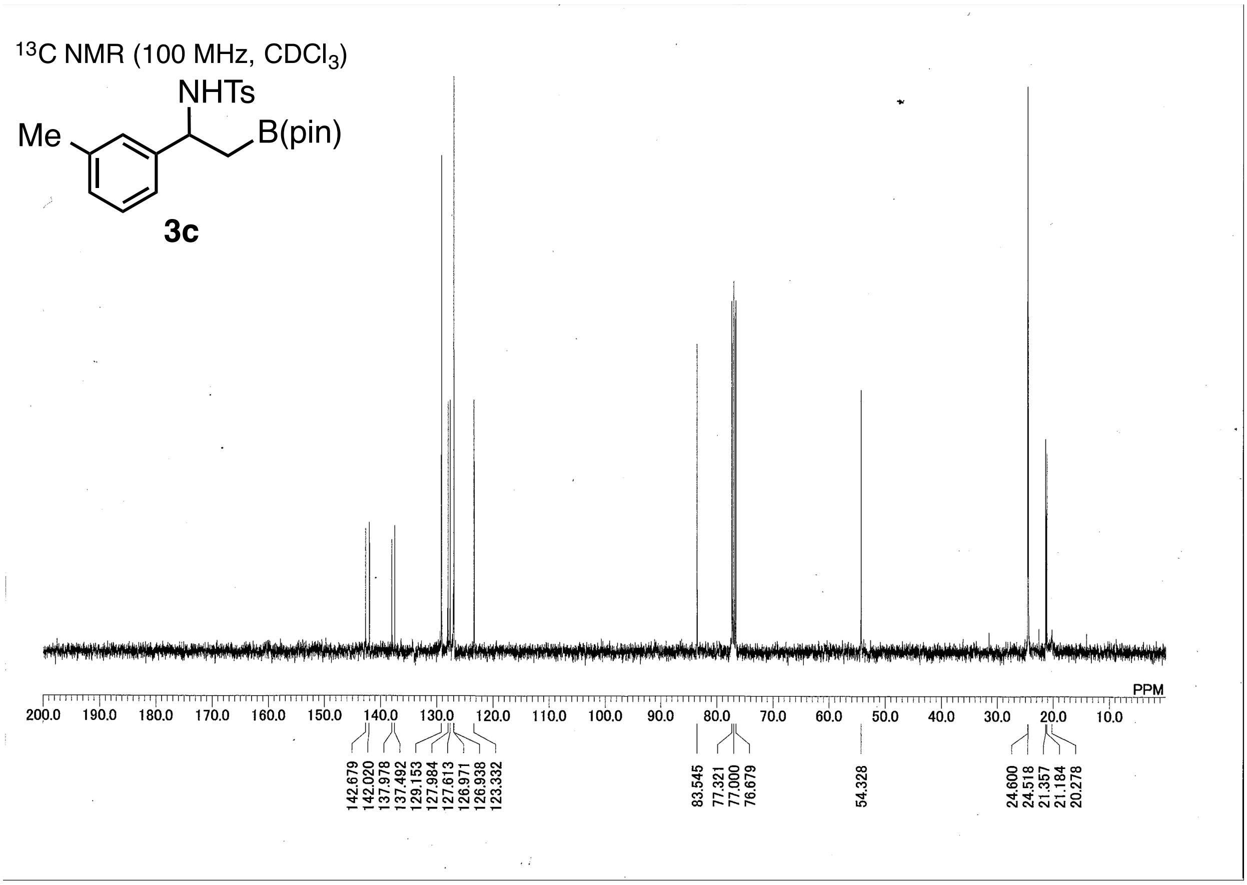

13C NMR (100 MHz, CDCl3)
NHTs
Me
B(pin)
3c
PPM
142.679
142.020
137.978
137.492
129.153
127.984
127.613
126.971
126.938
123.332
83.545
77.321
77.000
76.679
54.328
24.600
24.518
21.357
21.184
20.278

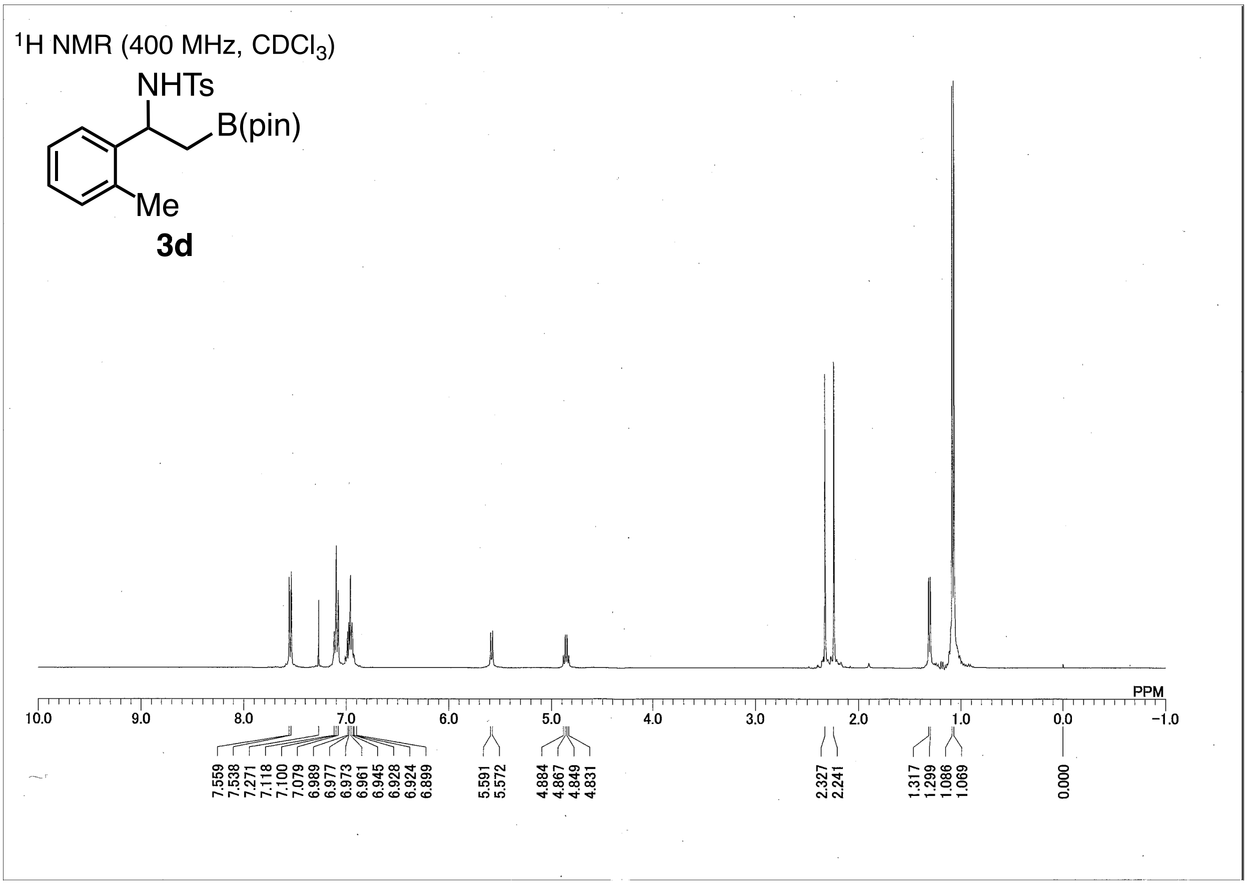

^{1}H NMR (400 MHz, $CDCl_3$)
NHTs
B(pin)
Me
3d
PPM

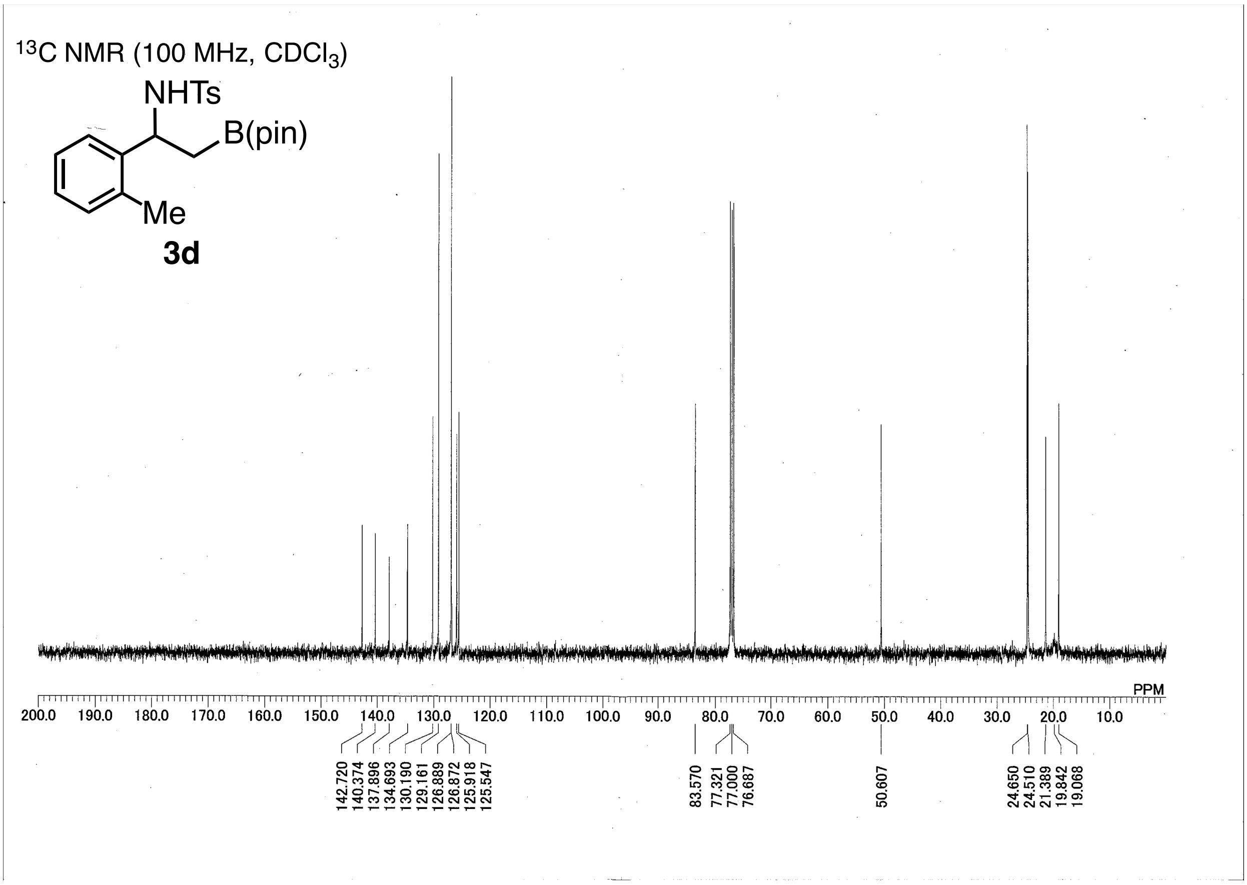

^{13}C NMR (100 MHz, $CDCl_3$)
NHTs
B(pin)
Me
3d
PPM

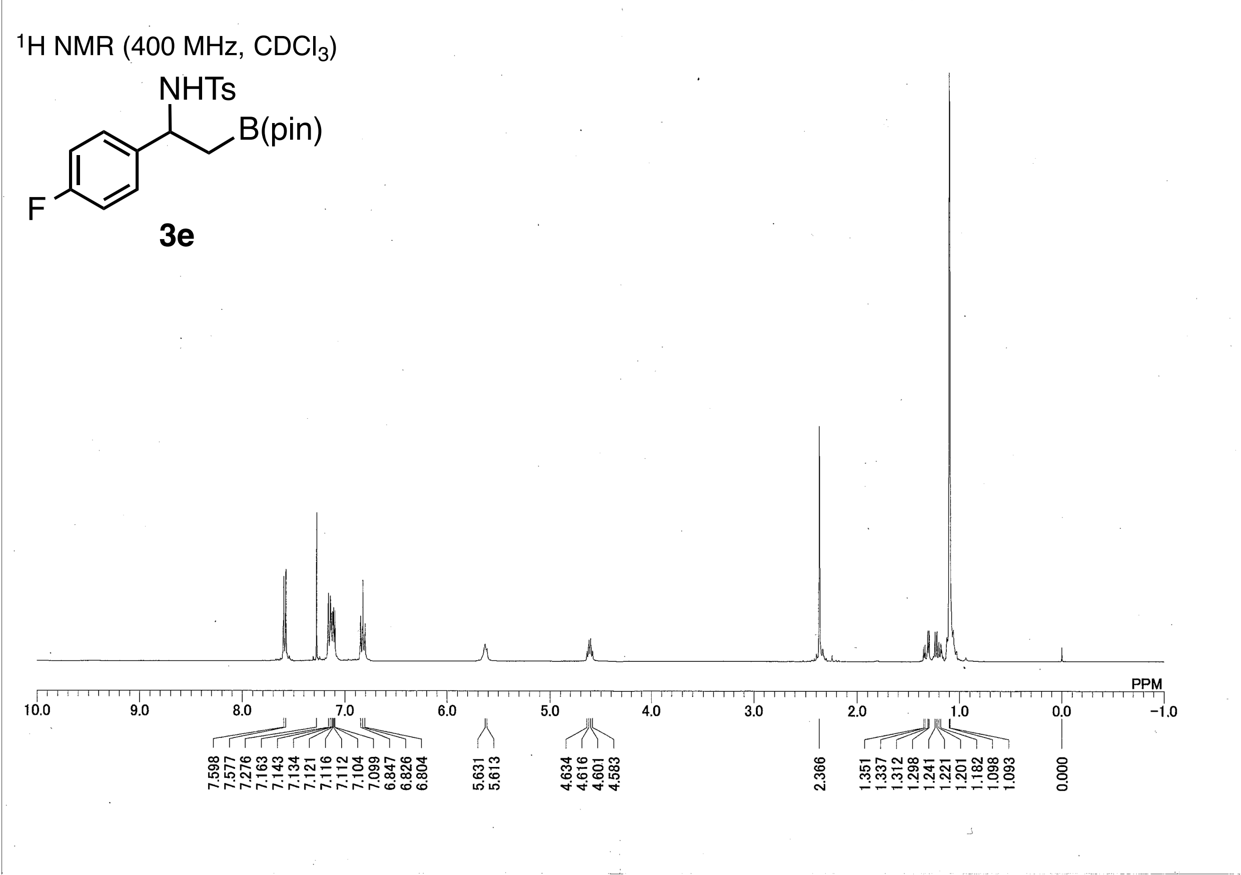

1H NMR (400 MHz, CDCl3)
NHTs
B(pin)
F
3e
PPM
10.0 9.0 8.0 7.0 6.0 5.0 4.0 3.0 2.0 1.0 0.0 −1.0
7.598 7.577 7.276 7.163 7.143 7.134 7.121 7.116 7.112 7.104 7.099 6.847 6.826 6.804
5.631 5.613
4.634 4.616 4.601 4.583
2.366
1.351 1.337 1.312 1.298 1.241 1.221 1.201 1.182 1.098 1.093
0.000

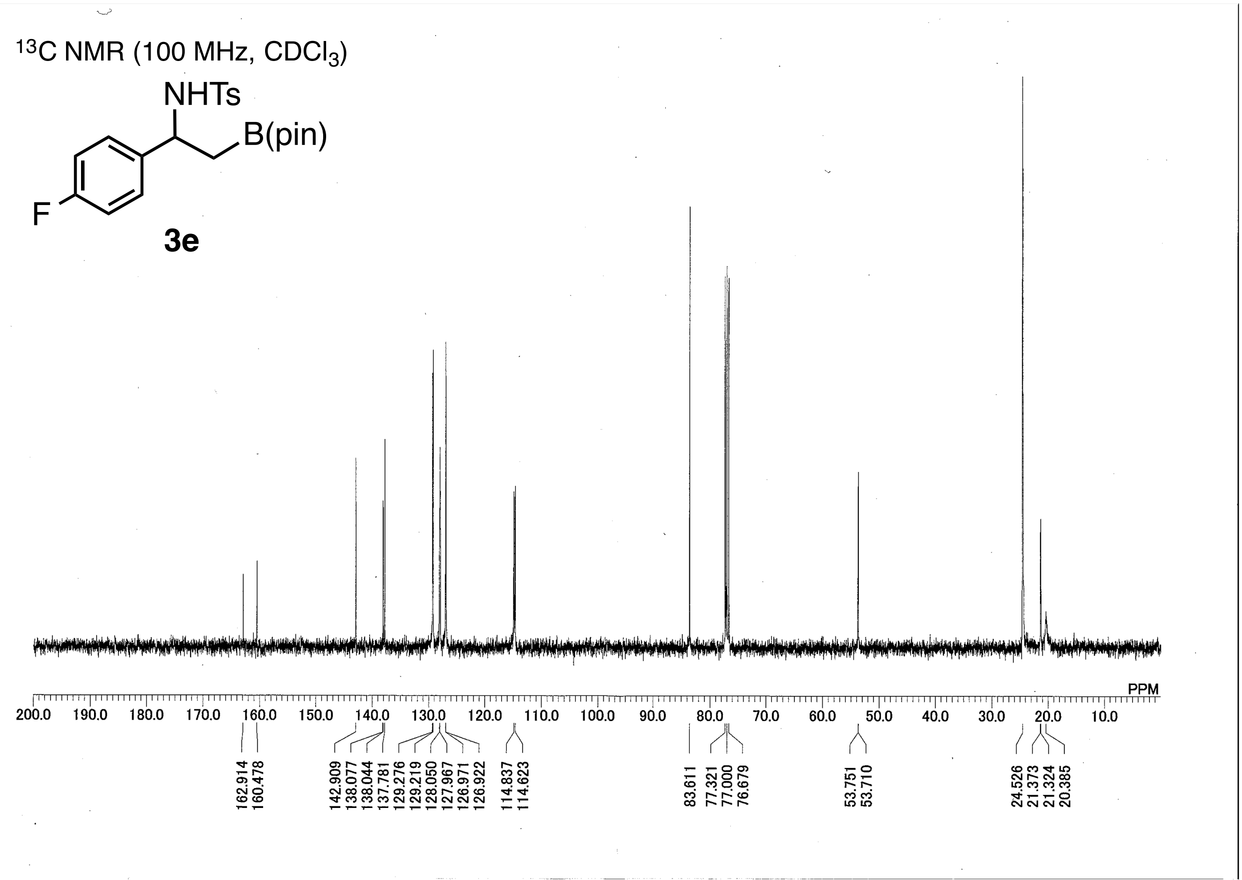

13C NMR (100 MHz, CDCl3)
NHTs
B(pin)
F
3e
PPM
200.0 190.0 180.0 170.0 160.0 150.0 140.0 130.0 120.0 110.0 100.0 90.0 80.0 70.0 60.0 50.0 40.0 30.0 20.0 10.0
162.914 160.478
142.909 138.077 138.044 137.781 129.276 129.219 128.050 127.967 126.971 126.922
114.837 114.623
83.611 77.321 77.000 76.679
53.751 53.710
24.526 21.373 21.324 20.385

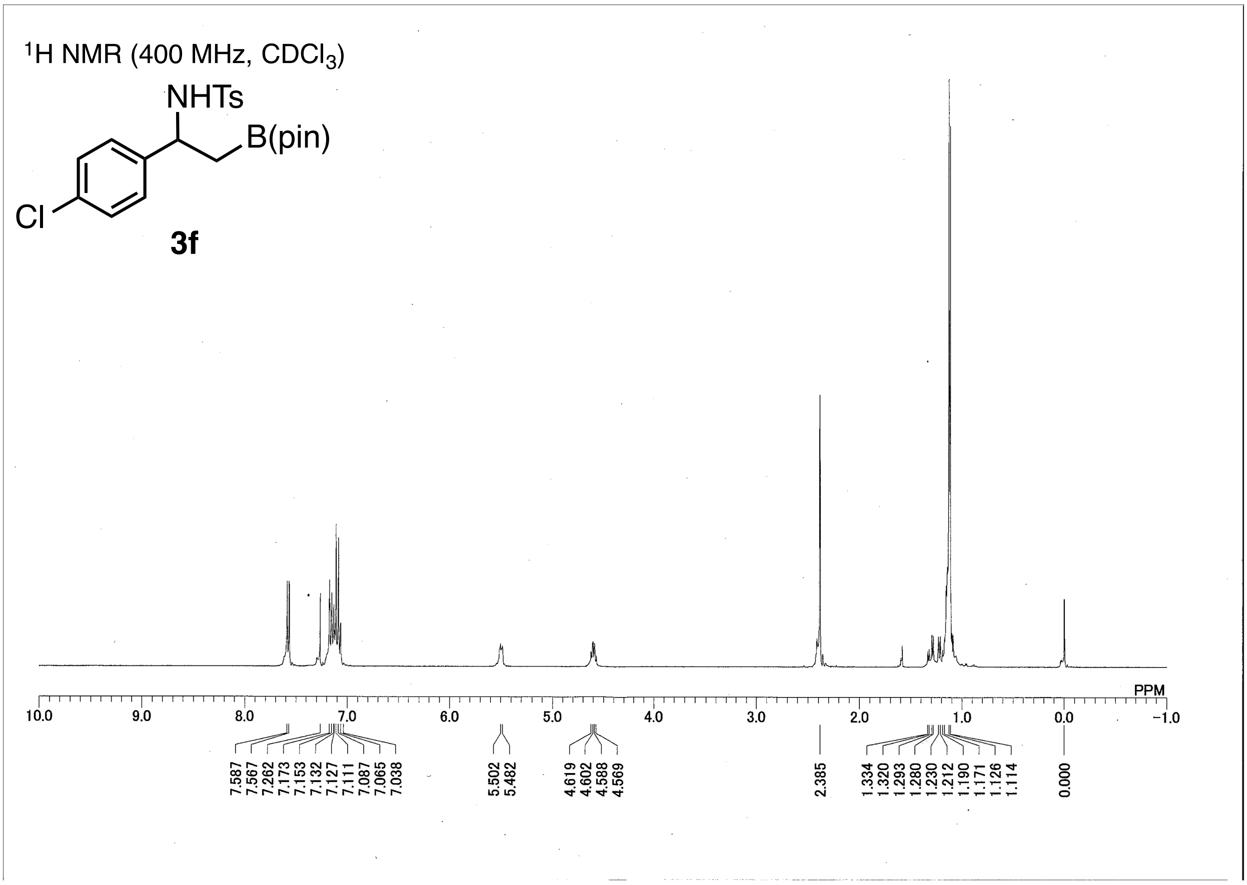

1H NMR (400 MHz, CDCl3)
NHTs
B(pin)
Cl
3f
PPM
7.587
7.567
7.262
7.173
7.153
7.132
7.127
7.111
7.087
7.065
7.038
5.502
5.482
4.619
4.602
4.588
4.569
2.385
1.334
1.320
1.293
1.280
1.230
1.212
1.190
1.171
1.126
1.114
0.000

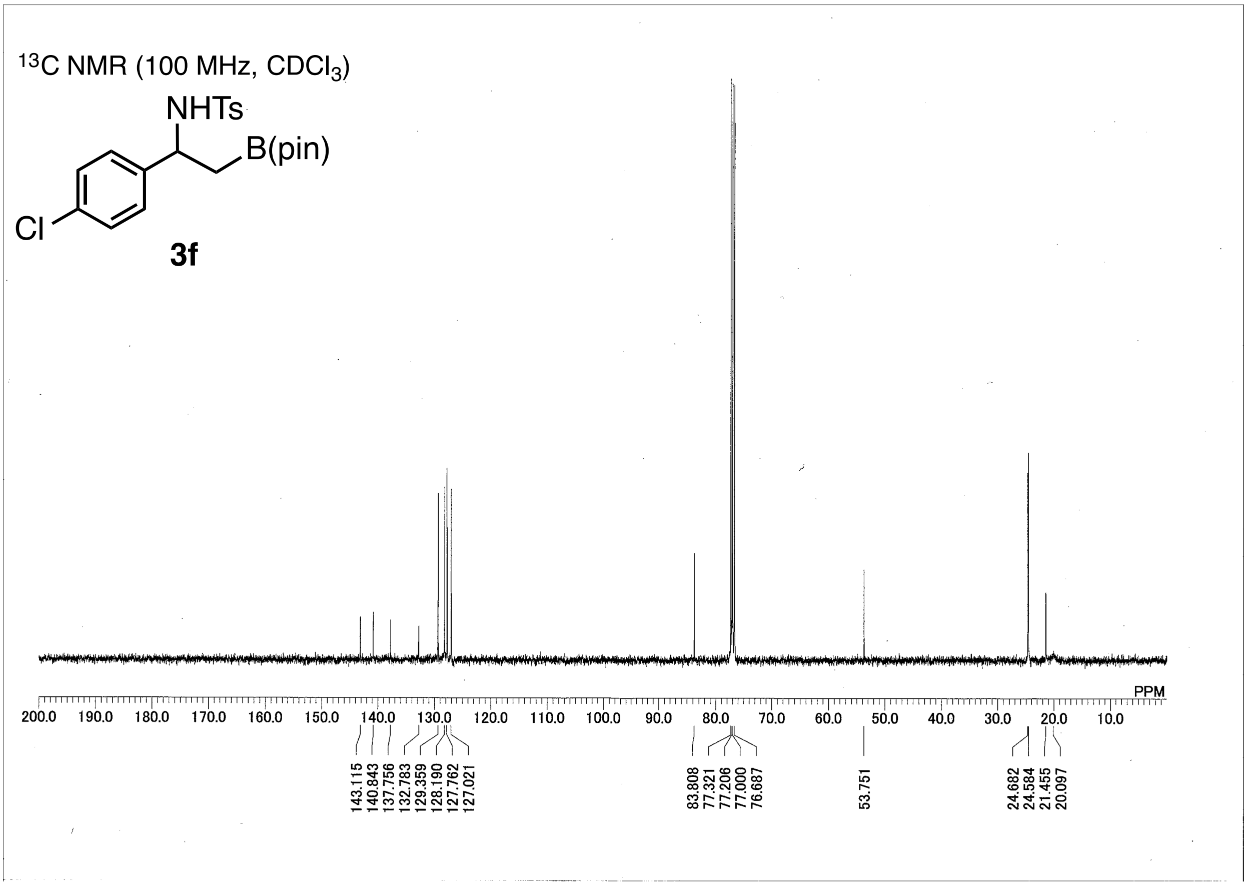

13C NMR (100 MHz, CDCl3)
NHTs
B(pin)
Cl
3f
PPM
143.115
140.843
137.756
132.783
129.359
128.190
127.762
127.021
83.808
77.321
77.206
77.000
76.687
53.751
24.682
24.584
21.455
20.097

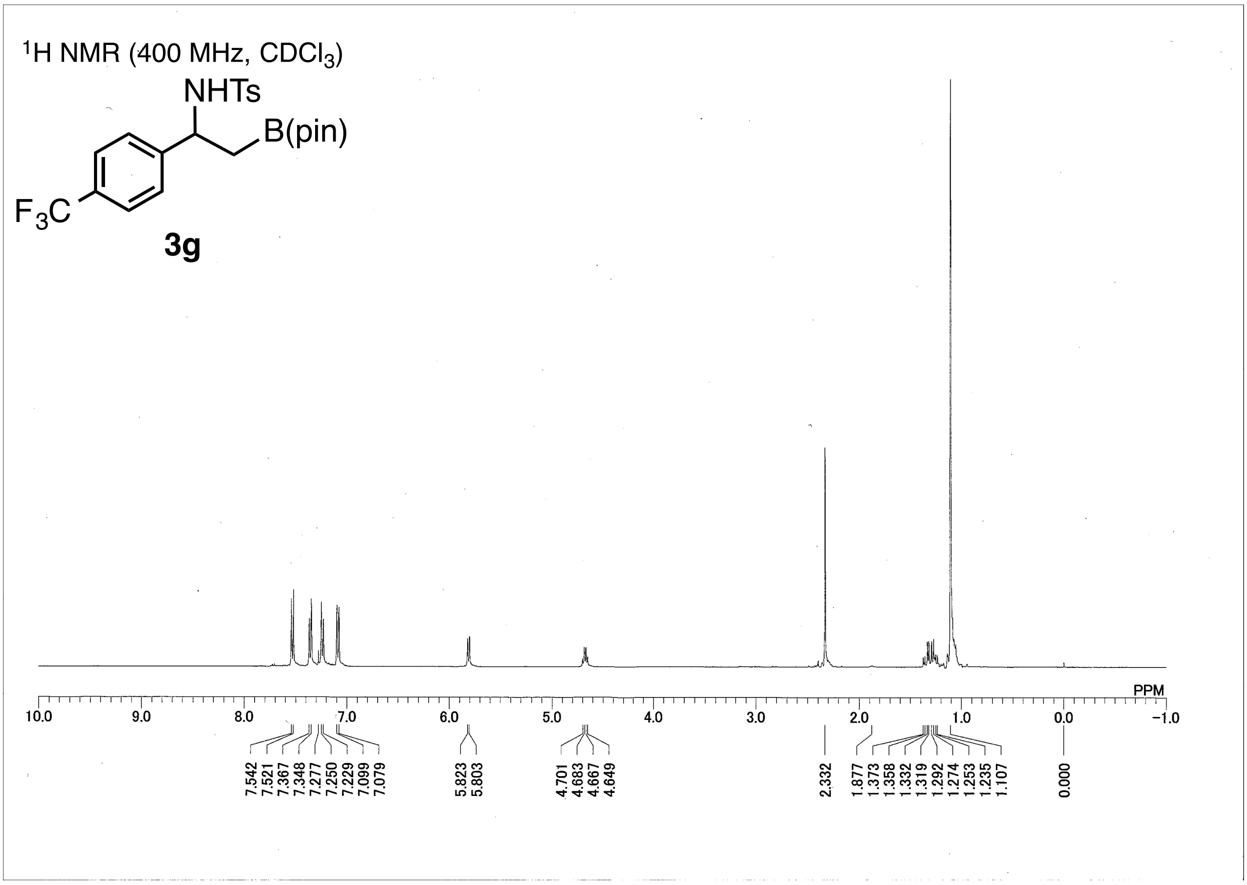

1H NMR (400 MHz, CDCl3)
NHTs
B(pin)
F3C
3g
PPM
7.542
7.521
7.367
7.348
7.277
7.250
7.229
7.099
7.079
5.823
5.803
4.701
4.683
4.667
4.649
2.332
1.877
1.373
1.358
1.332
1.319
1.292
1.274
1.253
1.235
1.107
0.000

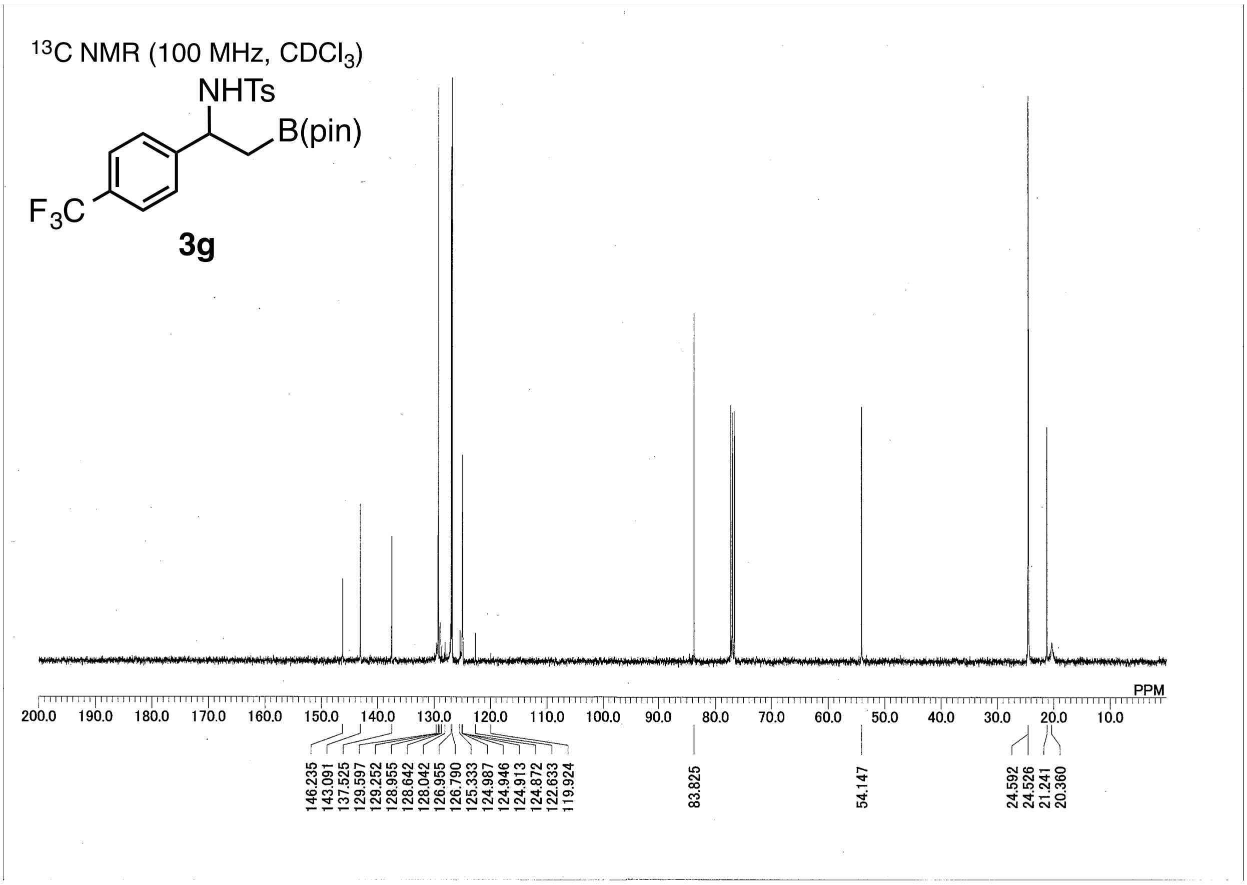

13C NMR (100 MHz, CDCl3)
NHTs
B(pin)
F3C
3g
PPM
146.235
143.091
137.525
129.597
129.252
128.955
128.642
128.042
126.955
126.790
125.333
124.987
124.946
124.913
124.872
122.633
119.924
83.825
54.147
24.592
24.526
21.241
20.360

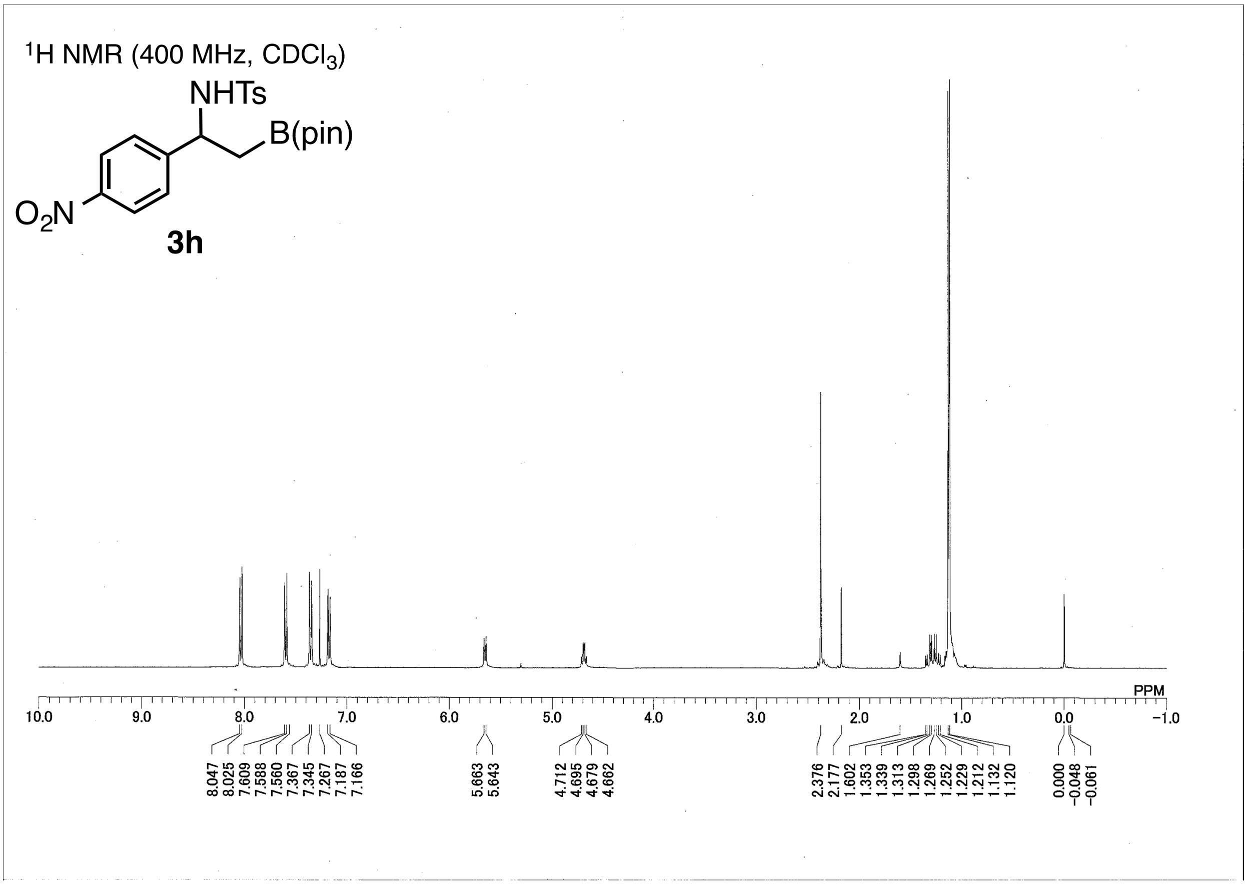

1H NMR (400 MHz, CDCl3)
NHTs
B(pin)
O2N
3h
PPM
10.0 9.0 8.0 7.0 6.0 5.0 4.0 3.0 2.0 1.0 0.0 −1.0
8.047 8.025 7.609 7.588 7.560 7.367 7.345 7.267 7.187 7.166
5.663 5.643
4.712 4.695 4.679 4.662
2.376 2.177 1.602 1.353 1.339 1.313 1.298 1.269 1.252 1.229 1.212 1.132 1.120
0.000 −0.048 −0.061

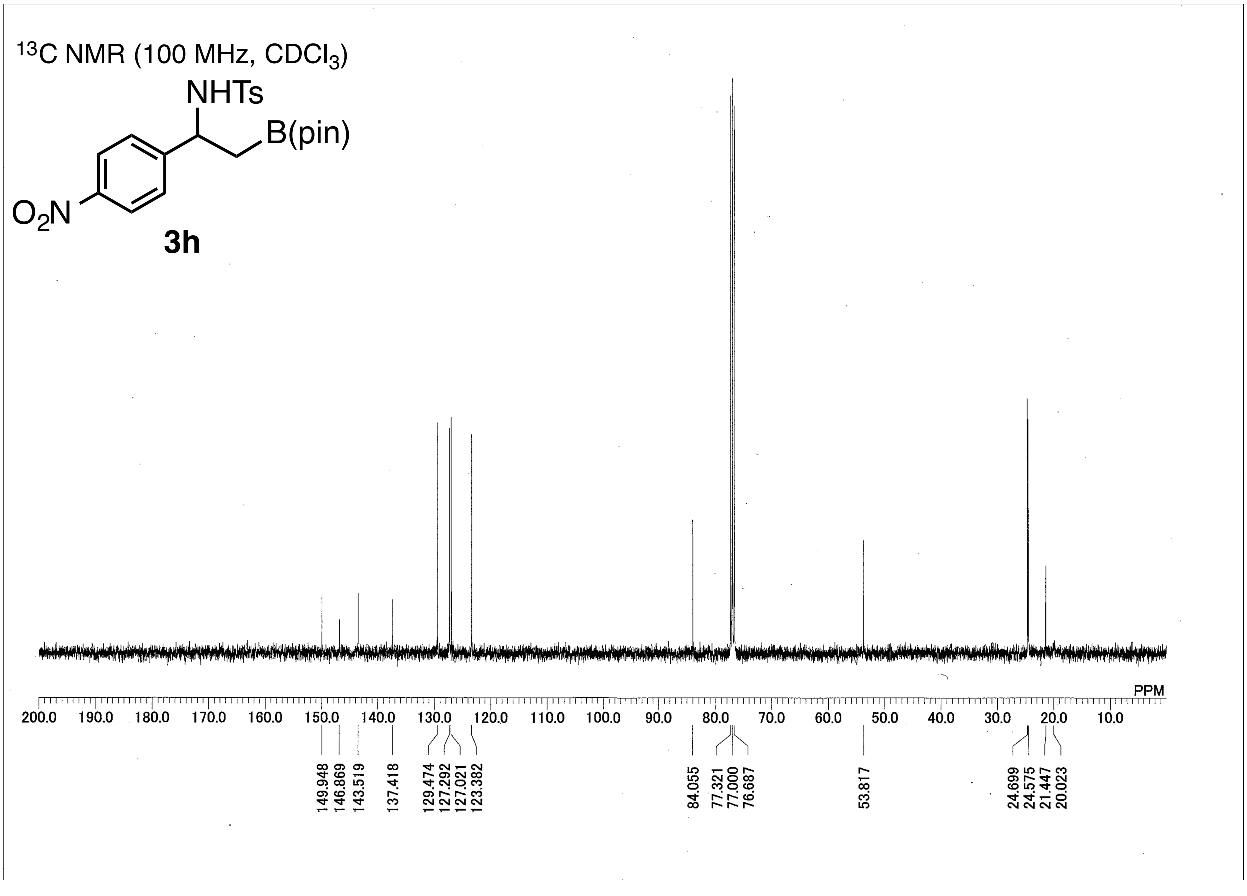

13C NMR (100 MHz, CDCl3)
NHTs
B(pin)
O2N
3h
PPM
200.0 190.0 180.0 170.0 160.0 150.0 140.0 130.0 120.0 110.0 100.0 90.0 80.0 70.0 60.0 50.0 40.0 30.0 20.0 10.0
149.948 146.869 143.519 137.418 129.474 127.292 127.021 123.382
84.055 77.321 77.000 76.687
53.817
24.699 24.575 21.447 20.023

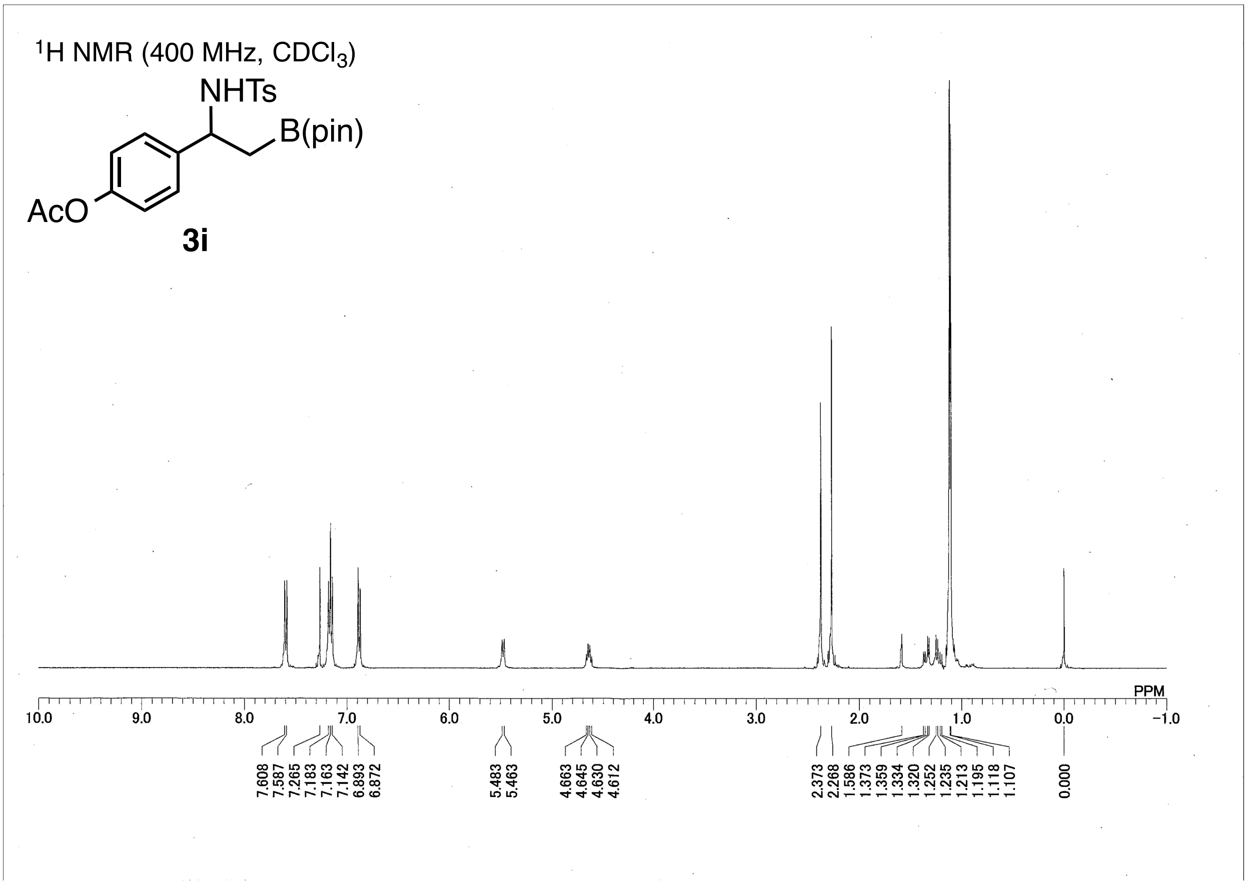

^{1}H NMR (400 MHz, CDCl$_3$)
NHTs
B(pin)
AcO
3i
PPM
7.608 7.587 7.265 7.183 7.163 7.142 6.893 6.872
5.483 5.463
4.663 4.645 4.630 4.612
2.373 2.268 1.586 1.373 1.359 1.334 1.320 1.252 1.235 1.213 1.195 1.118 1.107
0.000

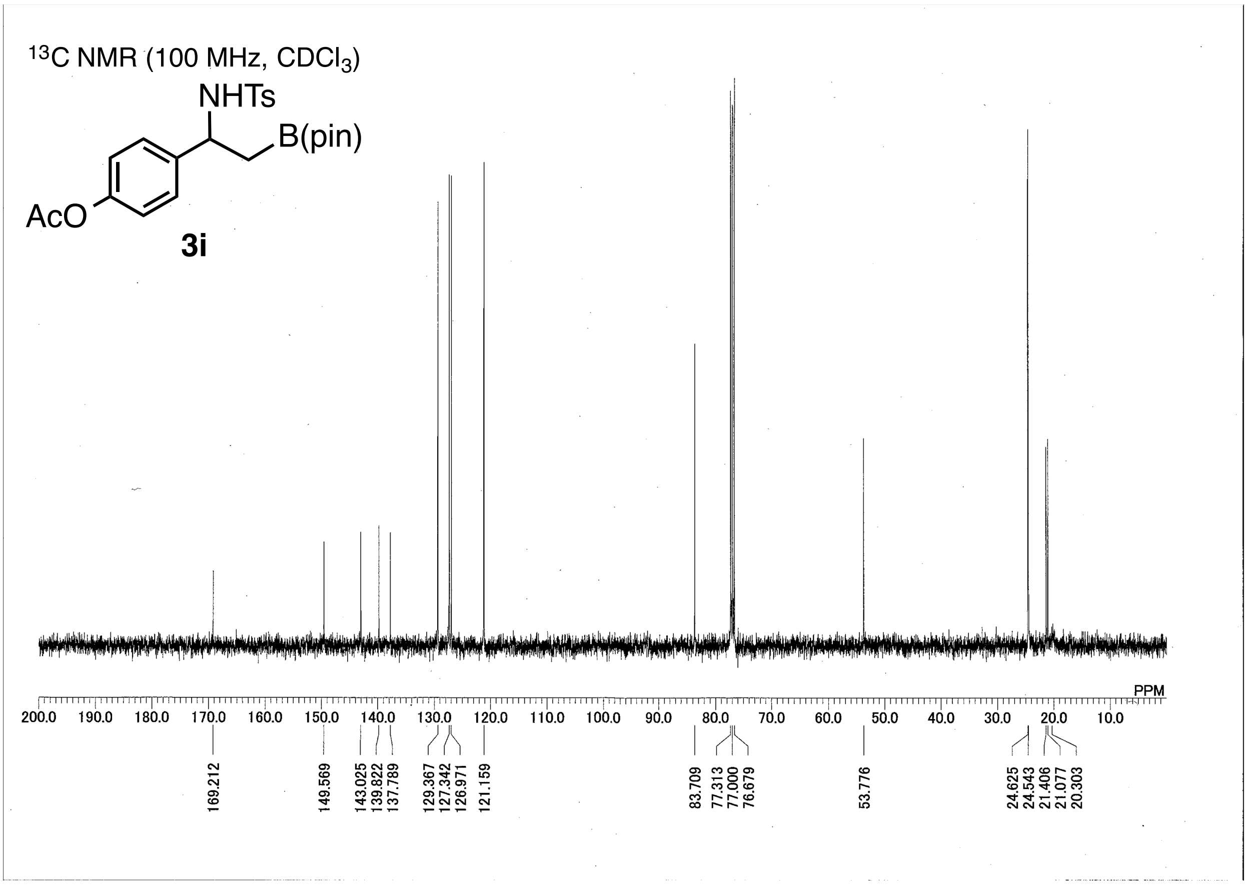

^{13}C NMR (100 MHz, CDCl$_3$)
NHTs
B(pin)
AcO
3i
PPM
169.212
149.569
143.025 139.822 137.789
129.367 127.342 126.971
121.159
83.709
77.313 77.000 76.679
53.776
24.625 24.543 21.406 21.077 20.303

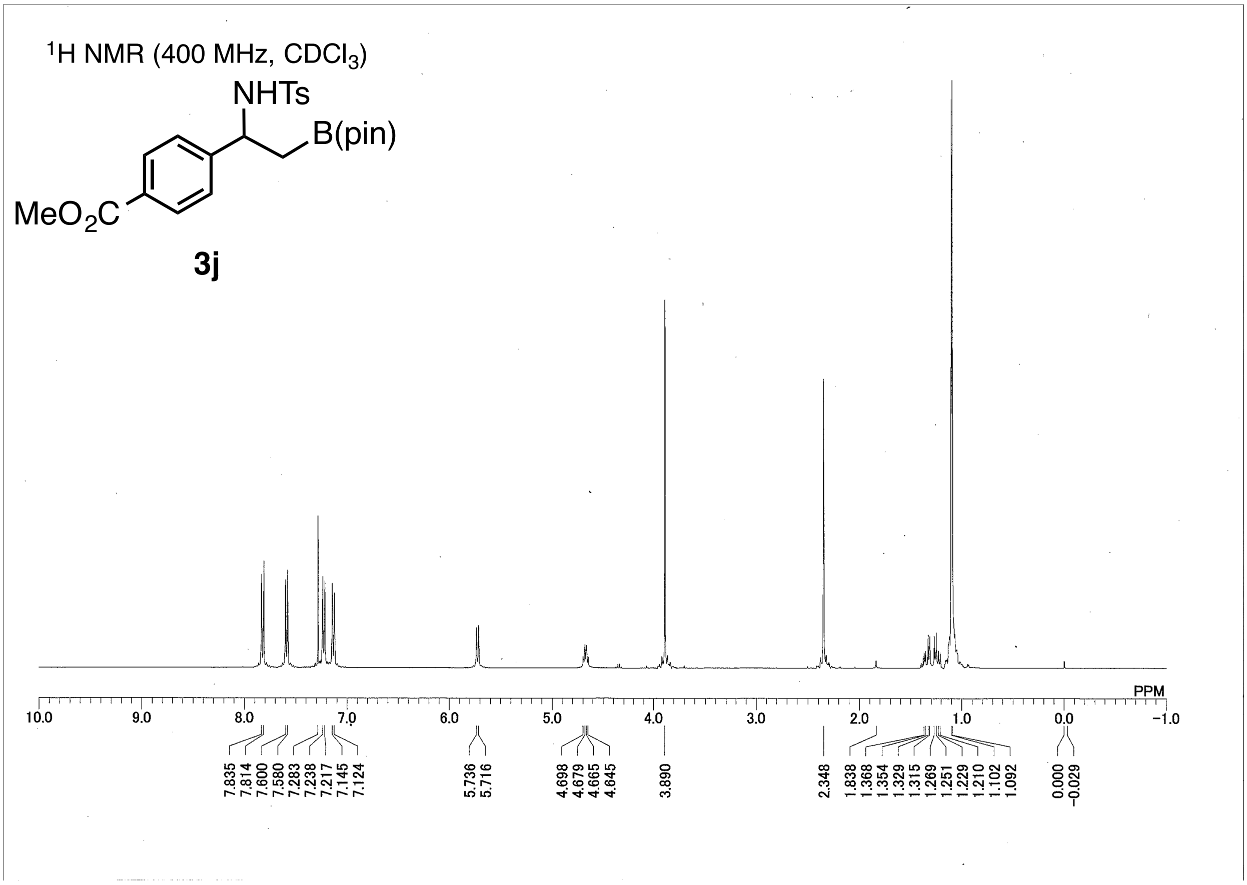

1H NMR (400 MHz, CDCl3)
NHTs
B(pin)
MeO2C
3j
PPM
10.0 9.0 8.0 7.0 6.0 5.0 4.0 3.0 2.0 1.0 0.0 −1.0
7.835 7.814 7.600 7.580 7.283 7.238 7.217 7.145 7.124
5.736 5.716
4.698 4.679 4.665 4.645
3.890
2.348
1.838 1.368 1.354 1.329 1.315 1.269 1.251 1.229 1.210 1.102 1.092
0.000 −0.029

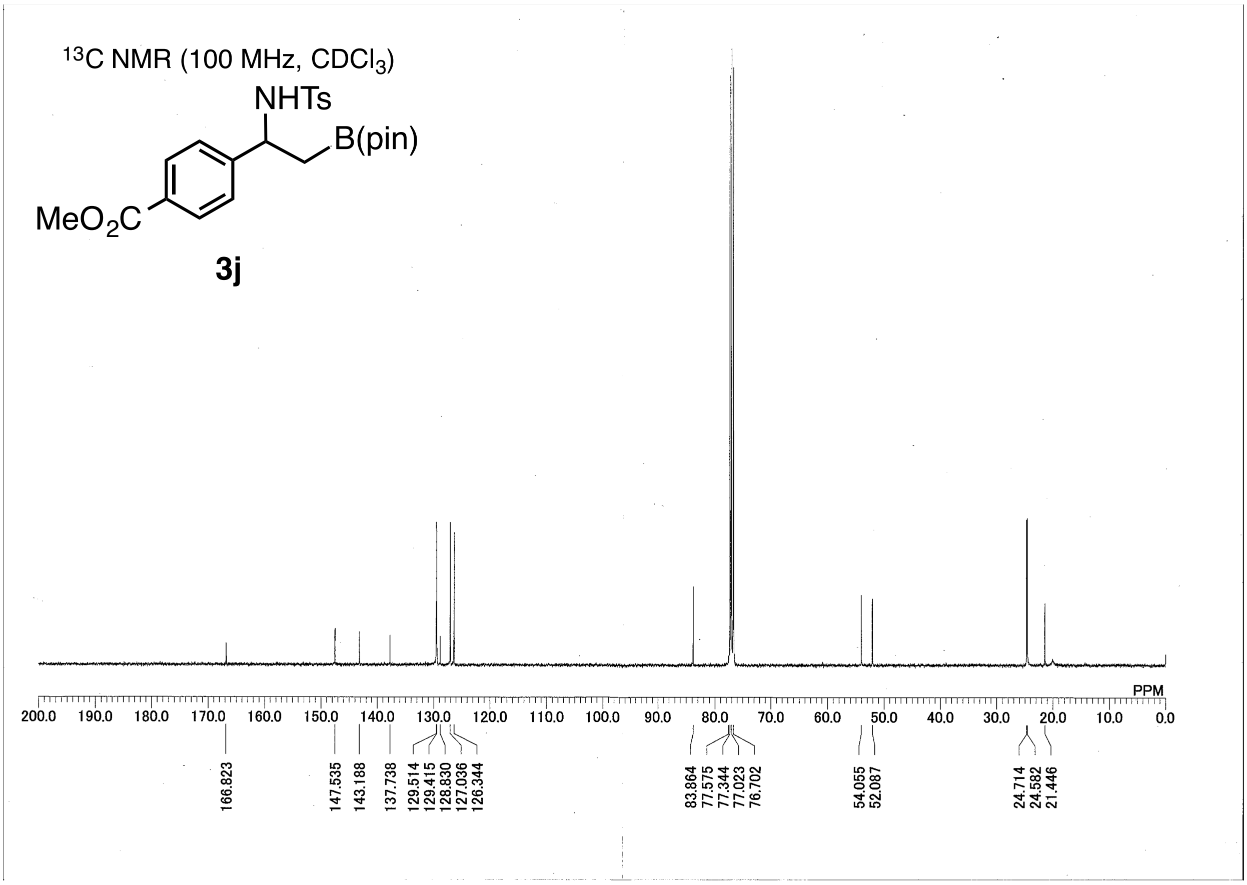

13C NMR (100 MHz, CDCl3)
NHTs
B(pin)
MeO2C
3j
PPM
200.0 190.0 180.0 170.0 160.0 150.0 140.0 130.0 120.0 110.0 100.0 90.0 80.0 70.0 60.0 50.0 40.0 30.0 20.0 10.0 0.0
166.823
147.535
143.188
137.738
129.514 129.415 128.830 127.036 126.344
83.864 77.575 77.344 77.023 76.702
54.055 52.087
24.714 24.582 21.446

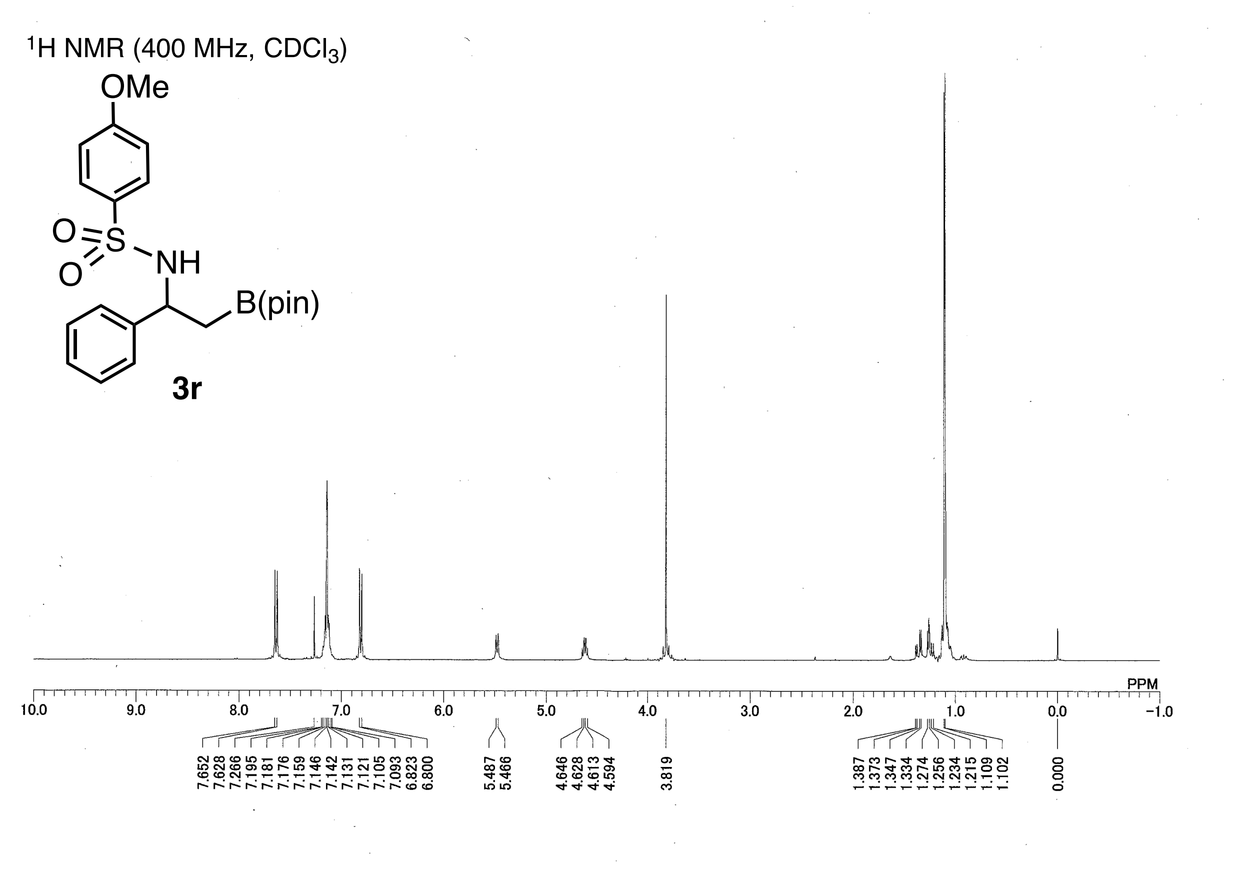

^{1}H NMR (400 MHz, CDCl$_3$)
OMe
O
S
O
NH
B(pin)
3r
PPM
10.0
9.0
8.0
7.0
6.0
5.0
4.0
3.0
2.0
1.0
0.0
−1.0
7.652
7.628
7.266
7.195
7.181
7.176
7.159
7.146
7.142
7.131
7.121
7.105
7.093
6.823
6.800
5.487
5.466
4.646
4.628
4.613
4.594
3.819
1.387
1.373
1.347
1.334
1.274
1.256
1.234
1.215
1.109
1.102
0.000

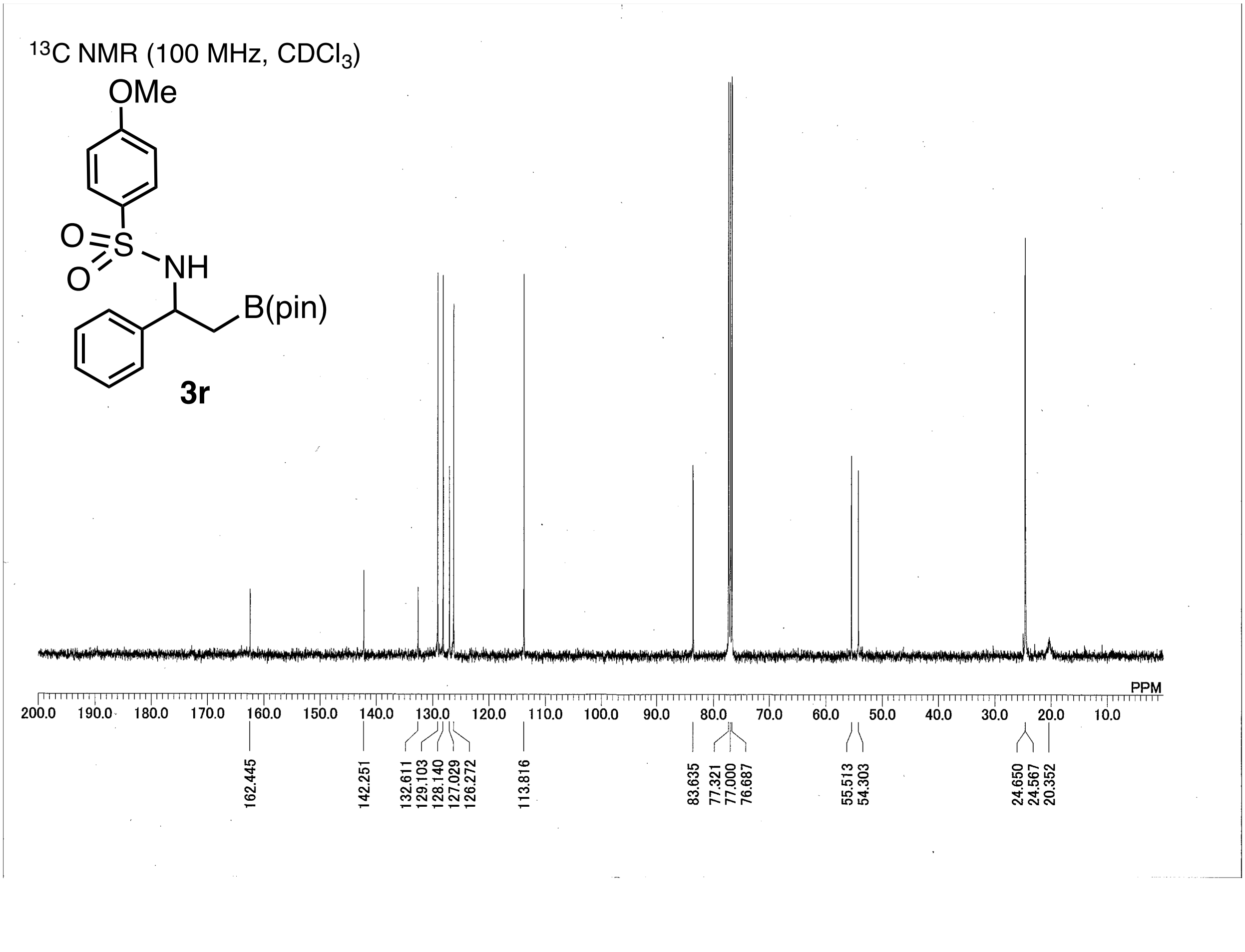

^{13}C NMR (100 MHz, CDCl$_3$)
OMe
O
S
O
NH
B(pin)
3r
PPM
200.0
190.0
180.0
170.0
160.0
150.0
140.0
130.0
120.0
110.0
100.0
90.0
80.0
70.0
60.0
50.0
40.0
30.0
20.0
10.0
162.445
142.251
132.611
129.103
128.140
127.029
126.272
113.816
83.635
77.321
77.000
76.687
55.513
54.303
24.650
24.567
20.352

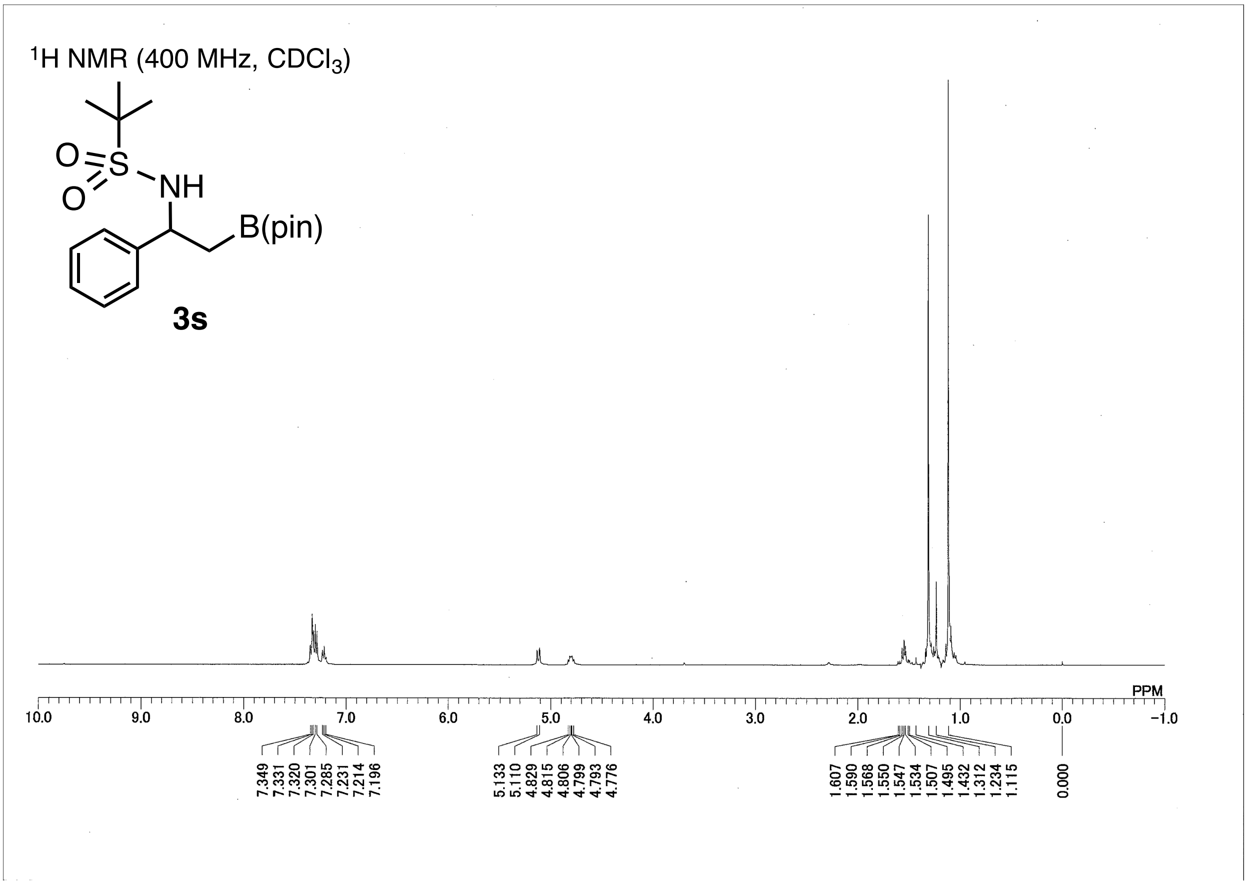

1H NMR (400 MHz, CDCl3)
NH
O
S
O
B(pin)
3s
PPM
10.0
9.0
8.0
7.0
6.0
5.0
4.0
3.0
2.0
1.0
0.0
−1.0
7.349
7.331
7.320
7.301
7.285
7.231
7.214
7.196
5.133
5.110
4.829
4.815
4.806
4.799
4.793
4.776
1.607
1.590
1.568
1.550
1.547
1.534
1.507
1.495
1.432
1.312
1.234
1.115
0.000

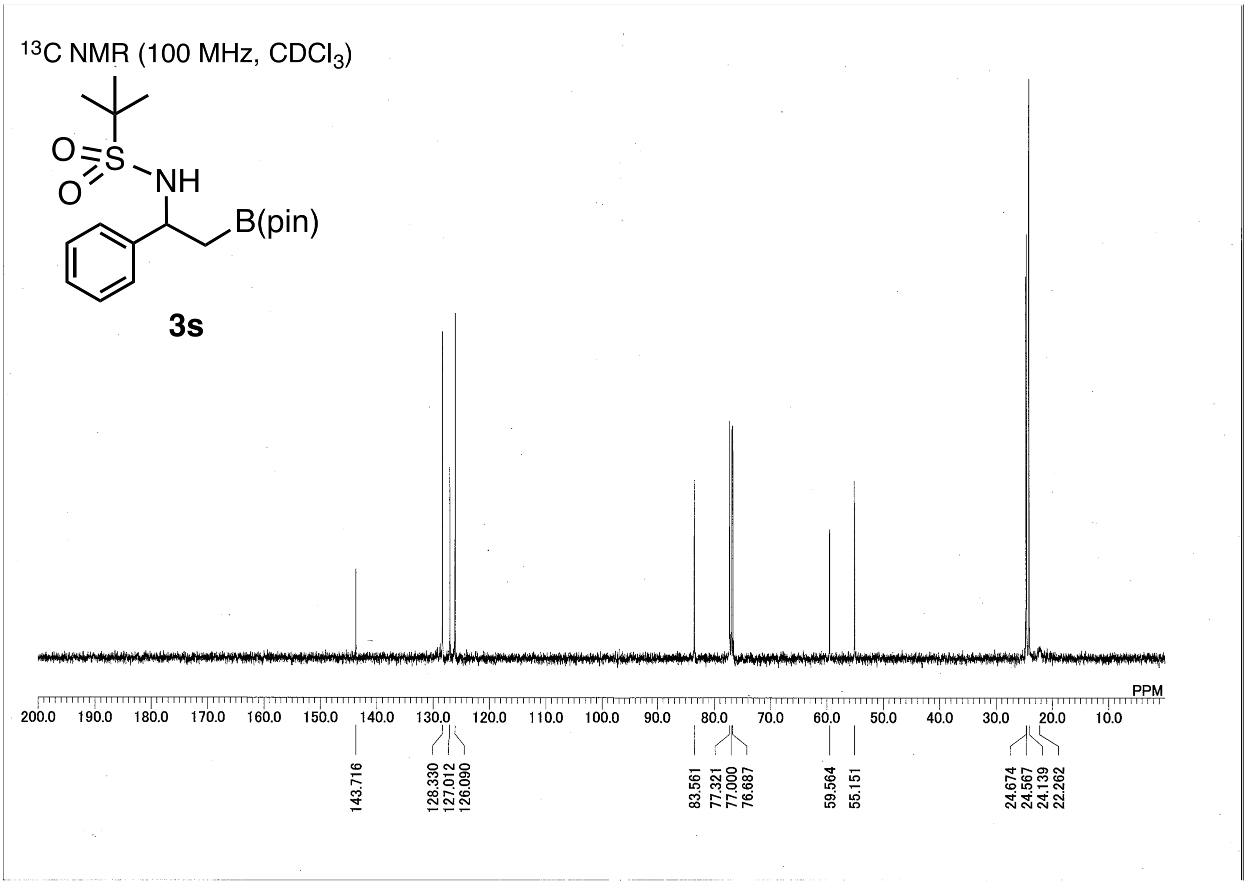

13C NMR (100 MHz, CDCl3)
NH
O
S
O
B(pin)
3s
PPM
200.0
190.0
180.0
170.0
160.0
150.0
140.0
130.0
120.0
110.0
100.0
90.0
80.0
70.0
60.0
50.0
40.0
30.0
20.0
10.0
143.716
128.330
127.012
126.090
83.561
77.321
77.000
76.687
59.564
55.151
24.674
24.567
24.139
22.262

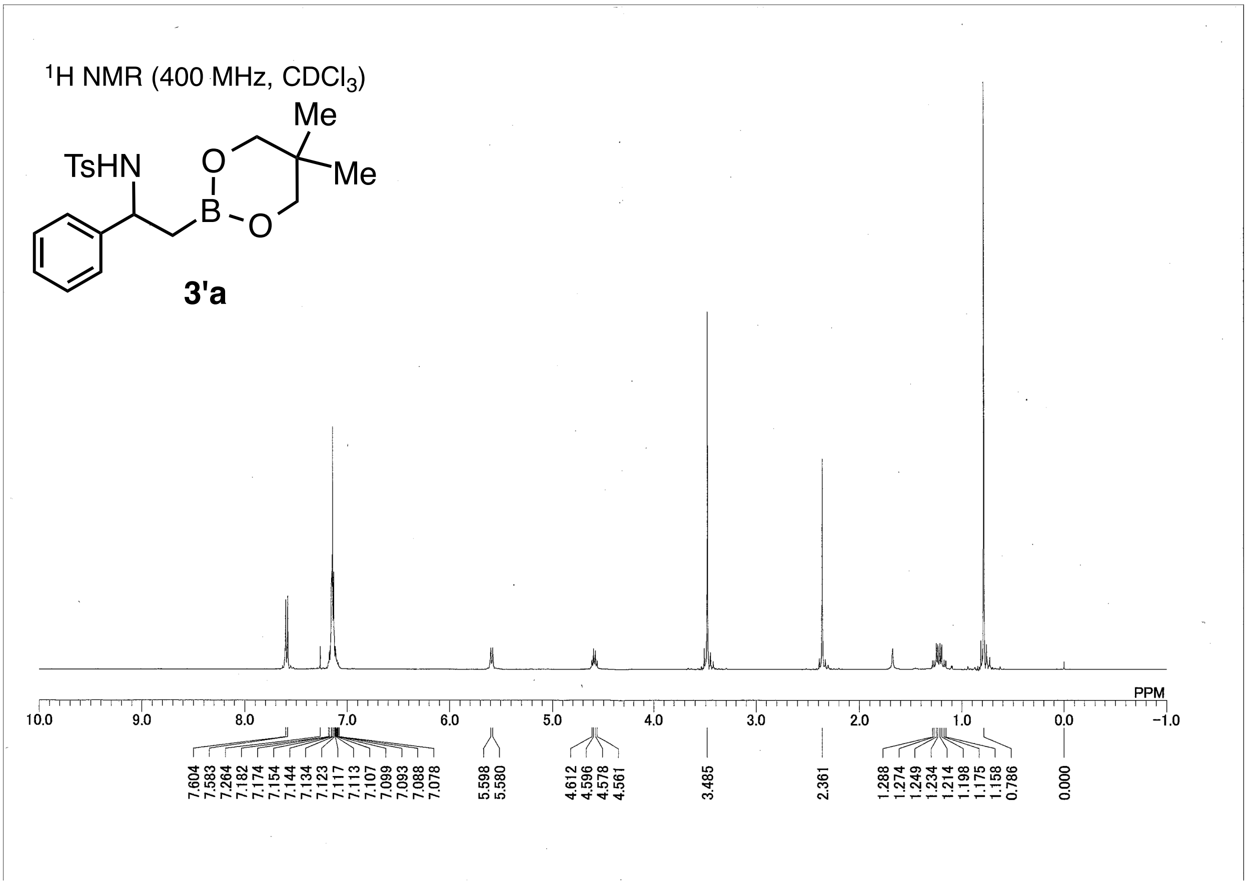
1H NMR (400 MHz, CDCl3)
TsHN
O
B
O
Me
Me
3'a
PPM
7.604 7.583 7.264 7.182 7.174 7.154 7.144 7.134 7.123 7.117 7.113 7.107 7.099 7.093 7.088 7.078
5.598 5.580
4.612 4.596 4.578 4.561
3.485
2.361
1.288 1.274 1.249 1.234 1.214 1.198 1.175 1.158 0.786
0.000

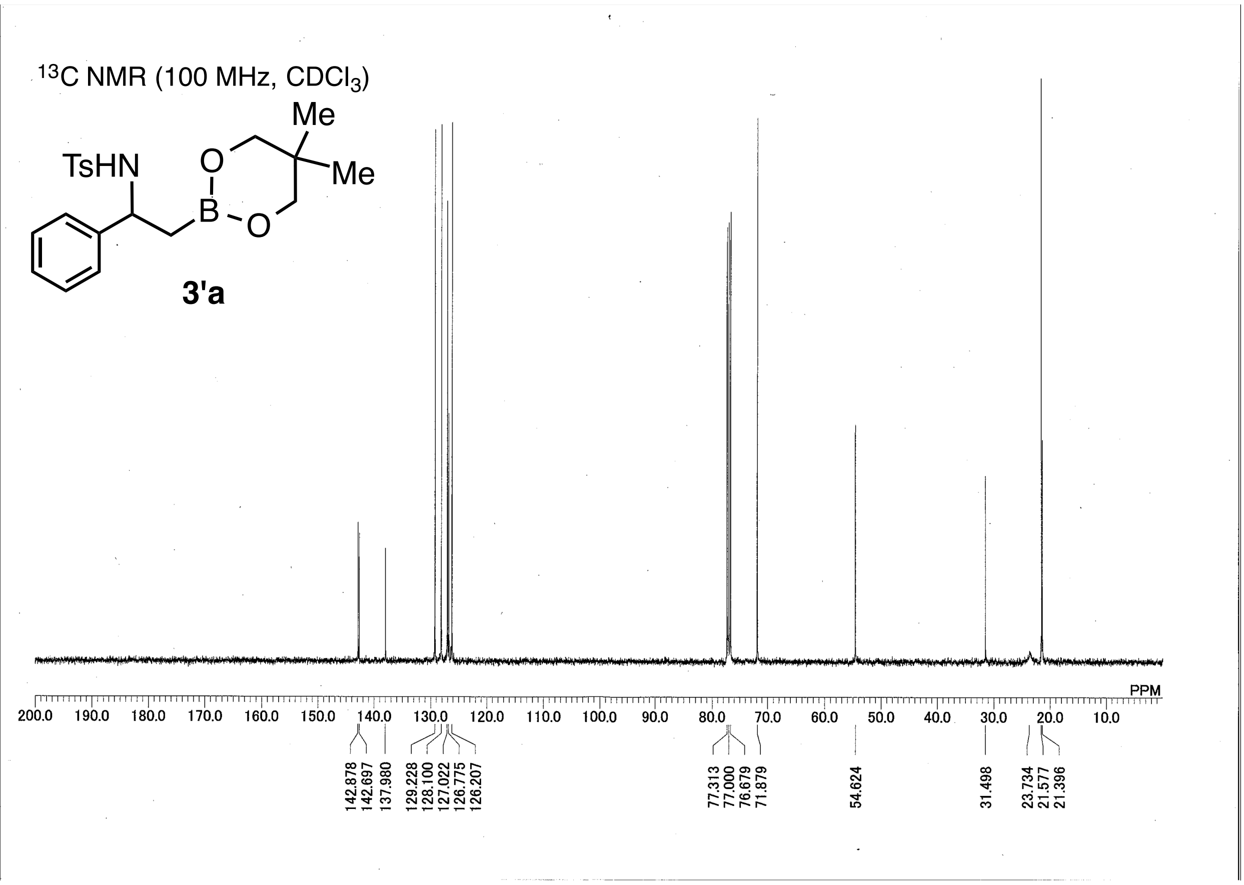
13C NMR (100 MHz, CDCl3)
TsHN
O
B
O
Me
Me
3'a
PPM
142.878 142.697 137.980
129.228 128.100 127.022 126.775 126.207
77.313 77.000 76.679 71.879
54.624
31.498
23.734 21.577 21.396

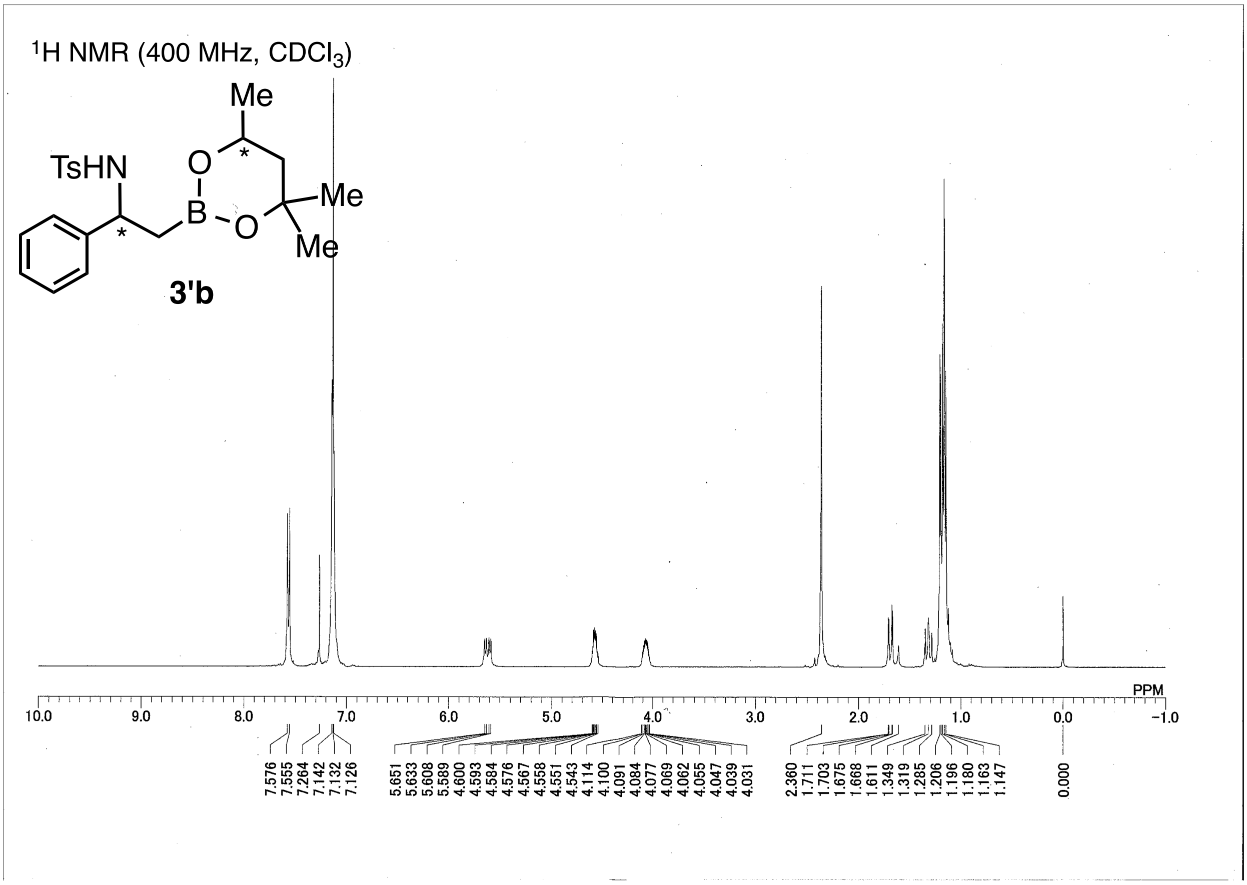
1H NMR (400 MHz, CDCl3)
TsHN
Me
Me
Me
3'b
PPM

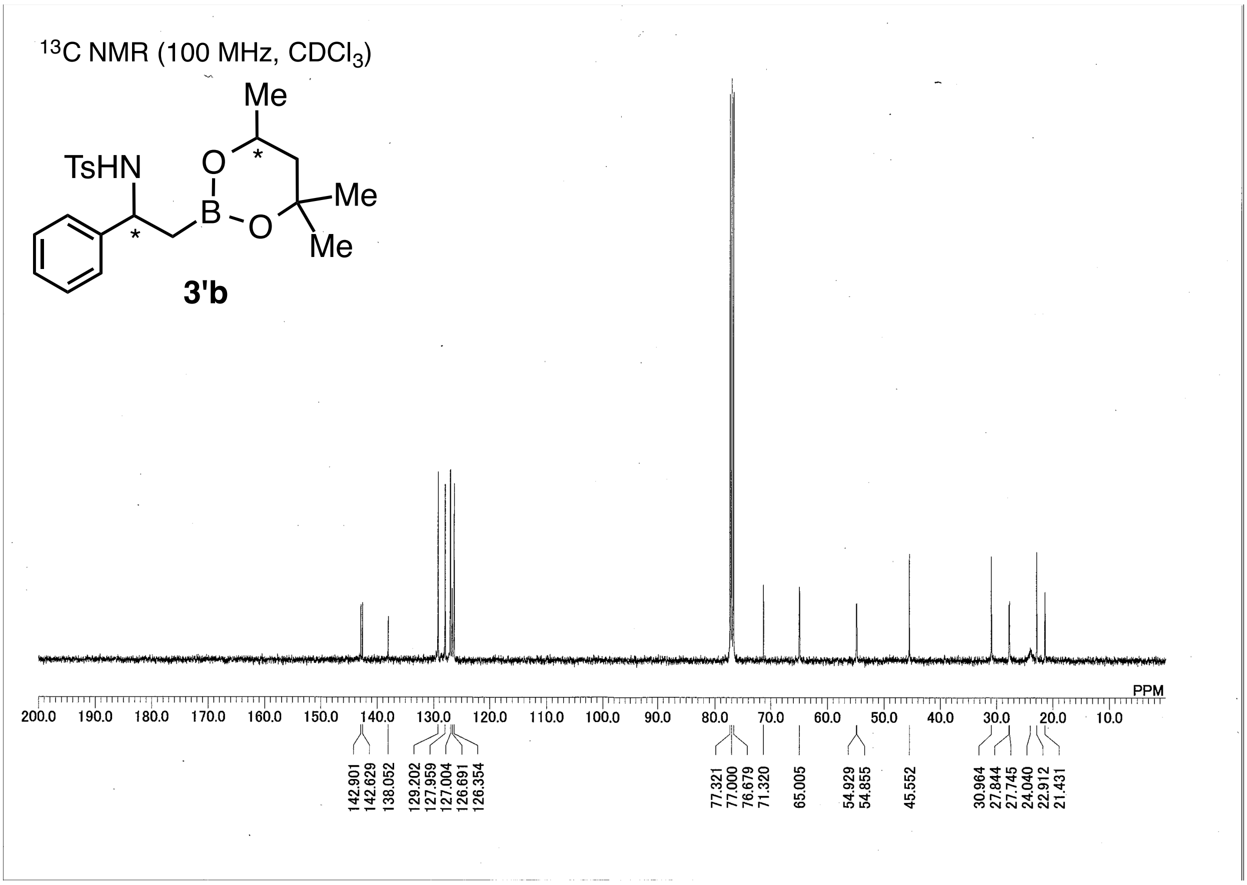
13C NMR (100 MHz, CDCl3)
TsHN
Me
Me
Me
3'b
PPM

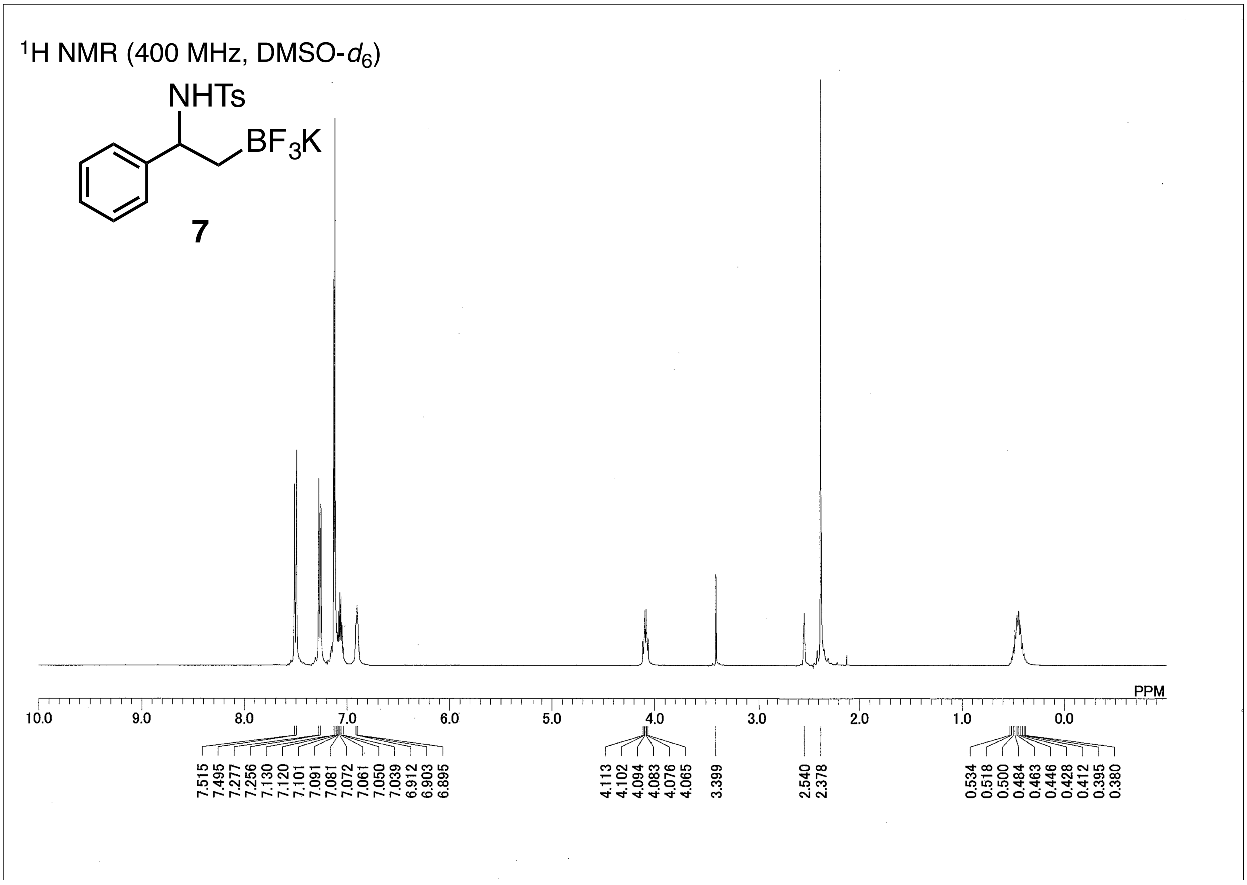
^{1}H NMR (400 MHz, DMSO-d_6)
NHTs
BF3K
7
PPM
7.515 7.495 7.277 7.256 7.130 7.120 7.101 7.091 7.081 7.072 7.061 7.050 7.039 6.912 6.903 6.895
4.113 4.102 4.094 4.083 4.076 4.065
3.399
2.540 2.378
0.534 0.518 0.500 0.484 0.463 0.446 0.428 0.412 0.395 0.380

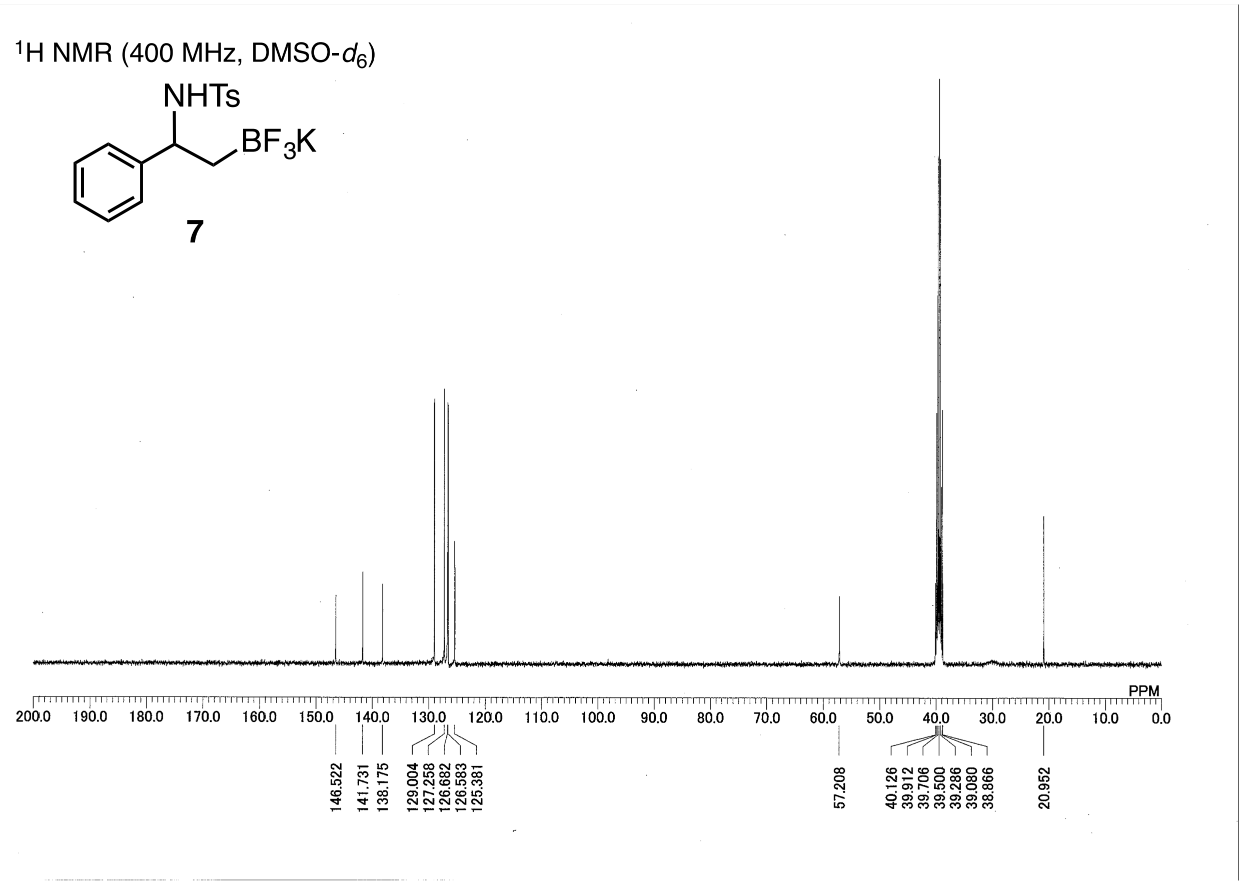
^{1}H NMR (400 MHz, DMSO-d_6)
NHTs
BF3K
7
PPM
146.522 141.731 138.175
129.004 127.258 126.682 126.583 125.381
57.208
40.126 39.912 39.706 39.500 39.286 39.080 38.866
20.952

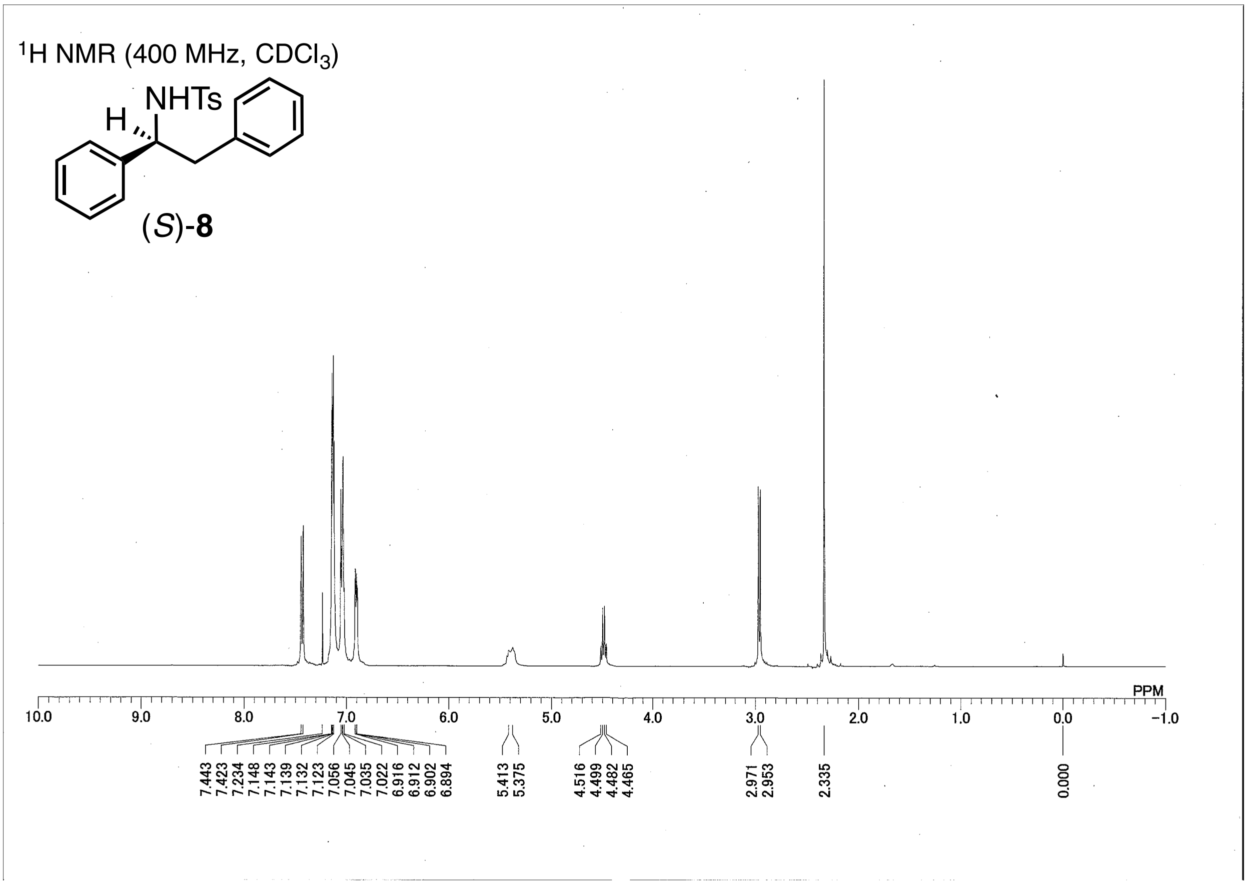

1H NMR (400 MHz, CDCl3)
NHTs
H
(S)-8
PPM
10.0 9.0 8.0 7.0 6.0 5.0 4.0 3.0 2.0 1.0 0.0 −1.0
7.443 7.423 7.234 7.148 7.143 7.139 7.132 7.123 7.056 7.045 7.035 7.022 6.916 6.912 6.902 6.894
5.413 5.375
4.516 4.499 4.482 4.465
2.971 2.953
2.335
0.000

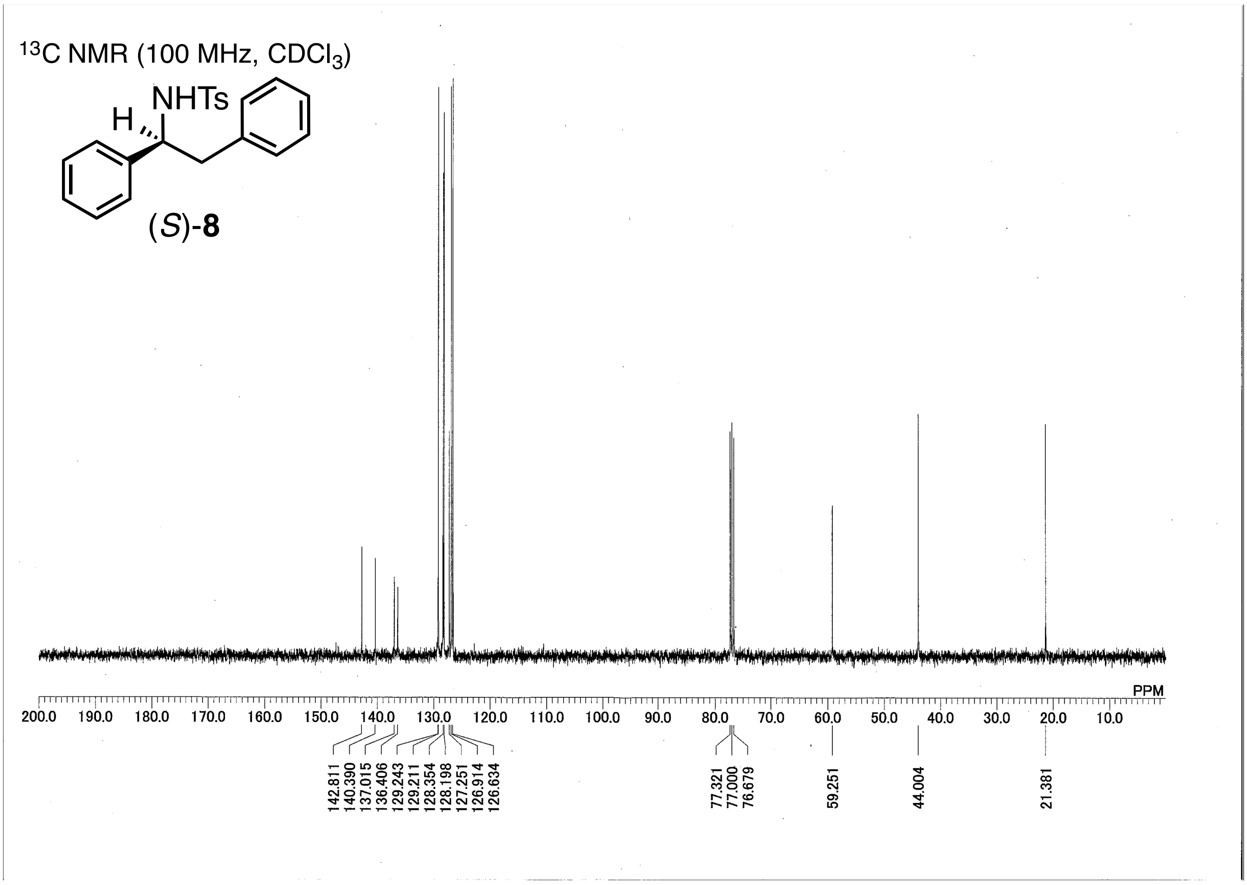

13C NMR (100 MHz, CDCl3)
NHTs
H
(S)-8
PPM
200.0 190.0 180.0 170.0 160.0 150.0 140.0 130.0 120.0 110.0 100.0 90.0 80.0 70.0 60.0 50.0 40.0 30.0 20.0 10.0
142.811 140.390 137.015 136.406 129.243 129.211 128.354 128.198 127.251 126.914 126.634
77.321 77.000 76.679
59.251
44.004
21.381

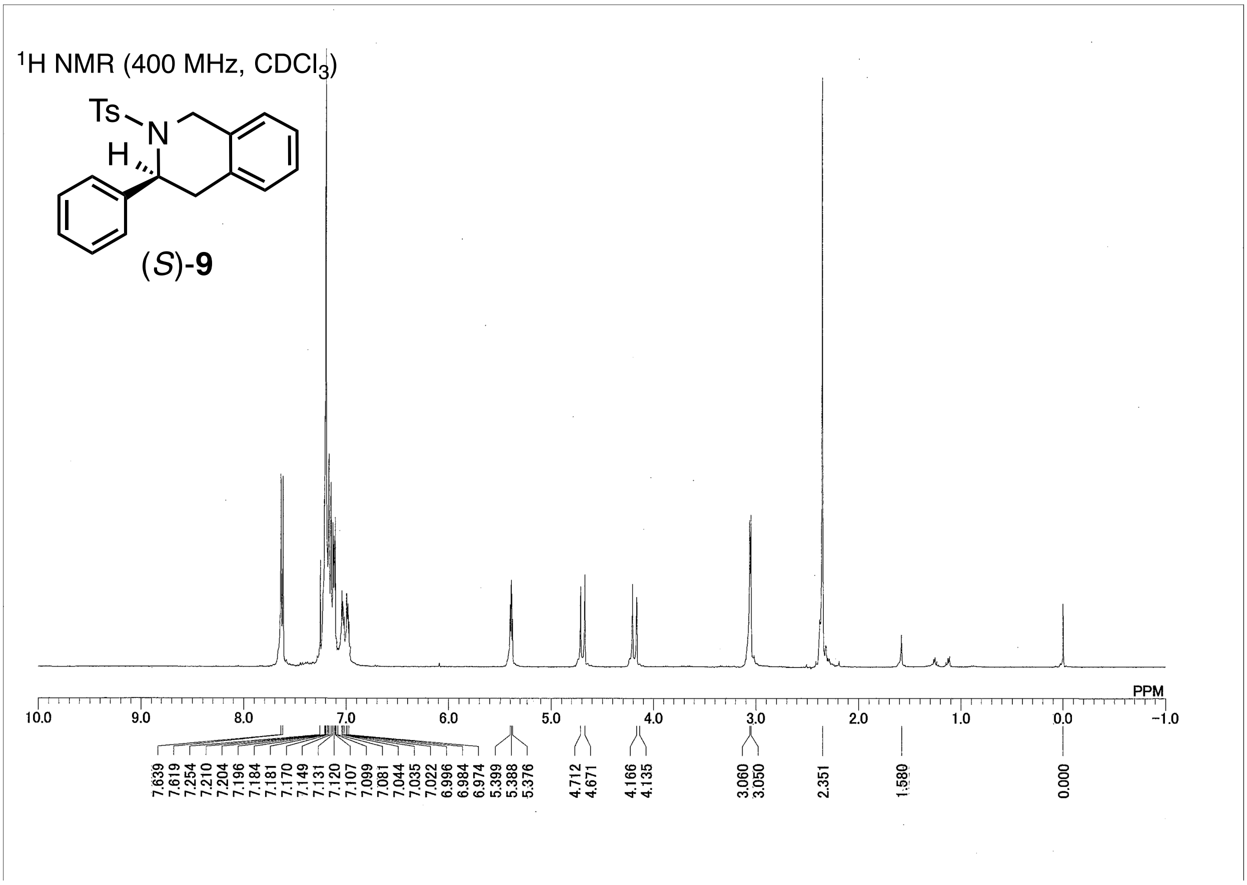

1H NMR (400 MHz, CDCl3)
Ts
N
H
(S)-9
PPM
10.0 9.0 8.0 7.0 6.0 5.0 4.0 3.0 2.0 1.0 0.0 −1.0
7.639 7.619 7.254 7.210 7.204 7.196 7.184 7.181 7.170 7.149 7.131 7.120 7.107 7.099 7.081 7.044 7.035 7.022 6.996 6.984 6.974 5.399 5.388 5.376 4.712 4.671 4.166 4.135 3.060 3.050 2.351 1.580 0.000

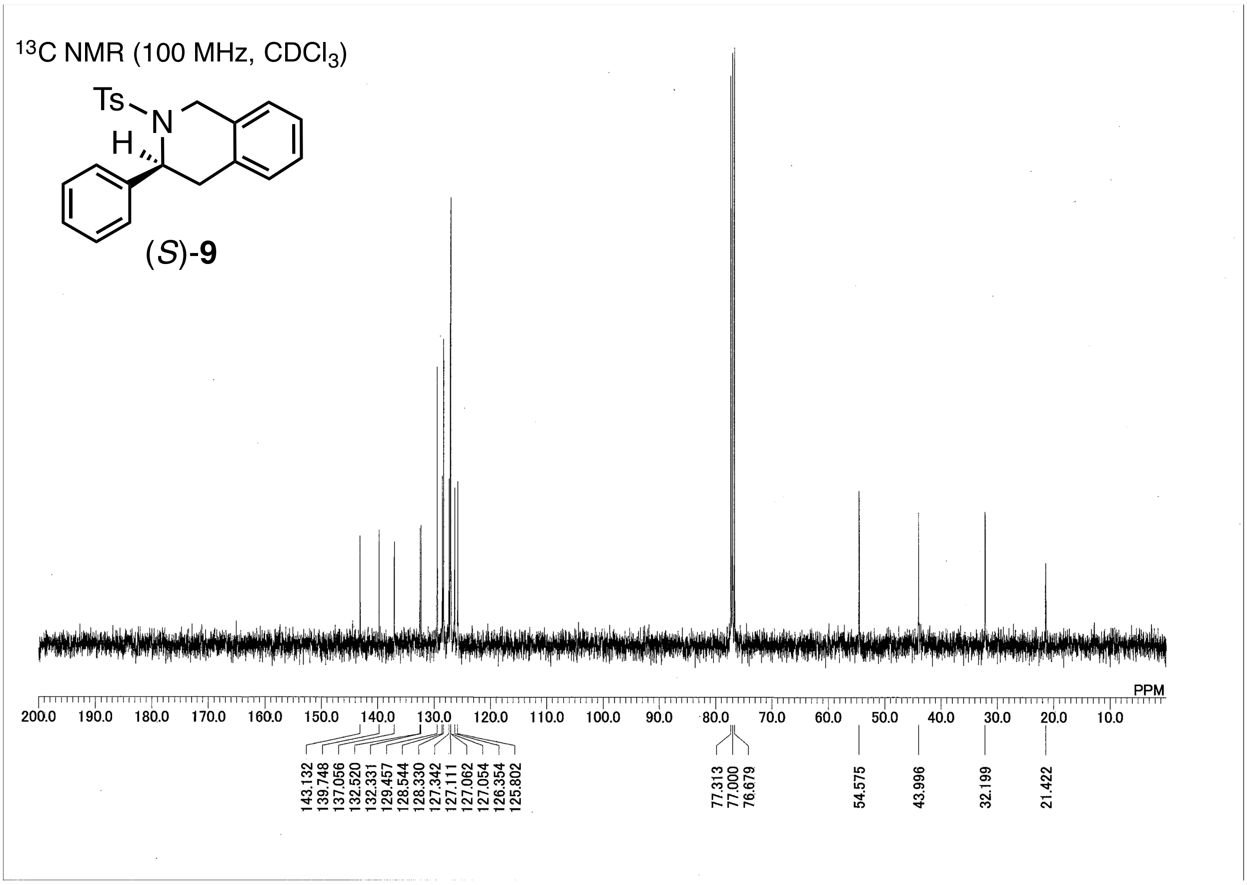

13C NMR (100 MHz, CDCl3)
Ts
N
H
(S)-9
PPM
200.0 190.0 180.0 170.0 160.0 150.0 140.0 130.0 120.0 110.0 100.0 90.0 80.0 70.0 60.0 50.0 40.0 30.0 20.0 10.0
143.132 139.748 137.056 132.520 132.331 129.457 128.544 128.330 127.342 127.111 127.062 127.054 126.354 125.802 77.313 77.000 76.679 54.575 43.996 32.199 21.422

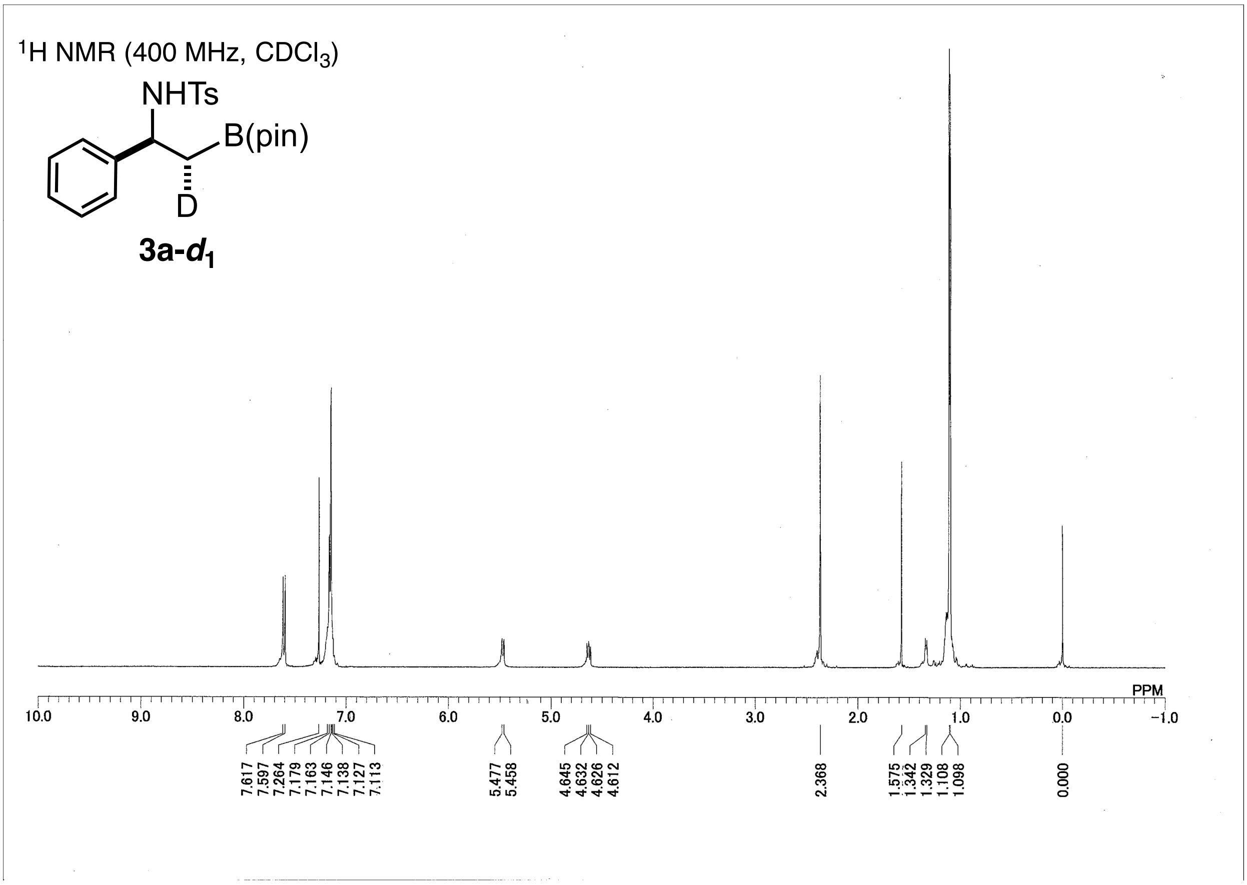
1H NMR (400 MHz, CDCl3)
NHTs
B(pin)
D
3a-d1
PPM
10.0 9.0 8.0 7.0 6.0 5.0 4.0 3.0 2.0 1.0 0.0 −1.0
7.617 7.597 7.264 7.179 7.163 7.146 7.138 7.127 7.113
5.477 5.458
4.645 4.632 4.626 4.612
2.368
1.575 1.342 1.329 1.108 1.098
0.000

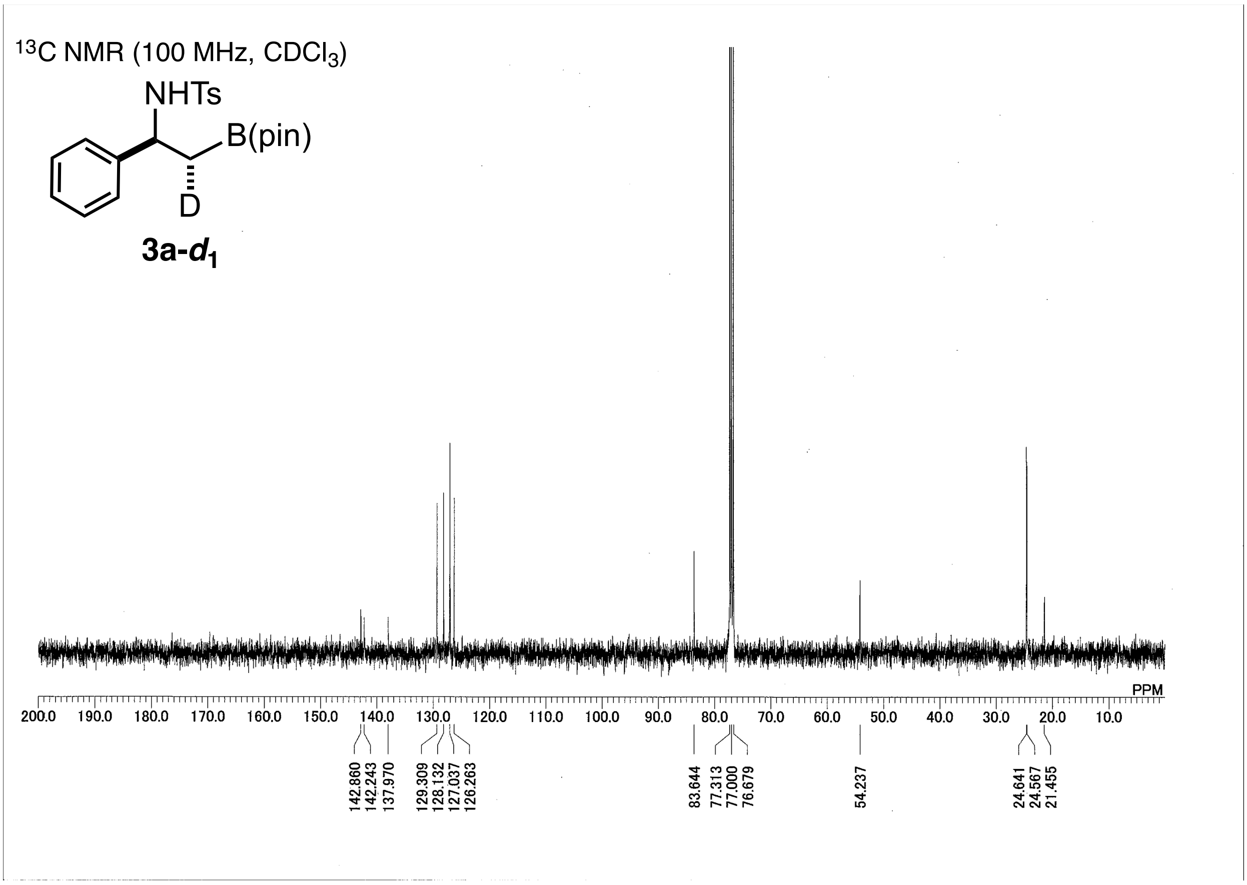
13C NMR (100 MHz, CDCl3)
NHTs
B(pin)
D
3a-d1
PPM
200.0 190.0 180.0 170.0 160.0 150.0 140.0 130.0 120.0 110.0 100.0 90.0 80.0 70.0 60.0 50.0 40.0 30.0 20.0 10.0
142.860 142.243 137.970
129.309 128.132 127.037 126.263
83.644
77.313 77.000 76.679
54.237
24.641 24.567 21.455

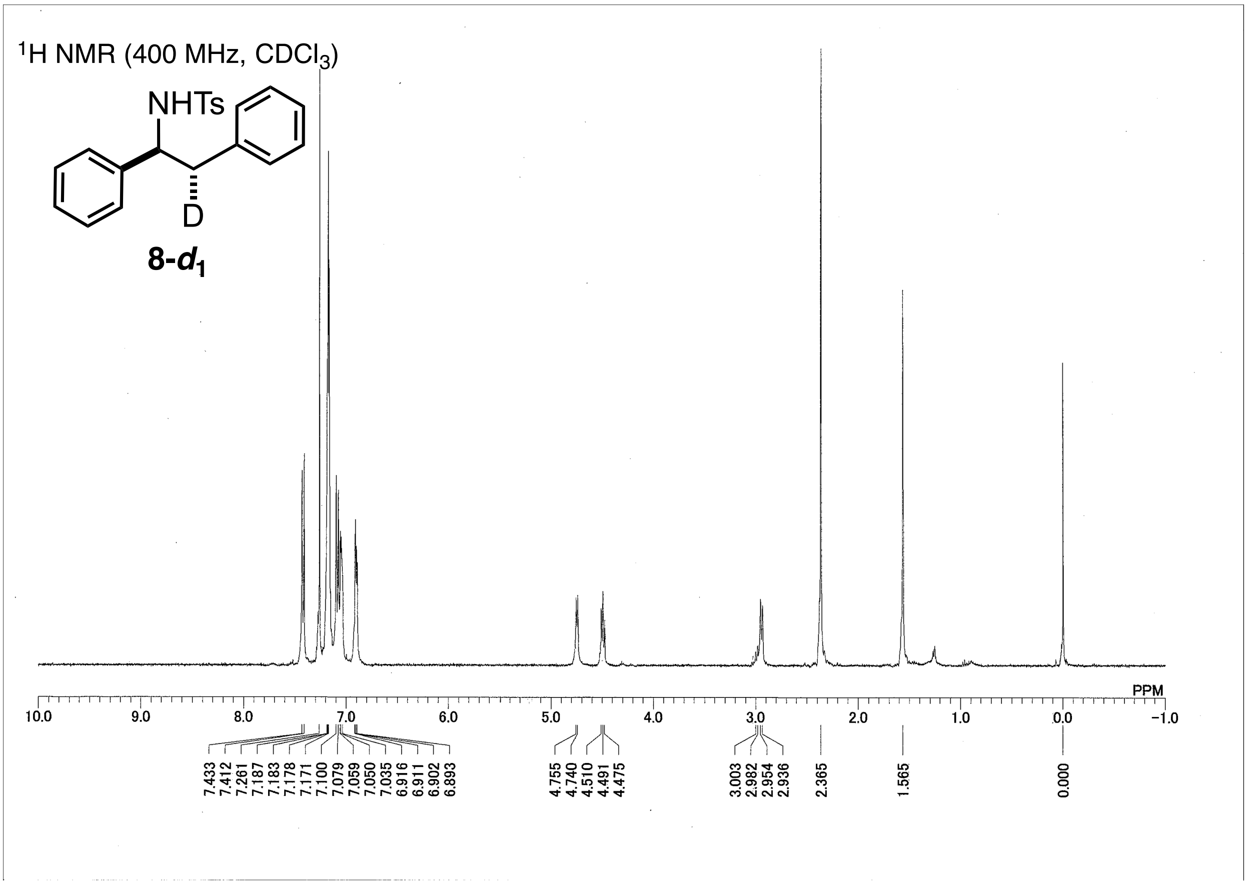
1H NMR (400 MHz, CDCl3)
NHTs
D
8-d1
PPM
10.0 9.0 8.0 7.0 6.0 5.0 4.0 3.0 2.0 1.0 0.0 −1.0
7.433 7.412 7.261 7.187 7.183 7.178 7.171 7.100 7.079 7.059 7.050 7.035 6.916 6.911 6.902 6.893
4.755 4.740 4.510 4.491 4.475
3.003 2.982 2.954 2.936
2.365
1.565
0.000

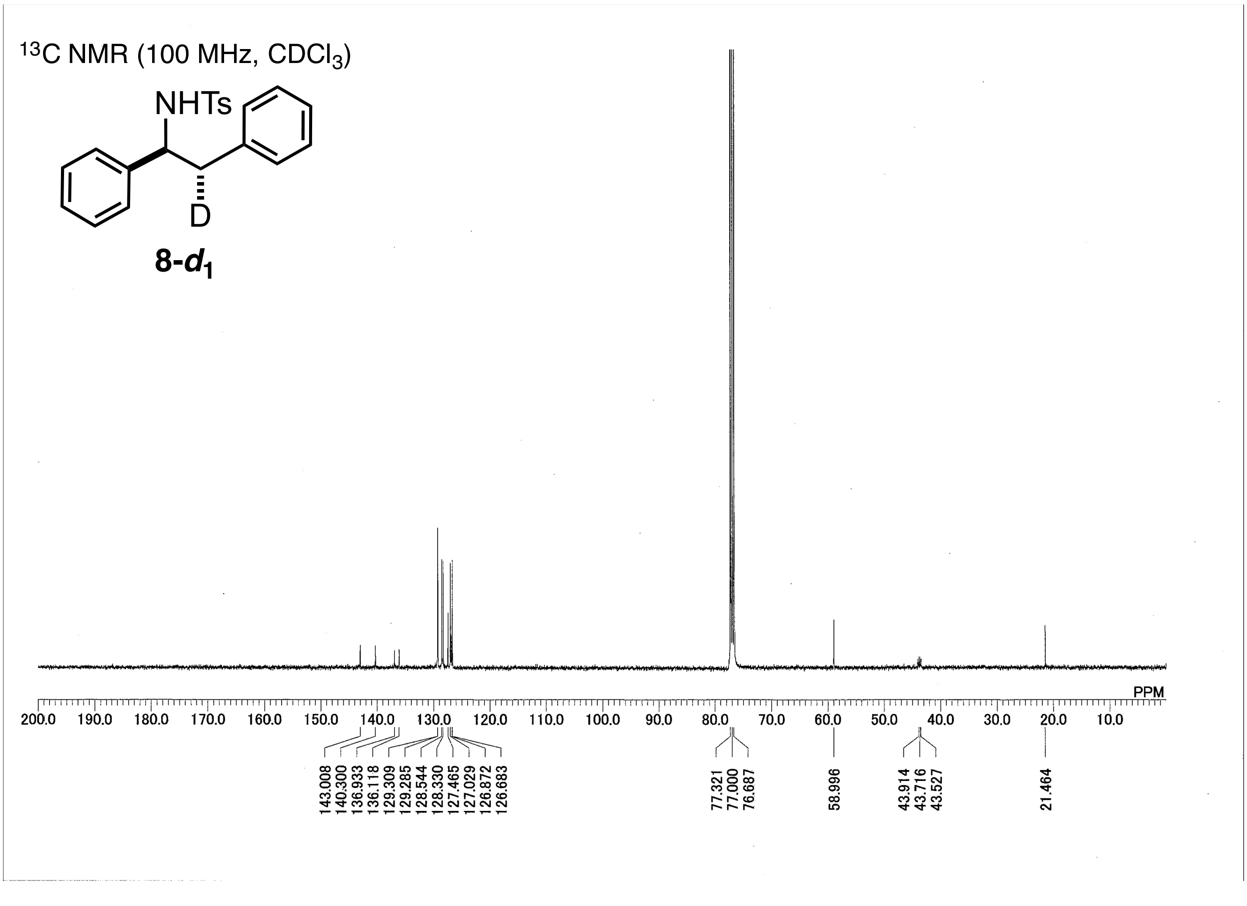
13C NMR (100 MHz, CDCl3)
NHTs
D
8-d1
PPM
200.0 190.0 180.0 170.0 160.0 150.0 140.0 130.0 120.0 110.0 100.0 90.0 80.0 70.0 60.0 50.0 40.0 30.0 20.0 10.0
143.008 140.300 136.933 136.118 129.309 129.285 128.544 128.330 127.465 127.029 126.872 126.683
77.321 77.000 76.687
58.996
43.914 43.716 43.527
21.464

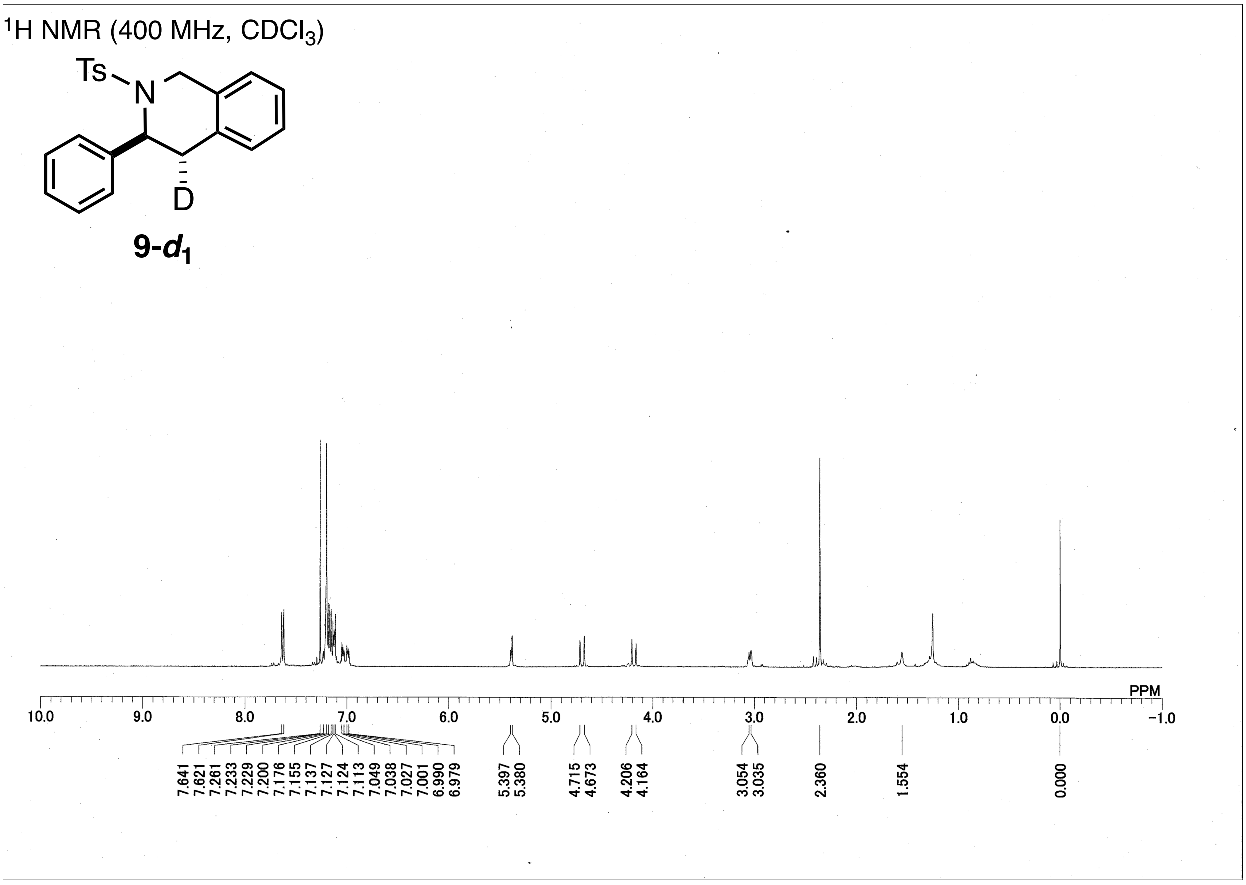

1H NMR (400 MHz, CDCl3)
Ts
N
D
9-d1
PPM
7.641 7.621 7.261 7.233 7.229 7.200 7.176 7.155 7.137 7.127 7.124 7.113 7.049 7.038 7.027 7.001 6.990 6.979
5.397 5.380
4.715 4.673
4.206 4.164
3.054 3.035
2.360
1.554
0.000

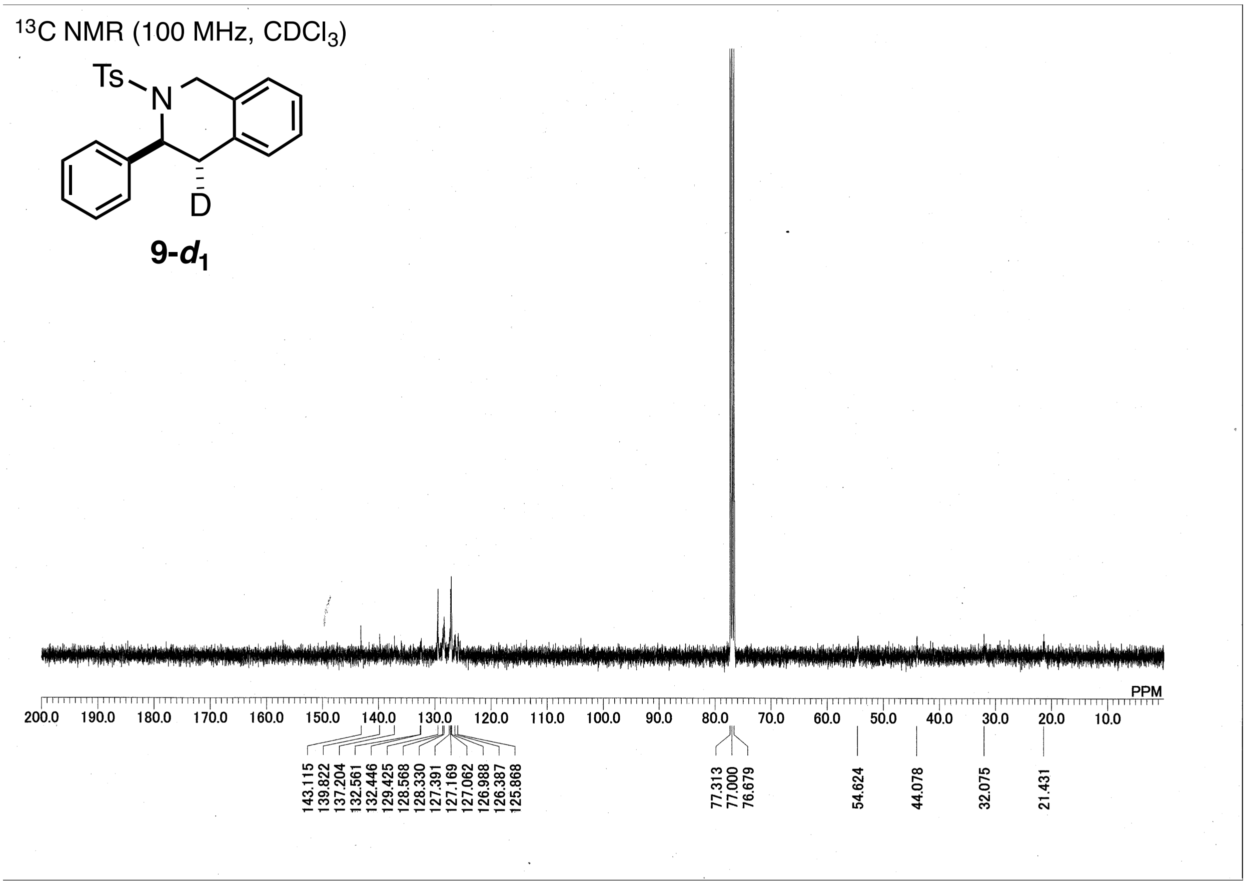

13C NMR (100 MHz, CDCl3)
Ts
N
D
9-d1
PPM
143.115 139.822 137.204 132.561 132.446 129.425 128.568 128.330 127.391 127.169 127.062 126.988 126.387 125.868
77.313 77.000 76.679
54.624
44.078
32.075
21.431

HPLC charts

(Chiralcel OJ; 1.0 mL/min; *i*-PrOH/*n*-hexane 30:70; λ = 254 nm)

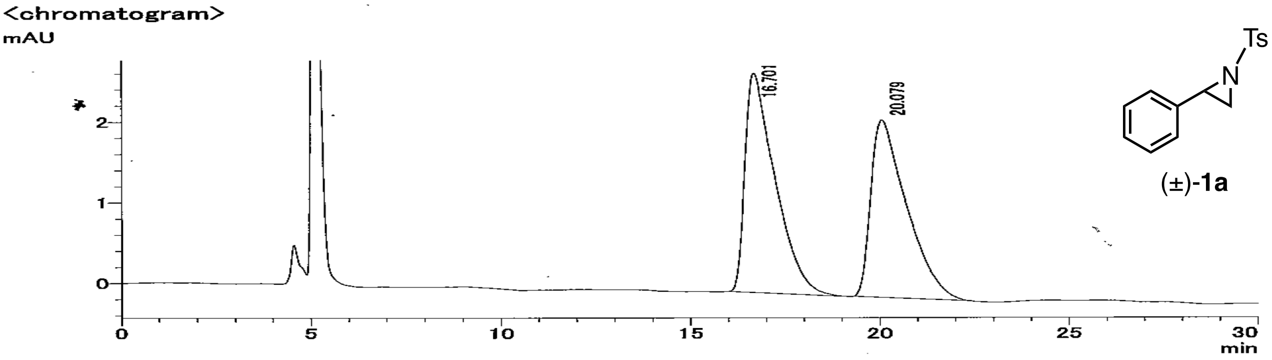

<peak report>

peak#	retention time	area	height	area%
1	16.701	150769	2718	51.037
2	20.079	144645	2203	48.963

(Chiralcel OJ; 1.0 mL/min; *i*-PrOH/*n*-hexane 30:70; λ = 254 nm)

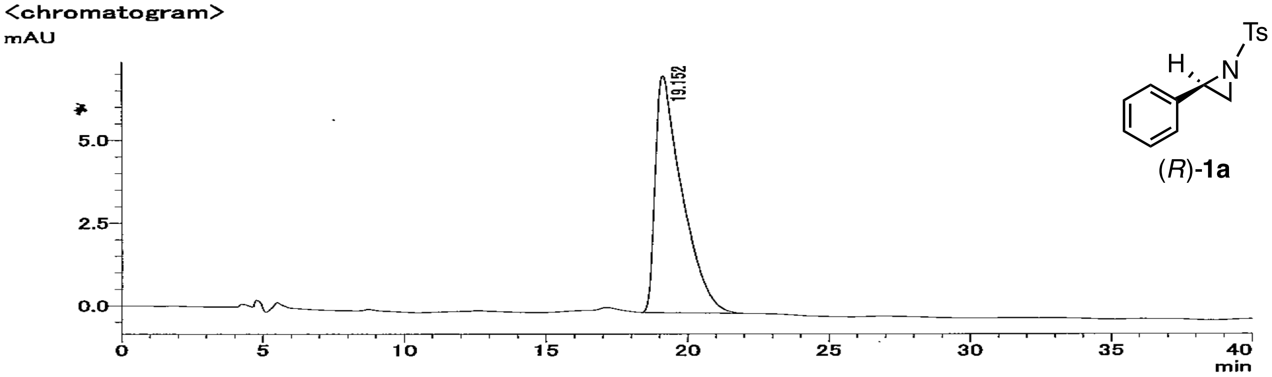

<peak report>

peak#	retention time	area	height	area%
1	19.152	472281	7161	100.000

(Chiralcel OJ; 1.0 mL/min; *i*-PrOH/*n*-hexane 30:70; λ = 254 nm)

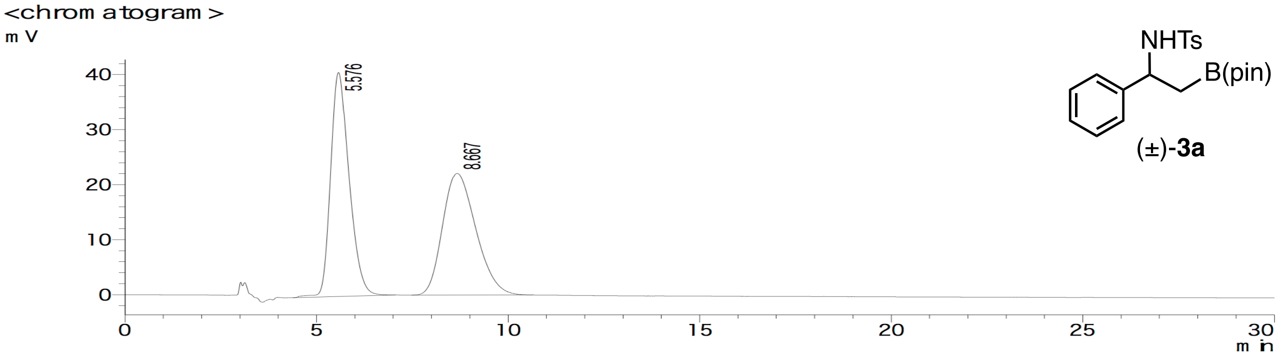

<peak report>

peak#	retention time	area	height	area%
1	5.576	1367969	40724	50.588
2	8.667	1336183	22106	49.412

(Chiralcel OJ; 1.0 mL/min; *i*-PrOH/*n*-hexane 20:80; λ = 254 nm)

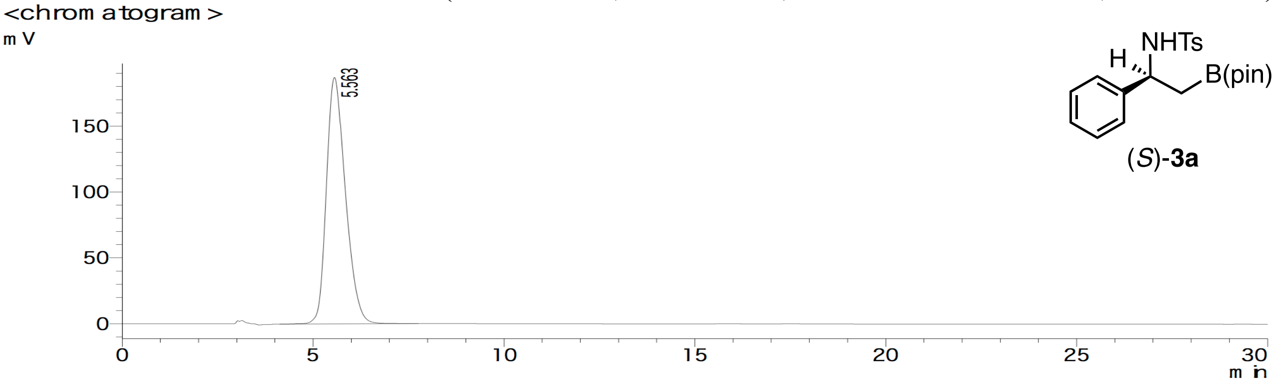

<peak report>

peak#	retention time	area	height	area%
1	5.563	6483014	187078	100.000

(Chiralcel OJ, 1.0 mL/min; *i*-PrOH/*n*-hexane 20:80; λ = 254 nm)

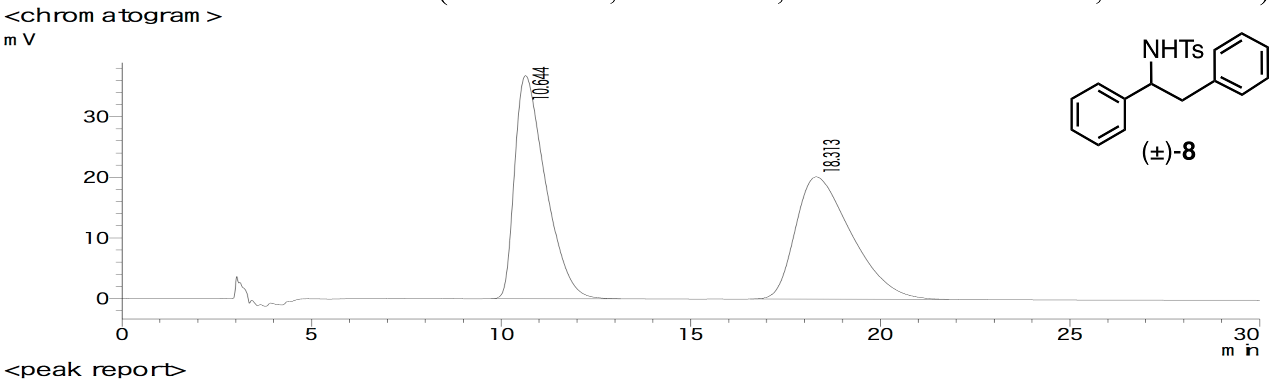

<peak report>

peak#	retention time	area	height	area%
1	10.644	2089056	36812	50.168
2	18.313	2075052	20184	49.832

(Chiralcel OJ, 1.0 mL/min; *i*-PrOH/*n*-hexane 20:80; λ = 254 nm)

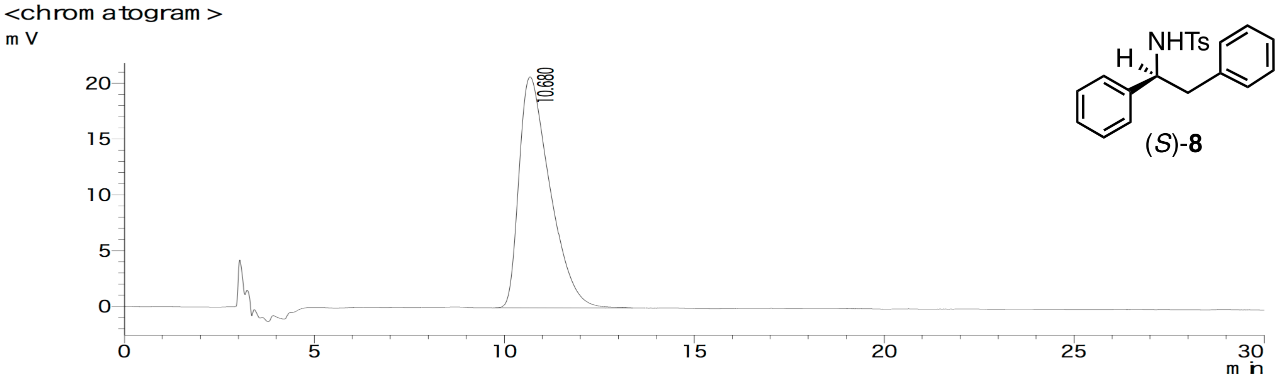

<peak report>

peak#	retention time	area	height	area%
1	10.680	1178040	20713	100.000

(Chiralcel OD-H, 1.0 mL/min; *i*-PrOH/*n*-hexane 20:80; λ = 254 nm)

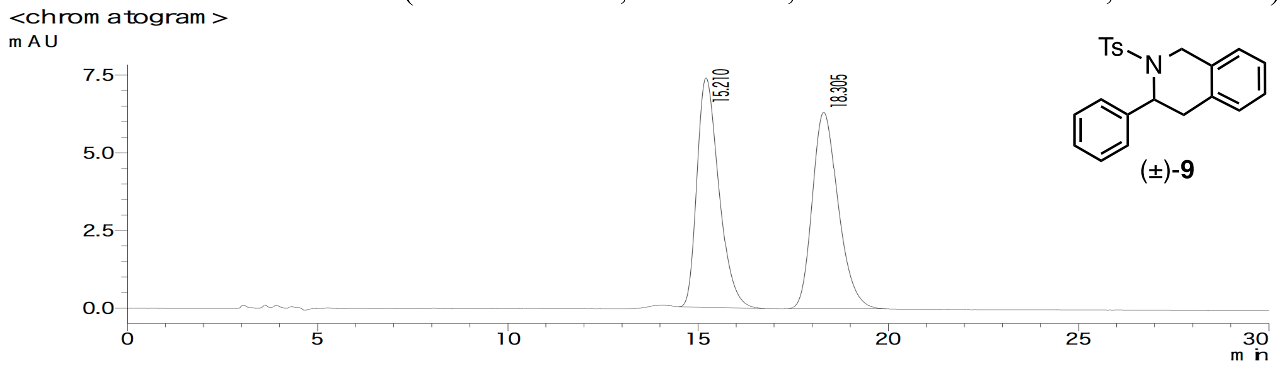

<peak report>

peak#	retention time	area	height	area%
1	15.210	296587	7387	49.737
2	18.305	299719	6325	50.263

(Chiralcel OD-H, 1.0 mL/min; *i*-PrOH/*n*-hexane 20:80; λ = 254 nm)

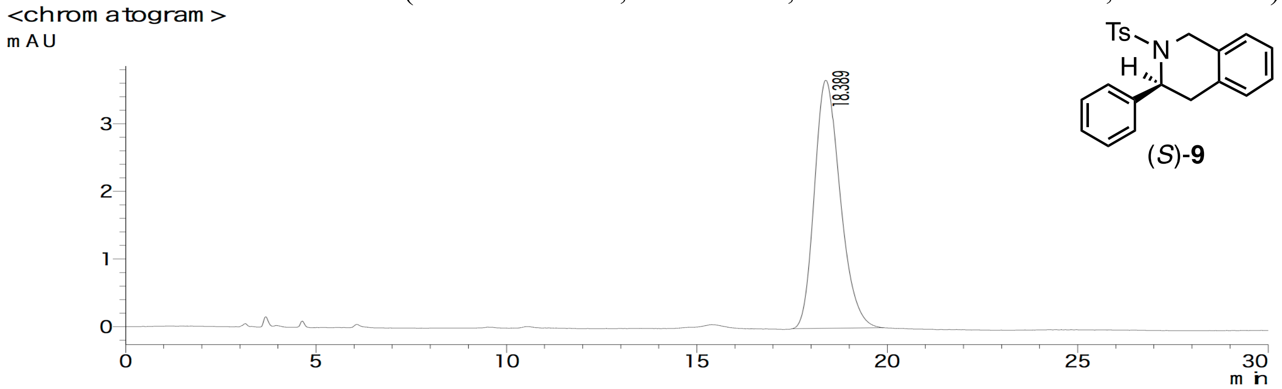

<peak report>

peak#	retention time	area	height	area%
1	18.389	172802	3672	100.000

References for the Experimental Part

S1. Tatsuno, Y.; Yoshida, T.; Otsuka, S. *Inorg. Synth.* **1979**, *19*, 220.

S2. Fraser, A. W.; Bewas, J. E.; Hull, L. E.; Baird, M. C. *Organometallics* **2012**, *31*, 2470.

S3. (a) Kano, D.; Minakata, S.; Komatsu, M. *J. Chem. Soc., Perkin Trans. 1* **2001**, 3186. (b) Jin, W.; Li, X.; Huang, Y.; Wu, F.; Wan, B. *Chem.—Eur. J.* **2010**, *16*, 8259. (c) Aggawal, V. K.; Ferrara, M. *Org. Lett.* **2000**, *2*, 4107. (d) Dauban, P.; Saniére, L.; Tarrade, A.; Dodd, R. H. *J. Am. Chem. Soc.* **2001**, *123*, 7707. (e) Craig II, R. A.; O'Connor, N. R.; Goldberg, A. F.; Stoltz, B. M. *Chem.—Eur. J.* **2014**, *20*, 4806. (f) Seayad, J.; Seayad, A. M.; Ng, J. K. P.; Chai, C. L. L. *ChemCatChem* **2012**, *4*, 774. (g) Ando, T.; Kano, D.; Minakata, S.; Ryu, I.; Komatsu, M. *Tetrahedron* **1998**, *54*, 13485. (h) Jensen, K. L.; Standley, E. A.; Jamison, T. F. *J. Am. Chem. Soc.* **2014**, *136*, 11145. (i) Ney, J. E.; Wolfe, J. P. *J. Am. Chem. Soc.* **2006**, *128*, 15415. (j) Chang, J. W. W.; Ton, T. M. U.; Zhang, Z.; Xu, Y.; Chan, P. W. H. *Tetrahedron Lett.* **2009**, *50*, 161. (k) Li, Z.; Ding, X.; He, C. *J. Org. Chem.* **2006**, *71*, 5876. (l) Han, H.; Park, S. B.; Kim, S. K.; Chang, S. *J. Org. Chem.* **2008**, *73*, 2862.

S4. (a) Evans, D. A.; Bilodeau, M. T.; Faul, M. M. *J. Am. Chem. Soc.* **1994**, *116*, 2742. (b) Gao, G.-Y.; Harden, J. D.; Zhang, X. P. *Org. Lett.* **2005**, *7*, 3191. (c) Huang, C.-Y.; Doyle, A. G. *J. Am. Chem. Soc.* **2012**, *134*, 9541. (d) Saikia, I.; Kashyap, B.; Phukan, P. *Chem. Commun.* **2011**, *47*, 2967. (e) Ruppel, J. V.; Jones, J. E.; Huff, C. A.; Kamble, R. M.; Chen, Y.; Zhang, X. P. *Org. Lett.* **2008**, *10*, 1995.

S5. Gao, M.; Thorpe, S. B.; Santos, W. L. *Org. Lett.* **2009**, *11*, 3478.

S6. Zhang, L.; Zuo, Z.; Leng, X.; Huang, Z. *Angew. Chem. Int. Ed.* **2014**, *53*, 2696.

S7. Mei, H.; Ji, X.; Han, J.; Pan, Y. *Eur. J. Org. Chem.* **2011**, 5783.

S8. Berger, M. L.; Schweifer, A.; Rebernik, P.; Hammerschmidt, F. *Bioorg. Med. Chem.* **2009**, *17*, 3456–3462.

S9. Lukanov, L. K.; Venkov, A. P.; Mollov, N. M. *Synthesis* **1987**, 204.

S10. Ueno, S.; Ohtsubo, M.; Kuwano, R. *J. Am. Chem. Soc.* **2009**, *131*, 12904.

S11. Coote, S. J.; Davies, S. G.; Fletcher, A. M.; Roberts, P. M.; Thomson, J. E. *Chem.—Asian J.* **2010**, *5*, 589.

S12. Mitchell, E. A.; Baird, M. C. *Organometallics* **2007**, *26*, 5230.

Computational Part

TSs for the Aziridine Ring Opening Starting from I and Iw

Table S14. TSs for the aziridine ring opening starting from **I** and **Iw**. ΔG and ΔE values are in kcal mol^{-1}.

TS	Group	ΔΔG	ΔΔE
TS1	TSI-II$_K$	0.0	0.0
TS2	TSI-II$_K$	0.3	-1.6
TS3	TSI-II$_L$	0.6	2.2
TS4	TSI-II$_A$	0.7	-1.1
TS5	TSI-II$_K$	0.8	-0.1
TS6	TSI-II$_B$	0.9	0.7
TS7	TSI-II$_B$	1.0	0.7
TS8	TSI-II$_A$	1.1	-0.2
TS9	TSI-II$_K$	1.3	0.7
TS10	TSI-II$_K$	2.3	0.6
TS11	TSI-II$_K$	2.4	0.6
TS12	TSI-II$_D$	2.6	-8.2
TS13	TSI-II$_K$	2.6	1.3
TS14	TSI-II$_K$	2.7	0.5
TS15	TSI-II$_K$	3.3	1.3
TS16	TSI-II$_K$	3.5	0.9
TS17	TSI-II$_K$	3.6	2.2
TS18	TSI-II$_B$	3.7	-7.8
TS19	TSI-II$_A$	3.7	-6.6
TS20	TSI-II$_B$	3.9	-5.4
TS21	TSI-II$_L$	4.1	2.6
TS22	TSI-II$_K$	4.5	1.6
TS23	TSI-II$_A$	4.5	-5.7
TS24	TSI-II$_B$	4.7	-6.9
TS25	TSI-II$_B$	4.8	-7.5
TS26	TSI-II$_B$	5.0	-6.4
TS27	TSI-II$_D$	5.2	-6.8
TS28	TSI-II$_A$	5.7	-5.6
TS29	TSI-II$_B$	5.8	-4.5
TS30	TSI-II$_A$	5.9	-6.2
TS31	TSI-II$_B$	6.1	-5.5
TS32	TSI-II$_M$	6.1	-7.5
TS33	TSI-II$_A$	6.3	-4.4
TS34	TSI-II$_A$	6.3	-5.9
TS35	TSI-II$_B$	6.6	-6.1
TS36	TSI-II$_B$	6.7	-3.9
TS37	TSI-II$_A$	7.1	-5.0
TS38	TSI-II$_A$	7.2	-4.9
TS39	TSI-II$_B$	7.2	-5.3
TS40	TSI-II$_B$	7.2	-2.8
TS41	TSI-II$_C$	7.4	-5.7

TS42	$TSI\text{-}II_B$	7.5	-2.8
TS43	$TSI\text{-}II_A$	7.7	-4.6
TS44	$TSI\text{-}II_B$	7.9	-4.8
TS45	$TSI\text{-}II_B$	8.1	-4.0
TS46	$TSI\text{-}II_B$	8.4	-3.9
TS47	$TSI\text{-}II_B$	8.7	-3.6
TS48	$TSI\text{-}II_A$	8.7	-2.8
TS49	$TSI\text{-}II_A$	9.2	-4.2
TS50	$TSI\text{-}II_B$	9.4	-3.0
TS51	$TSI\text{-}II_A$	9.4	-3.7
TS52	$TSI\text{-}II_A$	9.6	-3.6
TS53	$TSI\text{-}II_A$	9.7	-1.4
TS54	$TSI\text{-}II_B$	10.1	-2.6
TS55	$TSI\text{-}II_A$	10.6	-1.0
TS56	$TSI\text{-}II_B$	11.2	-0.1
TS57	$TSI\text{-}II_M$	12.8	12.1
TS58	$TSI\text{-}II_M$	13.2	11.8
TS59	$TSI\text{-}II_M$	13.5	12.0
TS60	$TSI\text{-}II_M$	14.0	12.4
TS61	$TSI\text{-}II_M$	14.2	12.2
TS62	$TSI\text{-}II_M$	14.2	12.4
TS63	$TSI\text{-}II_M$	14.3	12.9
TS64	$TSI\text{-}II_G$	14.7	1.1
TS65	$TSI\text{-}II_O$	15.5	13.2
TS66	$TSI\text{-}II_N$	15.7	12.3
TS67	$TSI\text{-}II_M$	16.2	13.2
TS68	$TSI\text{-}II_M$	16.4	12.5
TS69	$TSI\text{-}II_A$	17.5	4.5
TS70	$TSI\text{-}II_F$	18.0	6.5
TS71	$TSI\text{-}II_A$	18.0	5.1
TS72	$TSI\text{-}II_F$	18.3	6.4
TS73	$TSI\text{-}II_O$	18.4	15.8
TS74	$TSI\text{-}II_E$	18.5	8.4
TS75	$TSI\text{-}II_F$	18.7	7.9
TS76	$TSI\text{-}II_O$	18.8	16.2
TS77	$TSI\text{-}II_E$	18.9	8.4
TS78	$TSI\text{-}II_O$	19.1	16.3
TS79	$TSI\text{-}II_J$	19.2	6.8
TS80	$TSI\text{-}II_H$	19.3	7.4
TS81	$TSI\text{-}II_O$	19.6	14.9
TS82	$TSI\text{-}II_O$	20.3	15.1
TS83	$TSI\text{-}II_O$	20.5	17.3
TS84	$TSI\text{-}II_E$	20.7	8.3
TS85	$TSI\text{-}II_F$	20.9	7.5
TS86	$TSI\text{-}II_O$	21.7	16.2
TS87	$TSI\text{-}II_J$	21.9	9.7
TS88	$TSI\text{-}II_J$	22.1	7.0
TS89	$TSI\text{-}II_E$	22.1	8.3

TS90	TSI-II$_I$	22.5	10.1
TS91	TSI-II$_E$	22.8	10.6
TS92	TSI-II$_J$	23.0	7.3
TS93	TSI-II$_I$	23.1	9.1
TS94	TSI-II$_F$	23.1	10.2
TS95	TSI-II$_I$	23.3	9.2
TS96	TSI-II$_J$	23.4	9.0
TS97	TSI-II$_J$	23.9	9.5
TS98	TSI-II$_J$	24.0	9.8
TS99	TSI-II$_F$	24.2	10.6
TS100	TSI-II$_I$	24.3	10.0
TS101	TSI-II$_F$	24.5	12.1
TS102	TSI-II$_F$	24.8	11.0
TS103	TSI-II$_E$	24.9	12.4
TS104	TSI-II$_I$	24.9	9.8
TS105	TSI-II$_E$	25.0	12.7
TS106	TSI-II$_I$	25.9	10.3

Relaxed Potential Energy Surfaces for the Protonation of Intermediate IIIw and IIIw$_2$

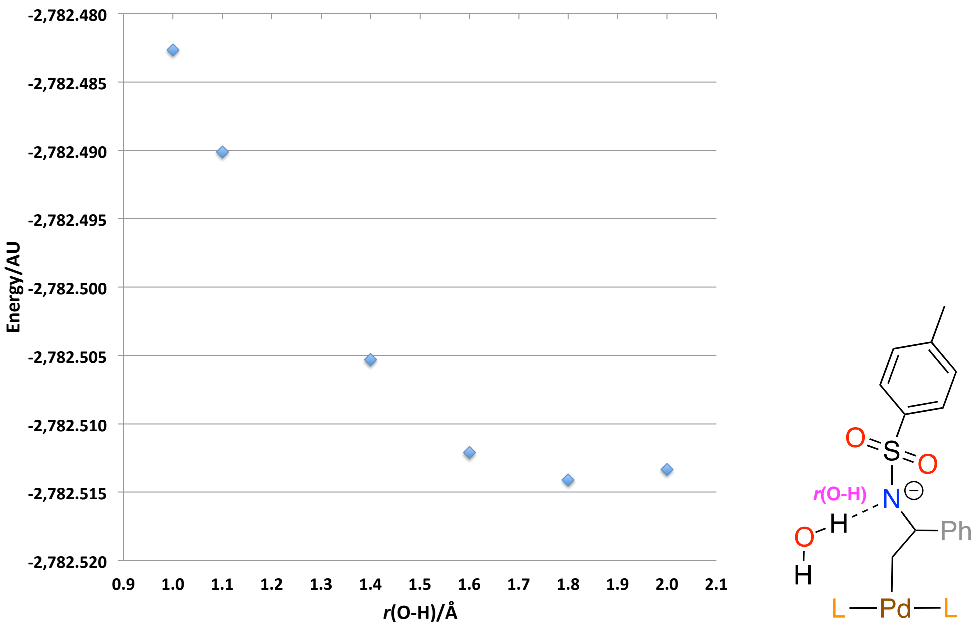

Figure S2. Relaxed potential energy scan for the proton transfer in **IIIw**.

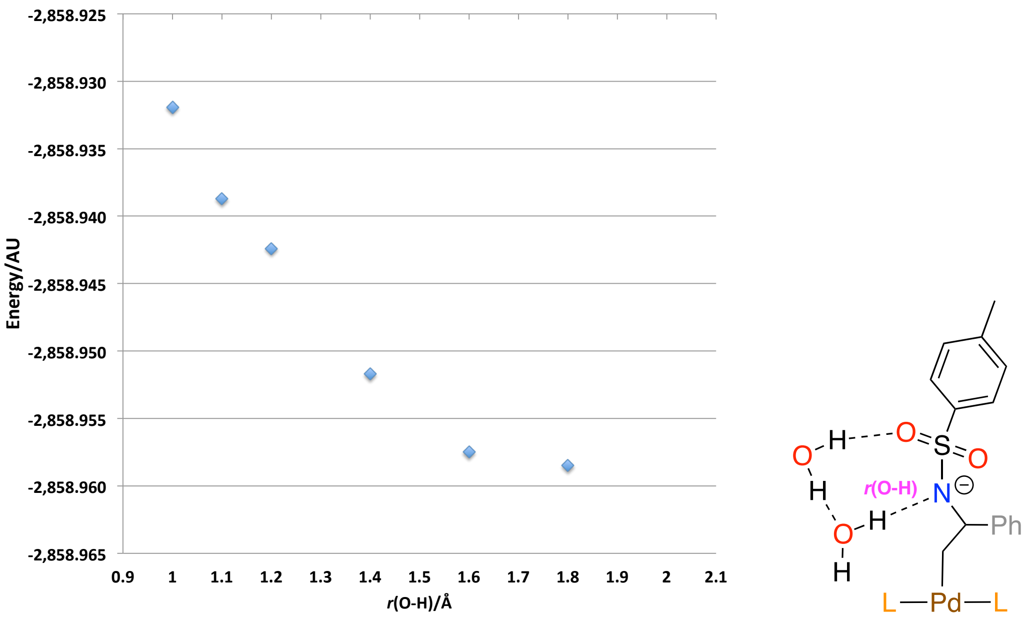

Figure S3. Relaxed potential energy scan for the proton transfer in **IIIw$_2$**.

Side reaction

Side reaction can start from the intermediate **III** (Figure 6), where the hydrogen atom in the β-position of the substrate can be transferred to the metal with a barrier of 0.8 kcal mol^{-1} (**TSIII-XI**). Starting from the resulting intermediate **XI** (0.4 kcal mol^{-1}), the hydrogen atom transfers from the metal to the α-carbon atom through **TSXI-XII** (5.1 kcal mol^{-1}), giving rise to an intermediate **XII** (–17.0 kcal mol^{-1}). In **XII**, side product **6** is directed away from the metal coordination sphere and will dissociate from the catalyst easily. It is important to note that H_2O molecules in solution stabilize the intermediate **III**, and the resulting intermediate **IIIw$_3$** is 8.1 kcal mol^{-1} stable than **III**. Therefore, we have calculated the barrier for the proton transfer starting from **IIIw$_3$**, which is 10.8 kcal mol^{-1} (**TSIIIw$_3$-XI**) higher than the intermediate **IIIw$_3$**. While the analogous transition state with four H_2O molecules, (**TSIIIw$_4$-XI**), is further 3.3 kcal mol^{-1} higher than **TSIIIw$_3$-XI**. Therefore, H_2O molecules in solution increase the overall barrier for the side reaction.

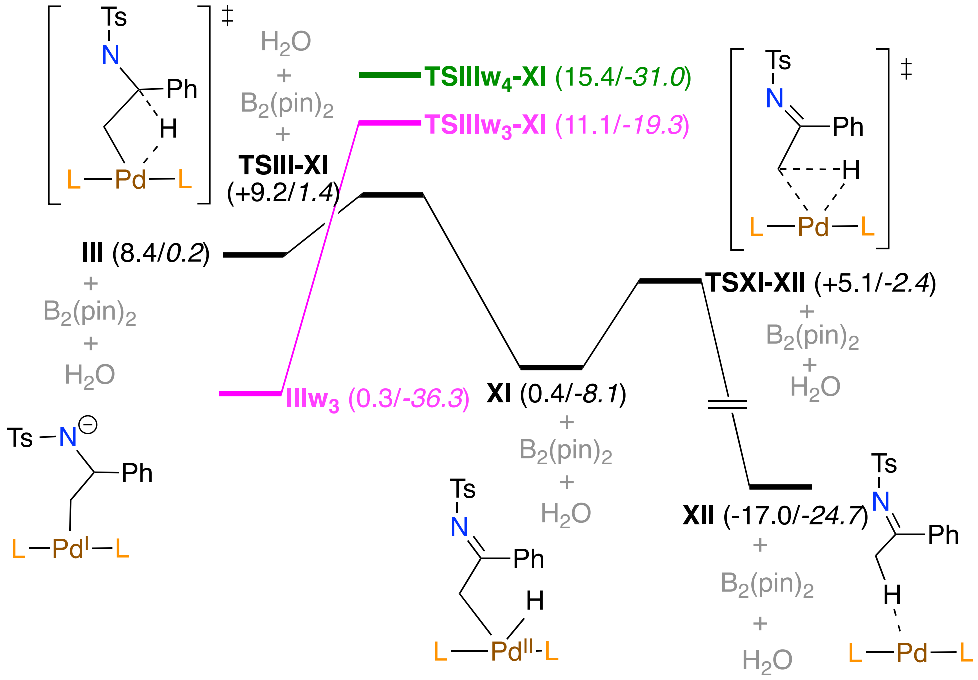

Figure S4. Free energy profile for the formation of side product **6**. Energies are in kcal mol^{-1}, ΔG values are in plane text, and ΔE values are in italics.

Energies of the structures (AU)

ZPE – Zero-point correction
G – Thermal correction to Gibbs Free Energy
E – Electronic energy

Table S15. Energies of PdL_2(A)(B)

	ZPE	G	PE
**PdL₂ (I)**	0.57327	0.51638	-1522.41303
**PL₂(H₂O) (Iw)**	0.59722	0.53433	-1598.89237
**PdL₂(bpy)**	0.73411	0.66090	-2018.01688
**PdL₃**	0.86377	0.79184	-2219.62444
**PdL₂(H₂O)₂**	0.62338	0.55962	-1675.37005
**PdL₂(H₂O)(bpy)**	0.75871	0.68181	-2094.50047
**PdL₃(H₂O)**	0.88849	0.81198	-2296.09801

Table S16. Potential Energies of **TS1** and **TS3**

	PE
**TS1**	-2706.75791
**A**	-1522.41105
**B**	-1184.30466
**TS3**	-2706.75238
**A**	-1522.40876
**B**	-1184.30767

Table S17. Energies of PdL_2(A)(B)

	ZPE	G	PE
**II**	0.84643	0.76492	-2706.76758
**III**	0.84613	0.76106	-2706.77309
**IV**	0.87423	0.79533	-2783.27320
**TSII-IIIw**	0.87104	0.78785	-2783.23898
**TSII-IIIw2**	0.89541	0.80630	-2859.72082
**TSII-IIIw3**	0.92126	0.82856	-2936.20175
**TSII-IIIw4**	0.94555	0.84845	-3012.68088
**IIIw**	0.872785	0.786413	-2783.25949
**IIIw2**	0.899366	0.810782	-2859.74690
**IIIw3**	0.924669	0.833764	-2936.23124
**IIIw4**	0.950315	0.860027	-3012.70069
**TSIII-IVw3**	0.918920	0.832340	-2936.21125
**TSIII-IVw4**	0.946210	0.856250	-3012.69372
**IVw3**	0.927074	0.840094	-2783.31392
**V**	1.24357	1.13770	-3606.22067
**VI**	0.95509	0.86475	-2909.00306
**TSVI-VII**	0.95399	0.86957	-2908.99064
**VII**	0.95458	0.86881	-2909.03436
**VIII**	1.04587	0.95790	-3118.86454

Table S18. Energies of **VIII'–X**

	ZPE	G	PE
**VII**	0.95458	0.86881	-2909.03436
**VIII**	1.04587	0.95790	-3118.86454
**IX**	1.08914	0.99485	-3046.96214
**X**	0.95414	0.86192	-2909.07902
**VII′**	0.95346	0.86145	-2909.03436
**VIII′**	1.04400	0.95224	-3118.86290
**TSVII′-X**	0.95239	0.86356	-2909.02731
**TSVIII′-IX**	1.04381	0.95090	-3118.86244

Table S19. Energies of intermediates and TSs for side reactions

	ZPE	G	PE
**TSIII-XI**	0.84424	0.76243	-2706.76386
**XI**	0.84464	0.76384	-2706.77938
**TSXI-XII**	0.84085	0.75843	-2706.76647
**XII**	0.84700	0.76498	-2706.80818
**TSIII-XIw3**	0.92126	0.82856	-2936.20175
**TSIII-XIw4**	0.94555	0.84845	-3012.68088

Table S20. Energies of each components

	ZPE	G	PE
**H_2O**	0.02112	0.00280	-76.46342
**Sol**	0.16490	0.13324	-273.10583
**L**	0.28541	0.24750	-697.18886
**bpy**	0.15838	0.12370	-495.58950
**$B_2(pin)_2$**	0.36570	0.32050	-822.92747
**HO-Bpin**	0.19751	0.16295	-487.38242
**OH^-**	0.007888	-0.00840	-75.86958
**AZ**	0.270702	0.220971	-1184.33967
**1l**	0.30398	0.25285	-1,110.51406
**TS(2-position)**	0.87783	0.78848	-2,632.92176
**TS(3-position)**	0.87811	0.79329	-2,632.92522

Table S21. Energies of TSs in Table S14

	ZPE	G	PE
TS1	0.845626	0.761906	-2705.229551
TS2	0.845633	0.763407	-2705.230564
TS3	0.845156	0.756755	-2705.225129
TS4	0.845841	0.763341	-2706.758215
TS5	0.845818	0.762028	-2706.756627
TS6	0.84501	0.760029	-2706.754504
TS7	0.844993	0.760171	-2706.75462
TS8	0.844611	0.760305	-2706.754525
TS9	0.844958	0.761838	-2706.756022
TS10	0.845007	0.760892	-2706.75465
TS11	0.845	0.762362	-2706.754706
TS12	0.845021	0.7625	-2706.754708
TS13	0.871705	0.785168	-2783.237801
TS14	0.845124	0.761891	-2706.753663
TS15	0.84481	0.762973	-2706.754659
TS16	0.845171	0.761084	-2706.752682
TS17	0.846101	0.764216	-2706.754769
TS18	0.84528	0.764191	-2706.754532
TS19	0.844714	0.761727	-2706.751878
TS20	0.871895	0.786666	-2783.237334
TS21	0.870109	0.783028	-2783.233683
TS22	0.869997	0.781377	-2783.23159
TS23	0.845736	0.762926	-2706.752299
TS24	0.84608	0.765455	-2706.754217
TS25	0.870402	0.783243	-2783.232505
TS26	0.871234	0.786029	-2783.235182
TS27	0.871019	0.786997	-2783.235964
TS28	0.871034	0.78565	-2783.234262
TS29	0.871814	0.787148	-2783.235561

TS30	0.87058	0.784854	-2783.232472
TS31	0.870802	0.783603	-2783.230976
TS32	0.870603	0.786278	-2783.233506
TS33	0.871005	0.785869	-2783.232744
TS34	0.845467	0.763512	-2783.210357
TS35	0.87009	0.783526	-2783.230083
TS36	0.869994	0.785884	-2783.232422
TS37	0.871111	0.787718	-2783.23377
TS38	0.869856	0.783115	-2783.229043
TS39	0.869942	0.785649	-2783.230926
TS40	0.869385	0.784956	-2783.230149
TS41	0.870289	0.786617	-2783.231788
TS42	0.870849	0.783286	-2783.228371
TS43	0.8711	0.783827	-2783.228556
TS44	0.869811	0.785794	-2783.230164
TS45	0.870813	0.787474	-2783.231522
TS46	0.870751	0.786416	-2783.230083
TS47	0.870968	0.787017	-2783.230243
TS48	0.871732	0.787677	-2783.230505
TS49	0.870783	0.785564	-2783.22829
TS50	0.869729	0.785781	-2783.227462
TS51	0.869725	0.787216	-2783.228519
TS52	0.871324	0.788902	-2783.230159
TS53	0.87025	0.784332	-2783.225476
TS54	0.871009	0.787578	-2783.22806
TS55	0.870687	0.78563	-2783.225326
TS56	0.871171	0.785584	-2783.224375
TS57	0.843997	0.759748	-2706.735406
TS58	0.843834	0.760768	-2706.735704
TS59	0.844338	0.76148	-2706.735976
TS60	0.843834	0.760768	-2706.735704
TS61	0.844338	0.76148	-2706.735976
TS62	0.844734	0.762564	-2706.735962
TS63	0.844521	0.762381	-2706.735742
TS64	0.844289	0.761894	-2706.735194
TS65	0.844419	0.76142	-2706.734627
TS66	0.846308	0.764884	-2706.735952
TS67	0.846311	0.764976	-2706.735953
TS68	0.845629	0.765035	-2706.735209
TS69	0.84521	0.764528	-2706.734663
TS70	0.845042	0.765973	-2706.735827
TS71	0.870234	0.787463	-2783.21612
TS72	0.870258	0.784876	-2783.212884
TS73	0.868978	0.785756	-2783.213764
TS74	0.869869	0.785205	-2783.212725
TS75	0.844865	0.766923	-2706.733564
TS76	0.870044	0.782504	-2783.209665
TS77	0.869842	0.783363	-2783.210198
TS78	0.846226	0.765022	-2706.731019
TS79	0.84623	0.76539	-2706.730985
TS80	0.8712	0.787321	-2783.213331
TS81	0.870519	0.785922	-2783.211772

TS82	0.845786	0.767907	-2706.732742
TS83	0.846021	0.768909	-2706.732604
TS84	0.845639	0.76542	-2706.728765
TS85	0.869965	0.786077	-2783.209681
TS86	0.87103	0.788677	-2783.211987
TS87	0.845217	0.767002	-2706.728447
TS88	0.871072	0.786875	-2783.208634
TS89	0.871331	0.79172	-2783.213151
TS90	0.871331	0.79172	-2783.213151
TS91	0.871196	0.787321	-2783.208137
TS92	0.871228	0.787507	-2783.208139
TS93	0.870345	0.786214	-2783.206445
TS94	0.871978	0.793267	-2783.213273
TS95	0.870584	0.789249	-2783.209027
TS96	0.872317	0.791068	-2783.210607
TS97	0.872315	0.791067	-2783.210606
TS98	0.872036	0.791285	-2783.210607
TS99	0.872022	0.791255	-2783.209863
TS100	0.871723	0.790593	-2783.209012
TS101	0.871055	0.78903	-2783.207153
TS102	0.870103	0.789344	-2783.207205
TS103	0.870102	0.789348	-2783.207205
TS104	0.870273	0.7864	-2783.203966
TS105	0.871485	0.789839	-2783.206994
TS106	0.869992	0.78614	-2783.203144

Cartesian coordinates of the optimized structures

PdL_2 (I)

Pd	3.36486200	3.22717900	17.47092400
P	5.66443900	3.41357600	17.56441000
P	1.06266900	3.05518400	17.46355900
C	6.19532900	5.02973600	18.41856300
C	5.36606800	6.19048300	17.83843500
H	4.29567000	5.96356700	17.89435600
H	5.61117500	6.39491100	16.79333800
H	5.56409300	7.10479500	18.41393100
C	5.80052700	4.86367500	19.90084000
H	5.90859300	5.82765900	20.41419400
H	6.43684400	4.13919000	20.41848800
H	4.75744400	4.54042500	19.99645600
C	7.69090200	5.36181800	18.33094800
H	7.91027800	6.23224100	18.96345000
H	7.99368600	5.61582900	17.31101400
H	8.31935200	4.53602100	18.68093000
C	6.52782200	3.20602900	15.88129100
C	6.25784200	4.44007600	15.00659900
H	6.81935300	5.31518600	15.34653200
H	5.19172300	4.69299400	14.99947400
H	6.56543000	4.23238500	13.97338500
C	5.84265500	1.98971200	15.22353500
H	4.77036700	2.16659700	15.09415300
H	5.95916200	1.07930600	15.82185500
H	6.29347700	1.80394700	14.23950100
C	8.03864600	2.93330100	15.95926800
H	8.43628100	2.80571100	14.94372400
H	8.25802400	2.01462900	16.51182100
H	8.58911000	3.75070400	16.42843000
C	6.52140600	2.12031400	18.58586100
H	7.57239900	2.35225700	18.78774300
H	6.46703100	1.16350200	18.05897900
H	5.99051600	2.00541300	19.53301600
C	0.41171100	2.43799300	19.14240600
C	-1.07807600	2.07104300	19.17823700
H	-1.29377300	1.17892800	18.58374200
H	-1.71408200	2.88656100	18.81795400
H	-1.37639500	1.85464400	20.21266800
C	1.26016800	1.23311200	19.58844800
H	2.32563400	1.48785100	19.57583100
H	1.11655700	0.36252900	18.94431000
H	0.97758100	0.94508700	20.60997600
C	0.67176900	3.58948700	20.13574700
H	0.48314300	3.23552000	21.15736700
H	0.01524700	4.44622600	19.95489000
H	1.71199900	3.93077400	20.07858700
C	0.36730300	2.01432800	16.03051500
C	-1.13555800	2.19980000	15.76477200
H	-1.37973300	3.23820800	15.52101900
H	-1.75324700	1.89518000	16.61179700
H	-1.42888800	1.58505200	14.90353000
C	0.68004100	0.52879700	16.27122400
H	0.47763700	-0.04207100	15.35554100
H	0.06144500	0.10272200	17.06661300
H	1.73336100	0.38351900	16.53597100
C	1.14565900	2.47397800	14.77954300
H	2.22175400	2.31950700	14.90782500
H	0.98446000	3.53583200	14.56281800
H	0.80583400	1.90010200	13.90707500
C	0.15069200	4.65756900	17.24327600
H	0.29862700	5.00594100	16.21736300
H	0.58007100	5.40683500	17.91147300
H	-0.92380500	4.57591100	17.43720000

$PL_2(H_2O)$ (Iw)

Pd	3.23954200	3.51037200	17.27937500
P	5.55212300	3.54311000	17.41400400
P	0.96136700	3.10037500	17.34716200
C	6.30864400	4.78549900	18.63865700
C	5.83083200	6.21000900	18.30474200
H	4.74055300	6.27554800	18.26166800
H	6.23097500	6.56941500	17.35396200
H	6.18042000	6.89585400	19.08797700
C	5.72720400	4.40575900	20.01687900
H	6.00099700	5.17707500	20.74766100
H	6.11585700	3.44999100	20.38213800
H	4.63346000	4.34363900	19.97980700
C	7.84217500	4.76396600	18.71080000
H	8.17632300	5.40271400	19.53882400
H	8.29614100	5.15430000	17.79552000
H	8.23933400	3.75967500	18.89147900
C	6.45475100	3.56742000	15.73909900
C	6.48216600	4.99738600	15.17618900
H	7.19138500	5.63526900	15.71158500
H	5.49198200	5.46485300	15.22227000
H	6.79370800	4.97023200	14.12400500
C	5.59300300	2.69679700	14.80043400
H	4.57835900	3.09824200	14.71281500
H	5.51306000	1.66390100	15.15644600
H	6.05200400	2.67148000	13.80314300
C	7.87979200	2.99058200	15.77135500
H	8.30597800	3.02364700	14.76003100
H	7.88825900	1.94536000	16.09500500
H	8.54531700	3.55472500	16.42773900
C	6.10241200	1.90971900	18.10883100
H	7.17120100	1.87113300	18.34196500
H	5.87123700	1.12673700	17.38128300
H	5.52880600	1.69660100	19.01295400
C	0.38859300	2.78987600	19.13429500
C	-1.02395500	2.20543900	19.27244200
H	-1.07743300	1.17522400	18.90952800
H	-1.77290900	2.79860000	18.73733600
H	-1.31074000	2.19345800	20.33215600
C	1.40575700	1.86924000	19.83424300
H	2.41779400	2.27941400	19.74773100
H	1.41482300	0.86123100	19.41281700
H	1.14917500	1.78574700	20.89875700
C	0.43537700	4.16237700	19.83710900
H	0.25291600	4.02157900	20.91014500
H	-0.32680800	4.84899000	19.45759800
H	1.41429600	4.63919400	19.72131400
C	0.48017200	1.65909600	16.20299900
C	-1.02755000	1.53050600	15.93140100
H	-1.43472000	2.43445300	15.46839100
H	-1.60270100	1.32397500	16.83586800

H	-1.19878200	0.70015500	15.23375600
C	1.02819700	0.34015000	16.77085200
H	0.93341600	-0.45153700	16.01623900
H	0.47731100	0.01596400	17.65853700
H	2.08767600	0.43318900	17.03539300
C	1.19539500	1.94273100	14.86493600
H	2.28068900	1.98425700	15.00032400
H	0.87613500	2.89260900	14.42182100
H	0.95841100	1.14485600	14.14859700
C	-0.17827200	4.44721700	16.77687500
H	-0.10065500	4.53182600	15.68900100
H	0.15650300	5.39294100	17.20725500
H	-1.22488900	4.26653300	17.04172400
O	2.38370200	6.54679700	17.69452000
H	2.58713100	6.73943000	16.76473600
H	2.62819500	5.58843900	17.75659900

PdL_3

Pd	3.42328600	2.16713800	18.40808100
P	5.58520300	3.07056600	17.74980500
P	1.47825800	2.76136700	17.07853700
C	5.73017600	4.97782800	17.91173800
C	4.43740500	5.62584800	17.40174900
H	3.57539100	5.21943800	17.93589000
H	4.28621400	5.45781700	16.33347000
H	4.47370500	6.71093000	17.57049300
C	5.82445900	5.27551300	19.42221100
H	5.77473900	6.36096300	19.57969900
H	6.76160000	4.92164300	19.86183100
H	4.99117400	4.81954900	19.96758000
C	6.92842400	5.61992000	17.19841500
H	6.98229000	6.68457300	17.46365000
H	6.83188100	5.56116900	16.11050800
H	7.88198000	5.16487000	17.48291300
C	6.06946100	2.52085800	15.97850200
C	5.16530400	3.22176100	14.95763700
H	5.41674600	4.28155700	14.85005000
H	4.11931600	3.14643700	15.25728700
H	5.27634800	2.75229400	13.97103900
C	5.76820700	1.00738400	15.95221100
H	4.73030300	0.80729900	16.22821300
H	6.41031700	0.45437100	16.64721800
H	5.94950400	0.61033200	14.94416300
C	7.53982200	2.72031300	15.57114800
H	7.68233100	2.33047600	14.55400000
H	8.22719900	2.17547300	16.22415500
H	7.83860700	3.76993300	15.56373500
C	7.18073400	2.59417600	18.60097800
H	8.01856100	3.22904600	18.29934900
H	7.42984300	1.56131000	18.34844400
H	7.07307000	2.65014800	19.68266200
C	0.04291500	3.40046100	18.18752000
C	-1.22706800	3.85927800	17.45801000
H	-1.73964500	3.02420300	16.97137400
H	-1.02958100	4.62816200	16.70548900
H	-1.92822700	4.29059700	18.18559200
C	-0.33503600	2.33265100	19.22140200
H	0.56343000	1.95063100	19.70392600
H	-0.87237900	1.49053600	18.78053600
H	-0.98373600	2.77403800	19.99018100
C	0.65258300	4.58953000	18.95987600
H	-0.05698500	4.93479000	19.72342500
H	0.87387300	5.43946600	18.30713900
H	1.58007400	4.28853300	19.46209800
C	0.79123900	1.49644700	15.79426800
C	-0.06097300	2.10911100	14.66806800
H	0.51332200	2.79688600	14.04167300
H	-0.93715700	2.64229200	15.04427600
H	-0.42107900	1.30171500	14.01622000
C	-0.02626800	0.38912500	16.47219800
H	-0.24170600	-0.40103600	15.74051000
H	-0.98553700	0.75711300	16.84616900
H	0.51751100	-0.06763800	17.30033900
C	2.03266900	0.84755800	15.15449700
H	2.64022900	0.33760800	15.90599100
H	2.66860300	1.58203900	14.65044200
H	1.71766800	0.10932200	14.40434500
C	1.62553300	4.23567300	15.94147200
H	2.31699800	4.00080800	15.12988900
H	2.03770800	5.08142300	16.48786400
H	0.67159900	4.53411200	15.50185100
P	3.33009300	0.74444800	20.36633700
C	2.15949900	1.10439500	21.77427600
H	2.13326700	0.30187700	22.51869400
H	2.47918700	2.02659500	22.26810100
H	1.14762600	1.26378500	21.40395000
C	2.82175200	-1.04776500	19.91282200
C	4.95984500	0.67460900	21.37739900
C	1.28829600	-1.04228100	19.77080700
H	0.96163600	-1.96136100	19.26693100
H	0.79190500	-1.00380000	20.74572100
H	0.94250400	-0.19142500	19.18279800
C	3.44512500	-1.36731000	18.54322100
H	4.53731200	-1.40496400	18.58922400
H	3.08748500	-2.34222000	18.18410100
H	3.17251900	-0.60023500	17.81191100
C	3.18805800	-2.14968400	20.91583500
H	2.80096000	-1.93565400	21.91708200
H	2.74026100	-3.09782200	20.58770000
H	4.26663600	-2.30864500	20.98746100
C	6.03493200	0.01039700	20.50384400
H	7.03485900	0.20570900	20.91322300
H	5.90140400	-1.07444200	20.46368900
H	6.00399900	0.39300200	19.48159900
C	5.32424900	2.15043900	21.63010000
H	5.27169100	2.73253000	20.71009300
H	4.64385700	2.61115900	22.35513100
H	6.34001100	2.22157100	22.04185900
C	4.91784800	-0.02098200	22.74991000
H	4.71391100	-1.08968200	22.68434900
H	5.89756200	0.09477200	23.23378000
H	4.17660000	0.42968200	23.41624200

$PdL_2(H_2O)_2$

Pd	3.29467900	3.36750600	17.30373000
P	5.61990400	3.56333500	17.48025900
P	0.98702300	2.99166400	17.34834300
C	6.29806600	4.97019300	18.57213500
C	5.77053600	6.33745100	18.10326700
H	4.67929600	6.37913300	18.10542500

H	6.12543200	6.60082100	17.10476200
H	6.13422400	7.10972700	18.79427700
C	5.73047200	4.70343500	19.98240500
H	5.95092300	5.56300500	20.62747700
H	6.17387000	3.81736800	20.44634600
H	4.64294700	4.57046400	19.95365700
C	7.83201300	5.02399400	18.64567000
H	8.13064400	5.75875100	19.40456600
H	8.27187400	5.34080800	17.69578200
H	8.27504100	4.06409900	18.92789600
C	6.52944200	3.49226800	15.80672600
C	6.51242500	4.87158800	15.13020800
H	7.18694800	5.57734000	15.62247800
H	5.50527200	5.30327300	15.12112200
H	6.84411900	4.77051300	14.08875900
C	5.71693600	2.51597000	14.93119600
H	4.69177700	2.87569000	14.79226400
H	5.66795700	1.51685200	15.37475200
H	6.19526100	2.42720000	13.94666700
C	7.97429800	2.97198000	15.89585000
H	8.41207200	2.95370600	14.88922900
H	8.01321800	1.95131800	16.28774700
H	8.61104400	3.60128500	16.52062500
C	6.28305200	2.06060000	18.34318300
H	7.35305700	2.12515500	18.55985100
H	6.08754900	1.18389800	17.72290400
H	5.73540400	1.92354200	19.27821200
C	0.43378000	2.59740900	19.12208700
C	-1.03726900	2.18008100	19.25959300
H	-1.23675100	1.21215500	18.79203200
H	-1.71823200	2.91942400	18.82525200
H	-1.29074700	2.08735600	20.32364000
C	1.35146100	1.50487600	19.70166700
H	2.40251500	1.80191900	19.61718500
H	1.23273400	0.54340700	19.19748400
H	1.11569400	1.35860300	20.76403000
C	0.66804100	3.88764700	19.93404200
H	0.48703100	3.68085600	20.99637000
H	-0.00326500	4.69653400	19.63195200
H	1.70004700	4.23987500	19.82934500
C	0.31683300	1.70135600	16.11509900
C	-1.18594200	1.84893200	15.81729100
H	-1.42436600	2.81365400	15.36093400
H	-1.80154400	1.73398200	16.71219900
H	-1.48612800	1.06796100	15.10667600
C	0.59303200	0.26833600	16.59870000
H	0.35344300	-0.43335500	15.78856600
H	-0.03395800	0.00048200	17.45354500
H	1.63911300	0.11584700	16.87593500
C	1.09979100	1.94393200	14.80836400
H	2.17539700	1.81008700	14.95868600
H	0.94729700	2.95789900	14.42160500
H	0.76024400	1.23787100	14.03920500
C	-0.04124600	4.47357900	16.91487100
H	0.04605600	4.65299000	15.83891700
H	0.35880700	5.34821100	17.42939400
H	-1.09833000	4.34348400	17.16428400
O	2.41162900	6.40168300	17.83538700
H	2.54434700	6.59411900	16.89270600
O	3.98323400	0.21507400	16.91864700
H	3.76121600	1.13899500	17.19111200
H	3.79523000	0.22679900	15.96702200
H	2.67532700	5.45021700	17.88628700

$PdL_3(H_2O)$

Pd	3.55042700	1.96258600	18.31084100
P	5.54498800	3.16888100	17.51808100
P	1.56330500	2.42569100	16.97901500
C	5.65197000	4.94664200	18.23118100
C	4.40436200	5.74378800	17.81126700
H	3.49473100	5.19228800	18.05960500
H	4.38671200	5.97193700	16.74470800
H	4.38190100	6.69861800	18.35340700
C	5.60362600	4.81888400	19.76456400
H	5.55405900	5.82186700	20.20885000
H	6.49338400	4.32573500	20.16564200
H	4.72023900	4.25915200	20.08680300
C	6.92279500	5.72423400	17.85543800
H	6.92104800	6.69402600	18.37130200
H	6.98896200	5.92599300	16.78418200
H	7.83197700	5.19616500	18.16036100
C	6.02537400	3.26497200	15.63991300
C	5.36630700	4.44646300	14.91097500
H	5.80305800	5.40783200	15.19215300
H	4.29248100	4.49387400	15.08559000
H	5.51974200	4.32565600	13.83024100
C	5.49476600	1.96362900	15.01620100
H	4.41232700	1.89072500	15.14260200
H	5.94165900	1.08139500	15.48110400
H	5.72412000	1.94398300	13.94188300
C	7.54212700	3.33473600	15.37869700
H	7.71003300	3.42299000	14.29692900
H	8.05923200	2.43210300	15.71374500
H	8.01560100	4.19709000	15.85501000
C	7.12078000	2.41083400	18.15944900
H	8.01517200	2.99899400	17.94076200
H	7.22229800	1.42271800	17.70235500
H	7.05143600	2.26486000	19.23514800
C	0.13546600	3.33545500	17.88931600
C	-1.09829800	3.66602200	17.03620800
H	-1.64642700	2.76515700	16.74639400
H	-0.84755400	4.22251500	16.12827000
H	-1.78630900	4.29070600	17.62176600
C	-0.31030700	2.52945200	19.11552800
H	0.54579100	2.30275100	19.75147700
H	-0.79639700	1.58937100	18.84855700
H	-1.02734600	3.11897800	19.70240600
C	0.75388900	4.64727100	18.41349100
H	0.03005300	5.15731400	19.06225000
H	1.01694900	5.33754300	17.60680300
H	1.65465900	4.44634800	19.00423600
C	0.82984300	1.02765700	15.87878100
C	0.04932200	1.50775300	14.64146600
H	0.67587900	2.09136800	13.96135400
H	-0.82446900	2.10959700	14.89792600
H	-0.30664300	0.63110600	14.08364000
C	-0.06759400	0.09183400	16.70061600
H	-0.32035900	-0.79152900	16.09936700
H	-1.00853300	0.57069700	16.98599700
H	0.43190200	-0.25579900	17.60656100

C	2.04265300	0.21761300	15.37950100
H	2.64692600	-0.14938600	16.21374000
H	2.68919600	0.81571900	14.73078500
H	1.69509600	-0.64537600	14.79605400
C	1.89906200	3.68405400	15.64939300
H	2.54278100	3.23272400	14.89074500
H	2.43994500	4.52139600	16.08885900
H	0.99789900	4.06103600	15.15834900
P	3.49786500	0.67858500	20.36544600
C	2.57277700	1.60777100	21.68983500
H	2.44119700	1.02525300	22.60748200
H	3.12246700	2.52164200	21.92903900
H	1.58947700	1.90217300	21.32415800
C	2.51970800	-0.97657100	20.29703700
C	5.14064000	0.36578100	21.33002600
C	1.02348400	-0.62417600	20.29402300
H	0.44028000	-1.51399500	20.02271500
H	0.67796200	-0.28886200	21.27692600
H	0.80156100	0.15733200	19.56846400
C	2.85802800	-1.66230200	18.96381100
H	3.92235900	-1.89009500	18.86849000
H	2.29315600	-2.60055300	18.87535600
H	2.58534200	-1.01632000	18.12611600
C	2.76197100	-1.96744600	21.44239600
H	2.60135900	-1.51445600	22.42510100
H	2.05307800	-2.80081600	21.34399400
H	3.76726100	-2.39411400	21.41233300
C	6.05686800	-0.51154400	20.46504600
H	7.03783400	-0.61259700	20.94906600
H	5.64843100	-1.51825000	20.33252800
H	6.21194000	-0.08482300	19.47288000
C	5.76761300	1.76173300	21.48448100
H	5.65324000	2.34001000	20.57110100
H	5.30277700	2.32656200	22.29937700
H	6.83707900	1.67008300	21.71543400
C	5.05246900	-0.23587700	22.74603200
H	4.84926000	-1.30549200	22.74664300
H	6.02201300	-0.09252600	23.24227300
H	4.29975800	0.25963100	23.36673900
O	5.40328700	-0.51220700	17.16903000
H	4.83915600	-0.67327100	16.39700500
H	4.92844400	0.21919900	17.63275200

II

Pd	-1.24672700	-0.56142600	0.02498600
P	-2.11302300	0.73426600	1.73648300
P	-0.75786300	-1.91880700	-1.78869500
C	-2.91405600	2.32257600	1.05930100
C	-0.92180400	1.39545300	2.99517200
C	-3.34449400	-0.25476800	2.79814600
C	-2.14987300	-1.72484700	-3.07531200
C	-0.49125900	-3.73954300	-1.31225100
C	0.73870100	-1.54255200	-2.81623700
H	1.62027000	-1.41379100	-2.18730600
H	0.57714400	-0.59467000	-3.33739600
H	0.94846700	-2.31611300	-3.56187600
H	-1.41768700	1.80517300	3.88071900
H	-0.24514400	0.59004100	3.28991900
H	-0.32359800	2.18847900	2.53536900
C	1.82637300	2.07851800	-1.34806000
C	1.52754400	1.16881900	-0.21011900
H	1.94660100	1.57480600	-2.30467300
H	1.40528400	0.11243200	-0.42753600
H	0.96446700	1.53134200	0.64146500
C	1.29949200	3.46821900	-1.40592300
C	0.45676900	3.82537200	-2.46420100
C	1.56604900	4.39826500	-0.39511700
C	-0.12809900	5.09103800	-2.50396500
H	0.25197200	3.10506500	-3.25251800
C	0.98492200	5.66517500	-0.43812500
H	2.23718100	4.12227300	0.41194300
C	0.13237400	6.01347000	-1.48868800
H	-0.78376300	5.35661800	-3.32831000
H	1.20033400	6.38309300	0.34827100
H	-0.32059900	7.00022400	-1.51933900
N	2.86798300	1.77396200	-0.33470500
S	4.11385000	0.70636400	-0.82053800
O	5.33738400	1.50801700	-0.90219200
O	3.69951300	-0.09977700	-1.98494400
C	4.14457800	-0.36459400	0.60185000
C	4.40577400	0.17296300	1.86356300
C	3.85193500	-1.71829300	0.43583600
C	4.34528900	-0.66331100	2.97520100
H	4.63588600	1.22712400	1.97262300
C	3.80796500	-2.54147100	1.55930600
H	3.65369300	-2.10995000	-0.55468500
C	4.03567100	-2.02561200	2.84354800
H	4.53173600	-0.25211500	3.96305200
H	3.56789800	-3.59387000	1.43986300
C	-1.83642000	-2.30235100	-4.46502700
H	-1.68289500	-3.38259600	-4.44676800
H	-2.68031400	-2.09962800	-5.13751800
H	-0.94939900	-1.83996700	-4.90833000
C	-3.44693800	-2.34013900	-2.52688700
H	-3.65880600	-1.97413000	-1.51627100
H	-4.28806100	-2.06143300	-3.17503400
H	-3.40235300	-3.43266100	-2.49858800
C	-2.36239700	-0.20213400	-3.21531200
H	-3.17626300	-0.01153900	-3.92737800
H	-2.62258600	0.24697400	-2.25130600
H	-1.46714700	0.30664500	-3.58983600
C	-4.22727900	-1.10641400	1.86718400
H	-3.60808500	-1.73684600	1.22058800
H	-4.87723800	-1.75284100	2.47196200
H	-4.86668200	-0.49530600	1.22572500
C	-2.49988800	-1.21574900	3.65942100
H	-3.17071800	-1.90805500	4.18412400
H	-1.81198800	-1.80821100	3.04891200
H	-1.91212300	-0.68545600	4.41476100
C	-4.22573100	0.59607200	3.72230000
H	-4.80956100	-0.06329300	4.37782800
H	-3.63240000	1.25441700	4.36539500
H	-4.93567500	1.21132800	3.16257900
C	-4.20276000	1.96682400	0.30025900
H	-4.54831200	2.84333500	-0.26289500
H	-4.03100300	1.15219000	-0.41171500
H	-5.01063200	1.66944400	0.97485100
C	-3.20270700	3.40310800	2.11435000

H	-3.61488700	4.29019300	1.61571900
H	-3.92654000	3.08075100	2.86491800
H	-2.29096200	3.71715400	2.63167100
C	-1.90105200	2.89154500	0.04627000
H	-1.68001800	2.16651400	-0.74252400
H	-2.30959300	3.79835000	-0.41708400
H	-0.95331500	3.17099800	0.51629200
C	-1.52523600	-4.12599300	-0.23858600
H	-1.31222300	-5.13925000	0.12715400
H	-1.48063000	-3.43254400	0.60715300
H	-2.54713300	-4.11760300	-0.62612400
C	-0.55454000	-4.73297600	-2.48016700
H	-0.25654700	-5.72913300	-2.12789800
H	-1.56617100	-4.82044300	-2.88626300
H	0.12163100	-4.45765300	-3.29626200
C	0.90821400	-3.80775300	-0.66694400
H	1.00478700	-3.09544600	0.15801300
H	1.06318500	-4.81344400	-0.25534000
H	1.70568500	-3.62134400	-1.39303000
C	3.89174000	-2.90761700	4.05599900
H	2.83393200	-2.98173700	4.33826000
H	4.25047500	-3.92247500	3.85718500
H	4.44011500	-2.50618200	4.91318200

III

Pd	0.20820100	-1.66243400	-0.77792600
P	2.11196300	-1.20367400	0.57943600
P	-1.61757800	-2.61015300	-1.92478400
C	1.85507700	-1.70756800	2.39178100
C	2.77887900	0.50629500	0.71987400
C	3.51853600	-2.18067700	-0.24060700
C	-2.37654800	-3.93741800	-0.80338200
C	-1.06017000	-3.30111400	-3.59973800
C	-3.06138800	-1.55001100	-2.35707800
H	-2.70783300	-0.62943200	-2.82504000
H	-3.60930800	-1.28370100	-1.44997500
H	-3.74393300	-2.06555700	-3.03850500
H	3.74294000	0.52008000	1.23550500
H	2.90399000	0.94609300	-0.27076300
H	2.05947700	1.12056700	1.26888500
C	-0.22641900	1.30667500	-0.94864500
C	-0.83638200	0.07168300	-0.30194400
H	0.82429000	1.38088500	-0.65459600
H	-0.75777800	0.12990900	0.78068000
H	-1.87086500	-0.06253000	-0.61096100
C	-0.26016700	1.30964500	-2.46183200
C	-1.43044500	1.63527600	-3.15755900
C	0.89711400	1.01772300	-3.19207600
C	-1.44665200	1.64538900	-4.55345300
H	-2.31118600	1.91698800	-2.58942300
C	0.88927900	1.03795600	-4.58844400
H	1.81576100	0.78920700	-2.65823900
C	-0.28681500	1.34734300	-5.27445900
H	-2.36174600	1.90340500	-5.08004700
H	1.80087700	0.81944500	-5.13854400
H	-0.29786300	1.36689400	-6.36064400
N	-1.05670300	2.37883700	-0.39674300
S	-0.37638800	3.09216400	0.86024500
O	0.17437300	2.15667100	1.89684800
O	-1.27794000	4.14620300	1.36857100
C	1.10998200	3.90079500	0.21025900
C	2.22205300	4.08767200	1.03274300
C	1.14107100	4.33083500	-1.11680800
C	3.36189100	4.70754500	0.52373400
H	2.19242900	3.72914200	2.05590400
C	2.28860900	4.94438600	-1.61750800
H	0.27844500	4.15635300	-1.75089200
C	3.41314200	5.14736500	-0.80653000
H	4.22885500	4.84781300	1.16537300
H	2.31353500	5.26654600	-2.65586400
C	-3.74187000	-4.45483800	-1.28550100
H	-3.68993200	-4.93721100	-2.26250600
H	-4.11483800	-5.19657000	-0.56819500
H	-4.48273700	-3.65153100	-1.33806000
C	-1.38616700	-5.10249700	-0.64537300
H	-0.39619100	-4.74319600	-0.34174000
H	-1.74756200	-5.78470000	0.13399100
H	-1.27632200	-5.68261500	-1.56582700
C	-2.56412800	-3.26907500	0.57524200
H	-3.02349300	-3.98901900	1.26389700
H	-1.60572200	-2.95054200	0.99514500
H	-3.21831000	-2.39217100	0.52897500
C	3.03324300	-3.60695700	-0.56472200
H	2.07875000	-3.59761800	-1.10414300
H	3.76919900	-4.10559200	-1.20801400
H	2.90284100	-4.21884500	0.32905800
C	3.80337000	-1.46104700	-1.57625000
H	4.52873000	-2.04796200	-2.15302700
H	2.89509900	-1.35854700	-2.18150400
H	4.22901100	-0.46486800	-1.42532000
C	4.81216500	-2.24747000	0.58261800
H	5.59908500	-2.72536200	-0.01485400
H	5.17175300	-1.25265400	0.86365200
H	4.68733300	-2.83925400	1.49334600
C	1.75338500	-3.23572800	2.51571000
H	1.40877300	-3.48973200	3.52562300
H	1.03238600	-3.65242100	1.80332900
H	2.71746200	-3.72830500	2.36397200
C	2.95691600	-1.17742300	3.32599000
H	2.75419500	-1.53026600	4.34479000
H	3.95464400	-1.52471000	3.04798800
H	2.96622000	-0.08468200	3.35669200
C	0.50946700	-1.09152100	2.82638100
H	-0.32323900	-1.54216000	2.27837600
H	0.35910000	-1.29355900	3.89454300
H	0.46928100	-0.00850000	2.67870200
C	0.34979500	-3.90018000	-3.44667900
H	0.69289000	-4.27417100	-4.41940900
H	1.06062000	-3.13574900	-3.11443300
H	0.38427700	-4.73197600	-2.73848900
C	-2.01068000	-4.33942900	-4.21029300
H	-1.67045400	-4.58126200	-5.22516600
H	-2.02793900	-5.27180100	-3.63960600
H	-3.03537200	-3.96307500	-4.29036900
C	-0.95797300	-2.08555800	-4.54100900
H	-0.35911200	-1.28175900	-4.10592900
H	-0.47250800	-2.39764900	-5.47418500
H	-1.93854400	-1.67723300	-4.79911000
C	4.64059600	5.84182500	-1.34463400
H	4.75309000	5.67900700	-2.42165100

H	4.58150300	6.92646000	-1.18392800
H	5.55090000	5.48979700	-0.84777300

IV

Pd	1.67206200	0.00428900	-0.07305100
P	1.73217500	-2.37801100	0.17497700
P	1.82959200	2.35539800	-0.33713900
C	0.57724800	-3.36153600	-0.88475200
C	1.29486300	-2.99618600	1.92360900
C	3.41998900	-3.04810400	-0.40256200
C	2.28289100	3.31427500	1.24578700
C	0.29806300	3.23426100	-0.87337200
C	3.04982300	2.71875500	-1.74537200
H	-0.42394700	3.21697700	-0.05560400
H	0.49529200	4.27154700	-1.15426500
H	-0.15712600	2.71965000	-1.71821200
H	0.62400100	-4.42642500	-0.64387400
H	-0.45398500	-3.03422000	-0.76204300
H	0.85990200	-3.23351500	-1.93291800
C	-1.29314900	-0.45931600	-0.73147600
C	0.05276700	-0.15619500	-1.39775500
H	-1.14626800	-1.23268000	0.02390200
H	0.30603900	-0.98630800	-2.07081700
H	-0.05349000	0.73286500	-2.02472300
C	-1.90237500	0.74865000	-0.04142400
C	-2.75002000	1.62705400	-0.72307500
C	-1.61810800	0.99686700	1.30410000
C	-3.31072700	2.72455400	-0.07140300
H	-2.99296300	1.42232400	-1.75975500
C	-2.17681600	2.09395900	1.96207400
H	-0.94986000	0.32868300	1.83901500
C	-3.02738300	2.96310500	1.27574300
H	-3.97753800	3.38922400	-0.61356600
H	-1.95122400	2.26616400	3.01095800
H	-3.46918300	3.81385700	1.78709100
N	-2.23983800	-0.98841700	-1.75898500
S	-3.40828300	-2.09315200	-1.25117700
O	-2.87975300	-2.97886400	-0.19536200
O	-3.94892600	-2.68140100	-2.48531500
C	-4.61574200	-1.02893200	-0.49688300
C	-4.56932600	-0.81676600	0.88057700
C	-5.51264400	-0.33655000	-1.30853400
C	-5.41906700	0.12864200	1.44416800
H	-3.86668000	-1.37298400	1.48921000
C	-6.36141600	0.60037300	-0.72540300
H	-5.53610600	-0.52560300	-2.37622400
C	-6.31165800	0.86217500	0.65072600
H	-5.37602400	0.31509900	2.51344200
H	-7.06291800	1.14814800	-1.34859600
H	-1.76007200	-1.40098600	-2.55782100
C	-7.16816800	1.93835800	1.26690100
H	-6.56819900	2.83842900	1.45493900
H	-7.99634700	2.22094300	0.61033800
H	-7.58380000	1.61933200	2.22863400
C	1.49648100	2.63919600	2.38609200
H	1.68668800	3.17885100	3.32330100
H	0.41898700	2.65187700	2.20214500
H	1.81402000	1.60155300	2.50909000
C	3.78540400	3.21598200	1.55920300
H	4.10745800	2.17206100	1.52455600
H	4.38693800	3.81255700	0.86671000
H	3.95817400	3.61862300	2.56667200
C	1.86792500	4.79617200	1.18151600
H	2.19484300	5.28758900	2.10674900
H	2.33102800	5.32752700	0.34632500
H	0.78383500	4.92191200	1.11032000
C	4.36316800	1.95151800	-1.51387400
H	4.92528400	2.33620600	-0.66283400
H	4.17402700	0.89388600	-1.32285900
H	4.99151300	2.04438600	-2.40939900
C	3.34768700	4.21366500	-1.93818100
H	3.88380600	4.63697400	-1.08529700
H	3.98510000	4.34065000	-2.82290200
H	2.44005400	4.80314500	-2.10329500
C	2.39727500	2.16589800	-3.02997200
H	2.15460100	1.10392700	-2.92603200
H	1.48424000	2.70547500	-3.29983000
H	3.10365600	2.27403300	-3.86243200
C	1.99296000	-2.12656900	2.98265000
H	3.08171500	-2.16958500	2.91204700
H	1.67675900	-1.08304100	2.88937700
H	1.70516500	-2.47797500	3.98175800
C	-0.22516800	-2.81607600	2.10458800
H	-0.52066700	-1.77291400	1.96436200
H	-0.81894900	-3.43041400	1.42429500
H	-0.49366200	-3.09837400	3.13045300
C	1.64647000	-4.47375400	2.15614000
H	1.26869500	-4.77898300	3.14030800
H	1.18613700	-5.13121900	1.41259800
H	2.72540600	-4.64570300	2.15096700
C	4.50464100	-2.76809200	0.64984500
H	5.48674100	-3.00236700	0.21771200
H	4.48048700	-1.71202400	0.93773300
H	4.38323900	-3.39679700	1.53783200
C	3.76001200	-2.25109100	-1.67949800
H	4.70222900	-2.62840900	-2.09761700
H	2.98879200	-2.35626500	-2.45157400
H	3.88060200	-1.18893100	-1.45796800
C	3.40307800	-4.54517500	-0.76047300
H	4.41557200	-4.83763900	-1.06641800
H	3.11849800	-5.17976700	0.08068600
H	2.73455100	-4.76512700	-1.59785600
O	3.47448200	0.16024900	1.07292000
H	3.24468500	-0.08956500	1.97947800

VI

Pd	0.37584700	0.12935600	0.14935300
P	1.16028900	-1.63092500	1.38266200
C	0.08822000	-3.12862800	1.50673800
C	1.43936100	-1.19781900	3.20543800
C	2.73884200	-2.27877400	0.55581000
H	0.63930300	-3.97264400	1.92779200
H	-0.76673900	-2.91581400	2.15243300
H	-0.29290200	-3.41518500	0.52678200
C	-2.21902900	-1.20130500	-0.22805800
C	-0.85029400	-1.10362400	-0.89719000
H	-2.13854200	-1.80193600	0.68320100
H	-0.44598200	-2.10296600	-1.07957400
H	-0.91852700	-0.57416500	-1.85283600
C	-2.79427000	0.14874500	0.15813100

C	-3.12440300	1.09706600	-0.81776100
C	-3.02697500	0.44889700	1.50202900
C	-3.66664800	2.32752200	-0.45120900
H	-2.97424500	0.85743500	-1.86503200
C	-3.57067400	1.68003700	1.87362900
H	-2.79350000	-0.29490300	2.25803800
C	-3.89082600	2.62277200	0.89759200
H	-3.92640000	3.05152000	-1.21793200
H	-3.74813300	1.89789800	2.92290400
H	-4.31864000	3.57981000	1.18193700
N	-3.13207600	-1.88456500	-1.18640100
S	-4.61971800	-2.42507000	-0.58830700
O	-4.53892300	-2.69649400	0.85716200
O	-5.02948700	-3.49209700	-1.51402100
C	-5.65795700	-0.99843100	-0.82058700
C	-6.15312900	-0.31883200	0.28877600
C	-5.91719500	-0.55791000	-2.11952900
C	-6.90258600	0.83779900	0.08899900
H	-5.93122900	-0.68008500	1.28522700
C	-6.66907800	0.59782200	-2.29938100
H	-5.52448500	-1.10487300	-2.96967500
C	-7.16059100	1.31902500	-1.20043000
H	-7.27998600	1.38349600	0.94919100
H	-6.87286800	0.95234400	-3.30628600
H	-2.69125300	-2.68200000	-1.64489900
O	-0.02037900	1.74829700	-1.20606300
H	-0.78914700	2.25017100	-0.88171200
C	3.69473900	1.15705100	-3.64898900
C	4.69700400	1.78035400	-2.61659600
C	1.46183900	4.36348100	0.55666000
C	1.10867500	3.16839500	1.50591500
B	2.54938400	1.93441500	-1.80687300
B	1.27172800	2.45044700	-0.78106800
C	-7.91562200	2.60835500	-1.40275500
H	-8.64519000	2.77558400	-0.60419400
H	-7.22568900	3.46262800	-1.39977100
H	-8.44419800	2.61834900	-2.36106000
C	1.90472600	3.15367800	2.80723400
H	1.79695400	4.11240800	3.32785300
H	1.53831800	2.37107100	3.47777500
H	2.96590600	2.97691000	2.61804000
C	-0.39165300	3.11754300	1.81653600
H	-0.66374000	2.12736100	2.18696100
H	-0.66456900	3.85409200	2.58048500
H	-1.00249400	3.31308800	0.93329600
C	0.70178800	5.65271700	0.86786000
H	0.91803600	6.00392400	1.88362600
H	1.00962700	6.43523500	0.16642000
H	-0.37727600	5.51501200	0.76565000
C	2.97307300	4.64934400	0.52420000
H	3.17525000	5.34582300	-0.29622700
H	3.32307300	5.10652100	1.45633300
H	3.54068100	3.73474400	0.33808400
C	5.12303600	3.20879300	-2.97608800
H	5.64499700	3.64650700	-2.11959500
H	5.79539100	3.22748100	-3.83992800
H	4.25129600	3.83236700	-3.19539200
C	5.92327600	0.92810300	-2.31028000
H	6.54043400	1.42958600	-1.55764100
H	5.64419000	-0.05290400	-1.92044100
H	6.53228700	0.78681300	-3.21048700
C	3.66704400	-0.37533400	-3.61963100
H	2.81237800	-0.72342500	-4.20805700
H	4.57935300	-0.80637200	-4.04527400
H	3.54771900	-0.74582700	-2.59865400
C	3.86134700	1.64743900	-5.08363600
H	4.85187500	1.38338000	-5.47183900
H	3.10856100	1.17611500	-5.72341200
H	3.73514500	2.72984500	-5.15445900
C	2.67354300	-0.29278500	3.34562200
H	3.60102100	-0.86171200	3.23861800
H	2.67200900	0.51571100	2.61179700
H	2.67865600	0.15665900	4.34624300
C	0.18448700	-0.42375100	3.65257700
H	0.06745700	0.50264300	3.08987000
H	-0.73004200	-1.01378400	3.52835000
H	0.27584300	-0.17322400	4.71668000
C	1.59336100	-2.43233300	4.11281200
H	1.78538300	-2.08738300	5.13639300
H	0.68431200	-3.03961800	4.14056400
H	2.42628100	-3.07469900	3.82105600
C	3.69419100	-1.10951900	0.25901500
H	4.52576200	-1.48369000	-0.35081500
H	3.20713900	-0.30393800	-0.29352900
H	4.12164200	-0.67687400	1.16452800
C	2.30846700	-2.89422400	-0.79205800
H	3.20594100	-3.22399700	-1.32880300
H	1.66190000	-3.76866400	-0.66716300
H	1.79424900	-2.16088900	-1.41946200
C	3.47490100	-3.34413500	1.38439000
H	4.31057500	-3.73514500	0.79094600
H	3.89515900	-2.92751600	2.30320000
H	2.83593500	-4.19338000	1.64653400
O	1.49720300	2.03777600	0.68368900
O	1.04956000	3.88576500	-0.72086400
O	3.88223700	1.88224000	-1.42014600
O	2.41193600	1.61028100	-3.14545500

TSVI-VII

Pd	0.88301500	-0.63599000	-0.28831500
P	1.49419900	-2.22537000	1.21587300
C	0.10612400	-3.33179800	1.72977800
C	1.99448800	-1.45228200	2.87642200
C	2.75831300	-3.48651000	0.56907800
H	0.40737200	-4.00615200	2.53403600
H	-0.73588000	-2.73360200	2.07315400
H	-0.22947900	-3.92974700	0.88042100
C	-2.13803700	-1.53687400	-0.14842800
C	-0.82360700	-1.73122300	-0.88995300
H	-2.08626100	-2.01803700	0.83072200
H	-0.61352600	-2.80345900	-0.97883600
H	-0.91738600	-1.32477600	-1.90784300
C	-2.50266600	-0.08359300	0.08123200
C	-2.83445600	0.75595500	-0.99142600
C	-2.54857700	0.43015400	1.37951300
C	-3.21158000	2.08042300	-0.76524800
H	-2.82988000	0.35435800	-1.99954800
C	-2.92909500	1.75252400	1.60964100
H	-2.30107100	-0.21729500	2.21565800
C	-3.26727400	2.57971700	0.53869900

H	-3.47923200	2.71480800	-1.60514200
H	-2.96617900	2.13372900	2.62626700
H	-3.57141700	3.60713800	0.71580300
N	-3.22063200	-2.19633000	-0.94327700
S	-4.70141900	-2.47610200	-0.18067400
O	-4.53523100	-2.60398500	1.27782200
O	-5.32391200	-3.56843600	-0.94547900
C	-5.57935200	-0.95834200	-0.49388000
C	-5.85165000	-0.09114500	0.56040200
C	-5.93505300	-0.64169000	-1.80536800
C	-6.47460100	1.12418800	0.28872900
H	-5.55513500	-0.36095500	1.56622900
C	-6.55812800	0.57589000	-2.05782800
H	-5.71718600	-1.33396300	-2.61128200
C	-6.82580800	1.48030000	-1.01915000
H	-6.67662900	1.81338600	1.10383800
H	-6.83582400	0.83421200	-3.07613700
H	-2.91506800	-3.08576600	-1.33756800
O	0.28249000	1.28655400	-1.16427800
H	-0.63006400	1.40290700	-0.83972800
C	4.15175300	0.13693900	-2.64008100
C	5.00525400	0.55590700	-1.38668800
C	2.34269900	3.60058000	0.65439500
C	1.07264200	3.28362000	1.52630300
B	2.79347100	0.27138400	-0.76374900
B	1.24981700	1.71859200	-0.16148500
C	-7.44120600	2.82618800	-1.30847100
H	-8.09428500	3.15197900	-0.49246800
H	-6.66082800	3.58981400	-1.42586500
H	-8.02646700	2.81034900	-2.23299300
C	1.29334800	3.31940600	3.03567900
H	1.59428600	4.31940700	3.36747100
H	0.35874000	3.06001700	3.54395600
H	2.05632200	2.60165200	3.34330500
C	-0.13247800	4.15672600	1.15831100
H	-1.01547500	3.77263400	1.67270400
H	0.01932500	5.20054200	1.45217700
H	-0.33084300	4.12312100	0.08258300
C	2.42412600	5.04131800	0.15192600
H	2.49555200	5.74258800	0.99138100
H	3.31932900	5.16137700	-0.46663100
H	1.55603200	5.30604700	-0.45457000
C	3.65251700	3.24332300	1.36304200
H	4.49285900	3.45536100	0.70202800
H	3.77678500	3.84716400	2.26725400
H	3.69640900	2.18711800	1.62915600
C	5.37551300	2.03936500	-1.38892000
H	5.87591000	2.28495300	-0.44849200
H	6.06447600	2.26644600	-2.20865300
H	4.48527900	2.66327400	-1.48350600
C	6.26279500	-0.27680200	-1.15049800
H	6.74197400	0.04824600	-0.22157500
H	6.04748200	-1.34291100	-1.06605300
H	6.97690100	-0.13202700	-1.96881200
C	4.35932200	-1.31838800	-3.06912200
H	3.59294600	-1.58298900	-3.80414600
H	5.34323400	-1.46126800	-3.52819600
H	4.26837900	-2.00500300	-2.22556800
C	4.29263300	1.05224700	-3.85369900
H	5.32172700	1.05018400	-4.23021100
H	3.63689000	0.69386400	-4.65338300
H	4.00512800	2.07751700	-3.61427300
C	3.06612000	-0.37237600	2.67424700
H	3.98409100	-0.75543100	2.22798100
H	2.68347100	0.42165600	2.03599800
H	3.31535900	0.05887000	3.65255500
C	0.72969700	-0.75459100	3.42333700
H	0.32044000	-0.04782400	2.69535600
H	-0.04711500	-1.46703400	3.71653500
H	1.00766700	-0.18583000	4.31952700
C	2.49287200	-2.49111800	3.89409500
H	2.65306300	-1.98972500	4.85673200
H	1.77431000	-3.29825200	4.06465200
H	3.44700700	-2.93325500	3.59434100
C	4.17737500	-2.90374600	0.61455700
H	4.84875600	-3.54876300	0.03307500
H	4.21883700	-1.89643800	0.20067300
H	4.56542800	-2.86664200	1.63693900
C	2.35516600	-3.75494200	-0.89506900
H	3.06521700	-4.46230300	-1.34190600
H	1.35624600	-4.19855600	-0.96400000
H	2.35329600	-2.83771400	-1.48830100
C	2.73215400	-4.82308700	1.33273900
H	3.51257800	-5.47381000	0.91887400
H	2.93467500	-4.70343500	2.39916400
H	1.77895900	-5.34617200	1.21883700
O	0.76173400	1.92593600	1.15022300
O	2.15231900	2.75095300	-0.50656800
O	2.79159300	0.25373300	-2.15024100
O	4.08479300	0.32218600	-0.27295700

VII

Pd	1.19482600	0.72034400	0.74851300
P	0.94562300	3.14592900	0.08102400
C	-0.59000400	3.92683400	0.76676700
C	0.82416500	3.55422400	-1.77054200
C	2.29478000	4.18690500	0.93601100
H	-0.69382500	4.97593500	0.47738000
H	-1.47575800	3.39119400	0.42498900
H	-0.55776500	3.87224100	1.85880100
C	-1.78002400	0.58655100	0.69582600
C	-0.62846600	0.58557300	1.70530000
H	-1.54281200	1.27452200	-0.11858600
H	-0.64428300	1.51756300	2.28787600
H	-0.77758600	-0.23560800	2.41391700
C	-2.03751300	-0.78380100	0.09560700
C	-2.85875600	-1.71266800	0.73974700
C	-1.44874000	-1.13262300	-1.12193700
C	-3.10070400	-2.96201100	0.17018400
H	-3.33677700	-1.43456100	1.67249800
C	-1.68231700	-2.38342000	-1.69297500
H	-0.79343400	-0.42021000	-1.61563200
C	-2.51206600	-3.30324900	-1.04945300
H	-3.75769000	-3.66542900	0.67486600
H	-1.22057500	-2.63945900	-2.64217100
H	-2.70441700	-4.27442000	-1.49787100
N	-3.01004100	1.08118300	1.38728400
S	-4.19256500	1.83321000	0.45121200
O	-3.59159800	2.57810300	-0.67189300
O	-5.05145800	2.54130000	1.41283700

C	-5.06515300	0.44558100	-0.23732900
C	-4.73774800	0.00060200	-1.51736200
C	-5.98220200	-0.23962600	0.55776000
C	-5.32237700	-1.16843000	-1.99239400
H	-4.02250300	0.55324200	-2.11412600
C	-6.56264000	-1.40412800	0.06267000
H	-6.22729000	0.13164000	1.54682600
C	-6.22636800	-1.89539400	-1.20627700
H	-5.05725100	-1.53439500	-2.98015100
H	-7.27652300	-1.94844200	0.67483000
H	-2.79606100	1.71228900	2.15826000
O	3.43213000	0.59132300	0.02172700
H	3.86770700	0.35974300	0.85808200
C	1.04011300	-3.37227600	1.97150200
C	2.15369200	-2.80286600	2.91732900
C	3.11849900	-1.66404800	-2.74293800
C	4.30306300	-2.29972000	-1.92450800
B	1.34912100	-1.13517600	1.53497200
B	3.53684700	-0.43734700	-0.89607900
C	-6.78908600	-3.20160100	-1.70522500
H	-7.04021500	-3.15010400	-2.76993900
H	-6.04950600	-4.00413000	-1.58454500
H	-7.68777500	-3.49169300	-1.15268800
C	4.18080400	-3.79774700	-1.68926600
H	4.20869300	-4.33558800	-2.64305700
H	5.01807000	-4.14642300	-1.07675300
H	3.25062400	-4.04573400	-1.17670500
C	5.68319800	-1.95237800	-2.48614700
H	6.44710000	-2.25013100	-1.76181900
H	5.87525000	-2.47780300	-3.42652300
H	5.78005900	-0.87669400	-2.66363000
C	3.38363400	-1.50874600	-4.23411100
H	3.54599700	-2.48775500	-4.69767700
H	2.51594300	-1.04545600	-4.71369700
H	4.25579400	-0.88032100	-4.42608200
C	1.77709200	-2.35492700	-2.49667400
H	0.98752700	-1.74098100	-2.93813000
H	1.74821800	-3.34278900	-2.96777000
H	1.56157800	-2.46165700	-1.42985700
C	3.57377900	-3.15655800	2.46603100
H	4.28738000	-2.57685600	3.06096000
H	3.78902300	-4.21987600	2.61726100
H	3.72879600	-2.90815600	1.41323000
C	1.97247300	-3.13394200	4.39469900
H	2.78143000	-2.67965500	4.97593200
H	1.02360000	-2.75301600	4.77819600
H	2.00574600	-4.21768600	4.55587000
C	-0.32335000	-3.50765700	2.65452300
H	-1.07878000	-3.72091500	1.89497500
H	-0.32261800	-4.31613300	3.39314000
H	-0.60828000	-2.57739300	3.15503100
C	1.39334500	-4.67251800	1.26001800
H	1.51402300	-5.49059800	1.97918800
H	0.58517700	-4.93804800	0.57097700
H	2.31650800	-4.57908000	0.68563500
C	1.96385500	2.84520800	-2.52340300
H	2.94898100	3.23184400	-2.25402700
H	1.96253600	1.76868500	-2.33834600
H	1.83150900	3.00242100	-3.60247000
C	-0.50423000	2.93528700	-2.25183900
H	-0.54353600	1.86412100	-2.02793000
H	-1.38473400	3.40482800	-1.80763700
H	-0.57623700	3.05013600	-3.34115900
C	0.83272800	5.05322400	-2.10126800
H	0.63232400	5.19067300	-3.17204700
H	0.06373200	5.60420900	-1.55073200
H	1.80331500	5.51030100	-1.88901300
C	3.63126100	4.04921000	0.18883100
H	4.43736900	4.46327200	0.80910300
H	3.86730000	3.00297400	-0.02410900
H	3.62930400	4.60353100	-0.75431000
C	2.45314900	3.56358400	2.33936500
H	3.21291400	4.12064800	2.90345600
H	1.52000000	3.60077000	2.91210800
H	2.75893800	2.51428000	2.28140100
C	1.94983200	5.67606500	1.10501600
H	2.78849300	6.18448700	1.59881200
H	1.77528800	6.17738600	0.15097800
H	1.06620200	5.82159000	1.73306800
O	0.91070800	-2.32053700	0.97457500
O	1.99765000	-1.36381000	2.74266300
O	4.19301700	-1.61025600	-0.63809100
O	3.01731500	-0.33044300	-2.15199700

VIII

Pd	-1.01294500	0.10371900	-0.42042500
P	-0.85574300	2.51152100	-0.35342200
P	-1.10198100	-2.26538200	-0.68690100
C	0.61792500	3.21544700	-1.23368200
C	-0.60394900	3.25601300	1.38568600
C	-2.23745200	3.42118900	-1.31555100
C	-1.72601300	-3.33952500	0.76597300
C	0.51732400	-3.11233000	-0.97181100
C	-2.02252600	-2.67136900	-2.30939400
H	1.09953600	-3.08759600	-0.05122800
H	0.36361700	-4.15177300	-1.26662300
H	1.09843800	-2.61250800	-1.74051600
H	0.63595900	4.30167400	-1.14599000
H	1.55134700	2.83477100	-0.82805500
H	0.56702700	2.96190900	-2.29467900
C	2.19413900	0.35553100	-0.62854300
C	0.96185900	0.09975900	-1.49665400
H	1.96418900	1.13251500	0.10150100
H	0.86332300	0.91800400	-2.21793700
H	1.12365600	-0.80001300	-2.09151000
C	2.65645700	-0.86523900	0.14694900
C	3.55511700	-1.78319100	-0.40602000
C	2.18011100	-1.09033200	1.44136200
C	3.96745600	-2.90121800	0.31670000
H	3.94882200	-1.59636500	-1.39876200
C	2.58738800	-2.20997000	2.16993600
H	1.48270500	-0.38565800	1.88288300
C	3.48359500	-3.12184900	1.60894300
H	4.67482700	-3.59734800	-0.12571000
H	2.20808600	-2.36537200	3.17637900
H	3.80803200	-3.99060300	2.17525400
N	3.32157500	0.85219800	-1.48762400
S	4.45393400	1.88651600	-0.79298900
O	3.82966500	2.76764000	0.21473700
O	5.18506400	2.48702300	-1.92007900

C	5.50981200	0.75254700	0.08059400
C	5.28527700	0.51825100	1.43675100
C	6.46843900	0.03369300	-0.63104200
C	6.01803600	-0.47404800	2.07868100
H	4.53602400	1.09388500	1.96655300
C	7.19706800	-0.95182200	0.02959800
H	6.63011900	0.23987800	-1.68345400
C	6.96871800	-1.23366700	1.38336500
H	5.83550500	-0.67684700	3.13016400
H	7.94464600	-1.52136700	-0.51573600
H	2.98738600	1.29591500	-2.34161200
C	-5.32810700	0.26543600	0.53495000
C	-4.74253000	0.12381000	1.99436700
B	-2.98004700	0.14967700	0.44130000
C	7.69774000	-2.35670900	2.07563100
H	7.03206500	-3.22062200	2.20154900
H	8.56847000	-2.68693900	1.50148600
H	8.03590100	-2.06050000	3.07450000
C	-5.31644300	-1.03496200	2.80928200
H	-4.80390600	-1.08542700	3.77524700
H	-6.38411200	-0.87878600	2.99957000
H	-5.19208200	-1.99634000	2.31153600
C	-4.85144700	1.40195300	2.83057400
H	-4.26651000	1.27733000	3.74683500
H	-4.46164800	2.27057700	2.30040700
H	-5.89098000	1.60358100	3.10883100
C	-6.26405800	1.45857400	0.33047500
H	-6.53087700	1.52685000	-0.72887400
H	-7.18699000	1.32251400	0.90499500
H	-5.81469600	2.40638500	0.62458800
C	-6.04948900	-0.98229400	0.01260700
H	-6.98887400	-1.14759900	0.55031900
H	-6.28105800	-0.83692000	-1.04686000
H	-5.44246700	-1.88220800	0.09998800
C	-1.19298300	-2.65836800	2.04222800
H	-1.47466000	-3.26342800	2.91490100
H	-0.10241400	-2.58035000	2.02666600
H	-1.61530400	-1.65953200	2.16350300
C	-3.25467100	-3.37073700	0.81299100
H	-3.66024500	-2.36410500	0.77736100
H	-3.68111400	-3.95777100	-0.00514600
H	-3.57937300	-3.82862300	1.75613000
C	-1.20895300	-4.79076000	0.73963000
H	-1.66958300	-5.33427400	1.57447800
H	-1.47024300	-5.31984000	-0.18035400
H	-0.12607500	-4.84699200	0.87400800
C	-3.41954900	-2.03416300	-2.32212100
H	-4.08982200	-2.48928000	-1.59197400
H	-3.37763400	-0.96470400	-2.11280200
H	-3.86644200	-2.17817600	-3.31496000
C	-2.15040600	-4.17658800	-2.59258800
H	-2.78578100	-4.68209100	-1.86005000
H	-2.61432100	-4.31512000	-3.57776200
H	-1.18155300	-4.68330500	-2.61717700
C	-1.20040800	-2.01415200	-3.43832200
H	-1.07683200	-0.94106600	-3.26341900
H	-0.20806600	-2.46127500	-3.54670300
H	-1.73148600	-2.15010300	-4.38890700
C	-1.75171700	2.87082400	2.32761900
H	-2.68055400	3.37907100	2.06792000
H	-1.93640400	1.79543600	2.32989900
H	-1.48897000	3.17464500	3.34941900
C	0.68342200	2.61857900	1.94421500
H	0.58740900	1.52925900	1.97956700
H	1.57759800	2.87443900	1.37081900
H	0.83668200	2.97438000	2.97098700
C	-0.44879900	4.78590100	1.38400600
H	-0.25760500	5.12258200	2.41094100
H	0.39180800	5.12337600	0.77218100
H	-1.35559800	5.29130100	1.03998300
C	-3.47864700	3.64364900	-0.44288500
H	-4.31677700	3.94979800	-1.08215600
H	-3.77273300	2.72975800	0.06617700
H	-3.32042000	4.43664600	0.29399800
C	-2.59831600	2.48787500	-2.48983800
H	-3.33296900	2.98786000	-3.13517200
H	-1.72090100	2.25291700	-3.10391900
H	-3.03086100	1.55270000	-2.13058100
C	-1.80548300	4.78296700	-1.89217900
H	-2.67855600	5.24625200	-2.36891600
H	-1.44610700	5.47282600	-1.12431600
H	-1.03146100	4.68637500	-2.65746000
O	-4.13597100	0.46014400	-0.27462200
O	-3.32676000	-0.12876300	1.76157000

IX

Pd	-1.68596300	-0.72959900	2.42759700
P	-2.63838300	1.29827700	3.04932400
P	-1.29093600	-2.85544900	1.60938000
C	-1.61327700	2.53899700	3.97154400
C	-4.10377400	1.01459200	4.23282900
C	-3.17003300	2.30590500	1.52078700
C	-2.64934900	-4.07456700	2.16746700
C	0.23984400	-3.77723900	2.12309200
C	-1.12536300	-2.84359600	-0.28920800
H	0.15745800	-4.04901500	3.17857500
H	0.40357500	-4.68752200	1.53802400
H	1.10800400	-3.12523100	2.02335100
H	-2.16037900	3.45552500	4.21087200
H	-1.26171700	2.10542700	4.90803900
H	-0.73494800	2.78943900	3.37012000
C	1.56700400	1.53413500	6.39435100
C	2.96583300	1.87216000	5.84976300
H	0.82513300	2.05580500	5.78127600
H	3.12855900	2.95736900	5.92639000
H	3.73051900	1.37500200	6.45578600
C	1.28865100	0.04395200	6.31804200
C	1.93653000	-0.85410200	7.17583300
C	0.38635400	-0.45137400	5.37572700
C	1.67008400	-2.21926300	7.10166200
H	2.62135900	-0.47136500	7.92569600
C	0.11678500	-1.81895500	5.29903400
H	-0.12041400	0.21791400	4.68825100
C	0.75415500	-2.70683000	6.16340500
H	2.16763000	-2.90259100	7.78427300
H	-0.59714200	-2.15764300	4.55537800
H	0.53841800	-3.77116000	6.11371400
N	1.47842600	2.00354600	7.79818700
S	-0.06788900	2.10248000	8.48126100
O	-1.08460200	2.37311600	7.44926800

O	0.08700900	3.02519600	9.61519300
C	-0.33515100	0.45089400	9.08490500
C	-1.21895200	-0.38982400	8.41204100
C	0.39413300	0.00737000	10.18785100
C	-1.35172200	-1.70640900	8.84076500
H	-1.76882800	-0.02303700	7.55610300
C	0.24558800	-1.31231400	10.60407800
H	1.06862700	0.68231800	10.70334300
C	-0.61438800	-2.19057900	9.92904600
H	-2.02578700	-2.37314100	8.31030000
H	0.81114100	-1.67043200	11.45999400
H	1.91042300	2.91779100	7.93168600
C	3.61240400	0.21999300	2.49233500
C	2.69743900	1.43214300	2.09192700
B	3.06806700	1.43002100	4.34125200
C	-0.71776400	-3.63825200	10.33677600
H	-0.10309700	-4.26630800	9.67839700
H	-0.36985000	-3.79436800	11.36231800
H	-1.74764400	-4.00280700	10.26265300
C	1.51664600	1.07331500	1.20161700
H	0.96711000	1.97980200	0.93525200
H	1.86475700	0.60786800	0.27267200
H	0.81512600	0.39364600	1.69722700
C	3.47688700	2.60876100	1.49756500
H	2.81231600	3.47518500	1.42750600
H	4.32903200	2.88298500	2.12753700
H	3.84694500	2.37598800	0.49411300
C	4.87393400	0.05928000	1.65268700
H	5.45197000	-0.79489600	2.01889000
H	4.61663100	-0.12893100	0.60449100
H	5.50887700	0.94641500	1.70293500
C	2.84885500	-1.10384300	2.56601400
H	2.57934900	-1.45272800	1.56442000
H	3.48524900	-1.85819300	3.03891500
H	1.93655100	-1.00821900	3.15950600
C	-2.79853800	-3.85793200	3.68798400
H	-3.61431000	-4.48728400	4.06771100
H	-1.88875200	-4.13608500	4.23153400
H	-3.02206300	-2.81175600	3.91723300
C	-3.97954400	-3.70238600	1.49335600
H	-4.19103900	-2.63292200	1.59809800
H	-3.97920300	-3.95224500	0.42826900
H	-4.79948600	-4.26145700	1.96287900
C	-2.33559300	-5.56035500	1.92514800
H	-3.15744500	-6.17064900	2.32239900
H	-2.22867800	-5.80261100	0.86657000
H	-1.42163700	-5.87248700	2.43989800
C	-2.20249000	-1.91613300	-0.88128300
H	-3.21319400	-2.30144100	-0.72698800
H	-2.15127300	-0.92370800	-0.42298000
H	-2.04280700	-1.81519200	-1.96329300
C	-1.19784800	-4.21800300	-0.96844600
H	-2.20040200	-4.65076400	-0.90757700
H	-0.95296300	-4.11180400	-2.03366200
H	-0.48705200	-4.93170400	-0.53900200
C	0.25021400	-2.21110600	-0.58190700
H	0.36343000	-1.25657800	-0.05912900
H	1.07647400	-2.86735800	-0.29125900
H	0.33978300	-2.02193100	-1.65938100
C	-4.97653900	-0.13211800	3.69099400
H	-5.48702100	0.13463900	2.76308900
H	-4.36719900	-1.01919200	3.49387100
H	-5.74343100	-0.38804100	4.43427500
C	-3.47979300	0.54001800	5.56160000
H	-2.78102300	-0.28793600	5.39371700
H	-2.95017700	1.34012400	6.08446900
H	-4.27796900	0.18134500	6.22481300
C	-4.97768300	2.24762900	4.49761300
H	-5.68845100	2.02441400	5.30424600
H	-4.38860200	3.11524200	4.81088700
H	-5.56266200	2.52550700	3.61608100
C	-4.31352400	1.58054000	0.79309700
H	-4.45329600	2.02211300	-0.20244000
H	-4.09064900	0.51533000	0.66747700
H	-5.26286500	1.67584200	1.32845200
C	-1.93473400	2.31550000	0.59836400
H	-2.18055600	2.83101100	-0.33961700
H	-1.09436700	2.84493600	1.05876500
H	-1.60522000	1.29740900	0.36897700
C	-3.56989900	3.76424900	1.79872500
H	-3.82531200	4.25355200	0.84938200
H	-4.43812200	3.84780900	2.45495400
H	-2.74882800	4.33240600	2.24628300
O	4.00351600	0.55799000	3.85708100
O	2.19306500	1.88206200	3.38560000

X

Pd	-0.88262300	-1.13489500	0.82580100
P	-0.79166600	-3.20036000	-0.01272800
C	0.46622500	-4.17718900	0.94161800
C	-0.22762800	-3.50430700	-1.81117700
C	-2.41511500	-4.13167600	0.34086800
H	0.40766700	-5.25371400	0.75236500
H	1.46413600	-3.81677000	0.67801600
H	0.31767900	-3.99303300	2.00769300
C	2.18890200	0.06863900	1.69033900
C	1.56695600	0.40220000	3.05583200
H	1.60041700	-0.74265200	1.23501900
H	1.52830000	-0.51626600	3.66326100
H	2.21788500	1.10318400	3.59357000
C	2.15716300	1.26359400	0.75152900
C	2.99740400	2.36612300	0.94177100
C	1.24451200	1.28273300	-0.30585700
C	2.91521600	3.46810200	0.09062100
H	3.73436400	2.34367100	1.73874700
C	1.15117300	2.38492900	-1.15504500
H	0.60166200	0.42120600	-0.46178500
C	1.98767900	3.48424100	-0.95620400
H	3.58025000	4.31433500	0.24057400
H	0.43606200	2.37052100	-1.97226000
H	1.92671200	4.34566300	-1.61616300
N	3.58086100	-0.37637900	1.94969700
S	4.34145200	-1.35329700	0.80135200
O	3.39526900	-2.30583600	0.19271000
O	5.53927200	-1.86289000	1.48390700
C	4.79762800	-0.16563400	-0.43686300
C	4.00897900	-0.02466800	-1.57736000
C	5.87958700	0.67875900	-0.18950300
C	4.29834200	1.00311200	-2.46894800
H	3.17410600	-0.69306800	-1.74814000

C	6.15641800	1.69743300	-1.09580200
H	6.48284700	0.54261300	0.70133300
C	5.35920100	1.88743000	-2.23340600
H	3.67520600	1.13523700	-3.34868200
H	6.99294400	2.36594000	-0.91167200
H	3.68017600	-0.85372400	2.84533800
O	-1.29553600	0.08696100	-2.05337500
H	-1.38206400	-0.39581000	-1.18418400
C	-1.96820800	1.67102400	2.29838500
C	-1.75906000	2.05040900	3.80063400
C	-3.08281000	2.97629600	-3.12007400
C	-4.08862300	2.33235200	-2.09552200
B	0.12111500	1.01266400	3.02188500
B	-2.19812900	1.07430700	-2.24646100
C	5.60476700	3.04483900	-3.16682700
H	5.50350000	2.74403700	-4.21501100
H	4.86936600	3.83953800	-2.98474900
H	6.60148400	3.47336000	-3.02572200
C	-4.68951100	3.30280500	-1.08698800
H	-5.28949100	4.06362500	-1.59804600
H	-5.34451400	2.76006500	-0.39801300
H	-3.91746600	3.80291300	-0.50018200
C	-5.19384200	1.51639300	-2.77226800
H	-5.72203400	0.93556300	-2.01006300
H	-5.91807100	2.16175800	-3.27914000
H	-4.77658600	0.81836700	-3.50463600
C	-3.70155000	3.38228600	-4.45251300
H	-4.48020200	4.13848000	-4.30341300
H	-2.93071900	3.81105200	-5.10044200
H	-4.13926500	2.52509300	-4.96809300
C	-2.28540100	4.14367300	-2.52801900
H	-1.48143900	4.40795000	-3.22155500
H	-2.91478300	5.02614400	-2.37629300
H	-1.82983600	3.87065900	-1.57155100
C	-2.42072400	1.06762100	4.76981500
H	-2.06558900	1.27540200	5.78340500
H	-3.50988400	1.16903500	4.75705000
H	-2.16531100	0.03280900	4.52119600
C	-2.13349500	3.48469100	4.15006200
H	-1.94729900	3.66471300	5.21317500
H	-1.54739000	4.20317300	3.57360900
H	-3.19700600	3.66061600	3.95551500
C	-1.69963700	2.82616300	1.33581000
H	-1.68534500	2.43417600	0.31881500
H	-2.48931800	3.58031800	1.40411200
H	-0.73658200	3.30267700	1.53925400
C	-3.28764800	0.98613400	1.98126900
H	-4.11989800	1.65187600	2.23905000
H	-3.34484700	0.76872400	0.91263700
H	-3.39740900	0.05329900	2.53803700
C	-1.37321200	-3.24025600	-2.80218700
H	-2.15470400	-4.00320900	-2.74403500
H	-1.82478100	-2.25699700	-2.64868800
H	-0.97115600	-3.26027200	-3.82360600
C	0.90235100	-2.49012300	-2.08373800
H	0.52605600	-1.46601400	-2.07181900
H	1.71384600	-2.57286100	-1.35644500
H	1.32062400	-2.68661700	-3.08079900
C	0.33483000	-4.91912200	-2.04105200
H	0.62061300	-5.01816300	-3.09627300
H	1.23257300	-5.09967300	-1.44346300
H	-0.38855800	-5.70708700	-1.82102400
C	-3.59278100	-3.29419200	-0.19128200
H	-4.54036100	-3.76667900	0.10071000
H	-3.56190500	-2.28231900	0.22699200
H	-3.58479500	-3.20652100	-1.28014500
C	-2.54239100	-4.20132000	1.87639700
H	-3.53997700	-4.57555300	2.14012800
H	-1.80544300	-4.87844600	2.31918400
H	-2.41546600	-3.20925700	2.32564400
C	-2.48222300	-5.55759900	-0.22395600
H	-3.39902500	-6.04724800	0.13023100
H	-2.50799000	-5.56721700	-1.31637100
H	-1.63616100	-6.16985700	0.10530100
O	-0.86200400	0.71194200	2.09228400
O	-0.31668200	1.89462700	3.96589500
O	-3.23955000	1.38428100	-1.39067500
O	-2.14170500	1.89562700	-3.35188600

VII′

Pd	1.19482600	0.72034400	0.74851300
P	0.94562300	3.14592900	0.08102400
C	-0.59000400	3.92683400	0.76676700
C	0.82416500	3.55422400	-1.77054200
C	2.29478000	4.18690500	0.93601100
H	-0.69382500	4.97593500	0.47738000
H	-1.47575800	3.39119400	0.42498900
H	-0.55776500	3.87224100	1.85880100
C	-1.78002400	0.58655100	0.69582600
C	-0.62846600	0.58557300	1.70530000
H	-1.54281200	1.27452200	-0.11858600
H	-0.64428300	1.51756300	2.28787600
H	-0.77758600	-0.23560800	2.41391700
C	-2.03751300	-0.78380100	0.09560700
C	-2.85875600	-1.71266800	0.73974700
C	-1.44874000	-1.13262300	-1.12193700
C	-3.10070400	-2.96201100	0.17018400
H	-3.33677700	-1.43456100	1.67249800
C	-1.68231700	-2.38342000	-1.69297500
H	-0.79343400	-0.42021000	-1.61563200
C	-2.51206600	-3.30324900	-1.04945300
H	-3.75769000	-3.66542900	0.67486600
H	-1.22057500	-2.63945900	-2.64217100
H	-2.70441700	-4.27442000	-1.49787100
N	-3.01004100	1.08118300	1.38728400
S	-4.19256500	1.83321000	0.45121200
O	-3.59159800	2.57810300	-0.67189300
O	-5.05145800	2.54130000	1.41283700
C	-5.06515300	0.44558100	-0.23732900
C	-4.73774800	0.00060200	-1.51736200
C	-5.98220200	-0.23962600	0.55776000
C	-5.32237700	-1.16843000	-1.99239400
H	-4.02250300	0.55324200	-2.11412600
C	-6.56264000	-1.40412800	0.06267000
H	-6.22729000	0.13164000	1.54682600
C	-6.22636800	-1.89539400	-1.20627700
H	-5.05725100	-1.53439500	-2.98015100
H	-7.27652300	-1.94844200	0.67483000
H	-2.79606100	1.71228900	2.15826000
O	3.43213000	0.59132300	0.02172700

H	3.86770700	0.35974300	0.85808200
C	1.04011300	-3.37227600	1.97150200
C	2.15369200	-2.80286600	2.91732900
C	3.11849900	-1.66404800	-2.74293800
C	4.30306300	-2.29972000	-1.92450800
B	1.34912100	-1.13517600	1.53497200
B	3.53684700	-0.43734700	-0.89607900
C	-6.78908600	-3.20160100	-1.70522500
H	-7.04021500	-3.15010400	-2.76993900
H	-6.04950600	-4.00413000	-1.58454500
H	-7.68777500	-3.49169300	-1.15268800
C	4.18080400	-3.79774700	-1.68926600
H	4.20869300	-4.33558800	-2.64305700
H	5.01807000	-4.14642300	-1.07675300
H	3.25062400	-4.04573400	-1.17670500
C	5.68319800	-1.95237800	-2.48614700
H	6.44710000	-2.25013100	-1.76181900
H	5.87525000	-2.47780300	-3.42652300
H	5.78005900	-0.87669400	-2.66363000
C	3.38363400	-1.50874600	-4.23411100
H	3.54599700	-2.48775500	-4.69767700
H	2.51594300	-1.04545600	-4.71369700
H	4.25579400	-0.88032100	-4.42608200
C	1.77709200	-2.35492700	-2.49667400
H	0.98752700	-1.74098100	-2.93813000
H	1.74821800	-3.34278900	-2.96777000
H	1.56157800	-2.46165700	-1.42985700
C	3.57377900	-3.15655800	2.46603100
H	4.28738000	-2.57685600	3.06096000
H	3.78902300	-4.21987600	2.61726100
H	3.72879600	-2.90815600	1.41323000
C	1.97247300	-3.13394200	4.39469900
H	2.78143000	-2.67965500	4.97593200
H	1.02360000	-2.75301600	4.77819600
H	2.00574600	-4.21768600	4.55587000
C	-0.32335000	-3.50765700	2.65452300
H	-1.07878000	-3.72091500	1.89497500
H	-0.32261800	-4.31613300	3.39314000
H	-0.60828000	-2.57739300	3.15503100
C	1.39334500	-4.67251800	1.26001800
H	1.51402300	-5.49059800	1.97918800
H	0.58517700	-4.93804800	0.57097700
H	2.31650800	-4.57908000	0.68563500
C	1.96385500	2.84520800	-2.52340300
H	2.94898100	3.23184400	-2.25402700
H	1.96253600	1.76868500	-2.33834600
H	1.83150900	3.00242100	-3.60247000
C	-0.50423000	2.93528700	-2.25183900
H	-0.54353600	1.86412100	-2.02793000
H	-1.38473400	3.40482800	-1.80763700
H	-0.57623700	3.05013600	-3.34115900
C	0.83272800	5.05322400	-2.10126800
H	0.63232400	5.19067300	-3.17204700
H	0.06373200	5.60420900	-1.55073200
H	1.80331500	5.51030100	-1.88901300
C	3.63126100	4.04921000	0.18883100
H	4.43736900	4.46327200	0.80910300
H	3.86730000	3.00297400	-0.02410900
H	3.62930400	4.60353100	-0.75431000
C	2.45314900	3.56358400	2.33936500
H	3.21291400	4.12064800	2.90345600
H	1.52000000	3.60077000	2.91210800
H	2.75893800	2.51428000	2.28140100
C	1.94983200	5.67606500	1.10501600
H	2.78849300	6.18448700	1.59881200
H	1.77528800	6.17738600	0.15097800
H	1.06620200	5.82159000	1.73306800
O	0.91070800	-2.32053700	0.97457500
O	1.99765000	-1.36381000	2.74266300
O	4.19301700	-1.61025600	-0.63809100
O	3.01731500	-0.33044300	-2.15199700

VIII′

Pd	-0.90835000	0.25336500	-0.23089700
P	-2.67016700	0.98728800	1.26789800
P	-0.96513200	-2.32559500	-1.03044100
C	-2.35740500	2.74823400	1.75551000
C	-2.76232900	0.19288700	3.00629200
C	-4.42168900	1.13731200	0.52183300
C	-1.29692300	-3.83485600	0.09687100
C	0.66520600	-2.85626900	-1.74502500
C	-2.05931900	-2.36921000	-2.59624000
H	1.41759000	-2.85290800	-0.95473700
H	0.61510400	-3.85339000	-2.18614900
H	0.99517600	-2.16181300	-2.51756800
H	-2.99406700	3.07439700	2.58099600
H	-1.30825000	2.87364100	2.01951900
H	-2.54841300	3.38993500	0.89535600
C	2.15537800	0.81424200	-0.33753600
C	0.99277500	0.52526900	-1.28938500
H	1.97579800	1.77070900	0.15564000
H	1.00171500	1.27547400	-2.09073400
H	1.16269700	-0.42982500	-1.78124100
C	2.32854700	-0.24713800	0.73146300
C	2.98832700	-1.45248300	0.46964600
C	1.84853900	-0.01042100	2.02205000
C	3.16031500	-2.40312800	1.47477300
H	3.40828600	-1.61634400	-0.51652100
C	2.01042600	-0.96231700	3.03120300
H	1.35672800	0.93483100	2.23199900
C	2.66876900	-2.16275900	2.76058700
H	3.69364800	-3.32502700	1.25897200
H	1.63449400	-0.75927100	4.03028100
H	2.80815900	-2.90011900	3.54642700
N	3.41320100	0.91666100	-1.14466100
S	4.71403300	1.76207000	-0.47738200
O	4.26353400	2.83492400	0.42772800
O	5.58079100	2.08999200	-1.62098800
C	5.50121600	0.52270200	0.53082000
C	5.31131600	0.54399600	1.91154100
C	6.23445600	-0.48987900	-0.08515700
C	5.84515700	-0.48480400	2.68018700
H	4.73650200	1.34344000	2.36244300
C	6.76529500	-1.50953500	0.70054400
H	6.37765000	-0.48024900	-1.16022700
C	6.56572600	-1.53076300	2.08767600
H	5.68591700	-0.48590400	3.75483900
H	7.33553800	-2.30620300	0.23054100
H	3.24196100	1.32656600	-2.06216000
C	-0.84224300	4.12409800	-1.97728900

C	0.29640100	4.30619600	-0.89902200
B	-0.58695900	2.17358200	-0.74318200
C	7.08345300	-2.66969900	2.92912900
H	6.25852900	-3.32497900	3.23777900
H	7.80665600	-3.28000700	2.38015500
H	7.56536100	-2.30565300	3.84311200
C	0.16083100	5.54643000	-0.02026000
H	0.97357300	5.55914600	0.71254900
H	0.23022400	6.46050100	-0.62015000
H	-0.78683500	5.55505900	0.52262500
C	1.71094000	4.24679300	-1.48262900
H	2.43919200	4.19628700	-0.66970000
H	1.84838100	3.35931700	-2.10629600
H	1.92686300	5.12874500	-2.09399400
C	-0.41933400	4.43817300	-3.41114600
H	-1.26188700	4.25707700	-4.08622400
H	-0.12144200	5.48768200	-3.51183300
H	0.41206700	3.80740400	-3.73183000
C	-2.13110700	4.88821800	-1.65866900
H	-1.99146900	5.96899200	-1.76437000
H	-2.91224600	4.57224200	-2.35684800
H	-2.48344600	4.68816300	-0.64420800
C	-0.40169100	-3.58424300	1.32990200
H	-0.63740300	-4.31526700	2.11452000
H	0.65725800	-3.69381900	1.08114300
H	-0.52247800	-2.58251300	1.74650000
C	-2.78191600	-3.87524200	0.49558500
H	-3.18844300	-2.88454900	0.70387400
H	-3.38631300	-4.32809400	-0.29619900
H	-2.90994000	-4.48718900	1.39774300
C	-0.91568700	-5.21447400	-0.47042300
H	-1.16231300	-5.98180100	0.27578000
H	-1.45796400	-5.46226900	-1.38536000
H	0.15550900	-5.29633900	-0.67205500
C	-3.51111500	-2.02922400	-2.23265600
H	-3.99179800	-2.81599300	-1.64750100
H	-3.56206000	-1.10032900	-1.66505500
H	-4.09726700	-1.89390800	-3.15113800
C	-2.03410100	-3.68915800	-3.37902300
H	-2.53637300	-4.49382200	-2.83493200
H	-2.56903700	-3.55649700	-4.32894700
H	-1.01823200	-4.01581300	-3.61913100
C	-1.52691800	-1.23173900	-3.49661200
H	-1.48699700	-0.27913500	-2.95731900
H	-0.52630300	-1.44384800	-3.88532700
H	-2.19630900	-1.11374300	-4.35838700
C	-2.93767700	-1.32465000	2.89014100
H	-3.91967000	-1.60550100	2.50487700
H	-2.17906700	-1.74866700	2.23563900
H	-2.82593600	-1.78538000	3.88041300
C	-1.38669100	0.45189800	3.65173600
H	-0.58041100	0.08136600	3.01484300
H	-1.20990900	1.51251300	3.85267400
H	-1.33367700	-0.08118900	4.60961200
C	-3.86300800	0.74915600	3.92267900
H	-3.73268200	0.32723500	4.92792900
H	-3.82761500	1.83758600	4.02117500
H	-4.86144800	0.46548900	3.57988800
C	-5.13379300	-0.22416100	0.56293900
H	-6.01352800	-0.19815800	-0.09277200
H	-4.49026800	-1.03854800	0.22287200
H	-5.48368200	-0.46436500	1.57104500
C	-4.21519500	1.57387200	-0.94248500
H	-5.18927400	1.62199500	-1.44717000
H	-3.75172500	2.56093300	-1.00793500
H	-3.56872100	0.88801600	-1.49273200
C	-5.31756000	2.19112200	1.19778400
H	-6.29421100	2.19720500	0.69621100
H	-5.49276400	1.98727700	2.25532900
H	-4.90152300	3.19839000	1.10796900
O	-1.14204200	2.70503100	-1.90059300
O	0.13044400	3.13555900	-0.05284100

TSIII´-IX

Pd	-1.05164300	0.25001000	0.15198000
P	-2.61122400	1.15279200	1.73093700
P	-1.31341000	-2.27543600	-0.68997400
C	-2.09125500	2.86355300	2.22862400
C	-2.69439600	0.32808700	3.45479600
C	-4.37670300	1.49318200	1.08749500
C	-1.79699600	-3.77805900	0.38830400
C	0.24067100	-2.96598000	-1.43939800
C	-2.42160800	-2.16599600	-2.24143900
H	0.92229800	-3.24922000	-0.63680200
H	0.03806300	-3.84015100	-2.06258900
H	0.75179900	-2.22281300	-2.05165600
H	-2.62960400	3.22948600	3.10673300
H	-1.01788700	2.88120900	2.41430300
H	-2.27738400	3.54397200	1.39707700
C	2.08024700	0.60491500	-0.13798700
C	0.83724400	0.51411800	-1.02552600
H	2.04195800	1.54920200	0.40875000
H	0.92974200	1.25641000	-1.82719900
H	0.81256500	-0.44259200	-1.54101200
C	2.19205300	-0.52247200	0.87066400
C	2.71882700	-1.77014100	0.52361400
C	1.79581900	-0.30401400	2.19268400
C	2.84211400	-2.78067300	1.47598600
H	3.07051000	-1.92741900	-0.48954100
C	1.90686600	-1.31564000	3.14896300
H	1.41296400	0.67337400	2.47071000
C	2.43318500	-2.55836400	2.79335300
H	3.27362300	-3.73720100	1.19340600
H	1.59702500	-1.12705000	4.17313300
H	2.53520600	-3.34305800	3.53794700
N	3.28793100	0.60706400	-1.02057500
S	4.70715800	1.29292100	-0.41168700
O	4.42121200	2.36825600	0.55456100
O	5.52969800	1.57606800	-1.59866500
C	5.42056500	-0.06044000	0.49906100
C	5.30477800	-0.08809200	1.88740000
C	6.01063800	-1.11144100	-0.20141200
C	5.76800300	-1.20297900	2.57909800
H	4.83892200	0.74141100	2.40491200
C	6.47152900	-2.21719800	0.50724300
H	6.09838200	-1.06271100	-1.28135900
C	6.34198500	-2.28670200	1.90160100
H	5.66548000	-1.24074800	3.65987800
H	6.93064700	-3.04361200	-0.02863500
H	3.10861700	1.05960700	-1.91597500

C	-0.69217800	4.07476600	-1.65672500
C	0.47620000	4.19047300	-0.59845700
B	-0.48431900	2.08613800	-0.46473700
C	6.77183600	-3.51846200	2.65719200
H	7.21882900	-3.26118500	3.62311400
H	5.90678100	-4.16345500	2.86118700
H	7.49698100	-4.10796500	2.08799500
C	0.41305600	5.42332400	0.29948100
H	1.24170100	5.39155800	1.01384900
H	0.50777500	6.34208900	-0.28970500
H	-0.52031300	5.46490500	0.86469300
C	1.87664200	4.08516700	-1.21172700
H	2.61610900	3.98179900	-0.41388600
H	1.96830900	3.21072900	-1.86109200
H	2.12076600	4.97251100	-1.80443200
C	-0.28247800	4.39054600	-3.09473700
H	-1.14516500	4.25688000	-3.75539800
H	0.06031900	5.42699500	-3.18767300
H	0.51338100	3.72819800	-3.44128700
C	-1.93150600	4.90215600	-1.30218200
H	-1.73726600	5.97497900	-1.40250600
H	-2.74461000	4.63370300	-1.98325200
H	-2.26912500	4.71128200	-0.28132500
C	-0.87339600	-3.67812300	1.62326700
H	-1.20860700	-4.38339100	2.39502700
H	0.15924700	-3.93158200	1.36783300
H	-0.84997500	-2.67750200	2.05886600
C	-3.27367800	-3.66461900	0.80084200
H	-3.54224400	-2.65560700	1.11823300
H	-3.93529700	-3.94681900	-0.02412600
H	-3.48080300	-4.34539700	1.63669100
C	-1.57143200	-5.16752800	-0.23511700
H	-1.84782700	-5.93385800	0.50164900
H	-2.17937300	-5.33578200	-1.12553000
H	-0.52374200	-5.33584400	-0.49793300
C	-3.77496200	-1.55041400	-1.85923500
H	-4.36343200	-2.20476600	-1.21170100
H	-3.63117900	-0.59977200	-1.34570500
H	-4.36430700	-1.36215400	-2.76639300
C	-2.66623700	-3.48730500	-2.98425100
H	-3.30111300	-4.16583700	-2.40766000
H	-3.18753400	-3.27734100	-3.92777900
H	-1.73879100	-4.01120500	-3.23464100
C	-1.70905700	-1.17741600	-3.19051000
H	-1.48480800	-0.23025000	-2.68873300
H	-0.77575800	-1.58797100	-3.58772200
H	-2.36523600	-0.96343700	-4.04388900
C	-3.01352500	-1.16389900	3.31251600
H	-4.04033700	-1.34404400	2.98772800
H	-2.34287900	-1.63255900	2.59518800
H	-2.88034600	-1.66430600	4.28065600
C	-1.27188700	0.44782100	4.03612100
H	-0.53294900	0.02872600	3.34977700
H	-0.99517100	1.48378400	4.25281600
H	-1.21805100	-0.11467600	4.97711400
C	-3.69320300	0.95660200	4.43776900
H	-3.54767300	0.50503600	5.42797500
H	-3.55847200	2.03597100	4.54958600
H	-4.72869500	0.76576700	4.14419900
C	-5.19978800	0.19502200	1.09490500
H	-6.11546400	0.33935100	0.50742700
H	-4.65187300	-0.64215900	0.65612400
H	-5.50131600	-0.08478200	2.10829800
C	-4.20743900	1.98661800	-0.36212400
H	-5.19730800	2.11557200	-0.81949900
H	-3.69267500	2.94823400	-0.40183700
H	-3.62605800	1.29364900	-0.97078300
C	-5.14619400	2.58265300	1.85666100
H	-6.12368800	2.72679200	1.37792000
H	-5.32752500	2.32204900	2.90015700
H	-4.62757700	3.54485700	1.82974100
O	-1.05735600	2.67276000	-1.59472100
O	0.27727200	3.01763900	0.23187000

TSVII´-IX

Pd	1.37729700	0.80402800	0.55351700
P	1.37255000	3.03239700	-0.45576300
C	0.14069500	4.12211400	0.40128300
C	0.85662000	3.18942400	-2.28363100
C	3.00411200	3.96012600	-0.13591900
H	0.04348100	5.11027300	-0.05903300
H	-0.84061300	3.64233800	0.40565900
H	0.45633100	4.25088300	1.44078900
C	-1.58265200	0.52916500	0.74225700
C	-0.40953100	0.35247100	1.71232000
H	-1.34460900	1.34952000	0.06407000
H	-0.28531800	1.24960100	2.33272100
H	-0.69291000	-0.44230300	2.41699700
C	-1.86081700	-0.72023400	-0.07801300
C	-2.79317600	-1.67317200	0.33631400
C	-1.16171900	-0.93341200	-1.26970500
C	-3.04048300	-2.80827800	-0.43665500
H	-3.34960900	-1.50228700	1.25069500
C	-1.40597000	-2.06402300	-2.04620000
H	-0.40453300	-0.21635300	-1.57318900
C	-2.35086900	-3.00666200	-1.63408400
H	-3.78168300	-3.53164400	-0.10744900
H	-0.85859900	-2.21042800	-2.97286500
H	-2.54769300	-3.88648400	-2.24104200
N	-2.79114500	0.91257300	1.53473200
S	-3.88898200	1.96299900	0.79642200
O	-3.19296600	2.99056700	-0.00163000
O	-4.80755600	2.37938900	1.86548100
C	-4.71178500	0.87119800	-0.33745500
C	-4.23645900	0.76760300	-1.64428200
C	-5.73061400	0.04489800	0.13351700
C	-4.77338500	-0.20632800	-2.47909500
H	-3.45004500	1.42890300	-1.98819400
C	-6.26122000	-0.91886900	-0.71902500
H	-6.08847200	0.15191100	1.15169600
C	-5.77404500	-1.07556600	-2.02410500
H	-4.39476500	-0.30930100	-3.49203100
H	-7.05199900	-1.57263000	-0.36158900
H	-2.54924500	1.32459700	2.43593300
O	3.79619100	0.13527000	0.36820400
H	3.91263600	-0.30386100	1.22551300
C	1.02241100	-3.20202600	1.87497000
C	1.93766200	-2.55534800	2.98298300
C	3.49709000	-1.55392800	-2.75682100
C	4.36199200	-2.57102800	-1.92784800

B	1.08391100	-0.91849000	1.58067800
B	3.86141300	-0.74440100	-0.68427600
C	-6.27801100	-2.18492500	-2.91059900
H	-6.38328300	-1.85393700	-3.94910800
H	-5.56665600	-3.02129600	-2.90569300
H	-7.24480800	-2.56753800	-2.57008400
C	3.88585000	-4.01543600	-1.99243400
H	3.95171500	-4.39170200	-3.01910700
H	4.52200200	-4.64281000	-1.36006500
H	2.85533900	-4.11558000	-1.64902500
C	5.85770100	-2.48190900	-2.23738900
H	6.41186500	-3.05269500	-1.48635100
H	6.08560800	-2.89655600	-3.22415500
H	6.20602200	-1.44486700	-2.20679900
C	4.00531900	-1.26943500	-4.16313200
H	4.00029600	-2.18331800	-4.76698600
H	3.35098000	-0.53749000	-4.64669800
H	5.01910500	-0.86367400	-4.14944800
C	2.01202100	-1.92289600	-2.77630800
H	1.44786300	-1.08770600	-3.20035500
H	1.83070000	-2.80759600	-3.39488200
H	1.63012400	-2.11179400	-1.76874000
C	3.43512300	-2.76992100	2.74198500
H	3.99663100	-2.12223600	3.42398000
H	3.72859900	-3.80611500	2.94151100
H	3.71900100	-2.53526600	1.71317700
C	1.58588400	-2.95604900	4.41320600
H	2.26046700	-2.45145700	5.11235200
H	0.56173700	-2.67583200	4.66841100
H	1.69806200	-4.03719700	4.55273000
C	-0.37964700	-3.56714400	2.37313600
H	-1.00350600	-3.81665900	1.51083700
H	-0.35464100	-4.42344000	3.05511200
H	-0.85353600	-2.72803700	2.89155100
C	1.64226400	-4.38898100	1.14745300
H	1.82961900	-5.21774500	1.83954800
H	0.95159100	-4.74028800	0.37372100
H	2.58088800	-4.11043300	0.66728000
C	1.60605300	2.11867400	-3.09520500
H	2.68413700	2.28817000	-3.12394300
H	1.44924600	1.12485200	-2.66890600
H	1.23549500	2.11783100	-4.12952500
C	-0.64844200	2.86112400	-2.34979600
H	-0.86571000	1.88459300	-1.90920000
H	-1.26940400	3.60292400	-1.84284400
H	-0.95865000	2.82500000	-3.40235700
C	1.08185200	4.57377600	-2.90739100
H	0.64474800	4.59349000	-3.91459300
H	0.60434100	5.37028300	-2.32747000
H	2.14441700	4.80985700	-3.00894700
C	4.08697000	3.43854500	-1.09518700
H	5.06907300	3.81010900	-0.77404300
H	4.12421200	2.34515100	-1.09940000
H	3.92577600	3.78426500	-2.12027200
C	3.41314500	3.57872800	1.30380800
H	4.35539900	4.08297400	1.55713400
H	2.66381700	3.88894300	2.04032600
H	3.55994500	2.50003700	1.40224400
C	2.91399400	5.49276900	-0.21729100
H	3.89639400	5.92344400	0.01814500
H	2.62750300	5.84807500	-1.20828200
H	2.20017100	5.89583900	0.50770500
O	0.85627000	-2.11862400	0.92320600
O	1.67400900	-1.13442500	2.82939800
O	4.18468600	-2.07368700	-0.56383200
O	3.61970800	-0.33365400	-1.96632400

TSII-IIIw

Pd	0.46242700	-1.90761900	-0.75722900
P	2.37923000	-1.69657900	0.57340000
P	-1.40301900	-2.53099400	-2.03173100
C	2.07475600	-2.14091100	2.39967500
C	3.13357900	-0.01009500	0.71405200
C	3.81034000	-2.71567700	-0.16127600
C	-2.80106800	-3.25503700	-0.95771800
C	-0.93236900	-3.71681400	-3.44250500
C	-2.27606800	-1.17015800	-2.93714200
H	-1.54424400	-0.55597500	-3.46215900
H	-2.79487100	-0.52714600	-2.22077000
H	-3.01189900	-1.54958200	-3.65150600
H	4.10548100	-0.02800700	1.21535500
H	3.26304700	0.41218800	-0.28506100
H	2.45956700	0.64541800	1.27139100
C	0.26033500	1.33950000	-0.91518200
C	-0.47938700	0.31074200	-0.15585800
H	1.28630500	1.46498900	-0.56807800
H	-0.19566000	0.11881800	0.86515900
H	-1.45285100	0.00710400	-0.49813000
C	0.16805000	1.38532100	-2.41215500
C	-0.73584300	2.21921500	-3.07323300
C	1.01026600	0.55175500	-3.15895000
C	-0.81306500	2.20197900	-4.46825500
H	-1.37543900	2.87877900	-2.49891500
C	0.93367000	0.53598800	-4.55057700
H	1.70446300	-0.10274400	-2.63869200
C	0.01554100	1.35847900	-5.20995700
H	-1.52311800	2.85068000	-4.97326000
H	1.58408600	-0.12182100	-5.11991000
H	-0.04823200	1.34472300	-6.29433300
N	-0.73637700	2.12149300	-0.18415400
S	-0.13266900	2.94966600	1.12638000
O	0.81634100	2.13597000	1.92268400
O	-1.29478500	3.53369000	1.81881100
C	0.80575300	4.24915600	0.33780700
C	2.18215300	4.32749300	0.53215400
C	0.13774100	5.13863800	-0.51185800
C	2.90291200	5.32081900	-0.13309700
H	2.67302400	3.62339900	1.19445500
C	0.87510500	6.11834600	-1.16572900
H	-0.93326900	5.04402500	-0.66731200
C	2.26485300	6.22621200	-0.98793000
H	3.97728600	5.39067300	0.01412600
H	0.36700900	6.81156700	-1.83114400
C	-4.16994800	-3.33048400	-1.65446200
H	-4.16556000	-3.98680100	-2.52623000
H	-4.90970800	-3.72591300	-0.94639100
H	-4.51950400	-2.34347700	-1.97135100
C	-2.39585700	-4.64548800	-0.44259100
H	-1.39275000	-4.63272000	-0.00146000
H	-3.10274200	-4.96641000	0.33316200

H	-2.41654200	-5.39866600	-1.23557400
C	-2.93084100	-2.31177600	0.25616000
H	-3.70150000	-2.69865900	0.93492700
H	-1.98765400	-2.23949500	0.80626300
H	-3.23579000	-1.30079900	-0.03581600
C	3.31744300	-4.13455900	-0.49601400
H	2.41841900	-4.09623400	-1.11709200
H	4.09843900	-4.66932000	-1.05209500
H	3.08444900	-4.71916300	0.39597300
C	4.17242000	-2.02332500	-1.49195200
H	4.89912700	-2.64142900	-2.03394600
H	3.28685400	-1.90633700	-2.12745400
H	4.62437600	-1.03866700	-1.33996100
C	5.06205500	-2.80217400	0.72251700
H	5.87037300	-3.28904200	0.16158300
H	5.42450200	-1.81598300	1.02917900
H	4.88332500	-3.39855900	1.62131700
C	1.86235900	-3.65659100	2.53605400
H	1.47229800	-3.88093800	3.53696700
H	1.13830300	-4.02349400	1.79975800
H	2.79521500	-4.21470500	2.41552000
C	3.18045600	-1.67365000	3.36144700
H	2.90979000	-1.96636400	4.38404100
H	4.15318900	-2.11699500	3.13938000
H	3.28905500	-0.58534300	3.35425300
C	0.76326300	-1.43186200	2.79577600
H	-0.07316800	-1.78233700	2.18250700
H	0.53631800	-1.65768800	3.84571200
H	0.83285500	-0.34313100	2.70357600
C	-0.01099400	-4.82116200	-2.89656700
H	0.35300400	-5.43674500	-3.72950000
H	0.85288300	-4.38574400	-2.38723000
H	-0.51876100	-5.48081200	-2.19005200
C	-2.12249700	-4.35239500	-4.17454900
H	-1.75641000	-4.90405300	-5.05013700
H	-2.65599900	-5.06454000	-3.53930200
H	-2.83814300	-3.60555000	-4.53269300
C	-0.11392100	-2.86966400	-4.43731200
H	0.70788900	-2.35262600	-3.93181100
H	0.31318700	-3.52792700	-5.20448400
H	-0.72462300	-2.11694400	-4.94300400
C	3.04242900	7.29910800	-1.70862700
H	2.88740100	7.23847400	-2.79230800
H	2.71927900	8.29860100	-1.39303100
H	4.11558300	7.21530300	-1.51411100
O	-3.00683200	3.82563900	-0.61710400
H	-2.37517300	3.07727100	-0.63839200
H	-2.90907000	4.10389900	0.30926700

TSII-IIIw2

Pd	0.34752100	-1.85881900	-0.76784800
P	2.19952900	-1.92334800	0.65187100
P	-1.46129100	-2.45617500	-2.10680200
C	1.96673700	-3.38457700	1.84717800
C	2.54630500	-0.53511700	1.82085800
C	3.83346700	-2.06616600	-0.31438300
C	-2.93220300	-3.11285400	-1.08863700
C	-0.90458500	-3.72715700	-3.40843000
C	-2.26806500	-1.13776500	-3.12946400
H	-1.50035300	-0.56537300	-3.64987200
H	-2.80109600	-0.44679100	-2.47002500
H	-2.98087300	-1.53775500	-3.85640600
H	3.53007700	-0.62535600	2.29145600
H	2.48053100	0.42428600	1.30553600
H	1.77516000	-0.53756100	2.59252700
C	0.30367300	1.37566800	-0.91933900
C	-0.48056800	0.41514900	-0.12082300
H	1.33445500	1.47257300	-0.57074600
H	-0.20529100	0.18593200	0.90070800
H	-1.44546100	0.10990900	-0.49037300
C	0.19746800	1.40356400	-2.41667500
C	-0.67201600	2.28308800	-3.06444400
C	0.98217000	0.52747000	-3.17623000
C	-0.76488100	2.28015400	-4.45831000
H	-1.26977800	2.96249800	-2.46717500
C	0.89216400	0.52616000	-4.56747500
H	1.63359500	-0.17661400	-2.66728800
C	0.01475900	1.40200700	-5.21325300
H	-1.44629200	2.96620000	-4.95374600
H	1.49831100	-0.16443600	-5.14653500
H	-0.05835000	1.39982200	-6.29714100
N	-0.68254700	2.19626400	-0.20477400
S	-0.05185200	3.12378100	1.01425100
O	0.97424400	2.41230300	1.81869200
O	-1.20254600	3.70589900	1.73905800
C	0.77695800	4.41929300	0.11707200
C	2.16124500	4.37297800	-0.03821100
C	0.01086300	5.41163200	-0.50109400
C	2.78428200	5.33899600	-0.82853900
H	2.73632600	3.60358500	0.46441600
C	0.65061100	6.36681700	-1.28202800
H	-1.06427900	5.43342600	-0.36060800
C	2.04331900	6.34199400	-1.46421600
H	3.86356100	5.31320100	-0.95012900
H	0.06359700	7.14651400	-1.76006000
C	-4.25690500	-3.20092500	-1.86500000
H	-4.20936400	-3.90160300	-2.70081300
H	-5.04757700	-3.54743000	-1.18695200
H	-4.56640800	-2.22619200	-2.25405500
C	-2.58949100	-4.48419200	-0.48428700
H	-1.62250300	-4.46724200	0.02941500
H	-3.35615800	-4.75871800	0.25141800
H	-2.56560600	-5.27177800	-1.24272100
C	-3.11330000	-2.11049100	0.07024700
H	-3.94321100	-2.43631300	0.70984900
H	-2.21011100	-2.04079500	0.68368900
H	-3.36285000	-1.10720400	-0.29457200
C	3.61098000	-2.97386200	-1.53774800
H	2.78119800	-2.60574500	-2.15074500
H	4.52012100	-2.99330200	-2.15372200
H	3.37932300	-4.00312800	-1.25148800
C	4.15745400	-0.64482400	-0.81881200
H	4.99207400	-0.69327100	-1.52963000
H	3.30461700	-0.19403000	-1.33626500
H	4.45200200	0.02196900	-0.00286700
C	5.02816600	-2.57916800	0.50225500
H	5.94146200	-2.50807200	-0.10314100
H	5.18941900	-1.98900500	1.41018200
H	4.90737300	-3.62681800	0.79069100
C	2.07235000	-4.71106000	1.07766900

H 1.75659700 -5.53587700 1.72974300
H 1.42144900 -4.70868400 0.19647500
H 3.09508000 -4.92333800 0.75176300
C 2.92929800 -3.38963900 3.04638100
H 2.69673200 -4.25052400 3.68643300
H 3.97631600 -3.47342100 2.74876200
H 2.81654500 -2.48982700 3.65796700
C 0.52287600 -3.24569000 2.37742100
H -0.19745100 -3.35730000 1.56098500
H 0.33460100 -4.03165800 3.12119600
H 0.33698000 -2.27559300 2.84830800
C -0.01000200 -4.77925700 -2.72731600
H 0.40763100 -5.45114700 -3.48886300
H 0.81733700 -4.29400600 -2.19971600
H -0.55532700 -5.39109800 -2.00546700
C -2.04548100 -4.41877100 -4.16600600
H -1.62682000 -5.03815000 -4.97003100
H -2.62679700 -5.07716600 -3.51509100
H -2.72943600 -3.69991400 -4.62865300
C -0.02675500 -2.95051100 -4.40923700
H 0.77543100 -2.41326600 -3.89373000
H 0.43182000 -3.65967600 -5.11013300
H -0.59994800 -2.22587500 -4.99434100
C 2.71568500 7.37097400 -2.33794400
H 2.47816800 7.20024400 -3.39552800
H 2.37792600 8.38354500 -2.09072800
H 3.80370000 7.33778800 -2.23087500
O -2.76455800 1.38779000 2.18976100
H -2.68028300 1.18968700 1.24362600
H -2.40379300 2.29797400 2.23301900
O -0.39864300 0.19556800 3.15259000
H -1.30544100 0.49548700 2.91765400
H 0.14966100 0.97334700 2.94316300

TSII-IIIw3
Pd 0.37271400 -1.89831800 -0.76699500
P 2.28393900 -1.70361000 0.56709200
P -1.46605100 -2.48799200 -2.09366500
C 1.97191200 -2.11644600 2.39780500
C 3.06065100 -0.02282100 0.69194200
C 3.70907900 -2.74291100 -0.15197600
C -2.93029900 -3.15362600 -1.07504900
C -0.96782900 -3.70312900 -3.47046500
C -2.26994900 -1.12006800 -3.05367200
H -1.50884000 -0.53702300 -3.57157400
H -2.79078400 -0.44901600 -2.36485900
H -2.99682100 -1.49241100 -3.78081600
H 4.03069300 -0.04508800 1.19693300
H 3.19876800 0.38941500 -0.31040600
H 2.39221500 0.64389100 1.24244200
C 0.25161700 1.38456700 -0.96709700
C -0.54580400 0.41732700 -0.19707100
H 1.26508600 1.50738200 -0.58820800
H -0.31089000 0.18522500 0.82746600
H -1.48857100 0.07979200 -0.58964500
C 0.18729900 1.40786500 -2.46325400
C -0.71997400 2.21902200 -3.14629000
C 1.04580100 0.56891600 -3.18437900
C -0.77977900 2.18266000 -4.54140100
H -1.37629900 2.86974400 -2.57884600
C 0.98484600 0.53219000 -4.57659000
H 1.73584900 -0.07481200 -2.64618100
C 0.06813400 1.33723700 -5.25919200
H -1.49104500 2.81416600 -5.06623000
H 1.64579100 -0.13007900 -5.12809800
H 0.01822000 1.30653900 -6.34387700
N -0.76888700 2.18128400 -0.26678500
S -0.20361000 3.03864400 1.03252500
O 0.77930800 2.27556400 1.84441000
O -1.39151400 3.57350700 1.73656600
C 0.66973700 4.38955300 0.27235600
C 2.06088800 4.35355800 0.19253600
C -0.06553400 5.42472400 -0.31211700
C 2.72282200 5.37785000 -0.48424900
H 2.61058500 3.54612400 0.66305200
C 0.61329800 6.43734600 -0.97898400
H -1.14739700 5.43379800 -0.23669100
C 2.01461100 6.42934800 -1.07811800
H 3.80710100 5.35989500 -0.54894800
H 0.05037900 7.24843700 -1.43284100
C -4.26524400 -3.21854200 -1.83610400
H -4.23956900 -3.90857500 -2.68153500
H -5.04864600 -3.56476700 -1.14962100
H -4.57090200 -2.23499200 -2.20534600
C -2.58412500 -4.53916200 -0.50668900
H -1.60306300 -4.53780100 -0.01866500
H -3.33352100 -4.82076100 0.24394700
H -2.58758800 -5.31286500 -1.28034400
C -3.09233300 -2.17403800 0.10362400
H -3.90591100 -2.51946800 0.75375700
H -2.18549100 -2.09788500 0.70777300
H -3.35889000 -1.16471300 -0.22784600
C 3.20182600 -4.16070000 -0.46821600
H 2.30909300 -4.12014500 -1.09771000
H 3.98101000 -4.71508200 -1.00769000
H 2.95260900 -4.72635900 0.43170300
C 4.08075300 -2.07228500 -1.49095300
H 4.79899500 -2.70658500 -2.02570100
H 3.19598100 -1.95128800 -2.12679400
H 4.54647900 -1.09201300 -1.35079100
C 4.95834300 -2.83268600 0.73484700
H 5.76555300 -3.32603500 0.17779900
H 5.32559000 -1.84777500 1.04013500
H 4.77345800 -3.42537700 1.63476100
C 1.70159200 -3.62159800 2.54976100
H 1.29722300 -3.81868300 3.55066300
H 0.96720900 -3.96847800 1.81433300
H 2.61328800 -4.21687600 2.44122600
C 3.09427200 -1.68348200 3.35618600
H 2.80155800 -1.93843700 4.38280700
H 4.04548200 -2.17949400 3.15308900
H 3.25791200 -0.60214600 3.32675800
C 0.68937700 -1.35309100 2.78103400
H -0.16907200 -1.65757900 2.17845300
H 0.45158300 -1.55099200 3.83400700
H 0.80564900 -0.27030300 2.67366200
C -0.11952900 -4.83671400 -2.86850000
H 0.26503000 -5.47320900 -3.67632600
H 0.73110100 -4.42771400 -2.31714000
H -0.68816600 -5.47090700 -2.18529800

C	-2.14087200	-4.30300100	-4.25830300
H	-1.75148800	-4.86837400	-5.11511700
H	-2.72452800	-4.99648600	-3.64697500
H	-2.81700400	-3.53652700	-4.65008800
C	-0.06336500	-2.90143800	-4.42753800
H	0.75343100	-2.41792400	-3.88162800
H	0.37387000	-3.58495700	-5.16670600
H	-0.61199000	-2.12747100	-4.97142500
C	2.73333300	7.54405400	-1.79547000
H	2.69802800	8.47164700	-1.21040900
H	3.78506300	7.29746200	-1.96657300
H	2.26841600	7.75583300	-2.76462500
O	-3.52073500	1.90371800	0.57857100
H	-2.71190600	1.97343000	0.03284300
H	-3.37804600	2.63512600	1.20323200
O	-2.26459500	0.01570100	2.38099900
H	-2.77571200	0.60195400	1.78693200
H	-1.89311900	0.61163800	3.06454900
O	-1.01417700	1.91098800	4.10220200
H	-0.16076400	1.81986000	3.64100800
H	-1.39992300	2.66650200	3.62253500

TSII-IIIw4

Pd	0.34066400	-1.83869700	-0.74124300
P	2.29527500	-1.71407000	0.52851000
P	-1.47153800	-2.46907900	-2.06369100
C	2.03311000	-2.35396100	2.29852800
C	3.09220900	-0.06516700	0.81388500
C	3.69890400	-2.66256800	-0.35022600
C	-2.94040400	-3.12433300	-1.04743300
C	-0.90620200	-3.73435700	-3.36805200
C	-2.28061200	-1.15910200	-3.09650700
H	-1.51643200	-0.57922000	-3.61377400
H	-2.83046600	-0.47431800	-2.44494600
H	-2.98181700	-1.57026100	-3.82866700
H	3.92317400	-0.11984600	1.52407700
H	3.47609300	0.31517700	-0.13787000
H	2.35322000	0.64227200	1.19254800
C	0.25091500	1.42145400	-0.95701300
C	-0.54508000	0.47059400	-0.16629000
H	1.27876200	1.52262300	-0.60935100
H	-0.30255300	0.22626900	0.85581300
H	-1.49596100	0.14203300	-0.55024300
C	0.13838200	1.44459100	-2.45023700
C	-0.80783000	2.23524500	-3.10385100
C	0.98722700	0.61919300	-3.19833500
C	-0.91438500	2.19363300	-4.49613900
H	-1.45480700	2.87627700	-2.51494500
C	0.87858500	0.57608100	-4.58748800
H	1.71028700	-0.00636100	-2.68369700
C	-0.07585700	1.36182200	-5.24040800
H	-1.65405100	2.81090700	-4.99824400
H	1.53303000	-0.07527100	-5.15948600
H	-0.16209400	1.32702900	-6.32271800
N	-0.74277100	2.22809400	-0.22186400
S	-0.11378200	3.06808200	1.05584600
O	0.91777100	2.30951400	1.80732200
O	-1.26208700	3.58555900	1.84366600
C	0.69765500	4.44251700	0.27114700
C	2.08411800	4.42513700	0.12226100
C	-0.07822900	5.47302400	-0.26591500
C	2.69903100	5.46296800	-0.57642200
H	2.66534700	3.61913900	0.55577000
C	0.55481500	6.50035900	-0.95616000
H	-1.15529200	5.46656700	-0.13927000
C	1.94899700	6.51134800	-1.12404300
H	3.77876900	5.45822300	-0.69662400
H	-0.03957900	7.30742800	-1.37554300
C	-4.25560700	-3.26349800	-1.83248300
H	-4.19482100	-4.00100000	-2.63507300
H	-5.04793500	-3.58715900	-1.14504100
H	-4.57347500	-2.31073400	-2.26715900
C	-2.57236000	-4.46993800	-0.40102500
H	-1.61300400	-4.41306800	0.12490600
H	-3.34192200	-4.74153400	0.33278300
H	-2.51906600	-5.27870900	-1.13593400
C	-3.15010900	-2.09179100	0.07809400
H	-3.97135200	-2.42417800	0.72611400
H	-2.26090600	-1.96151700	0.69935100
H	-3.43486600	-1.10833400	-0.31368200
C	3.43137000	-4.17494100	-0.28816000
H	2.41295200	-4.41652700	-0.60796800
H	4.12887200	-4.69761100	-0.95559400
H	3.57766600	-4.57423100	0.71906200
C	3.63703300	-2.21665000	-1.82620300
H	4.41375500	-2.73902300	-2.40040100
H	2.65990800	-2.44178800	-2.26524400
H	3.81446300	-1.14026300	-1.93390400
C	5.11069700	-2.36383200	0.18245700
H	5.84304400	-2.93638100	-0.40185100
H	5.36872000	-1.30558600	0.07973000
H	5.23546200	-2.64403100	1.22986500
C	1.13378200	-3.60314600	2.24063200
H	0.88841600	-3.92150600	3.26218600
H	0.20088000	-3.38074600	1.71230700
H	1.61724100	-4.44264400	1.73401700
C	3.32478700	-2.67230500	3.06496200
H	3.07462200	-2.91997600	4.10465800
H	3.85549800	-3.53057800	2.64330600
H	4.01163900	-1.81937000	3.08979300
C	1.26188000	-1.25107900	3.05263200
H	0.32932800	-0.97620100	2.55294700
H	0.99364600	-1.62500600	4.04882500
H	1.86319100	-0.34778400	3.19371800
C	-0.00838100	-4.78285900	-2.68732800
H	0.41867000	-5.44983900	-3.44805400
H	0.81115500	-4.29348000	-2.15214100
H	-0.55372900	-5.39961600	-1.96941200
C	-2.04101600	-4.43546900	-4.12611400
H	-1.61730500	-5.04233900	-4.93717700
H	-2.60804500	-5.10760100	-3.47672800
H	-2.73871200	-3.72388300	-4.57927200
C	-0.03434300	-2.95064200	-4.36915000
H	0.75526000	-2.39402200	-3.85429200
H	0.44007600	-3.65697400	-5.06253000
H	-0.61704700	-2.24057100	-4.96286600
C	2.62243800	7.64371100	-1.85739200
H	2.77247800	8.50360100	-1.19188400
H	3.60414900	7.34597500	-2.23755800
H	2.01577300	7.98806900	-2.70121600

O -3.39021700 1.84357100 1.11929200
H -2.82175200 1.82283100 0.32870600
H -2.95313700 2.55927200 1.61807000
O -1.84784700 -0.19083200 2.44053500
H -2.47569000 0.45977500 2.06429700
H -1.75918900 0.02944600 3.39551400
O -0.15577400 2.82075500 4.41979800
H 0.50871800 2.47727900 3.79294000
H -0.77771400 3.25067200 3.80303400
O -1.46585600 0.52154800 5.10199600
H -0.96702700 1.36059000 4.92912900
H -2.31400200 0.81489700 5.46844600

TSIII-IVw3
Pd -1.69991800 0.11439100 -0.03898200
P -1.74248500 -2.25028000 -0.50521700
P -1.82934200 2.46035100 -0.04175100
C -0.38521100 -3.37199300 0.00736800
C -1.79541800 -2.43772100 -2.39659200
C -3.28041500 -2.98654800 0.32554800
C -3.12542700 2.79179900 -1.38838300
C -0.32065200 3.31879300 -0.65323900
C -2.22785400 3.36513600 1.57558900
H 0.07531300 2.79265200 -1.52148900
H -0.52682800 4.36001500 -0.91556200
H 0.45547800 3.29213900 0.11432900
H -0.69714700 -4.41546900 -0.09880700
H 0.49851100 -3.20798600 -0.61396500
H -0.10607100 -3.16542400 1.05147700
C 1.26798700 -0.40896000 0.15102300
C 0.07277800 0.09723500 0.96030100
H 0.92485000 -1.05105300 -0.66167000
H -0.20284800 -0.56980600 1.77208700
H 0.25796800 1.09732500 1.34160800
C 2.03375900 0.74341000 -0.47584200
C 2.74695500 1.63835900 0.33082400
C 2.03429000 0.93352200 -1.85888000
C 3.43626600 2.70823300 -0.23427700
H 2.78648300 1.46245000 1.40147200
C 2.71994400 2.00895500 -2.43141700
H 1.50688200 0.22719900 -2.49194400
C 3.41953100 2.90156500 -1.61970800
H 3.99847200 3.38573800 0.40253300
H 2.71567500 2.14018400 -3.51030000
H 3.96093900 3.73368600 -2.06124600
N 2.09168100 -1.18817200 1.09471300
S 3.24507300 -2.13184600 0.41590300
O 2.86951800 -2.57939000 -0.94911200
O 3.60924500 -3.14737100 1.42125200
C 4.64886500 -1.03628700 0.21230600
C 5.01268300 -0.59184600 -1.05712100
C 5.30108700 -0.56308100 1.34965800
C 6.02705000 0.35304900 -1.18246300
H 4.48525500 -0.97073200 -1.92383600
C 6.31723500 0.37920700 1.20878500
H 5.00775800 -0.92274800 2.33027800
C 6.68639700 0.86014200 -0.05454100
H 6.30301600 0.71287400 -2.17045200
H 6.82788900 0.75344600 2.09232100
H 1.46349300 -1.76798600 1.90678600
C 7.74560000 1.92370600 -0.20565600
H 8.52090200 1.61984600 -0.91893400
H 7.30896900 2.85622700 -0.58507400
H 8.23196900 2.14648500 0.75185900
C -2.46888700 2.41075200 -2.73192000
H -3.23705100 2.40316100 -3.51487500
H -1.69641100 3.12579500 -3.02871300
H -2.01824300 1.41272400 -2.69401000
C -4.32119000 1.84767300 -1.15606400
H -4.02417100 0.79692000 -1.25844600
H -4.77861200 1.96970500 -0.17290400
H -5.08945700 2.03730000 -1.91628600
C -3.60752100 4.24686200 -1.46620600
H -4.25344600 4.36385000 -2.34566500
H -4.19155100 4.53617600 -0.58922400
H -2.77471600 4.94889800 -1.57594000
C -3.67123300 3.06483700 2.01156900
H -4.40740500 3.50631300 1.33388500
H -3.84138400 1.98814500 2.09942800
H -3.83658600 3.50567700 3.00263700
C -2.00090600 4.88432400 1.47533600
H -2.63384600 5.36038300 0.72448400
H -2.24191800 5.33649200 2.44528400
H -0.95815700 5.12860300 1.25169100
C -1.27221300 2.80445700 2.64928200
H -1.41875000 1.73403000 2.80124200
H -0.22057100 2.99060500 2.40380100
H -1.48018100 3.31189900 3.59932900
C -3.09155600 -1.83315800 -2.96033800
H -3.97747400 -2.40514500 -2.67151300
H -3.23090400 -0.79573400 -2.63588600
H -3.04360800 -1.83017200 -4.05655900
C -0.60173500 -1.62644900 -2.94072900
H -0.67290400 -0.57241800 -2.65413300
H 0.35694900 -2.01554500 -2.58391400
H -0.59617600 -1.68546100 -4.03669700
C -1.63988000 -3.89148100 -2.87561400
H -1.60947100 -3.90094000 -3.97273400
H -0.70674700 -4.33637300 -2.51901600
H -2.46721200 -4.53028900 -2.56357900
C -4.41925400 -1.94899300 0.29625500
H -5.30174600 -2.36733400 0.79849600
H -4.13483500 -1.03749000 0.83209100
H -4.71856700 -1.68349100 -0.72163500
C -2.88608000 -3.24203800 1.79521600
H -3.79099300 -3.50073800 2.36101400
H -2.18438900 -4.07361200 1.89090100
H -2.43198900 -2.36461400 2.25922300
C -3.76063200 -4.30582800 -0.29510000
H -4.56173000 -4.72176900 0.32921100
H -4.16798400 -4.16560300 -1.29993900
H -2.96200800 -5.05243900 -0.34334900
O 0.60291900 -2.36683000 2.75385400
H 1.14567700 -2.97904400 3.27395500
H -0.31017200 -1.51804300 3.60796200
O -0.93799200 -0.87052200 4.12596900
H -0.37609700 -0.10677200 4.32578900
O -3.27630900 -0.07845000 3.02452100
H -2.39491500 -0.35785500 3.39840500
H -3.85172700 -0.83648700 3.20329300

TSIII-IVw4

Pd	1.74678700	0.11182600	-0.11204600
P	1.68313700	-2.25481400	0.58284900
P	1.74205800	2.49086700	-0.00416400
C	0.14262700	-3.25137800	0.47842800
C	2.00393600	-2.15134700	2.45651300
C	2.92175400	-3.44057900	-0.26036300
C	2.82026800	3.09449800	1.44874900
C	0.11151800	3.24085000	0.42806300
C	2.17120100	3.37833800	-1.63181900
H	-0.36311500	2.66706200	1.22365300
H	0.22827200	4.27977000	0.74515200
H	-0.56166100	3.21630600	-0.42946500
H	0.29961200	-4.23689700	0.91982800
H	-0.69256600	-2.78359300	0.99380800
H	-0.12940100	-3.34470900	-0.57789600
C	-1.38495500	-0.36340400	0.03979800
C	-0.19422000	0.19488000	-0.75229000
H	-1.03195500	-0.94156300	0.89410600
H	-0.01946700	-0.40395500	-1.64364100
H	-0.41737400	1.21023600	-1.06843200
C	-2.24348900	0.75700100	0.59935800
C	-2.97216500	1.59281600	-0.25585900
C	-2.29736200	0.98781100	1.97521500
C	-3.72635700	2.64565600	0.25384000
H	-2.96564000	1.38490500	-1.32127800
C	-3.04685800	2.04897700	2.49295200
H	-1.75593300	0.32490000	2.64491100
C	-3.76042600	2.88284100	1.63244800
H	-4.29966600	3.27654300	-0.42017500
H	-3.08074100	2.21457900	3.56661700
H	-4.35155000	3.70295500	2.03077400
N	-2.11144200	-1.22784500	-0.90850600
S	-3.30211300	-2.15690600	-0.29885200
O	-3.05669200	-2.54900000	1.11193000
O	-3.56092800	-3.21806600	-1.29348400
C	-4.75029900	-1.09828300	-0.26074700
C	-5.25441900	-0.64550200	0.95558600
C	-5.30572300	-0.66933400	-1.46645500
C	-6.30831300	0.26572700	0.96096900
H	-4.80519500	-0.99259800	1.87801700
C	-6.36109100	0.23784900	-1.44675100
H	-4.90689900	-1.03993200	-2.40500000
C	-6.86858000	0.72965400	-0.23511600
H	-6.69389800	0.63166800	1.90916000
H	-6.79673300	0.57541400	-2.38405300
H	-1.38215400	-1.83587900	-1.69753400
C	-7.96912200	1.76174800	-0.22009600
H	-8.63142900	1.62907500	0.64188100
H	-7.55210900	2.77586700	-0.15585300
H	-8.57694100	1.71473400	-1.12940700
C	2.08366900	2.65062100	2.73113500
H	2.73669700	2.82306200	3.59524700
H	1.16301400	3.21761000	2.89190200
H	1.83024500	1.58634300	2.70764200
C	4.20717900	2.42964000	1.44170100
H	4.13463300	1.34616500	1.54740300
H	4.78148700	2.67080200	0.54154700
H	4.78513600	2.80196800	2.29611100
C	3.01036400	4.61939600	1.48223900
H	3.50094200	4.89336300	2.42461100
H	3.64980600	4.96917200	0.66749400
H	2.06343800	5.16394600	1.43791900
C	3.67130600	3.25576400	-1.94443900
H	4.28922600	3.79794700	-1.22302300
H	3.99368300	2.21268500	-1.98871300
H	3.86578200	3.69034800	-2.93249500
C	1.76166100	4.86246200	-1.65495700
H	2.29688300	5.46325400	-0.91800000
H	1.99492000	5.27171200	-2.64584600
H	0.68845100	4.99567200	-1.49308600
C	1.37892500	2.64276800	-2.73241700
H	1.64659800	1.58617600	-2.78120500
H	0.29745100	2.71923700	-2.57976400
H	1.60891300	3.10047500	-3.70238900
C	3.23356600	-1.27032300	2.73594700
H	4.15713500	-1.69502900	2.33999900
H	3.10531500	-0.27813600	2.29697700
H	3.35335500	-1.14723400	3.82021500
C	0.77319400	-1.44840000	3.06696400
H	0.57908900	-0.49036800	2.57326900
H	-0.13124700	-2.05914200	2.99996800
H	0.96370900	-1.25028800	4.12954800
C	2.18534500	-3.51689200	3.13626900
H	2.25527900	-3.37010600	4.22202200
H	1.34080400	-4.18704500	2.95040700
H	3.10263700	-4.01612400	2.81373300
C	4.37359900	-3.15990000	0.15986800
H	5.04413900	-3.73089200	-0.49623200
H	4.62508200	-2.10229000	0.06933400
H	4.57173800	-3.48700200	1.18533600
C	2.76519400	-3.18943600	-1.77224300
H	3.34355700	-3.94526100	-2.32089500
H	1.72111600	-3.24944200	-2.09501000
H	3.14325100	-2.20681100	-2.05334600
C	2.60935500	-4.92743800	0.00119400
H	3.40690400	-5.52824100	-0.45412300
H	2.57295500	-5.17949700	1.06327200
H	1.66766700	-5.23397500	-0.45980200
O	-0.52160700	-2.40347000	-2.46895300
H	-1.05379900	-2.99066500	-3.02835300
H	0.46866300	-1.55268500	-3.28859200
O	1.10402800	-0.93086900	-3.82269800
H	0.56561700	-0.14725600	-4.01005300
H	3.90808200	-0.13220400	-1.50806500
O	4.03937500	-0.04669300	-0.51434500
H	4.50485000	0.79245100	-0.38574500
O	3.54812400	-0.15062700	-3.12757800
H	2.60738100	-0.45748900	-3.32574600
H	4.11365300	-0.76217500	-3.62475800

IVw3

Pd	1.69805900	0.07469800	-0.17721600
P	1.78044900	-2.33319900	0.26301700
P	1.50568500	2.47482000	-0.06937600
C	0.35858800	-3.34240000	-0.35274700
C	1.66829300	-2.59560000	2.15040100
C	3.22677500	-3.32118100	-0.50202200
C	1.74604300	3.00738700	1.74804400

C	-0.17063700	3.14909400	-0.42982900
C	2.58185200	3.53836500	-1.23015300
H	-0.86519700	2.83023300	0.34495800
H	-0.16280500	4.24078100	-0.47085100
H	-0.53591900	2.76491000	-1.38097700
H	0.47485900	-4.38953000	-0.07203900
H	-0.59019200	-2.99901400	0.04889100
H	0.31946300	-3.28513100	-1.44357900
C	-1.38021200	-0.55700500	-0.42935700
C	-0.11022400	-0.15918600	-1.20526100
H	-1.10495600	-1.17734100	0.42260600
H	0.12426300	-0.96777200	-1.91148500
H	-0.31485100	0.72898500	-1.80926500
C	-2.19320800	0.60102100	0.11203900
C	-2.95112800	1.40786500	-0.74439000
C	-2.22569800	0.85653100	1.48405000
C	-3.70110400	2.46695500	-0.24085400
H	-2.96305700	1.18328500	-1.80587700
C	-2.96367900	1.92824600	1.99440600
H	-1.67690700	0.20742100	2.15868900
C	-3.70087800	2.73868300	1.13117000
H	-4.29057500	3.08026600	-0.91636100
H	-2.97149000	2.11805500	3.06411100
H	-4.28398600	3.56697600	1.52369200
N	-2.24959400	-1.35281700	-1.34491000
S	-3.41642200	-2.34745800	-0.63872400
O	-2.99276400	-2.77106900	0.70988600
O	-3.72255200	-3.35893100	-1.66150100
C	-4.80480100	-1.25180800	-0.44582500
C	-5.14377200	-0.79002600	0.82365700
C	-5.48776500	-0.82121100	-1.58320800
C	-6.17365300	0.13759700	0.94855700
H	-4.59148100	-1.13834200	1.68743700
C	-6.51628100	0.10444800	-1.43849700
H	-5.21190500	-1.20001800	-2.56131300
C	-6.86406900	0.60733300	-0.17616100
H	-6.43516400	0.51435300	1.93331100
H	-7.05366500	0.44913100	-2.31770900
H	-1.72167400	-1.93062100	-1.99634000
C	-7.93275500	1.66094200	-0.03202100
H	-8.51131200	1.52153500	0.88695400
H	-7.48201300	2.66109100	0.01587000
H	-8.62451200	1.65145300	-0.87970800
C	1.07741100	1.92141800	2.61006200
H	1.15988600	2.19997900	3.66897700
H	0.01623900	1.81199600	2.37323000
H	1.55993000	0.95307400	2.46962500
C	3.23945100	3.07171400	2.10943500
H	3.75063600	2.15847300	1.79292500
H	3.73344500	3.93375600	1.65192800
H	3.33490300	3.18213600	3.19799500
C	1.06301900	4.34776800	2.07843800
H	1.27346900	4.59260000	3.12739400
H	1.42864800	5.17416400	1.46823700
H	-0.02382100	4.29307100	1.96761300
C	4.03766700	3.05195200	-1.23564100
H	4.50028200	3.10504500	-0.24938200
H	4.10593900	2.02516900	-1.58672100
H	4.61534000	3.68710400	-1.92041000
C	2.55865900	5.03488900	-0.87135600
H	3.05727400	5.24288000	0.07825400
H	3.10103000	5.58373700	-1.65156700
H	1.54645900	5.44791300	-0.83042600
C	2.01030500	3.36540900	-2.65248900
H	2.03585200	2.32171200	-2.97613900
H	0.99183400	3.75468200	-2.74255000
H	2.63961200	3.93498600	-3.34825600
C	2.76926300	-1.80872400	2.88419200
H	3.77378300	-2.14950000	2.62353300
H	2.70007000	-0.73758400	2.68504300
H	2.64565600	-1.95225000	3.96478400
C	0.30885400	-2.01547900	2.59125200
H	0.21250100	-0.97079100	2.28337800
H	-0.54267300	-2.57619700	2.19623900
H	0.24674200	-2.04978200	3.68626800
C	1.74724900	-4.06911400	2.58100700
H	1.53407000	-4.13379500	3.65552700
H	1.01742900	-4.70025600	2.06618000
H	2.74485500	-4.48556900	2.42161700
C	4.56195300	-3.00128400	0.18754300
H	5.37595800	-3.44363300	-0.39959900
H	4.74803300	-1.92832800	0.24003600
H	4.61373700	-3.43086700	1.19263700
C	3.29430200	-2.87320800	-1.97325700
H	4.04923400	-3.47650800	-2.49324500
H	2.34230900	-3.02314100	-2.49413300
H	3.58017400	-1.83002700	-2.07204000
C	3.01454900	-4.84822300	-0.48714700
H	3.91864800	-5.31820100	-0.89350100
H	2.85421600	-5.25620600	0.51187800
H	2.18091600	-5.15464400	-1.12390400
O	2.81191400	0.17709300	-3.34126500
H	2.15380600	0.07233500	-2.63307700
H	3.66386500	0.12948400	-2.84355700
O	5.05487000	-0.09477000	-1.68779900
H	5.71940200	0.60733700	-1.75400500
O	3.71764200	0.35164200	0.51328400
H	4.57727600	0.07824600	-0.80582600
H	3.92420600	-0.32540400	1.17024000

P

C	1.39308300	0.98024400	-0.99541700
C	0.88197200	0.86994700	-2.44435800
H	0.53688800	0.97041700	-0.31090500
H	0.08517400	1.61647100	-2.59878600
H	1.69340900	1.14068700	-3.13111200
C	2.29669100	-0.18487400	-0.62739000
C	3.43558400	-0.48054800	-1.38734400
C	1.99391000	-0.98443300	0.47735200
C	4.25026400	-1.55941200	-1.04980600
H	3.69563500	0.15204500	-2.23029200
C	2.80472300	-2.06863200	0.81599800
H	1.12531900	-0.74424100	1.08303900
C	3.93432900	-2.35954500	0.05186000
H	5.13551400	-1.77271700	-1.64213300
H	2.55512800	-2.68145600	1.67744700
H	4.56995800	-3.20042200	0.31432900
N	2.13031300	2.26179500	-0.87490600
S	2.67836600	2.72796000	0.65542700
O	1.83460600	2.16500900	1.72163300

O 2.85801100 4.18397800 0.55190800
C 4.27042100 1.93609300 0.73882500
C 4.50369000 0.95984900 1.70314000
C 5.25308900 2.29413500 -0.18536100
C 5.73862700 0.31616300 1.72388000
H 3.72046500 0.69780900 2.40344400
C 6.48017000 1.64127800 -0.14911100
H 5.05241800 3.06147500 -0.92494500
C 6.73706300 0.63645800 0.79619000
H 5.92428500 -0.45680700 2.46431300
H 7.25054500 1.90898200 -0.86719700
H 1.60787500 3.05064000 -1.25539600
C -0.70212000 -1.41121200 -1.91035900
C -0.57917600 -2.85642500 -2.47260600
C -0.29748000 -2.64209600 -4.01716700
C 0.64678100 -1.40913600 -4.05446400
H -1.70835100 -1.00956000 -2.12435900
H -0.59446600 -1.36730500 -0.82112300
H 1.69846300 -1.71846800 -3.92449600
H 0.62474700 -0.87398400 -5.01318400
B 0.29947700 -0.55965300 -2.77710000
C 8.04719700 -0.10996600 0.78919300
H 8.33575500 -0.42201900 1.79793300
H 7.97064700 -1.01777200 0.17615100
H 8.85489800 0.49973900 0.37251500
C -1.59230500 -2.28337500 -4.77370200
H -1.35448900 -2.01506800 -5.80987000
H -2.29220000 -3.12488700 -4.80362300
H -2.11355100 -1.42984100 -4.32584400
C 0.32388500 -3.86770600 -4.70104700
H 0.45231300 -3.67694500 -5.77347400
H 1.30899000 -4.10848800 -4.28967200
H -0.31287300 -4.75554800 -4.59924000
C 0.61984200 -3.53714300 -1.78138000
H 0.46637400 -3.54770600 -0.69693900
H 0.73773000 -4.57503000 -2.11031600
H 1.56496800 -3.01422000 -1.96221400
C -1.83017100 -3.69829000 -2.18891900
H -1.76661100 -4.68740700 -2.66012800
H -1.94278000 -3.85591800 -1.10941800
H -2.74267600 -3.21110700 -2.54661500

X
Pd -1.28647500 -0.84648900 0.57820600
P -0.80251600 -3.07207200 0.40868900
C 0.81283300 -3.67559900 1.08381000
C -0.71273400 -3.58923900 -1.42238900
C -2.04891800 -4.15277900 1.35878100
H 0.99497100 -4.72707200 0.84253700
H 1.63023800 -3.08053100 0.67559300
H 0.81151600 -3.55477700 2.17040000
C 2.11815200 -0.27767400 0.76559000
C 1.42669300 -0.20348400 2.13051600
H 1.63037700 -1.06068200 0.17943300
H 1.60194900 -1.16042800 2.64793500
H 1.93947400 0.55842600 2.74141700
C 2.03572100 1.00616000 -0.03479000
C 2.68298200 2.16947200 0.39501800
C 1.30489300 1.03997800 -1.22451300
C 2.59334100 3.34571400 -0.34704600
H 3.27770000 2.14056100 1.30254100
C 1.21288900 2.21481900 -1.97235900
H 0.80091200 0.13970200 -1.56100300
C 1.85533700 3.37300200 -1.53421700
H 3.10751300 4.23980000 -0.00553000
H 0.64797500 2.22017500 -2.89979300
H 1.78899800 4.28820900 -2.11591600
N 3.54369500 -0.63526300 1.00168100
S 4.44748700 -1.20678900 -0.29960700
O 3.59132600 -1.89307400 -1.28374500
O 5.57750400 -1.92495600 0.31049700
C 5.02819500 0.30206800 -1.04505200
C 4.49785000 0.71437100 -2.26508500
C 5.95578400 1.08265100 -0.35556600
C 4.88787000 1.94386200 -2.78723200
H 3.77570000 0.08909300 -2.77475800
C 6.33678600 2.30762800 -0.89443400
H 6.36118500 0.73968900 0.59010400
C 5.79802500 2.76268100 -2.10652000
H 4.46436700 2.28148700 -3.72896700
H 7.05452000 2.92647500 -0.36277700
H 3.65840400 -1.30287100 1.76344200
O -3.73536100 -0.42078400 -0.70917900
H -4.53023300 -0.51347600 -0.16097000
C -0.87150900 1.36151000 1.44791500
C -1.65632900 2.08147800 2.58283500
C -2.08780100 0.93887600 3.58432800
C -0.86607900 -0.02102000 3.61582700
H -1.62117600 1.09589200 0.64258000
H -0.19896000 2.03585500 0.91298900
H -0.15946600 0.30779200 4.39627600
H -1.16061600 -1.03725000 3.90464100
C -2.54741500 1.10708700 -2.42869600
C -4.88883600 1.78656500 -1.60346800
C -2.89916200 2.55839500 -2.87201900
H -2.52489800 0.42838400 -3.29531600
H -1.55460300 1.03668600 -1.96940900
C -4.48630600 2.59450700 -2.87288700
H -4.85909400 2.43361000 -0.71295800
H -5.91551900 1.40176400 -1.66081600
B -0.14157600 0.11261400 2.18173800
B -3.74469700 0.70408900 -1.48783400
C 6.15584400 4.12222500 -2.65111800
H 6.28846800 4.09672000 -3.73794100
H 5.35506400 4.84289200 -2.43904400
H 7.07579800 4.50712200 -2.20113200
C -5.06695300 4.01509400 -2.85738100
H -4.72041400 4.60382400 -3.71585100
H -6.16185800 3.97559200 -2.90717800
H -4.79864600 4.55690000 -1.94532000
C -5.05115700 1.86127100 -4.10570400
H -6.14214000 1.78628600 -4.02762000
H -4.82291700 2.39479700 -5.03391000
H -4.65782800 0.84244100 -4.20041400
C -2.28167600 2.90850300 -4.23200900
H -2.59809900 3.89984700 -4.57937400
H -1.18784000 2.92343200 -4.15559900
H -2.54605700 2.17813700 -5.00292500
C -2.33308000 3.53648200 -1.82314100
H -1.25281200 3.39359500 -1.71997400

H	-2.50695800	4.57947000	-2.10807400
H	-2.77321300	3.38256000	-0.83343000
C	-3.32342600	0.18366800	3.05696700
H	-3.55153500	-0.65948700	3.71928900
H	-4.21079700	0.82595200	3.01957500
H	-3.15198000	-0.22905000	2.05337100
C	-2.43869300	1.46512100	4.98461600
H	-2.78096500	0.63857600	5.61951700
H	-1.57487100	1.92005800	5.47877600
H	-3.24397700	2.21095700	4.95466100
C	-0.66752500	3.05629300	3.25543800
H	-0.26477200	3.75080800	2.50884500
H	-1.15313000	3.65110300	4.03619600
H	0.18029800	2.53052000	3.70701900
C	-2.83489800	2.90446600	2.04463400
H	-3.43595300	3.33216100	2.85691000
H	-2.46705500	3.73631000	1.43297600
H	-3.49906800	2.30217100	1.41599800
C	-1.85806800	-2.89809700	-2.18386600
H	-2.84402300	-3.24106700	-1.86282200
H	-1.82417700	-1.81413200	-2.04117200
H	-1.75959200	-3.11003000	-3.25666300
C	0.61981800	-3.04333900	-1.96924700
H	0.73331900	-1.97437200	-1.77151500
H	1.49130000	-3.55000200	-1.54969200
H	0.64378100	-3.18644300	-3.05704400
C	-0.76002600	-5.10377400	-1.66705400
H	-0.58199800	-5.30115900	-2.73209800
H	0.01219900	-5.63703000	-1.10355000
H	-1.73311700	-5.53320100	-1.41395700
C	-3.41730400	-4.09177400	0.66085400
H	-4.18181600	-4.52889400	1.31597500
H	-3.70426200	-3.05726900	0.44396900
H	-3.42708200	-4.65377400	-0.27721500
C	-2.18681200	-3.50364200	2.74900800
H	-2.87448900	-4.10069700	3.36236500
H	-1.22824400	-3.45351800	3.27612600
H	-2.57738800	-2.48797200	2.67192300
C	-1.61628200	-5.61570300	1.55458200
H	-2.39277400	-6.14265500	2.12405100
H	-1.47805900	-6.15064200	0.61420600
H	-0.68794700	-5.68831200	2.12897000

TSIII-XI

Pd	-0.66650200	-1.07604200	-1.43836000
P	1.64708800	-1.26352800	-2.13750000
P	-2.01312300	-2.95949200	-1.86605700
C	1.86374200	-1.08039400	-4.01717100
C	2.76451500	-2.62599300	-1.41933400
C	2.45734900	0.27192400	-1.49261900
C	-2.78010800	-3.64113600	-0.26051700
C	-1.15449200	-4.46258900	-2.52414500
C	-3.33088400	-2.69169500	-3.22065700
C	-1.12008500	1.17742500	-0.51766300
C	-2.15989200	0.13410400	-0.57638700
H	-0.15251900	0.52182300	-1.12788900
H	-2.45715500	-0.26368000	0.38883900
H	-2.99024100	0.31793200	-1.24763000
C	-1.24965100	2.31609600	-1.51647600
C	-1.61227800	2.06859600	-2.84630900
C	-0.99637100	3.63016200	-1.11520200
C	-1.73483200	3.11574300	-3.75717400
H	-1.77615200	1.04583400	-3.17112000
C	-1.12012000	4.68094600	-2.02738800
H	-0.70951000	3.80837700	-0.08585400
C	-1.48901700	4.42992900	-3.34950700
H	-2.01405400	2.90512800	-4.78611300
H	-0.92637600	5.69914100	-1.70077800
H	-1.58121600	5.24828000	-4.05814900
N	-0.53048400	1.59252100	0.68303100
S	-0.27039600	0.47958300	1.79452000
O	0.45955800	-0.71017700	1.23564400
O	-1.44090800	0.09742900	2.63274500
C	0.87582600	1.34172500	2.86617100
C	0.77458000	1.15495600	4.24231200
C	1.90732800	2.11405900	2.32678800
C	1.71438600	1.74987400	5.08518200
H	-0.03899300	0.55787800	4.63915900
C	2.83635900	2.70219800	3.17939500
H	1.95732200	2.26301800	1.25415300
C	2.75547300	2.53046900	4.57029700
H	1.63466400	1.60799100	6.15997500
H	3.63698800	3.30890000	2.76270100
H	-1.86424000	-5.20108200	-2.90534800
H	-0.46888900	-4.18244000	-3.32315900
H	-0.56997000	-4.92627100	-1.72993400
H	3.54384500	0.26418600	-1.62220700
H	2.04210600	1.14156900	-2.00909500
H	2.21157600	0.35759000	-0.43258200
C	1.56404500	-2.41937100	-4.70750000
H	2.30426300	-3.18718500	-4.46632700
H	0.57504000	-2.79302100	-4.42739700
H	1.57110100	-2.28170000	-5.79611200
C	3.24137700	-0.55425900	-4.45396400
H	3.23434500	-0.39989000	-5.54062800
H	3.47297900	0.40880700	-3.98962300
H	4.05110900	-1.24806500	-4.22597500
C	0.79854600	-0.05617700	-4.45794600
H	-0.20764800	-0.39651600	-4.19997400
H	0.94344800	0.92358500	-3.99282800
H	0.85237300	0.07904200	-5.54587700
C	4.13448700	-2.75013600	-2.10276700
H	4.74590900	-3.46590400	-1.53841100
H	4.05933700	-3.12310200	-3.12817600
H	4.67550500	-1.79907600	-2.11762100
C	2.02130900	-3.96539000	-1.49694700
H	2.64689200	-4.75798100	-1.06729400
H	1.09482000	-3.91399300	-0.92049700
H	1.77579200	-4.25210000	-2.52277700
C	2.98410400	-2.29165000	0.07073500
H	3.66045000	-1.44151900	0.19949400
H	2.05158400	-2.06011000	0.59020100
H	3.44998800	-3.15911500	0.55558800
C	-3.19848200	-5.12044100	-0.35992300
H	-3.61879400	-5.42745500	0.60571300
H	-3.96131700	-5.29595100	-1.12117000
H	-2.34926900	-5.77745900	-0.56615700
C	-3.99727200	-2.81071000	0.17889500
H	-4.30793800	-3.14117800	1.17760200

H	-3.77250400	-1.74616700	0.24309400
H	-4.85165300	-2.94534700	-0.49036100
C	-1.67143400	-3.51660400	0.80584600
H	-0.78207400	-4.09691100	0.53345300
H	-1.35593500	-2.48333000	0.96767500
H	-2.04490100	-3.91577100	1.75726900
C	-4.35195400	-3.83798500	-3.30951300
H	-5.00155900	-3.87604600	-2.43206700
H	-4.99217600	-3.67514000	-4.18560400
H	-3.87870000	-4.81630300	-3.43055300
C	-4.08111600	-1.36765600	-3.00865000
H	-4.85788200	-1.27120900	-3.77761900
H	-4.56827000	-1.31023400	-2.03316400
H	-3.40757000	-0.51427500	-3.10599900
C	-2.57320000	-2.59326000	-4.56058300
H	-1.79717800	-1.82225500	-4.52797900
H	-2.10834900	-3.54050100	-4.84878900
H	-3.28245900	-2.31903600	-5.35130200
C	3.75586900	3.19555200	5.48392800
H	3.55914500	4.27228700	5.56735300
H	3.71766000	2.77369800	6.49293900
H	4.77825400	3.08335500	5.10567300

XI

Pd	-0.36878800	-0.99840700	-1.76707700
P	1.75130700	-1.34929000	-2.75511900
P	-1.83694300	-2.99409800	-1.84286400
C	1.72030800	-1.53854900	-4.64605100
C	2.86135400	-2.63111300	-1.89499100
C	2.77404800	0.18595400	-2.56959700
C	-2.53423500	-3.51570300	-0.13979600
C	-1.13306400	-4.61753900	-2.40827700
C	-3.25092300	-2.77231400	-3.10770000
C	-1.07784900	1.40857500	-0.22843900
C	-1.77332600	0.19683700	-0.60471100
H	0.34796800	0.38116300	-1.74786900
H	-2.00227700	-0.42136400	0.25386100
H	-2.64820700	0.37122400	-1.22529100
C	-1.15270800	2.61018200	-1.12036200
C	-1.57246800	2.51048800	-2.45670400
C	-0.80500000	3.87249200	-0.61787400
C	-1.64561200	3.64284700	-3.26505200
H	-1.81003100	1.53837900	-2.87328600
C	-0.88245200	5.00627000	-1.42523200
H	-0.47969200	3.94604000	0.41347600
C	-1.30231300	4.89650200	-2.75232900
H	-1.96503800	3.54528500	-4.29909300
H	-0.61547400	5.97700900	-1.01667900
H	-1.36014200	5.77926400	-3.38314300
N	-0.36345400	1.60511900	0.86731700
S	-0.15280600	0.39536900	1.97315900
O	0.81238600	-0.61321600	1.47318500
O	-1.41723400	-0.12208000	2.54613100
C	0.67382600	1.32680200	3.25494300
C	0.10865200	1.39033000	4.52379700
C	1.90148500	1.93638800	2.98231100
C	0.78234600	2.07937200	5.53464600
H	-0.84459300	0.90800700	4.70812800
C	2.55801600	2.62047400	3.99847300
H	2.32615600	1.87814800	1.98591500
C	2.01054500	2.70170800	5.29015300
H	0.34427400	2.13312500	6.52778500
H	3.51144600	3.10037300	3.79170600
H	-1.91609900	-5.34640300	-2.63133200
H	-0.51924400	-4.47656500	-3.29638900
H	-0.49366800	-5.02835000	-1.62600900
H	3.81724100	0.03659400	-2.86371600
H	2.33718900	0.97799700	-3.18287000
H	2.73172000	0.51261300	-1.52991600
C	1.25387400	-2.95076000	-5.02222100
H	1.98062100	-3.71510300	-4.73177000
H	0.29755500	-3.18503700	-4.55156600
H	1.11660000	-3.01760500	-6.10889900
C	3.05527400	-1.22993200	-5.34600200
H	2.90729600	-1.29697300	-6.43161200
H	3.40810700	-0.21834300	-5.12740300
H	3.84474900	-1.93415000	-5.07864600
C	0.67185900	-0.51652200	-5.13590300
H	-0.29296500	-0.66307800	-4.64224000
H	0.98635200	0.51383100	-4.94146800
H	0.53501000	-0.62633100	-6.21929200
C	4.15507900	-2.95516700	-2.65617800
H	4.79002700	-3.58933400	-2.02422100
H	3.96242800	-3.50534800	-3.58212000
H	4.73059400	-2.05712200	-2.90020800
C	2.06506700	-3.91952800	-1.65751700
H	2.69660500	-4.64682600	-1.13149900
H	1.19208000	-3.71518700	-1.03391200
H	1.72789000	-4.38109800	-2.58848000
C	3.21535900	-2.03429900	-0.51541300
H	3.93837700	-1.21776200	-0.59789500
H	2.33455000	-1.66382500	0.01858100
H	3.67609800	-2.81983700	0.09677600
C	-3.00547400	-4.98156300	-0.08167700
H	-3.39233400	-5.18162700	0.92554400
H	-3.80703600	-5.19833300	-0.79068300
H	-2.18983000	-5.68724300	-0.25907100
C	-3.70573600	-2.62269800	0.30682100
H	-3.94744500	-2.85503100	1.35123500
H	-3.47769300	-1.55915800	0.25706900
H	-4.60558300	-2.80660100	-0.28666200
C	-1.35387600	-3.35312100	0.83977200
H	-0.51832800	-4.00958500	0.56882700
H	-0.97403400	-2.33212100	0.88126800
H	-1.67890900	-3.63454400	1.84931500
C	-4.35735000	-3.83523400	-3.02084000
H	-4.93660400	-3.75785000	-2.09799200
H	-5.05471800	-3.69318900	-3.85673200
H	-3.96469000	-4.85364100	-3.09663400
C	-3.87040900	-1.37314300	-2.95620900
H	-4.67442300	-1.25103600	-3.69335800
H	-4.29854700	-1.20897800	-1.96459800
H	-3.12198400	-0.59730500	-3.13706200
C	-2.60960700	-2.85235200	-4.50736500
H	-1.77893100	-2.14910400	-4.60985700
H	-2.24317700	-3.85669200	-4.73980600
H	-3.36187000	-2.59248100	-5.26282900
C	2.74155400	3.44224900	6.38287100
H	2.94153400	4.48084600	6.09341200
H	2.16365800	3.45557900	7.31168200

H	3.71106200	2.97557000	6.59641700

TSXI-XII

Pd	-0.25602800	-1.21015000	-2.17870500
P	1.82190300	-0.73537000	-3.12120500
P	-1.51954500	-3.18846400	-2.70419500
C	1.74737200	-0.40799000	-4.99203200
C	3.16012100	-1.99789000	-2.65606100
C	2.53475600	0.84940200	-2.47807100
C	-1.97113700	-4.11462700	-1.10225500
C	-0.70579200	-4.53770600	-3.68570500
C	-3.06762100	-2.82525300	-3.75218400
C	-1.06802400	1.50321000	-0.33245000
C	-1.38143000	0.10698400	-0.55063200
H	0.05179900	0.10575700	-1.40262000
H	-1.22567400	-0.53414700	0.31099000
H	-2.33717300	-0.04808600	-1.04411200
C	-1.61265000	2.50474000	-1.29176200
C	-1.90115100	2.14775400	-2.61938800
C	-1.80508000	3.83520100	-0.89002400
C	-2.37925700	3.09827700	-3.51996800
H	-1.71218700	1.13339200	-2.95426100
C	-2.29761900	4.77986800	-1.78738500
H	-1.56781200	4.10915900	0.13200200
C	-2.58570600	4.41572100	-3.10579700
H	-2.58395900	2.80948200	-4.54728400
H	-2.45508300	5.80342800	-1.45914000
H	-2.96320700	5.15543500	-3.80618600
N	-0.28177500	2.01524300	0.59662200
S	0.53708800	1.01109300	1.63573800
O	1.65618600	0.36217100	0.91103400
O	-0.34039200	0.13726700	2.44572800
C	1.23504100	2.23825100	2.72872700
C	0.90549200	2.21507100	4.07973300
C	2.13321000	3.18108900	2.22138300
C	1.48064900	3.15700200	4.93516700
H	0.20914800	1.46994200	4.44728500
C	2.69354900	4.11407900	3.08561600
H	2.38093200	3.18230100	1.16550000
C	2.37635200	4.11758100	4.45448200
H	1.22715600	3.14395300	5.99180800
H	3.39014100	4.85288200	2.69731900
H	-1.41154300	-5.30158300	-4.02520000
H	-0.20318700	-4.10424400	-4.55185100
H	0.05772500	-5.01759600	-3.06937800
H	3.57512200	1.01217400	-2.77732400
H	1.92539600	1.67944200	-2.84777000
H	2.45767100	0.84354000	-1.38853800
C	1.53903300	-1.73152500	-5.74189300
H	2.43210900	-2.36280300	-5.71637600
H	0.70432800	-2.29525700	-5.31485600
H	1.30710800	-1.52750200	-6.79505200
C	2.96885500	0.33318300	-5.56037300
H	2.80870500	0.52157100	-6.63009700
H	3.11711100	1.30321600	-5.07710700
H	3.89171800	-0.24155500	-5.46215800
C	0.49929500	0.47531100	-5.20110500
H	-0.40573000	-0.03267000	-4.85487700
H	0.57072800	1.42674400	-4.66400700
H	0.38666200	0.70137700	-6.26952000
C	4.48637800	-1.83438100	-3.41172900
H	5.23619700	-2.50803500	-2.97667900
H	4.38976700	-2.09348600	-4.47027100
H	4.88067000	-0.81575400	-3.33980600
C	2.61156900	-3.41761800	-2.86842200
H	3.36121900	-4.15070200	-2.54323900
H	1.70489400	-3.56618600	-2.27794100
H	2.37280800	-3.62449600	-3.91449900
C	3.40596500	-1.80382100	-1.14471200
H	3.95284000	-0.88122900	-0.93314200
H	2.47009300	-1.77220700	-0.57738800
H	4.00759200	-2.64208400	-0.77092800
C	-2.41049800	-5.57418300	-1.30501100
H	-2.61625600	-6.02597100	-0.32599100
H	-3.31927000	-5.66029400	-1.90423900
H	-1.62946600	-6.17382000	-1.78171200
C	-3.06060000	-3.34997300	-0.33293200
H	-3.18425000	-3.79900800	0.66064000
H	-2.79620300	-2.29868800	-0.18876100
H	-4.02991700	-3.39774400	-0.83695100
C	-0.68081600	-4.10209400	-0.25424400
H	0.14300800	-4.62465700	-0.75285300
H	-0.35086100	-3.07993800	-0.04260100
H	-0.86632600	-4.61056300	0.70041500
C	-4.09097500	-3.96765100	-3.81532900
H	-4.57730500	-4.13663800	-2.85096800
H	-4.87730600	-3.71348200	-4.53832000
H	-3.63877100	-4.90950300	-4.14254800
C	-3.74098100	-1.54893900	-3.21865100
H	-4.60358700	-1.29706700	-3.84925300
H	-4.10054700	-1.66185700	-2.19312300
H	-3.04656600	-0.70432700	-3.24083600
C	-2.55823000	-2.53134900	-5.17804900
H	-1.76704000	-1.77456000	-5.17269000
H	-2.17281900	-3.42857100	-5.67166200
H	-3.38671900	-2.14719300	-5.78643400
C	2.98929500	5.14337100	5.37566400
H	2.66286500	6.15670600	5.11073900
H	2.70923500	4.96268600	6.41758300
H	4.08369100	5.13094800	5.31112900

XII

Pd	-1.20204500	0.34750400	-0.09868100
P	-0.07177600	2.04491000	-1.21486900
P	-2.72441100	-1.35308700	0.38347600
C	0.42521900	3.54980500	-0.25452700
C	-1.18288800	2.81790300	-2.56469100
C	1.58868700	1.50157300	-1.97758400
C	-2.41807500	-2.60312400	1.72138000
C	-2.83916200	-2.49206000	-1.14602600
C	-4.44438100	-0.69246700	0.87747400
H	-2.29237300	-2.09468700	2.67806100
H	-3.23273500	-3.32682000	1.81481200
H	-1.48279600	-3.12306900	1.50911600
H	1.03453900	4.24926700	-0.83573900
H	-0.47669600	4.06476600	0.08736200
H	0.98280700	3.24009600	0.62775600
C	1.16894000	0.13453300	2.69586700
C	-0.09083600	-0.47178000	3.24317500
H	-0.89487300	-0.23091800	2.53199300

H	-0.03075500	-1.55302600	3.32435400
H	-0.33710800	-0.03572700	4.21449300
C	1.35159000	1.59885900	2.86224200
C	0.27021500	2.42618000	3.20696000
C	2.61642900	2.17884000	2.66737300
C	0.45142900	3.79996500	3.35594500
H	-0.72021900	2.00489300	3.33119300
C	2.79628100	3.54966900	2.82233000
H	3.44668100	1.53623500	2.39923500
C	1.71390000	4.36481000	3.16852600
H	-0.39595700	4.42892700	3.61153400
H	3.78060600	3.98437800	2.67575800
H	1.85496900	5.43493800	3.28908800
N	2.11688300	-0.48241600	2.06887800
S	2.13270700	-2.14959400	1.81250000
O	0.81560100	-2.73179100	1.49288300
O	2.91169500	-2.73833500	2.91508700
C	3.10024400	-2.18203300	0.31732300
C	2.57533600	-2.80931200	-0.80928200
C	4.37846500	-1.61984800	0.30348500
C	3.34320200	-2.86456200	-1.97147900
H	1.57978500	-3.23459800	-0.77196700
C	5.12578700	-1.67436200	-0.86793600
H	4.77304600	-1.14049100	1.19233100
C	4.61915900	-2.29144600	-2.02318700
H	2.93787900	-3.34930000	-2.85540500
H	6.11866500	-1.23322600	-0.88982000
C	2.58261000	1.39086000	-0.80584100
H	2.85770900	2.37230100	-0.40775700
H	2.18404400	0.78814400	0.01349700
H	3.49941700	0.90019300	-1.15554500
C	1.41525800	0.09899400	-2.58371900
H	1.05734200	-0.60373000	-1.82721900
H	0.70861300	0.08785500	-3.41739700
H	2.38280400	-0.26368600	-2.95261800
C	2.17809200	2.45194400	-3.02864600
H	3.18433800	2.10637100	-3.30154300
H	1.58303400	2.47769700	-3.94545300
H	2.27585400	3.47528900	-2.65149700
C	-1.34773100	1.83283800	-3.73292800
H	-0.43762700	1.74421800	-4.33278900
H	-1.62065300	0.83584600	-3.37111900
H	-2.14774300	2.18304500	-4.39871500
C	-2.56402000	3.01043900	-1.90946200
H	-3.24873200	3.47921600	-2.62936100
H	-2.97899300	2.04977500	-1.59841500
H	-2.51510100	3.65654400	-1.02556200
C	-0.71047600	4.18410700	-3.09024500
H	0.27261500	4.14439700	-3.56096300
H	-1.42327500	4.54318200	-3.84450200
H	-0.67669900	4.93332500	-2.29341800
C	-1.37473400	-2.77341500	-1.54085200
H	-0.85617500	-1.84153600	-1.78308400
H	-0.82070400	-3.26048200	-0.73186600
H	-1.35246500	-3.43502900	-2.41778200
C	-3.51958900	-1.74239300	-2.30164400
H	-3.40202200	-2.31511100	-3.23121900
H	-4.59221300	-1.61149000	-2.12969200
H	-3.06917400	-0.75507100	-2.44956700
C	-3.54248000	-3.83807100	-0.90993600
H	-4.58303700	-3.72504500	-0.60000200
H	-3.53777100	-4.41304000	-1.84548700
H	-3.02476000	-4.43972400	-0.15722900
C	-5.58337900	-1.71972600	0.81398600
H	-6.49569000	-1.27983200	1.23881000
H	-5.81203900	-2.01160600	-0.21482900
H	-5.35913000	-2.62457000	1.38793700
C	-4.79325000	0.52394300	0.00352800
H	-4.88477000	0.26811200	-1.05497000
H	-5.75222000	0.94755300	0.33143600
H	-4.02037300	1.29162200	0.09798200
C	-4.30684300	-0.18926500	2.32984300
H	-4.19589900	-1.01039600	3.04372100
H	-3.44970700	0.48588700	2.43755300
H	-5.21076300	0.36894600	2.60526900
C	5.41949500	-2.30295700	-3.30126800
H	5.23324900	-1.39032000	-3.88272700
H	6.49470200	-2.34789600	-3.10079700
H	5.15058200	-3.15434200	-3.93401700

TSIII-XIw3

Pd	-0.50481800	-1.09142200	-1.57317200
P	1.70196100	-1.58802300	-2.44373300
P	-2.09235800	-2.83665000	-1.88435500
C	1.82043200	-1.45914300	-4.33735400
C	2.63733800	-3.09561000	-1.76532800
C	2.78204500	-0.19588700	-1.89215100
C	-2.86463200	-3.42019200	-0.24343800
C	-1.49101400	-4.43988800	-2.59066200
C	-3.42623700	-2.38434200	-3.16995000
C	-0.72182700	1.15761200	-0.52122300
C	-1.81123700	0.17734400	-0.49047700
H	0.20118100	0.40151100	-1.30491400
H	-2.01597400	-0.25368900	0.48155300
H	-2.68716600	0.44213500	-1.07068400
C	-0.91653100	2.30739300	-1.49261500
C	-1.18865800	2.07734000	-2.84526200
C	-0.87449200	3.62478000	-1.02316400
C	-1.41699300	3.14127500	-3.71627200
H	-1.20150800	1.05668000	-3.21403200
C	-1.10237900	4.69150600	-1.89495700
H	-0.67007600	3.79920700	0.02734300
C	-1.37188300	4.45522900	-3.24397400
H	-1.61919000	2.94398900	-4.76540900
H	-1.07093200	5.70947700	-1.51642300
H	-1.54290500	5.28631500	-3.92200700
N	0.04083000	1.54020300	0.57930500
S	0.27965600	0.52475600	1.81069400
O	1.29391100	-0.52495600	1.49302700
O	-0.96608600	-0.00726300	2.42172800
C	1.02560800	1.66338500	2.97416500
C	0.32944200	2.82379900	3.32115600
C	2.26350200	1.37581400	3.54585600
C	0.89089700	3.71007600	4.23498600
H	-0.63227400	3.03339600	2.86547300
C	2.81263800	2.27718500	4.46174900
H	2.81575000	0.49051700	3.25327600
C	2.13993700	3.45211400	4.82040100
H	0.35579800	4.61903600	4.49739700

H 3.78368800 2.06079600 4.89943700
H -2.32227500 -5.06196000 -2.93030100
H -0.82150100 -4.25214100 -3.42867900
H -0.93585600 -4.99375900 -1.83510000
H 3.83072900 -0.35243900 -2.15811000
H 2.43416800 0.74188600 -2.32951200
H 2.71355200 -0.09395900 -0.80968800
C 1.34098100 -2.76221300 -4.99315300
H 2.02109000 -3.59624400 -4.79902500
H 0.34298100 -3.04039600 -4.64324000
H 1.28453100 -2.62687500 -6.08064800
C 3.22501100 -1.10182700 -4.85353500
H 3.18765700 -1.01445900 -5.94694300
H 3.56950800 -0.14162200 -4.46036500
H 3.97079900 -1.85867700 -4.60833300
C 0.86238800 -0.32097400 -4.73970400
H -0.16681800 -0.54916000 -4.45073700
H 1.13547100 0.63174800 -4.27560900
H 0.89292500 -0.18543800 -5.82833400
C 3.99037100 -3.34766900 -2.44802800
H 4.52158900 -4.13206700 -1.89436100
H 3.87705200 -3.69532900 -3.47919300
H 4.62924700 -2.45997000 -2.44622600
C 1.76495800 -4.35096200 -1.86958200
H 2.31898900 -5.20945000 -1.46980200
H 0.86333600 -4.22637400 -1.26827500
H 1.47218300 -4.59096400 -2.89431500
C 2.87221100 -2.81580100 -0.26753800
H 3.63153100 -2.05093500 -0.09326600
H 1.95613800 -2.49680600 0.23956900
H 3.21802200 -3.74234800 0.20853600
C -3.44360100 -4.84613700 -0.31614000
H -3.87352000 -5.09499000 0.66179000
H -4.23870900 -4.94279700 -1.05881300
H -2.67774000 -5.59551500 -0.53186800
C -3.97400200 -2.46757200 0.23472500
H -4.26513100 -2.74875700 1.25405100
H -3.64938900 -1.42829500 0.26200500
H -4.86804000 -2.53296700 -0.39134500
C -1.70908100 -3.41134600 0.77978600
H -0.87130200 -4.03826700 0.45365300
H -1.32498000 -2.40560700 0.96463000
H -2.07050100 -3.81484600 1.73386800
C -4.60169500 -3.37590800 -3.21134500
H -5.20125700 -3.34149200 -2.29896700
H -5.25934500 -3.10729900 -4.04741000
H -4.27867800 -4.40829000 -3.37282000
C -3.97315800 -0.96878400 -2.92610000
H -4.74428000 -0.75158600 -3.67573500
H -4.42920400 -0.85732100 -1.93965100
H -3.18624800 -0.21892600 -3.03000800
C -2.72963900 -2.38863700 -4.54638000
H -1.84011400 -1.75219500 -4.55145700
H -2.43591100 -3.39371300 -4.86124000
H -3.42501000 -1.99742600 -5.29908900
C 2.73120600 4.40523500 5.82878400
H 3.82407800 4.34841800 5.83853400
H 2.44191400 5.43998000 5.61833100
H 2.38120400 4.16773300 6.84182300
O 4.16916900 -0.28318700 1.42380100
H 3.20283600 -0.40295800 1.39120500
O 4.25607800 2.51527900 1.24863400
H 4.28990700 0.68635500 1.29782400
H 3.57013300 2.67362400 0.55170200
H 3.78259000 2.66989400 2.08165500
O 2.23705100 2.76421300 -0.56264200
H 1.48759700 2.31286500 -0.07911000
H 1.89142700 3.64996500 -0.75168200

TSIII-XIw4
Pd -0.50481800 -1.09142200 -1.57317200
P 1.70196100 -1.58802300 -2.44373300
P -2.09235800 -2.83665000 -1.88435500
C 1.82043200 -1.45914300 -4.33735400
C 2.63733800 -3.09561000 -1.76532800
C 2.78204500 -0.19588700 -1.89215100
C -2.86463200 -3.42019200 -0.24343800
C -1.49101400 -4.43988800 -2.59066200
C -3.42623700 -2.38434200 -3.16995000
C -0.72182700 1.15761200 -0.52122300
C -1.81123700 0.17734400 -0.49047700
H 0.20118100 0.40151100 -1.30491400
H -2.01597400 -0.25368900 0.48155300
H -2.68716600 0.44213500 -1.07068400
C -0.91653100 2.30739300 -1.49261500
C -1.18865800 2.07734000 -2.84526200
C -0.87449200 3.62478000 -1.02316400
C -1.41699300 3.14127500 -3.71627200
H -1.20150800 1.05668000 -3.21403200
C -1.10237900 4.69150600 -1.89495700
H -0.67007600 3.79920700 0.02734300
C -1.37188300 4.45522900 -3.24397400
H -1.61919000 2.94398900 -4.76540900
H -1.07093200 5.70947700 -1.51642300
H -1.54290500 5.28631500 -3.92200700
N 0.04083000 1.54020300 0.57930500
S 0.27965600 0.52475600 1.81069400
O 1.29391100 -0.52495600 1.49302700
O -0.96608600 -0.00726300 2.42172800
C 1.02560800 1.66338500 2.97416500
C 0.32944200 2.82379900 3.32115600
C 2.26350200 1.37581400 3.54585600
C 0.89089700 3.71007600 4.23498600
H -0.63227400 3.03339600 2.86547300
C 2.81263800 2.27718500 4.46174900
H 2.81575000 0.49051700 3.25327600
C 2.13993700 3.45211400 4.82040100
H 0.35579800 4.61903600 4.49739700
H 3.78368800 2.06079600 4.89943700
H -2.32227500 -5.06196000 -2.93030100
H -0.82150100 -4.25214100 -3.42867900
H -0.93585600 -4.99375900 -1.83510000
H 3.83072900 -0.35243900 -2.15811000
H 2.43416800 0.74188600 -2.32951200
H 2.71355200 -0.09395900 -0.80968800
C 1.34098100 -2.76221300 -4.99315300
H 2.02109000 -3.59624400 -4.79902500
H 0.34298100 -3.04039600 -4.64324000
H 1.28453100 -2.62687500 -6.08064800
C 3.22501100 -1.10182700 -4.85353500

H	3.18765700	-1.01445900	-5.94694300
H	3.56950800	-0.14162200	-4.46036500
H	3.97079900	-1.85867700	-4.60833300
C	0.86238800	-0.32097400	-4.73970400
H	-0.16681800	-0.54916000	-4.45073700
H	1.13547100	0.63174800	-4.27560900
H	0.89292500	-0.18543800	-5.82833400
C	3.99037100	-3.34766900	-2.44802800
H	4.52158900	-4.13206700	-1.89436100
H	3.87705200	-3.69532900	-3.47919300
H	4.62924700	-2.45997000	-2.44622600
C	1.76495800	-4.35096200	-1.86958200
H	2.31898900	-5.20945000	-1.46980200
H	0.86333600	-4.22637400	-1.26827500
H	1.47218300	-4.59096400	-2.89431500
C	2.87221100	-2.81580100	-0.26753800
H	3.63153100	-2.05093500	-0.09326600
H	1.95613800	-2.49680600	0.23956900
H	3.21802200	-3.74234800	0.20853600
C	-3.44360100	-4.84613700	-0.31614000
H	-3.87352000	-5.09499000	0.66179000
H	-4.23870900	-4.94279700	-1.05881300
H	-2.67774000	-5.59551500	-0.53186800
C	-3.97400200	-2.46757200	0.23472500
H	-4.26513100	-2.74875700	1.25405100
H	-3.64938900	-1.42829500	0.26200500
H	-4.86804000	-2.53296700	-0.39134500
C	-1.70908100	-3.41134600	0.77978600
H	-0.87130200	-4.03826700	0.45365300
H	-1.32498000	-2.40560700	0.96463000
H	-2.07050100	-3.81484600	1.73386800
C	-4.60169500	-3.37590800	-3.21134500
H	-5.20125700	-3.34149200	-2.29896700
H	-5.25934500	-3.10729900	-4.04741000
H	-4.27867800	-4.40829000	-3.37282000
C	-3.97315800	-0.96878400	-2.92610000
H	-4.74428000	-0.75158600	-3.67573500
H	-4.42920400	-0.85732100	-1.93965100
H	-3.18624800	-0.21892600	-3.03000800
C	-2.72963900	-2.38863700	-4.54638000
H	-1.84011400	-1.75219500	-4.55145700
H	-2.43591100	-3.39371300	-4.86124000
H	-3.42501000	-1.99742600	-5.29908900
C	2.73120600	4.40523500	5.82878400
H	3.82407800	4.34841800	5.83853400
H	2.44191400	5.43998000	5.61833100
H	2.38120400	4.16773300	6.84182300
O	4.16916900	-0.28318700	1.42380100
H	3.20283600	-0.40295800	1.39120500
O	4.25607800	2.51527900	1.24863400
H	4.28990700	0.68635500	1.29782400
H	3.57013300	2.67362400	0.55170200
H	3.78259000	2.66989400	2.08165500
O	2.23705100	2.76421300	-0.56264200
H	1.48759700	2.31286500	-0.07911000
H	1.89142700	3.64996500	-0.75168200

H_2O

O	-1.78973400	0.70678400	0.00000000
H	-0.82199100	0.75696000	0.00000000
H	-2.06547100	1.63576200	0.00000000

Sol

O	2.97719300	1.95587800	20.10825300
C	1.82373700	2.75503100	19.94436800
H	1.64076900	2.81031800	18.86757000
H	0.93514800	2.32060300	20.42372900
H	1.95830100	3.77756700	20.32448100
C	3.43585600	1.72257400	21.45457800
C	4.66973000	0.83755100	21.26117500
H	5.12514700	0.58879200	22.22524300
H	4.39215200	-0.09231900	20.75420500
H	5.41248900	1.35538600	20.64573200
C	2.37434800	0.97766000	22.27681200
H	2.04657800	0.07846600	21.74438700
H	2.78850700	0.67474600	23.24446600
H	1.49728800	1.60229900	22.47543200
C	3.83110600	3.03989500	22.13733000
H	4.30872300	2.83785400	23.10205700
H	4.53816900	3.59385300	21.51070600
H	2.96216800	3.67819100	22.32766600

L

P	0.00302200	0.53813800	-0.74475300
C	-1.56406500	-0.25666800	0.01275500
C	-0.02079800	2.19854200	0.10648600
C	1.56676000	-0.25128200	0.01735600
H	0.15249700	2.14705900	1.18650200
H	0.73839700	2.84607800	-0.33999200
H	-0.99342400	2.67023800	-0.06420200
C	1.86936100	-1.55331900	-0.74946100
H	1.94043400	-1.36975900	-1.82705100
H	2.82921400	-1.96695400	-0.41219200
H	1.10541000	-2.31798900	-0.58823000
C	2.71483100	0.73939400	-0.26840800
H	3.67541500	0.26345900	-0.03212300
H	2.74100800	1.03762000	-1.32329600
H	2.63645500	1.64467600	0.34127300
C	1.52541200	-0.53651100	1.52447800
H	2.51569300	-0.86809200	1.86643000
H	1.26030300	0.35373300	2.10431000
H	0.81440200	-1.32982900	1.77160200
C	-1.52261700	-1.78188700	-0.17594500
H	-2.50637000	-2.20957700	0.05904500
H	-1.27793900	-2.05582400	-1.20848400
H	-0.79326400	-2.25722000	0.48643400
C	-1.84327500	0.07190100	1.48819600
H	-2.78607700	-0.40127700	1.79547500
H	-1.05916900	-0.29436400	2.15389500
H	-1.95214600	1.14833700	1.65310000
C	-2.72554100	0.29955800	-0.83895200
H	-2.61167200	0.03199500	-1.89455000
H	-3.67822600	-0.11400800	-0.48191900
H	-2.79667000	1.39125500	-0.77376800

bpy

C	4.05499600	0.83423400	0.39960300
C	5.36902600	1.44926900	0.95912600
C	6.24391600	1.71084000	-0.33027000
C	5.94467100	0.48586700	-1.23908100

H	3.49237800	0.27092200	1.15513600
H	3.36756300	1.63224100	0.06888800
H	6.19595800	0.66429300	-2.29238200
H	6.56686200	-0.37537500	-0.93782300
C	3.38448200	-2.56063300	-1.61205900
C	2.49392700	-0.61103100	-2.94943400
C	2.57973100	-3.07479000	-2.83937600
H	2.80778200	-2.71572300	-0.68360200
H	4.32373200	-3.10719100	-1.45778900
C	1.61525100	-1.87155600	-3.18807400
H	3.12564100	-0.41138500	-3.83253800
H	1.90110400	0.30233200	-2.81119200
B	4.45427400	0.05114000	-0.92062800
B	3.49980700	-0.98861000	-1.78302600
C	1.05639800	-1.92402000	-4.61686500
H	0.49907400	-2.85183700	-4.79920000
H	0.36615400	-1.08830800	-4.78426700
H	1.84792700	-1.85171000	-5.36920600
C	0.42107800	-1.81556500	-2.21452400
H	-0.15635800	-0.90010400	-2.38891600
H	-0.25639800	-2.66445600	-2.35334800
H	0.73860600	-1.80754800	-1.16570500
C	1.84177800	-4.38672200	-2.53756800
H	1.21724400	-4.70318400	-3.38288700
H	2.56246100	-5.19034400	-2.34335900
H	1.19917600	-4.30170400	-1.65569800
C	3.57132900	-3.34332100	-3.98888700
H	4.31154900	-4.08811100	-3.67388400
H	3.06611600	-3.73563100	-4.87761000
H	4.11902600	-2.44168400	-4.28542100
C	7.74346900	1.85999900	-0.03742900
H	8.28884600	2.08484400	-0.96207200
H	7.93906200	2.67953900	0.66604400
H	8.17219400	0.94462000	0.38195000
C	5.77814300	2.98148600	-1.07013000
H	6.30625300	3.06795400	-2.02696500
H	4.70437800	2.96628200	-1.28937600
H	5.98982600	3.88806700	-0.49356700
C	5.10936000	2.71305000	1.79091800
H	4.55010900	2.46108000	2.70012500
H	6.04724700	3.18945400	2.10384000
H	4.52176800	3.45327600	1.23906900
C	6.03346400	0.40515300	1.87941000
H	6.95125400	0.79018800	2.33617600
H	5.34799700	0.13731800	2.69204700
H	6.28616300	-0.51835600	1.34624800

$B_2(pin)_2$

C	2.46482700	1.40233100	2.02275000
C	3.68874700	2.01042200	1.25295700
C	3.23639800	-2.92016900	-2.25250500
C	1.90086000	-2.27019000	-2.75678100
B	2.90492600	0.16794100	0.14993500
B	2.76146200	-1.05089800	-1.02519700
C	1.80763200	-2.07434100	-4.26529500
H	1.85281900	-3.03940400	-4.78232300
H	0.85459500	-1.59856000	-4.51666100
H	2.61364700	-1.43970600	-4.63937700
C	0.64679900	-2.97725800	-2.23371400
H	-0.22679900	-2.35542100	-2.45031800
H	0.50590900	-3.95172600	-2.71208400
H	0.69933500	-3.12393400	-1.15065100
C	3.16509600	-4.42017200	-1.99183200
H	2.93051500	-4.96081600	-2.91555500
H	4.13289800	-4.77685800	-1.62589900
H	2.40810800	-4.66299900	-1.24323400
C	4.44313900	-2.59121200	-3.13673000
H	5.35644000	-2.89686800	-2.61782300
H	4.39488700	-3.11973800	-4.09410400
H	4.50725300	-1.51677100	-3.33349300
C	4.91030800	2.30699300	2.11493300
H	5.71426200	2.70718300	1.48934300
H	4.66945600	3.05447800	2.87901600
H	5.28119800	1.40753300	2.61056700
C	3.32519900	3.23372500	0.40612400
H	4.16468300	3.46953700	-0.25466900
H	2.44740100	3.03601400	-0.21656300
H	3.12057800	4.10847800	1.03151300
C	1.38801600	2.40602600	2.41825000
H	0.57164800	1.88757900	2.93062300
H	1.79535500	3.15905600	3.10214200
H	0.97301800	2.91442300	1.54564400
C	2.86979300	0.55195900	3.23046000
H	3.24253900	1.17211600	4.05175400
H	1.99457600	-0.00116100	3.58402800
H	3.64412100	-0.17239900	2.96028900
O	1.93221900	-0.95882200	-2.11660500
O	3.45534300	-2.23565400	-0.98240600
O	4.02707100	0.94316200	0.31629100
O	1.90478800	0.48313100	1.03774700

$[B(pin)B(pin)(OH)]^-$

O	2.08235300	-1.04340800	0.30363400
H	1.38766500	-1.71980800	0.24451700
C	6.49301200	0.29369800	-0.00924100
C	6.14792600	1.06916100	-1.32331100
C	3.24286100	-3.17418600	-2.26222700
C	2.08667900	-2.28443100	-2.84807400
B	4.38436100	-0.31438600	-0.73497400
B	2.94644300	-1.27345700	-0.85356700
C	2.16735300	-2.05665600	-4.36237200
H	2.12560700	-3.00318900	-4.91839500
H	1.32592400	-1.43392300	-4.69062100
H	3.09147500	-1.53623200	-4.62525200
C	0.69321000	-2.85845700	-2.51095000
H	-0.05758200	-2.08882700	-2.72209300
H	0.44457800	-3.75516700	-3.09460700
H	0.62967200	-3.11104000	-1.44782500
C	2.96255800	-4.68158200	-2.28483200
H	2.79919700	-5.04760100	-3.30729200
H	3.81844600	-5.22369100	-1.86424300
H	2.08422000	-4.92738100	-1.68156300
C	4.58971400	-2.90847700	-2.97006700
H	5.38641000	-3.37843000	-2.38028900
H	4.62245700	-3.32436100	-3.98553200
H	4.79251900	-1.83569500	-3.02011600
C	7.23161800	1.03954400	-2.39876400
H	6.88524300	1.58282700	-3.28467500
H	8.15285500	1.51776700	-2.04378500

H	7.46337100	0.01582000	-2.70162100
C	5.70725100	2.51646400	-1.06622800
H	5.27337800	2.92104300	-1.98604800
H	4.94020700	2.55273300	-0.28715900
H	6.54636000	3.15462700	-0.76578700
C	7.06945400	1.14951400	1.11601300
H	7.25375200	0.52397200	1.99582300
H	8.02209100	1.60363700	0.81632600
H	6.37958100	1.94478000	1.40790600
C	7.39786400	-0.92188200	-0.25307600
H	8.42438700	-0.62734200	-0.50069100
H	7.41879600	-1.53108300	0.65601500
H	7.00427900	-1.54365100	-1.06271300
O	2.25611800	-1.05584300	-2.18170100
O	3.32230100	-2.74174300	-0.92451700
O	4.99349000	0.35421200	-1.80466700
O	5.20577300	-0.21434500	0.39381400

HO-Bpin

O	2.88298800	-0.44513500	0.05973000
H	3.40180500	-0.80674200	0.79285000
C	3.22639300	-3.15255500	-2.26290500
C	2.05645000	-2.29542300	-2.87525700
B	2.79877500	-1.34587700	-0.95918100
C	2.18814700	-2.00372000	-4.36519400
H	2.18496200	-2.93574700	-4.94104600
H	1.34132100	-1.39544800	-4.69785700
H	3.10705900	-1.45733900	-4.58739800
C	0.66995700	-2.86837300	-2.56575500
H	-0.08758800	-2.12590400	-2.83326100
H	0.47592400	-3.78241000	-3.13563800
H	0.56421700	-3.09396000	-1.49997200
C	2.95323800	-4.64945100	-2.18397000
H	2.79503000	-5.06436800	-3.18544700
H	3.81302000	-5.15889700	-1.73752400
H	2.07447200	-4.86540200	-1.57279900
C	4.57666600	-2.89087100	-2.93696200
H	5.36851100	-3.34789000	-2.33586800
H	4.61308700	-3.32185200	-3.94221100
H	4.77941100	-1.81790100	-3.01158000
O	2.17068100	-1.04478500	-2.14219700
O	3.31924800	-2.62149800	-0.90989600

OH^-

O	0.01122300	1.75325500	0.00000000
H	-0.31703600	2.68023100	0.00000000

Subs

C	-0.27020800	-0.80765400	-0.79177700
C	0.44299000	-0.70535700	0.51670700
H	-0.54075900	0.15262200	-1.22661400
H	0.60425300	0.29379200	0.91363900
H	0.35698000	-1.50863400	1.24294200
C	-1.15917600	-1.94607700	-1.15036200
C	-2.45205100	-1.68345600	-1.61672400
C	-0.74002700	-3.27397100	-1.00371100
C	-3.32003700	-2.73259500	-1.92148700
H	-2.77859200	-0.65411600	-1.74148700
C	-1.60640700	-4.32159200	-1.31107000
H	0.27250000	-3.47577100	-0.66866200
C	-2.89976400	-4.05472200	-1.76787100
H	-4.32090200	-2.51613700	-2.28382700
H	-1.27006000	-5.34853600	-1.19949400
H	-3.57294100	-4.87245700	-2.00861400
N	1.18260400	-1.08580800	-0.69618800
S	2.20375900	0.10171500	-1.38609200
O	2.36342100	-0.27360600	-2.79383000
O	1.73534700	1.45560500	-1.03918200
C	3.70457000	-0.22918300	-0.49365100
C	4.45598900	-1.36001900	-0.82493800
C	4.10067600	0.63454400	0.52506400
C	5.61896800	-1.62402300	-0.11111400
H	4.13416700	-2.01014900	-1.63093000
C	5.27218900	0.35317200	1.22757200
H	3.50685400	1.51312200	0.74998500
C	6.04320100	-0.77565200	0.92522200
H	6.21269400	-2.49881900	-0.36225900
H	5.59344100	1.02353600	2.01971600
C	7.30272900	-1.08617400	1.69411800
H	7.56536900	-0.27357800	2.37727500
H	8.14864800	-1.25056900	1.01711500
H	7.18282300	-2.00042000	2.28841500

TS1

Pd	0.45042100	-1.87562400	-0.77208200
P	2.38585500	-1.64047100	0.53452100
P	-1.40893700	-2.57262800	-2.01013900
C	2.11759800	-2.12565200	2.35653100
C	3.11191000	0.05565700	0.69777900
C	3.82650700	-2.61931100	-0.23813400
C	-2.73341100	-3.36655700	-0.89311700
C	-0.92756000	-3.73142600	-3.44125600
C	-2.37325300	-1.25740500	-2.88705200
H	-1.68719300	-0.61762400	-3.44256800
H	-2.88960900	-0.63144800	-2.15443900
H	-3.11845500	-1.67327300	-3.57110300
H	4.08448400	0.04778600	1.19777900
H	3.23181500	0.49647600	-0.29456900
H	2.42355300	0.68654200	1.26570100
C	0.17057400	1.33125800	-0.91711100
C	-0.55183800	0.29273800	-0.15423000
H	1.21160000	1.43700700	-0.61001700
H	-0.26127100	0.10801700	0.86653200
H	-1.53694200	0.00503000	-0.47905900
C	0.02508300	1.37716900	-2.41103400
C	-0.97265900	2.13951200	-3.02121400
C	0.89080100	0.61344900	-3.20324800
C	-1.11426700	2.12767600	-4.41079400
H	-1.63130800	2.73069100	-2.39471800
C	0.74992900	0.60111600	-4.59035900
H	1.65852700	0.01314500	-2.72298300
C	-0.25763900	1.35585900	-5.19830500
H	-1.89500700	2.72172800	-4.87790600
H	1.42116600	-0.00058900	-5.19658900
H	-0.36946400	1.34528800	-6.27891600
N	-0.79738100	2.13512600	-0.17064100
S	-0.15624600	2.90974200	1.14782500
O	0.75170900	2.04066700	1.94021300
O	-1.27056700	3.56481400	1.84650500

C	0.87083900	4.16114100	0.38466900
C	2.25759100	4.02958100	0.39696500
C	0.25635000	5.22508900	-0.28243000
C	3.03660100	4.97618700	-0.27054000
H	2.71485800	3.20611000	0.93349300
C	1.04707400	6.15948200	-0.94079200
H	-0.82452800	5.31555000	-0.27541400
C	2.44743600	6.04761600	-0.95054700
H	4.11913100	4.88065200	-0.25972900
H	0.57471800	6.99116800	-1.45727300
C	-4.11768000	-3.52132400	-1.54454700
H	-4.10718100	-4.18734200	-2.40896000
H	-4.81345900	-3.94539700	-0.80896500
H	-4.52735800	-2.55731600	-1.86066000
C	-2.23219700	-4.72838100	-0.38618900
H	-1.21552700	-4.65214200	0.01585100
H	-2.88976900	-5.08366700	0.41743000
H	-2.23798800	-5.48725800	-1.17406500
C	-2.87503100	-2.42541900	0.32111600
H	-3.60052000	-2.85038400	1.02649900
H	-1.91874300	-2.29997600	0.83794600
H	-3.24229700	-1.43341600	0.03580700
C	3.36558200	-4.04861800	-0.57327500
H	2.43688100	-4.03186900	-1.14977700
H	4.13645400	-4.54820500	-1.17438600
H	3.19548500	-4.65466500	0.31857000
C	4.13974200	-1.90726600	-1.57096500
H	4.87797800	-2.49582800	-2.13012800
H	3.23914700	-1.81678300	-2.18959200
H	4.55932400	-0.90815100	-1.42041600
C	5.10014800	-2.67872300	0.61633500
H	5.90575900	-3.14750700	0.03628900
H	5.44825900	-1.68518300	0.91580100
H	4.95581800	-3.27962300	1.51833900
C	1.95419500	-3.64907800	2.47107600
H	1.58157500	-3.90040300	3.47225500
H	1.23444700	-4.02776600	1.73645400
H	2.90275100	-4.17497100	2.33219400
C	3.22129700	-1.63987200	3.31121800
H	2.98064500	-1.96886100	4.33031700
H	4.20648000	-2.03860000	3.06060800
H	3.28670300	-0.54829500	3.33024100
C	0.78981700	-1.46451500	2.77974600
H	-0.04336700	-1.84191500	2.17827800
H	0.58911300	-1.70452600	3.83199500
H	0.81869000	-0.37393500	2.69090400
C	0.07015200	-4.78676100	-2.93572000
H	0.42107800	-5.39062400	-3.78272200
H	0.93663400	-4.30642200	-2.47409600
H	-0.36985300	-5.46447300	-2.20136800
C	-2.10931400	-4.42737800	-4.13035400
H	-1.74751500	-4.96024300	-5.01933500
H	-2.58187800	-5.16551300	-3.47707500
H	-2.87457300	-3.71825700	-4.46166200
C	-0.19046500	-2.84368600	-4.46449800
H	0.62346800	-2.28669800	-3.98948200
H	0.24008200	-3.48001000	-5.24829600
H	-0.85517700	-2.12193900	-4.94670300
C	3.28796300	7.06140700	-1.68630200
H	3.12012100	6.99989000	-2.76878500
H	3.03675700	8.08324100	-1.37927000
H	4.35500000	6.90440200	-1.50343000

TS2

Pd	-0.94772600	-0.39864900	-0.08684500
P	-1.87194700	0.73703600	1.72501300
P	-0.42155900	-1.90820900	-1.80103300
C	-3.77237700	0.78477200	1.61863800
C	-1.45960100	2.53439400	1.89400400
C	-1.29588900	-0.00025800	3.38120800
C	-2.04386300	-2.48447100	-2.61408800
C	0.62548600	-3.38637300	-1.22507300
C	0.55393500	-1.29152000	-3.25028600
H	1.57154500	-1.06384700	-2.92312700
H	0.10440400	-0.36687300	-3.62017700
H	0.59084400	-2.01567300	-4.06933900
H	-1.79955900	2.96026600	2.84255600
H	-0.38174900	2.68098200	1.80853700
H	-1.91789100	3.08195700	1.06695700
C	1.09117900	1.72480900	-1.29838000
C	1.15792700	0.85122700	-0.10865200
H	1.09479700	1.16635700	-2.23495800
H	1.67804300	-0.08963300	-0.18452600
H	1.00400700	1.29238900	0.86172800
C	0.09608100	2.84976900	-1.32079800
C	-1.18896900	2.61284100	-1.82354000
C	0.41805300	4.11570700	-0.82782200
C	-2.14304500	3.62981900	-1.82692300
H	-1.44081100	1.62276200	-2.19329300
C	-0.53846900	5.13358700	-0.82647400
H	1.42011700	4.28728200	-0.45027300
C	-1.82116700	4.89378300	-1.32289400
H	-3.13653600	3.43662800	-2.22196000
H	-0.27971600	6.11550200	-0.43981200
H	-2.56396500	5.68663100	-1.32462000
N	2.40669100	2.06058900	-0.75250600
S	3.67266100	1.34499500	-1.55075100
O	3.37218600	-0.04989500	-1.96435600
O	4.88156400	1.58664800	-0.75112700
C	3.77272400	2.31355900	-3.05065700
C	3.25455100	1.80988900	-4.24228200
C	4.31382100	3.60080600	-2.98988500
C	3.28019700	2.60924600	-5.38589500
H	2.85355200	0.80300800	-4.27224500
C	4.33397500	4.38232900	-4.13932600
H	4.71647300	3.97325300	-2.05417700
C	3.81708300	3.90120100	-5.35341700
H	2.88059900	2.22032100	-6.31875800
H	4.75681900	5.38290500	-4.09873500
C	-1.90404800	-3.66835700	-3.58105700
H	-1.64069000	-4.59336500	-3.06083500
H	-2.86264000	-3.83990200	-4.08803500
H	-1.15297800	-3.48360400	-4.35576200
C	-3.06645400	-2.82560700	-1.51476200
H	-3.17873500	-1.98562800	-0.82177700
H	-4.04231300	-3.03170800	-1.97375100
H	-2.77947700	-3.70323000	-0.93175100
C	-2.58279300	-1.26269600	-3.38723300
H	-3.60043200	-1.47623800	-3.73750300
H	-2.62634900	-0.37596800	-2.74443900

H	-1.97404200	-1.02506300	-4.26458800
C	-1.36122300	-1.53656900	3.29324800
H	-0.80191100	-1.89684300	2.42313300
H	-0.92013600	-1.97294700	4.19897800
H	-2.38576700	-1.90551900	3.20991000
C	0.18692800	0.39397800	3.53946000
H	0.59399300	-0.09733400	4.43190200
H	0.78190600	0.06463500	2.68184400
H	0.32245400	1.47218600	3.66683400
C	-2.07469700	0.48719000	4.61146500
H	-1.59017800	0.11035400	5.52155000
H	-2.09738800	1.57968700	4.68108500
H	-3.10474800	0.12033400	4.61583800
C	-4.35602200	-0.60816700	1.90349900
H	-5.42022700	-0.61755700	1.63485000
H	-3.85347800	-1.38308800	1.31560900
H	-4.28271300	-0.87645200	2.96096500
C	-4.43385800	1.82551700	2.53786200
H	-5.52197100	1.78697500	2.39734100
H	-4.23162000	1.64464600	3.59501300
H	-4.11083900	2.84245700	2.29722000
C	-4.09235100	1.15942500	0.15684100
H	-3.68241000	0.42227100	-0.54047600
H	-5.18131900	1.20413000	0.02291700
H	-3.67771800	2.13478800	-0.11238400
C	-0.21929500	-4.31910100	-0.34251600
H	0.43886800	-5.05024900	0.14387100
H	-0.74192100	-3.76097900	0.44217500
H	-0.95926500	-4.87901300	-0.92116700
C	1.27220200	-4.18560400	-2.36949900
H	1.85478400	-5.01190500	-1.94236600
H	0.53801500	-4.61825100	-3.05228000
H	1.96222900	-3.56897000	-2.95300100
C	1.75143700	-2.79088600	-0.35334000
H	1.33814100	-2.28555600	0.52598300
H	2.40393500	-3.60265900	-0.00602100
H	2.37552400	-2.07836800	-0.90202000
C	3.86035800	4.75860300	-6.59383200
H	3.26718800	4.32209000	-7.40278500
H	3.47671700	5.76548800	-6.39391700
H	4.88944200	4.87193400	-6.95755800

TS3

Pd	-1.99689400	-0.36595500	-0.70103500
P	-1.50079700	1.61608000	-1.83744700
P	-3.11490900	-2.40929800	-0.36934500
C	0.25447900	2.18146300	-1.98437100
C	-2.37675000	3.13837200	-1.09588400
C	-1.96448600	1.40592100	-3.68198600
C	-3.63356500	-2.93636300	1.32518100
C	-2.11813900	-3.91311900	-0.97688000
C	-4.80916600	-2.32169000	-1.24897100
H	0.34249400	3.15947000	-2.46751100
H	0.71427900	2.23030200	-0.99672500
H	0.80489900	1.44790300	-2.58158500
H	-4.08313200	-3.93386300	1.33850900
H	-4.35673100	-2.21515300	1.71308000
H	-2.77271800	-2.92205300	1.99027200
C	-0.24480300	-0.03440100	1.21712700
C	0.95391700	-0.60159200	0.58082800
H	-0.39875100	1.02002300	1.04253300
H	1.22967300	-0.13173800	-0.36594200
H	1.00870800	-1.68766000	0.50231200
C	-1.01037600	-0.65321300	2.30014500
C	-2.16153900	-0.00178100	2.77659300
C	-0.59339600	-1.83666800	2.93394700
C	-2.87961400	-0.52012500	3.85088100
H	-2.48952800	0.91227000	2.29124200
C	-1.30415100	-2.34457900	4.01821500
H	0.31816600	-2.32503200	2.61017500
C	-2.45043900	-1.69111500	4.48064200
H	-3.76718300	-0.00358400	4.20465000
H	-0.95793200	-3.24905000	4.50997700
H	-3.00117200	-2.08922300	5.32772600
N	1.59521800	-0.10842400	1.80074500
S	2.38891700	1.33682500	1.67367000
O	2.81741300	1.70542500	3.02993800
O	1.63888800	2.34335500	0.88114000
C	3.84406400	0.91758600	0.71779700
C	4.88283000	0.21703100	1.33811100
C	3.90689000	1.23508000	-0.63802300
C	5.98862800	-0.16419200	0.58646900
H	4.82046200	-0.01397000	2.39603400
C	5.02318800	0.84284500	-1.37834200
H	3.10158100	1.79642000	-1.09766700
C	6.07619500	0.13992700	-0.78194900
H	6.80061100	-0.70523100	1.06573500
H	5.07845100	1.09250500	-2.43473900
C	7.29134500	-0.26680200	-1.57815100
H	7.55520600	-1.31428600	-1.39372800
H	7.12655600	-0.14014200	-2.65227300
H	8.16344900	0.33882600	-1.30083700
C	-1.63814700	3.47044200	0.21575300
H	-2.13674800	4.31880100	0.70155300
H	-1.67161500	2.63014400	0.91482100
H	-0.59054000	3.73910700	0.06387000
C	-3.82487800	2.76219900	-0.73215400
H	-3.84722100	1.87429100	-0.09177700
H	-4.29047700	3.59482400	-0.18857200
H	-4.43824600	2.55599800	-1.61232100
C	-2.37355700	4.38354000	-1.99424600
H	-2.98530700	4.24454800	-2.88946700
H	-2.79487100	5.23159800	-1.43892400
H	-1.36297800	4.66687000	-2.30544000
C	-1.36125200	2.46900600	-4.61642800
H	-1.67715400	2.25687600	-5.64632800
H	-1.68758800	3.48157600	-4.37432600
H	-0.26791200	2.45080800	-4.60208300
C	-1.40112700	0.03163200	-4.09727400
H	-1.83210100	-0.77097900	-3.49363600
H	-1.63300500	-0.15384900	-5.15445400
H	-0.31236400	-0.01595100	-3.98240600
C	-3.49109600	1.38108300	-3.85307600
H	-3.93444200	2.36884500	-3.70060500
H	-3.73884000	1.06252200	-4.87401500
H	-3.96512100	0.68093300	-3.15951300
C	-1.03942400	-4.16524100	0.09511300
H	-0.33018500	-4.91662800	-0.27447500
H	-0.47927000	-3.25275100	0.31634600
H	-1.46409100	-4.53878000	1.03104500

C	-1.40177000	-3.54085300	-2.28758400
H	-0.78830700	-2.64381900	-2.15305800
H	-0.74944700	-4.36840300	-2.59634400
H	-2.10217500	-3.34593900	-3.10319300
C	-2.92876800	-5.20203800	-1.17297400
H	-2.24438400	-6.03062200	-1.39799400
H	-3.49081400	-5.47726900	-0.27454500
H	-3.63058300	-5.12373300	-2.00750300
C	-4.62255900	-2.34921900	-2.77384800
H	-4.31735400	-3.33657400	-3.13145900
H	-5.57302600	-2.10021400	-3.26347400
H	-3.87568800	-1.62065400	-3.10081700
C	-5.80255500	-3.41907300	-0.83126100
H	-6.73883200	-3.28344500	-1.38839100
H	-5.43611700	-4.42482500	-1.04327600
H	-6.04712400	-3.36230400	0.23324300
C	-5.40523800	-0.95621800	-0.84990900
H	-5.53856600	-0.87137700	0.23444200
H	-4.75843400	-0.13359800	-1.16718600
H	-6.39094700	-0.83453400	-1.31840600

TS4

Pd	0.43256600	-1.88963700	-0.73953600
P	2.65502400	-1.54324600	0.00464800
P	-1.65148000	-2.88674300	-1.16291000
C	2.73773200	-2.27873700	1.76231700
C	3.32979100	0.15754700	0.28142200
C	3.97522400	-2.31783200	-1.12879400
C	-2.56867500	-3.29817600	0.45875900
C	-1.49371000	-4.43854700	-2.25433600
C	-2.91036900	-1.87528400	-2.07241900
H	-2.44332200	-1.43098000	-2.95313800
H	-3.25915900	-1.06184800	-1.42995200
H	-3.77840900	-2.46459600	-2.38208000
H	4.37082600	0.13663000	0.61651000
H	3.28021800	0.72378700	-0.65042500
H	2.72014000	0.67569700	1.02552000
C	0.21920100	1.31773000	-0.89560400
C	-0.46610900	0.26533800	-0.12137100
H	1.26626200	1.43378400	-0.62608000
H	-0.14131100	0.08740200	0.89186200
H	-1.46647600	-0.01094000	-0.40656400
C	0.03396000	1.39586700	-2.37971400
C	1.14506700	1.59213400	-3.20491200
C	-1.23228300	1.26644600	-2.96306800
C	1.00237500	1.63006900	-4.59516600
H	2.12913500	1.70634700	-2.75862200
C	-1.37866400	1.29707600	-4.34855100
H	-2.10329400	1.14865200	-2.32728100
C	-0.26030500	1.47181000	-5.17030200
H	1.87563200	1.77516500	-5.22427700
H	-2.36513500	1.18682600	-4.78965900
H	-0.37401800	1.49111500	-6.25002100
N	-0.72651700	2.12246400	-0.10551800
S	-0.01457000	2.84954700	1.20972800
O	0.95080800	1.95775200	1.90368200
O	-1.09101900	3.46099600	2.00163200
C	0.94907200	4.14770900	0.44206200
C	2.33040400	4.01377000	0.31833700
C	0.28400700	5.25266400	-0.09758300
C	3.05148600	4.99886500	-0.35855700
H	2.83060400	3.15961600	0.76005400
C	1.01779900	6.22589600	-0.76552100
H	-0.79122000	5.34235900	0.01251000
C	2.41047000	6.11293900	-0.91134800
H	4.12982100	4.90147400	-0.45307100
H	0.50577000	7.08898800	-1.18319800
O	1.14879600	-1.65984000	-3.85287500
H	0.95083800	-1.75546200	-2.89027100
H	0.89576200	-0.73980200	-4.03727200
C	-4.05121300	-3.66806400	0.27768900
H	-4.19635300	-4.57607800	-0.30678800
H	-4.49706500	-3.83624200	1.26635900
H	-4.61465300	-2.85965100	-0.19828300
C	-1.82431200	-4.42573700	1.19303300
H	-0.75796000	-4.19772700	1.29647800
H	-2.24372200	-4.54501900	2.19999600
H	-1.92136100	-5.38633200	0.68054400
C	-2.51232200	-2.03337800	1.33911700
H	-3.02756600	-2.23413300	2.28640100
H	-1.48518300	-1.74495500	1.56819600
H	-3.01290400	-1.17843600	0.87281500
C	3.43103900	-3.63637800	-1.70601300
H	2.52568600	-3.45960600	-2.29160500
H	4.18287000	-4.07811500	-2.37278600
H	3.19806800	-4.37016900	-0.93059600
C	4.18607900	-1.33766800	-2.30123400
H	4.83195800	-1.81774200	-3.04748200
H	3.24399500	-1.08905900	-2.79850500
H	4.68364300	-0.41708000	-1.98120700
C	5.32851100	-2.57003100	-0.44853300
H	6.05206300	-2.90347300	-1.20357300
H	5.73391500	-1.66507800	0.01503300
H	5.26862500	-3.35090100	0.31402300
C	2.61603900	-3.80931900	1.68401500
H	2.46346200	-4.21503800	2.69237200
H	1.76136200	-4.10607800	1.06604000
H	3.51578300	-4.27598800	1.27337900
C	3.98470400	-1.88625200	2.57259600
H	3.91909700	-2.34565400	3.56720600
H	4.91569200	-2.22455200	2.11542000
H	4.04619200	-0.80399000	2.72009100
C	1.49873500	-1.73239800	2.50195700
H	0.57977300	-2.07622700	2.01880000
H	1.50309300	-2.10171300	3.53573300
H	1.47815800	-0.63818900	2.53991600
C	-0.22872800	-5.20514300	-1.82654800
H	-0.09077800	-6.07453300	-2.48225000
H	0.65738500	-4.57060300	-1.90554200
H	-0.29054900	-5.56715500	-0.79668000
C	-2.70217500	-5.38701400	-2.22456100
H	-2.55227000	-6.17222800	-2.97669200
H	-2.82169800	-5.88231900	-1.25764100
H	-3.63734100	-4.87394200	-2.47106500
C	-1.30441100	-3.95182200	-3.70580200
H	-0.50762800	-3.21005900	-3.80596600
H	-1.04007000	-4.81424700	-4.33118600
H	-2.22810600	-3.52638000	-4.11100700
C	3.18775800	7.17563600	-1.64760000

H 4.25976000 6.95786800 -1.64995800
H 2.85664100 7.25552900 -2.69016900
H 3.04347000 8.16075900 -1.18822900

TS5
Pd -0.94925900 -0.32322500 -0.03328800
P -1.98042800 0.82719800 1.71141800
P -0.39579400 -1.93424900 -1.65016400
C -3.66488100 1.55112100 1.20948400
C -1.11650200 2.32183900 2.38684100
C -2.12674200 -0.28987600 3.24436500
C -1.98241900 -2.40179200 -2.59478600
C 0.42644200 -3.46677900 -0.87395900
C 0.76632600 -1.52549400 -3.03324300
H 1.69021200 -1.11571600 -2.62101300
H 0.30757800 -0.76512900 -3.67220100
H 1.00501800 -2.39850700 -3.64818600
H -1.60775500 2.72909400 3.27444100
H -0.08675000 2.06763900 2.64771600
H -1.08457400 3.09114300 1.61071400
C 1.09439400 1.68281100 -1.34145200
C 1.11329100 0.93980500 -0.06436700
H 1.16845600 1.02971600 -2.21077000
H 1.66616400 0.01359900 -0.01946600
H 0.89818100 1.46876100 0.84954600
C 0.09983000 2.78086500 -1.57503700
C -0.84373700 2.65236100 -2.59815400
C 0.11094400 3.94489900 -0.79882300
C -1.77258400 3.66525800 -2.83921300
H -0.84803900 1.75441300 -3.20969500
C -0.81963200 4.95746800 -1.03480200
H 0.87050000 4.05922100 -0.03234200
C -1.76447400 4.82044700 -2.05569000
H -2.49929200 3.55282700 -3.63895500
H -0.80012400 5.85939700 -0.42915200
H -2.48537700 5.61145800 -2.24183100
N 2.37216600 2.11534300 -0.75886600
S 3.68775500 1.37352500 -1.44846100
O 3.48258300 -0.08355700 -1.64814800
O 4.87447600 1.81216000 -0.70053700
C 3.71606300 2.11607900 -3.07758900
C 3.37227800 1.36019600 -4.19572800
C 4.01455800 3.47685000 -3.19500500
C 3.32708100 1.97755100 -5.44713900
H 3.15614400 0.30394500 -4.08328400
C 3.96554900 4.07567800 -4.44820300
H 4.27999500 4.05032300 -2.31346700
C 3.61713900 3.33841400 -5.59230800
H 3.06354700 1.39092700 -6.32331100
H 4.19870000 5.13292900 -4.54548300
C -1.76050300 -3.23260900 -3.86967000
H -1.31727400 -4.20897000 -3.66799100
H -2.72858300 -3.40393400 -4.35848500
H -1.12185800 -2.71018100 -4.58824500
C -2.94526800 -3.13473400 -1.64690400
H -3.07195600 -2.58328500 -0.70837900
H -3.92979100 -3.22879600 -2.12303800
H -2.59616300 -4.14369600 -1.40943300
C -2.63043400 -1.06138500 -3.00087400
H -3.56405800 -1.25835900 -3.54410800
H -2.85724500 -0.44878100 -2.12297100
H -1.98128300 -0.47531300 -3.66092000
C -2.68481400 -1.66235600 2.82535100
H -2.10020600 -2.08634100 2.00435200
H -2.63416800 -2.35045400 3.67935700
H -3.72719800 -1.60775800 2.50476100
C -0.68047300 -0.50207200 3.74010600
H -0.67581900 -1.27365000 4.52002500
H -0.02792500 -0.83884500 2.92612400
H -0.25283000 0.40708300 4.17365500
C -2.97503200 0.28372200 4.38786300
H -2.88173800 -0.36358600 5.26964500
H -2.65291500 1.28744900 4.68345300
H -4.03601800 0.32500300 4.12586000
C -4.68880600 0.42123100 1.01616400
H -5.57898800 0.82121300 0.51405900
H -4.28470900 -0.38489700 0.39370600
H -5.01395400 -0.00649500 1.96891100
C -4.22235900 2.60267500 2.18339900
H -5.18837000 2.96292300 1.80649800
H -4.38689600 2.20628400 3.18710600
H -3.56088400 3.47037200 2.26276700
C -3.42202500 2.23040000 -0.15141300
H -3.07744100 1.51289500 -0.90018400
H -4.35779000 2.68335100 -0.50500300
H -2.67245100 3.02345100 -0.09318000
C -0.25794400 -3.77950100 0.46790300
H 0.24982400 -4.62702100 0.94702200
H -0.19944800 -2.91650500 1.13783300
H -1.31207100 -4.04311900 0.35113500
C 0.42399500 -4.71821400 -1.76273000
H 1.03628700 -5.49803600 -1.29159200
H -0.58215500 -5.12687900 -1.89117500
H 0.84722600 -4.52352700 -2.75320300
C 1.88572800 -3.06732000 -0.57507100
H 1.94199600 -2.14457200 0.01026400
H 2.35996900 -3.86368400 0.01215300
H 2.47724300 -2.92431800 -1.48263100
C 3.55589700 4.01032300 -6.94165400
H 3.32320800 3.29414600 -7.73519200
H 2.78715500 4.79264400 -6.95741100
H 4.50960000 4.49169300 -7.18830500

TS6
Pd -0.20631900 2.30948200 -0.83286800
P -1.80268800 3.30084900 0.62442400
P 1.29451200 2.00203500 -2.58945300
C -1.97406800 2.69332400 2.36417600
C -3.57577800 3.14711000 -0.05582500
C -1.35073200 5.13749100 0.89865500
C 0.69560100 2.98861000 -4.10200800
C 1.46796700 0.29085400 -3.28468000
C 3.09027500 2.42715200 -2.13776400
H 0.48515200 -0.18120000 -3.35275600
H 1.93555000 0.29019800 -4.27324000
H 2.08597400 -0.31701100 -2.61844700
H -2.85996800 3.10041600 2.86036400
H -2.01684900 1.60352900 2.38340000
H -1.08589200 2.98785100 2.92725700
O 1.37777800 2.18740900 2.31319100

H	0.78998300	1.46678800	2.62216200
H	0.99407600	2.45546600	1.45716600
C	-1.09480000	-0.62457500	0.20182400
C	0.18918600	0.06153200	-0.05141500
H	-1.73756700	-0.10074900	0.91239900
H	0.68300500	0.56661700	0.75967500
H	0.79355100	-0.32094100	-0.85733800
C	-1.86510500	-1.15466500	-0.97769700
C	-2.03749600	-0.37131700	-2.12339200
C	-2.44123800	-2.42708600	-0.92367600
C	-2.77937800	-0.84913000	-3.20429500
H	-1.58389800	0.61746300	-2.15082400
C	-3.18637800	-2.90511800	-2.00223300
H	-2.28970100	-3.03753800	-0.03874000
C	-3.35744700	-2.11885800	-3.14506900
H	-2.90182000	-0.23145800	-4.09018100
H	-3.63018100	-3.89559100	-1.95333400
H	-3.93386400	-2.49615100	-3.98513600
N	-0.18207400	-1.60603400	0.79061900
S	-0.05982500	-1.50663100	2.44126500
O	-0.94901900	-2.50636800	3.06282300
O	-0.19920300	-0.10645900	2.94303000
C	1.63070600	-2.01346800	2.69968600
C	1.90303100	-3.36360800	2.91239400
C	2.65671900	-1.06658400	2.65483800
C	3.22592400	-3.77053800	3.07669700
H	1.08811800	-4.07767700	2.95151100
C	3.97149800	-1.49337700	2.82117800
H	2.43833200	-0.01546400	2.49986300
C	4.27714100	-2.84574900	3.03470600
H	3.44488600	-4.82211700	3.24165900
H	4.77506500	-0.76211700	2.78966800
C	5.70463100	-3.28611600	3.24426700
H	6.39506800	-2.71281000	2.61675400
H	6.01126400	-3.13418200	4.28734500
H	5.83313000	-4.34851800	3.01543600
C	0.24644200	4.38736800	-3.64481800
H	-0.16104200	4.93854800	-4.50222400
H	-0.53475000	4.30698300	-2.88342400
H	1.06589300	4.97620400	-3.22609800
C	-0.54957600	2.24670600	-4.63103600
H	-1.29087900	2.09985700	-3.83913500
H	-1.01675400	2.84915500	-5.41989300
H	-0.30379000	1.27095700	-5.05994600
C	1.72101700	3.11873600	-5.23748400
H	1.23631800	3.57553400	-6.10989900
H	2.56078600	3.76081200	-4.95830800
H	2.11836100	2.14926900	-5.55428100
C	3.22677600	3.94309000	-1.92267300
H	4.20395700	4.16048500	-1.47357500
H	3.16658800	4.49960800	-2.86268100
H	2.45441400	4.32190100	-1.24481700
C	4.13293000	1.94474500	-3.16052300
H	4.00515200	2.40296100	-4.14227300
H	5.13478600	2.20907100	-2.79895700
H	4.10884200	0.85808600	-3.28462500
C	3.36446700	1.71898800	-0.79427700
H	4.40239400	1.91009500	-0.49353100
H	2.70979700	2.08462600	-0.00002200
H	3.23962400	0.63263800	-0.86180600
C	-3.55225300	3.36746500	-1.57955200
H	-3.28394400	4.39169900	-1.84916500
H	-2.83411400	2.69521700	-2.05831600
H	-4.54782100	3.16071900	-1.99308900
C	-4.59363800	4.09648200	0.59337000
H	-4.40230400	5.14390400	0.34735000
H	-5.59798600	3.85226600	0.22397600
H	-4.61426900	3.99557100	1.68307600
C	-4.02482300	1.69730100	0.21086900
H	-4.11066100	1.48033900	1.27951800
H	-5.01336500	1.54171700	-0.23908500
H	-3.34638100	0.97165600	-0.24129300
C	-1.63084600	5.95257100	-0.37291600
H	-2.69975800	6.06687400	-0.56975100
H	-1.20826200	6.95892500	-0.25791700
H	-1.16436000	5.49346400	-1.24905000
C	0.16888600	5.17242400	1.15917000
H	0.72128700	4.77253600	0.30185800
H	0.48404400	6.21143600	1.32117900
H	0.45506900	4.59539200	2.04307500
C	-2.05927400	5.78284400	2.10225900
H	-1.72588800	6.82460500	2.19361000
H	-3.14582000	5.79275400	2.00043700
H	-1.80772600	5.27781300	3.03908400

TS7

Pd	0.64808900	1.92790800	0.17307300
P	-1.02289000	2.90375100	1.58376800
P	2.74310000	1.25234100	-0.65185900
C	-0.15462200	3.88189600	2.97649500
C	-2.06831300	1.72759000	2.56019300
C	-2.30963900	4.01919100	0.72357000
C	3.10179700	1.58402400	-2.49102900
C	3.04460900	-0.57188900	-0.50422600
C	4.11424900	1.98179300	0.45058600
H	2.50987700	-1.10245800	-1.29600300
H	4.10588100	-0.82613300	-0.57542300
H	2.65298700	-0.92068900	0.45405600
O	0.69723200	-0.40464700	2.62474100
H	-0.18747800	-0.76108000	2.40248600
H	0.78438500	0.38314500	2.04892200
H	-1.44173100	1.25002900	3.31692200
H	-2.90328500	2.22977300	3.05782700
H	-2.45260700	0.93273300	1.91983000
C	-1.71960300	-0.11122700	-0.91295900
C	-0.27215400	-0.16769800	-0.61690800
H	-2.32505100	0.23170100	-0.07144000
H	0.04672900	-0.49855500	0.35559800
H	0.38269500	-0.33136700	-1.45642000
C	-2.15801700	0.50388200	-2.21474200
C	-1.62136700	1.72188600	-2.64161800
C	-3.14194400	-0.11995200	-2.98681800
C	-2.06377400	2.31757400	-3.82289200
H	-0.85901700	2.19842400	-2.02989600
C	-3.58723200	0.47431700	-4.16798900
H	-3.54335800	-1.07355400	-2.65795300
C	-3.05011300	1.69392200	-4.58944200
H	-1.63742100	3.26453400	-4.14292500
H	-4.35202600	-0.01693200	-4.76308000
H	-3.39626800	2.15284800	-5.51130500

N -1.52965600 -1.55875200 -0.99875800
S -1.94074200 -2.37968600 0.38281000
O -3.28033800 -2.97465200 0.21844200
O -1.72918600 -1.56711000 1.61787200
C -0.73386300 -3.69377900 0.37756900
C -1.01205100 -4.86008200 -0.33463500
C 0.48346900 -3.52496300 1.03967800
C -0.05020200 -5.86614200 -0.38972400
H -1.96859200 -4.97270300 -0.83262100
C 1.43204800 -4.54306000 0.97334200
H 0.69008400 -2.61931300 1.59916500
C 1.18338000 -5.72435000 0.26055700
H -0.26005700 -6.77609000 -0.94569500
H 2.38163900 -4.41877300 1.48742000
C 2.20792700 -6.83096200 0.21993300
H 3.22545100 -6.43671400 0.30687300
H 2.05755600 -7.53375900 1.04981500
H 2.13935900 -7.40480000 -0.70976000
C 3.21276800 3.10119800 -2.71267200
H 3.21978700 3.31531500 -3.78876700
H 2.36187100 3.62983100 -2.26724400
H 4.13371000 3.51178500 -2.28981700
C 1.87846900 1.07122400 -3.27432100
H 0.95850800 1.56544200 -2.96151700
H 2.02551300 1.27309100 -4.34272600
H 1.74008800 -0.01016100 -3.16771600
C 4.34567700 0.86825000 -3.04623100
H 4.28378400 -0.21588100 -2.91068000
H 4.40951800 1.05766800 -4.12547600
H 5.27660900 1.21580000 -2.59805000
C 3.82858700 3.47596400 0.68548000
H 4.54163800 3.87369200 1.41889900
H 3.92913500 4.06303200 -0.23062800
H 2.81785100 3.63103200 1.07007300
C 5.54261100 1.82052600 -0.08930400
H 5.72346400 2.44542100 -0.96771200
H 6.25359700 2.13484800 0.68565300
H 5.77637100 0.78223500 -0.34461900
C 4.02031400 1.23504600 1.79803200
H 4.63895800 1.75434700 2.54020200
H 2.99791700 1.19724200 2.18174200
H 4.38862400 0.20802700 1.71971100
C -3.06370600 4.98329300 1.65165100
H -2.41522600 5.75523000 2.07211300
H -3.84455300 5.49261800 1.07223600
H -3.55906000 4.45883000 2.47455100
C -1.59975000 4.82053700 -0.38348000
H -0.87986000 5.53649600 0.02163600
H -1.06339600 4.15861700 -1.06864700
H -2.34347500 5.38303300 -0.96232500
C -3.35046300 3.08816300 0.07367300
H -4.03885800 3.68961900 -0.53308400
H -2.89122600 2.35868100 -0.59179500
H -3.94642200 2.55541500 0.82076600
C 0.45395600 5.17215100 2.40305700
H -0.30717800 5.91786100 2.15913900
H 1.12709500 5.61941100 3.14546000
H 1.03763100 4.97221700 1.49820500
C -1.05318500 4.20773800 4.18249800
H -0.45384000 4.73841400 4.93349900
H -1.90047600 4.84464300 3.92942500
H -1.43575700 3.29938600 4.65669200
C 0.99479500 2.98926100 3.48279800
H 0.64715000 2.01532300 3.83939100
H 1.72864400 2.81161300 2.69574900
H 1.50176600 3.49129600 4.31710000

TS8
Pd -0.84432700 -0.34679300 0.08661300
P -0.42924800 -0.82520100 2.33077500
P -1.78843500 -0.45183700 -2.09732500
C -1.31793400 0.35871900 3.52919600
C 1.33834400 -0.70250100 2.86350300
C -0.87205700 -2.63636100 2.72515700
C -3.68481100 -0.38670200 -1.89683500
C -1.23719500 -2.03398200 -2.99769300
C -1.53338100 0.83396100 -3.41046200
H -0.47064400 1.02202100 -3.56192400
H -1.99093600 1.77291900 -3.09499500
H -1.97138200 0.52605700 -4.36393200
H 1.50978300 -1.13494000 3.85338600
H 1.95879500 -1.21784900 2.12602400
H 1.64149500 0.34819200 2.88470000
C 1.04321300 1.87730400 -1.27030300
C 1.07477200 1.18592400 0.02650100
H 1.09923500 1.19169500 -2.11860000
H 1.65986100 0.28839400 0.12856700
H 0.84514500 1.75237500 0.91656300
C 0.06938900 2.99208500 -1.52237600
C -1.12624100 3.09641000 -0.81001900
C 0.34715200 3.91943500 -2.53479900
C -2.04207200 4.10918700 -1.10613500
H -1.35619800 2.35454400 -0.05249700
C -0.56698000 4.92726800 -2.83461800
H 1.27928900 3.84734800 -3.08612700
C -1.76638800 5.02521200 -2.12155200
H -2.97010000 4.17706800 -0.54503800
H -0.34377400 5.63959700 -3.62405000
H -2.47727700 5.81276500 -2.35497400
N 2.34774900 2.33049400 -0.76603900
S 3.61247700 1.52350700 -1.48182000
O 3.34658900 0.06891600 -1.66694100
O 4.84490500 1.92283300 -0.79155400
C 3.59329300 2.23022300 -3.12430500
C 2.97891700 1.53917300 -4.16904900
C 4.06909400 3.53118500 -3.30415800
C 2.83296000 2.16807800 -5.40539400
H 2.63038500 0.52458800 -4.01170800
C 3.91966600 4.14110600 -4.54567200
H 4.53945000 4.05298000 -2.47790900
C 3.29552600 3.47373800 -5.61211600
H 2.35312700 1.63553100 -6.22223300
H 4.28703300 5.15346000 -4.69178100
O 1.99252300 -2.04684000 -0.33168700
H 1.09142200 -1.66407800 -0.29038100
H 2.51913700 -1.37095800 -0.80371900
C -4.46189700 -0.16137200 -3.20433000
H -4.30665200 -0.96071600 -3.93101700
H -5.53522200 -0.12392600 -2.97763300
H -4.19594600 0.78827600 -3.67706200

C -4.18662700 -1.66459800 -1.20732000
H -3.62707000 -1.86751700 -0.28946500
H -5.24342900 -1.54062000 -0.93776700
H -4.11457700 -2.54077000 -1.85763500
C -3.95764200 0.80678800 -0.95846800
H -5.03648300 0.88234300 -0.76960900
H -3.44182500 0.67605400 -0.00089200
H -3.62579200 1.75476900 -1.39229600
C -2.19712800 -2.98600500 2.02570000
H -2.10922100 -2.83214300 0.94681200
H -2.43651600 -4.04226400 2.20517900
H -3.03649000 -2.38641300 2.38574200
C 0.23289000 -3.51764300 2.11027100
H -0.06513800 -4.56964800 2.20356600
H 0.39210200 -3.30684200 1.05086600
H 1.19073000 -3.39729200 2.62348100
C -0.96856500 -2.95988000 4.22360500
H -1.11163700 -4.04123100 4.34578800
H -0.05563500 -2.68846300 4.76308700
H -1.81524100 -2.46326400 4.70356900
C -2.83076200 0.08540100 3.50669400
H -3.35300400 0.88205800 4.05157000
H -3.21590900 0.07008100 2.48111300
H -3.08925700 -0.86309800 3.98481600
C -0.78728100 0.31564000 4.97253700
H -1.32698200 1.05889000 5.57346000
H -0.92625100 -0.65552000 5.44877300
H 0.27588800 0.56967000 5.01908900
C -1.08580900 1.77924400 2.97903900
H -1.49996100 1.88324100 1.97325800
H -1.58037400 2.50833300 3.63313700
H -0.02309200 2.04231700 2.94366400
C -1.18746500 -3.20707300 -2.00189000
H -0.80056900 -4.09795400 -2.51302600
H -0.52507100 -2.98743600 -1.16147200
H -2.17302500 -3.45404900 -1.60056400
C -2.10759300 -2.42914000 -4.20042200
H -1.63333000 -3.26723300 -4.72688000
H -3.10399000 -2.75932000 -3.89349500
H -2.22075100 -1.61304500 -4.92089300
C 0.20048500 -1.75895700 -3.48929600
H 0.85141800 -1.41021800 -2.68186400
H 0.63127300 -2.69114500 -3.87437100
H 0.22190300 -1.02483400 -4.30066000
C 3.15443900 4.14255500 -6.95662200
H 4.08664900 4.06498300 -7.53087900
H 2.36147000 3.68006800 -7.55214200
H 2.92778200 5.20855100 -6.84966400

TS9

Pd 1.35788500 0.56674200 0.07894300
P 0.48057200 2.12614900 1.61433100
P 2.69482900 -0.40065600 -1.58973000
C 1.78138900 2.78349500 2.83467000
C -0.87005500 1.54589900 2.74420200
C -0.36919700 3.55170200 0.68182500
C 2.15740300 0.15399200 -3.32951800
C 2.79192900 -2.24193700 -1.74520900
C 4.51277700 0.07038200 -1.26197600
H 1.78441200 -2.64785100 -1.84216300
H 3.38688600 -2.56619200 -2.60431800
H 3.22979500 -2.64905700 -0.83022000
O 2.53799800 -1.37861100 2.35615200
H 1.75926400 -1.91565300 2.57571500
H 2.21588200 -0.80726200 1.62095400
H -0.58090400 0.59131200 3.18977100
H -1.08495400 2.26174500 3.54277400
H -1.78889600 1.38985500 2.17152300
C -0.76142000 -2.00365800 -0.25735100
C -0.76677100 -0.52453500 -0.22092000
H -0.34181100 -2.41783700 -1.17385800
H -0.87632100 0.01206900 -1.15133100
H -1.11782800 -0.05203500 0.67876600
C -0.31526400 -2.75697700 0.96118700
C 0.59283500 -3.81189300 0.84087200
C -0.79490400 -2.41352800 2.23233000
C 1.04081100 -4.49823600 1.97149400
H 0.95777900 -4.09367900 -0.14202800
C -0.34919500 -3.09556500 3.36420000
H -1.52753800 -1.61913200 2.32999000
C 0.57669200 -4.13748200 3.23709700
H 1.75433800 -5.30959900 1.86315500
H -0.72385800 -2.81729400 4.34503000
H 0.92879500 -4.66412500 4.11897500
N -2.20914700 -1.75839300 -0.31202400
S -2.89508200 -1.81008800 -1.83106600
O -4.11556400 -2.62665400 -1.72427800
O -1.90889200 -2.13833500 -2.88726100
C -3.40520500 -0.11529100 -2.10610600
C -4.39858500 0.44166200 -1.29714600
C -2.77754500 0.64281700 -3.09200600
C -4.74731100 1.77678100 -1.47374400
H -4.88966400 -0.16739800 -0.54590100
C -3.14280200 1.97924900 -3.25936000
H -2.02179300 0.18468400 -3.71951800
C -4.12105400 2.56785000 -2.45041300
H -5.52016900 2.21548200 -0.84774400
H -2.65735300 2.57344600 -4.02917100
C -4.47877300 4.02548400 -2.59958600
H -3.98758800 4.62909200 -1.82460200
H -4.16417800 4.41761800 -3.57145200
H -5.55736000 4.18552800 -2.49784700
C 3.22398200 -0.00618200 -4.42238700
H 3.61195000 -1.02842600 -4.47165500
H 2.77328800 0.22036400 -5.39728600
H 4.06484800 0.67817400 -4.28487100
C 1.70323200 1.62354600 -3.26175400
H 0.89198200 1.74809300 -2.53903800
H 2.51232000 2.29808200 -2.97149600
H 1.33868600 1.93781300 -4.24852600
C 0.93974700 -0.70865200 -3.71098500
H 0.48445500 -0.30337000 -4.62345100
H 1.21655200 -1.74599000 -3.91904400
H 0.16919000 -0.72053500 -2.93875800
C 5.53926400 -0.70261500 -2.10745800
H 5.43407300 -0.52536700 -3.17805200
H 6.54834100 -0.38260000 -1.81720100
H 5.47891400 -1.78066600 -1.93013900
C 4.68842100 1.58493200 -1.46251600
H 5.67266900 1.89131800 -1.08601700

H 4.63553100 1.87197600 -2.51661400
H 3.92612800 2.14807400 -0.91294400
C 4.77891100 -0.26030100 0.21925100
H 4.61812000 -1.31750000 0.44749900
H 5.82185900 -0.01847100 0.46133000
H 4.13141300 0.31776500 0.88136500
C -1.23748100 2.89296800 -0.40691300
H -0.62939000 2.29756300 -1.09099300
H -2.01721300 2.24501700 0.00503700
H -1.74048800 3.67246100 -0.99102700
C 0.69676300 4.41137400 -0.01805400
H 1.37791900 3.79083200 -0.61105000
H 0.20456000 5.11875700 -0.69761300
H 1.28949900 4.99493900 0.69161200
C -1.28355400 4.43630100 1.54644500
H -1.75749100 5.18910300 0.90332600
H -2.08637800 3.85686700 2.01267500
H -0.74531500 4.96787400 2.33209500
C 3.09794000 3.03592500 2.07892700
H 3.86748300 3.36057300 2.79107700
H 3.44963600 2.12276000 1.59339500
H 3.00232900 3.80972300 1.31331500
C 2.02508500 1.66042300 3.86451900
H 2.25590700 0.69936400 3.39381600
H 2.88032900 1.94057300 4.49262700
H 1.16453200 1.52495200 4.52748100
C 1.36586900 4.05799800 3.58433600
H 1.30671200 4.92688800 2.92348500
H 0.40349600 3.94378500 4.09331300
H 2.11762600 4.28084000 4.35217700

TS10

Pd 0.48339600 -1.91392800 -0.77556000
P 2.65250600 -1.58865100 0.14898300
P -1.46133800 -2.56801700 -1.95136700
C 2.86620100 -1.73106400 2.04042600
C 3.35997100 0.09580600 -0.16121800
C 3.88936900 -2.70850100 -0.76533200
C -2.95270900 -3.16881300 -0.92087300
C -1.01715500 -3.81837900 -3.31031200
C -2.21996100 -1.18927600 -2.92472700
H -1.44213600 -0.67235500 -3.48287700
H -2.67527100 -0.46187600 -2.24798000
H -2.98772000 -1.54703400 -3.61501800
H 4.43410700 0.13112600 0.03627200
H 3.18838800 0.38683300 -1.19892800
H 2.86265300 0.82236000 0.48588400
C 0.22597600 1.30573900 -0.94537500
C -0.44015400 0.22283800 -0.17844000
H 1.26184900 1.45002300 -0.65065900
H -0.13725100 0.06066600 0.84488400
H -1.44591700 -0.04075600 -0.45565400
C 0.07098700 1.33599600 -2.43980000
C 0.88859700 0.51093000 -3.22289800
C -0.87700500 2.14983200 -3.06136000
C 0.75020900 0.48998000 -4.60984800
H 1.60961300 -0.14011500 -2.73842800
C -1.01671500 2.12964500 -4.45135600
H -1.49878500 2.78956500 -2.44479900
C -0.20773400 1.29866200 -5.22877500
H 1.38312600 -0.16157700 -5.20576100
H -1.75905100 2.76469900 -4.92687600
H -0.31908400 1.28250300 -6.30927600
N -0.73562600 2.10314000 -0.19295700
S -0.11260700 2.83187000 1.16454800
O -0.11073800 4.29169300 0.95772800
O 1.15490400 2.20213100 1.61945500
C -1.36573200 2.44097200 2.37553800
C -2.64971700 2.97199700 2.22631100
C -1.05919700 1.59618100 3.43887300
C -3.63364500 2.63378300 3.14857300
H -2.86956800 3.63614300 1.39754800
C -2.05793200 1.27021600 4.35794500
H -0.05329900 1.20773000 3.54470200
C -3.35511700 1.77793500 4.22716200
H -4.63519700 3.04089000 3.03525200
H -1.82248600 0.61345400 5.19122300
C -4.29940700 -3.04384200 -1.65623300
H -4.33482300 -3.63955400 -2.57081200
H -5.09727700 -3.40561100 -0.99510700
H -4.53495200 -2.00748000 -1.91348100
C -2.76685000 -4.62239300 -0.45539600
H -1.80622100 -4.78149800 0.04167800
H -3.56297900 -4.86812500 0.25988800
H -2.85236100 -5.32857500 -1.28616600
C -2.99641400 -2.25368600 0.31999500
H -3.80735500 -2.57854300 0.98411800
H -2.05494700 -2.28558700 0.87695700
H -3.19468300 -1.20984000 0.05294900
C 3.39878100 -4.16565500 -0.72361100
H 2.37834600 -4.24254600 -1.10742500
H 4.04771100 -4.78511600 -1.35608800
H 3.40929100 -4.58699400 0.28258400
C 3.86447300 -2.24631000 -2.23736400
H 4.46563000 -2.93640700 -2.84248300
H 2.84263200 -2.25114600 -2.63374400
H 4.28178400 -1.24317300 -2.36669000
C 5.32928300 -2.63069300 -0.23743600
H 5.98281900 -3.23323800 -0.88135400
H 5.72013200 -1.60847500 -0.24292600
H 5.41600300 -3.02628400 0.77761300
C 2.95160600 -3.19746400 2.49246700
H 2.89940700 -3.23044700 3.58883400
H 2.13248600 -3.80337900 2.09668300
H 3.89993200 -3.65813500 2.20186600
C 4.09056400 -0.96530100 2.57562100
H 4.14831600 -1.11134800 3.66177700
H 5.02995200 -1.32157100 2.14578100
H 4.01568500 0.11023800 2.39528300
C 1.59834700 -1.09517200 2.64471900
H 0.69519000 -1.63033500 2.33156700
H 1.65947800 -1.13606700 3.74031300
H 1.49544900 -0.04518500 2.35559500
C -0.21549700 -4.97722500 -2.69271800
H 0.10836800 -5.65981700 -3.48894000
H 0.67619300 -4.60209200 -2.18352200
H -0.79434900 -5.55443100 -1.96970300
C -2.22313500 -4.37252400 -4.08346400
H -1.86445200 -4.99299400 -4.91474400
H -2.85828800 -5.00303600 -3.45555000

H	-2.84214400	-3.57793000	-4.51194200
C	-0.08852800	-3.06944700	-4.28765900
H	0.74431000	-2.59630300	-3.75685100
H	0.32759000	-3.78601900	-5.00705500
H	-0.61280500	-2.29420000	-4.85310100
C	-4.43259700	1.43246200	5.22505500
H	-4.09330400	0.66964900	5.93206000
H	-4.73167200	2.31562600	5.80315100
H	-5.33216300	1.05643600	4.72402400

TS11

Pd	-1.28557500	0.00498700	0.00140500
P	-1.92415200	0.46496200	2.17987100
P	-1.21055400	-1.00684700	-2.14212000
C	-3.61099500	1.34234600	2.28635800
C	-0.84509700	1.60418500	3.16763300
C	-1.90026500	-1.12711100	3.21790600
C	-3.02258200	-1.33489200	-2.63907800
C	-0.18677000	-2.61204200	-2.12680300
C	-0.58360900	-0.15672000	-3.66686100
H	0.44011200	0.18739900	-3.52243400
H	-1.19665900	0.72571700	-3.86203800
H	-0.61127700	-0.81481100	-4.53990200
H	-1.09417000	1.60707400	4.23224000
H	0.20094600	1.31332200	3.05086700
H	-0.95122600	2.62357400	2.78523100
C	1.11377800	1.69649000	-1.19411800
C	0.69833600	1.38090400	0.17886700
H	1.43622600	0.81179100	-1.74541200
H	1.15979100	0.53867600	0.67470100
H	0.23406900	2.15385200	0.77113800
C	0.30308200	2.61226500	-2.06457600
C	-1.05494700	2.83457600	-1.82592300
C	0.90741800	3.19755100	-3.18444600
C	-1.80956600	3.61723900	-2.70298700
H	-1.52598000	2.35349900	-0.97267300
C	0.15644800	3.98064300	-4.05916200
H	1.96430300	3.03209100	-3.37088300
C	-1.20650800	4.18973100	-3.82378600
H	-2.86725000	3.77522700	-2.50929600
H	0.63321300	4.42744900	-4.92734300
H	-1.79113200	4.79766100	-4.50865700
N	2.17918600	2.35151700	-0.42180200
S	3.58955300	1.46280900	-0.40322200
O	3.35115000	0.01175300	-0.20470200
O	4.52657400	2.15186300	0.49558800
C	4.14618900	1.66557700	-2.09327600
C	3.96583000	0.63131000	-3.01041700
C	4.63235100	2.91007900	-2.50234400
C	4.26666400	0.85296900	-4.35474800
H	3.60080300	-0.33020200	-2.66839200
C	4.93186100	3.11317300	-3.84564100
H	4.76126600	3.70479800	-1.77566500
C	4.74860500	2.09223700	-4.79294400
H	4.12495900	0.05013700	-5.07363900
H	5.30881800	4.07987600	-4.16936400
C	-3.23108100	-1.76547100	-4.10029300
H	-2.73150000	-2.70592000	-4.33973100
H	-4.30470100	-1.90694900	-4.28062200
H	-2.88214900	-1.00334900	-4.80327000
C	-3.63869600	-2.37799300	-1.69323800
H	-3.46088400	-2.11515800	-0.64515500
H	-4.72369800	-2.42189600	-1.85419100
H	-3.23911600	-3.38084000	-1.86988700
C	-3.75104400	0.00781800	-2.42024600
H	-4.80989800	-0.10357700	-2.68846400
H	-3.68412300	0.31984600	-1.37326100
H	-3.33121600	0.81117100	-3.03461700
C	-2.58583100	-2.25757400	2.42893100
H	-2.13870400	-2.36229100	1.43663400
H	-2.46078300	-3.20494400	2.96931800
H	-3.65693600	-2.08604700	2.30096600
C	-0.40967600	-1.49656100	3.37327400
H	-0.33153700	-2.49142300	3.82893900
H	0.09668900	-1.52893900	2.40200600
H	0.12618400	-0.79430700	4.01870900
C	-2.53724000	-1.00573300	4.60931500
H	-2.35432800	-1.92936100	5.17350800
H	-2.11407500	-0.17945500	5.18939900
H	-3.62049200	-0.86809800	4.55171800
C	-4.73513800	0.36156900	1.91526000
H	-5.67224000	0.91606700	1.77730500
H	-4.51389000	-0.16510700	0.98082100
H	-4.90669100	-0.38237400	2.69844000
C	-3.90720600	1.98458100	3.65233500
H	-4.88902300	2.47380600	3.61222200
H	-3.93638500	1.25569700	4.46436000
H	-3.17192900	2.75286800	3.90930600
C	-3.57437000	2.45803900	1.22155300
H	-3.47265700	2.04088300	0.21609800
H	-4.50899200	3.03204400	1.26571200
H	-2.74820900	3.15966900	1.38081200
C	-0.41227800	-3.33780300	-0.78840200
H	0.24502200	-4.21544700	-0.73394000
H	-0.17863500	-2.67745600	0.05254900
H	-1.44207800	-3.68378600	-0.66901200
C	-0.46936100	-3.57058700	-3.29170600
H	0.25594300	-4.39434700	-3.26796000
H	-1.46689200	-4.01358200	-3.22492000
H	-0.37399400	-3.07885200	-4.26525400
C	1.29310900	-2.17869000	-2.18054600
H	1.54382100	-1.45772500	-1.39593200
H	1.92895200	-3.05990800	-2.02852900
H	1.55985300	-1.74665300	-3.15027200
C	5.09071200	2.32364300	-6.24372200
H	6.17444400	2.25925600	-6.40538100
H	4.61584800	1.57946100	-6.89021900
H	4.77359800	3.31849600	-6.57462400

TS12

Pd	-1.27958100	0.00122100	0.00314900
P	-1.93305200	0.46705100	2.17561500
P	-1.18857900	-1.02135100	-2.13511300
C	-3.63230100	1.32159600	2.26858100
C	-0.87453300	1.62838600	3.15998300
C	-1.89157400	-1.11668600	3.22583600
C	-2.99688800	-1.36676700	-2.63366700
C	-0.15142700	-2.61735900	-2.10837800
C	-0.56515300	-0.17480600	-3.66330200
H	0.45570900	0.17771200	-3.51876000

H -1.18438300 0.70186100 -3.86480500
H -0.58599900 -0.83795800 -4.53270200
H -1.12663200 1.63301800 4.22386400
H 0.17634200 1.35366300 3.04768200
H -0.99566700 2.64400000 2.77200800
C 1.10290300 1.71055200 -1.19531700
C 0.69038300 1.39763100 0.17911800
H 1.43553900 0.82623100 -1.74107400
H 1.16073700 0.56287000 0.67929800
H 0.21788200 2.16844200 0.76762500
C 0.28264900 2.61186700 -2.07178500
C -1.07682100 2.82427600 -1.83259500
C 0.87988400 3.19304100 -3.19762800
C -1.83995100 3.59249300 -2.71505800
H -1.54195800 2.34672700 -0.97423900
C 0.12045100 3.96174400 -4.07774800
H 1.93785800 3.03541900 -3.38463500
C -1.24397000 4.16053200 -3.84187800
H -2.89866600 3.74268200 -2.52082700
H 0.59172500 4.40525900 -4.95060400
H -1.83519200 4.75714300 -4.53102900
N 2.16054300 2.38168700 -0.42617000
S 3.58135800 1.50990800 -0.40397500
O 3.36039300 0.05706800 -0.19856800
O 4.51056500 2.21432900 0.49102800
C 4.13433600 1.71156000 -2.09536200
C 3.96338000 0.67180400 -3.00810500
C 4.60664300 2.95947900 -2.51021100
C 4.25970300 0.89119800 -4.35381000
H 3.60893300 -0.29209900 -2.66166600
C 4.90187700 3.16030000 -3.85479000
H 4.72820500 3.75853700 -1.78703000
C 4.72791600 2.13369900 -4.79771900
H 4.12531700 0.08403900 -5.06925500
H 5.26804300 4.12963500 -4.18299000
C -3.19950400 -1.80910400 -4.09217300
H -2.69338300 -2.74799500 -4.32396600
H -4.27182100 -1.95882900 -4.27356900
H -2.85413200 -1.04978100 -4.79993100
C -3.60669900 -2.40784800 -1.68145500
H -3.43205700 -2.13623900 -0.63502700
H -4.69113600 -2.46076900 -1.84349400
H -3.19961800 -3.40902300 -1.85048000
C -3.73581300 -0.02818900 -2.42516100
H -4.79368000 -0.14958700 -2.69300000
H -3.67165700 0.29173200 -1.38039000
H -3.32186800 0.77406000 -3.04489900
C -2.55988000 -2.26305000 2.44490500
H -2.10435000 -2.37602200 1.45742500
H -2.42968400 -3.20248600 2.99781700
H -3.63163400 -2.10388500 2.30670600
C -0.39659800 -1.46456400 3.38846000
H -0.30602500 -2.45556300 3.85020800
H 0.11339100 -1.49533800 2.41900100
H 0.12717400 -0.75103300 4.03150700
C -2.53450100 -0.99255900 4.61428100
H -2.33944200 -1.90828000 5.18721300
H -2.12566500 -0.15483600 5.18814600
H -3.61956000 -0.87187700 4.55223200
C -4.73996900 0.32299900 1.89545700
H -5.68348100 0.86340800 1.74584300
H -4.50358300 -0.20741900 0.96681700
H -4.90763100 -0.41748800 2.68277500
C -3.94586900 1.96637300 3.62950300
H -4.93332600 2.44324500 3.58016900
H -3.97194800 1.24098300 4.44468400
H -3.22188300 2.74480800 3.88795800
C -3.60558200 2.43295600 1.19888400
H -3.49270700 2.01328200 0.19573700
H -4.54850700 2.99374600 1.23561100
H -2.79042000 3.14707600 1.35932100
C -0.37452300 -3.33838200 -0.76701200
H 0.28958800 -4.21050600 -0.70677600
H -0.14829900 -2.67237600 0.07147800
H -1.40183700 -3.69200600 -0.64869000
C -0.42253800 -3.58455500 -3.26889600
H 0.30970300 -4.40190300 -3.23862100
H -1.41651100 -4.03544900 -3.20202700
H -0.32889900 -3.09736500 -4.24488200
C 1.32482000 -2.17130100 -2.16004100
H 1.56656300 -1.44284600 -1.37944200
H 1.96788600 -3.04585400 -2.00019400
H 1.59092500 -1.74327100 -3.13172000
C 5.06524900 2.36294200 -6.24995500
H 4.73595000 3.35257600 -6.58459700
H 6.14943900 2.31067700 -6.41288700
H 4.59829600 1.61047500 -6.89260600

TS13
Pd 1.44532100 1.94064800 0.36433800
P 0.13059800 3.33365400 1.71692900
P 3.25419500 1.06873800 -0.87754900
C 0.49548800 3.14371400 3.57534000
C -1.71238400 3.21299100 1.68154700
C 0.43353200 5.11594100 1.10967300
C 3.20720400 1.62223300 -2.69549200
C 3.34816800 -0.77089000 -1.01674400
C 4.91329100 1.53314800 -0.06620300
H 2.51443600 -1.12593200 -1.62889800
H 4.28791200 -1.11236300 -1.46111000
H 3.24079300 -1.20434200 -0.01986200
O 2.19421800 -0.76397300 2.25482000
H 1.44897700 -1.35063900 2.04450700
H 2.01617100 0.05935700 1.75086800
H -2.02567700 2.26498700 2.12514900
H -2.19219500 4.03024000 2.22773300
H -2.05920800 3.22002600 0.65026600
C -1.02494500 0.22415300 -1.18055500
C -0.14151800 0.14188100 0.00925300
H -0.47549300 0.20332500 -2.11916700
H 0.74973700 -0.45693800 -0.08438600
H -0.58365400 0.27615900 0.98585100
C -2.14094000 1.22884200 -1.20580300
C -1.97425100 2.42567800 -1.91103800
C -3.34226500 0.99531800 -0.52976100
C -2.98488600 3.38753700 -1.92244300
H -1.04975800 2.60431700 -2.45177400
C -4.35504900 1.95506400 -0.54251000
H -3.47403900 0.05265600 -0.01013300
C -4.17763000 3.15637900 -1.23293100

H	-2.84143400	4.31395300	-2.47127000
H	-5.28557800	1.76327700	-0.01555300
H	-4.96652100	3.90308400	-1.24233300
N	-1.40831500	-1.09299900	-0.66843800
S	-0.63654700	-2.37791600	-1.38566300
O	-1.66401900	-3.32884100	-1.84271900
O	0.39530400	-1.95406600	-2.36699900
C	0.25522800	-3.11111700	-0.01284900
C	-0.30336400	-3.12775400	1.26862100
C	1.51587700	-3.65625800	-0.24650500
C	0.42855800	-3.67133300	2.32212700
H	-1.28584100	-2.70086600	1.43566400
C	2.23376800	-4.20033200	0.81823800
H	1.93517100	-3.63147700	-1.24557400
C	1.71174700	-4.20498600	2.11659000
H	0.00241300	-3.67571900	3.32186500
H	3.22267300	-4.61319200	0.63873400
C	2.51855300	-4.73106600	3.27599300
H	3.28173600	-5.44065400	2.94242500
H	1.88146600	-5.23064200	4.01312300
H	3.03238200	-3.90859500	3.78981300
C	-0.27650900	5.22063700	-0.25538100
H	0.00384100	6.16902900	-0.73099400
H	0.02303100	4.40448100	-0.92117900
H	-1.36543300	5.20158000	-0.16366000
C	-0.09223300	6.22143900	2.03560000
H	0.50474700	6.30565900	2.94798100
H	-0.02968800	7.18649300	1.51608600
H	-1.13853800	6.06870400	2.31801200
C	1.93839300	5.32353200	0.86531400
H	2.52867300	5.24278400	1.78112800
H	2.31904200	4.58512300	0.15371300
H	2.10394100	6.32526700	0.44738300
C	-0.41733400	3.96815800	4.49926700
H	-0.16766200	3.73556100	5.54240900
H	-0.29674100	5.04380900	4.36563400
H	-1.47366100	3.72145300	4.35674300
C	1.96756500	3.48868100	3.84914700
H	2.16114300	4.56092700	3.74936400
H	2.22737600	3.20084900	4.87573500
H	2.63470200	2.94869300	3.16829000
C	0.27522200	1.65136100	3.90104800
H	0.95720900	1.00095800	3.35016400
H	0.45446500	1.49116100	4.97203700
H	-0.75202900	1.33043700	3.69443000
C	4.86073200	3.00419100	0.38256300
H	4.01674400	3.17165400	1.05771000
H	5.78626200	3.25477200	0.91724500
H	4.76199500	3.69730700	-0.45584400
C	5.04995000	0.66199400	1.19716300
H	5.93399800	0.98942500	1.75902900
H	4.18108700	0.75440300	1.85385600
H	5.18085100	-0.39676200	0.95830800
C	6.14300000	1.30246200	-0.95663700
H	6.17287800	1.98743700	-1.80797100
H	7.05184900	1.47598200	-0.36596000
H	6.19136400	0.27687800	-1.33653100
C	1.75070900	1.40891400	-3.14867300
H	1.06753500	2.00496900	-2.53648200
H	1.64409700	1.72215400	-4.19546700
H	1.44796700	0.35961500	-3.08185800
C	3.52677400	3.12237800	-2.79917000
H	3.30784300	3.47034400	-3.81669700
H	2.91456700	3.70804200	-2.10437500
H	4.57941300	3.33827100	-2.59748200
C	4.12328200	0.81514000	-3.63039800
H	5.17908500	0.90008600	-3.36880300
H	3.85515200	-0.24530600	-3.63862500
H	4.00434600	1.18945000	-4.65535700

TS14

Pd	-0.92544100	-0.37770000	0.04484000
P	-0.32914300	-0.89582800	2.23168600
P	-2.09426300	-0.39446700	-2.00995000
C	-1.36914200	0.04631800	3.51513800
C	1.39955300	-0.48942400	2.76307700
C	-0.40484600	-2.77516500	2.50910000
C	-3.95499000	-0.33301600	-1.60239600
C	-1.65155000	-1.95501000	-3.00840000
C	-1.96354900	0.92520000	-3.30444300
H	-0.91646300	1.12362900	-3.53287200
H	-2.39297300	1.85367000	-2.92420100
H	-2.47920600	0.64207400	-4.22673700
H	1.66109000	-0.93234600	3.72813300
H	2.10656900	-0.84120000	2.00893300
H	1.51112900	0.59532100	2.84040500
C	1.00258000	1.79247300	-1.41384500
C	1.02423700	1.04693700	-0.14591000
H	1.05955900	1.15265300	-2.29862000
H	1.55852200	0.11191600	-0.10038700
H	0.82746100	1.57600600	0.77388200
C	0.03521400	2.92230500	-1.62005800
C	0.24832100	3.79755500	-2.69157800
C	-1.07688500	3.10387500	-0.79667000
C	-0.65447400	4.82727900	-2.94812700
H	1.12664000	3.66141500	-3.31711600
C	-1.98271200	4.13708200	-1.05079900
H	-1.25254800	2.41073500	0.01895900
C	-1.77681300	4.99706400	-2.13006300
H	-0.48457000	5.49907900	-3.78481500
H	-2.84880600	4.26508500	-0.40699000
H	-2.48113900	5.79937600	-2.33128800
N	2.29876600	2.20655500	-0.86981100
S	3.62508300	1.56890300	-1.65305400
O	4.80834100	2.14337200	-0.99578300
O	3.45952700	1.71746300	-3.11632900
C	3.64619900	-0.19606100	-1.32514000
C	4.14350400	-0.66347700	-0.10673600
C	3.07332800	-1.07607000	-2.24340700
C	4.05424800	-2.02190900	0.18983900
H	4.59389600	0.03305500	0.59232500
C	2.98356400	-2.43125900	-1.92875900
H	2.71465000	-0.69693600	-3.19340700
C	3.46861400	-2.92472700	-0.71046400
H	4.44602500	-2.38997300	1.13443500
H	2.53074200	-3.11559600	-2.64133600
C	-4.87472400	-0.10961200	-2.81463600
H	-4.82169500	-0.92112900	-3.54287800
H	-5.91422000	-0.04746600	-2.46692700
H	-4.64930300	0.82818500	-3.33073000

C	-4.37009400	-1.61308100	-0.85992700
H	-3.70671500	-1.81236500	-0.01174900
H	-5.39047400	-1.49520700	-0.47277500
H	-4.36679100	-2.48924900	-1.51508600
C	-4.12217400	0.86223900	-0.64041400
H	-5.17871800	0.95492600	-0.35665100
H	-3.52593900	0.72125400	0.26721100
H	-3.81368800	1.80645200	-1.10010300
C	-1.70878200	-3.32833200	1.90683200
H	-1.79297600	-3.05946000	0.85105200
H	-1.70779400	-4.42339700	1.98600900
H	-2.59715700	-2.95811600	2.42314000
C	0.77363500	-3.35753500	1.70293500
H	0.67893000	-4.44969000	1.66116600
H	0.78396300	-2.97747500	0.67552500
H	1.74005300	-3.12581400	2.15947000
C	-0.28400700	-3.21972000	3.97357700
H	-0.19884000	-4.31343000	4.01456400
H	0.60240800	-2.80440900	4.46372700
H	-1.16422800	-2.93969700	4.55874400
C	-2.80956100	-0.49020500	3.51203600
H	-3.45256500	0.18608200	4.08951200
H	-3.21148200	-0.55227400	2.49464100
H	-2.87808900	-1.48002500	3.97203300
C	-0.79956200	0.01749900	4.94313000
H	-1.45301700	0.60353800	5.60207800
H	-0.74034000	-0.99296900	5.35158600
H	0.19743900	0.46498300	4.99239100
C	-1.39632600	1.51012200	3.02828500
H	-1.85778800	1.58759100	2.03954400
H	-1.97763700	2.11823400	3.73309000
H	-0.39276300	1.94621300	2.96926300
C	-1.46279600	-3.13894600	-2.04438800
H	-1.13076400	-4.02060000	-2.60873700
H	-0.70593000	-2.90317800	-1.28978400
H	-2.38598600	-3.40431400	-1.52384200
C	-2.65691300	-2.33337800	-4.10359900
H	-2.24535400	-3.15264000	-4.70748800
H	-3.60470500	-2.68149700	-3.68396500
H	-2.86553900	-1.50000300	-4.78169900
C	-0.28314600	-1.65681500	-3.65303000
H	0.43329600	-1.30072800	-2.90664200
H	0.12033100	-2.57906300	-4.09060000
H	-0.35119000	-0.91439100	-4.45346600
C	3.38897800	-4.39707000	-0.39314400
H	4.16103900	-4.95661400	-0.93633100
H	3.53362700	-4.58424700	0.67471800
H	2.41899200	-4.81392700	-0.68421400

TS15

Pd	0.63303000	-2.02028600	-0.54188200
P	2.84996500	-1.78849800	0.16425600
P	-1.45861900	-2.90134300	-1.07540600
C	3.22149400	-3.12233800	1.47204400
C	3.36262900	-0.24890000	1.05038500
C	4.07294900	-1.86845200	-1.28956900
C	-2.41130100	-3.46797700	0.47263300
C	-1.26137600	-4.32459200	-2.32345900
C	-2.69708500	-1.80170700	-1.90203300
H	-2.23108500	-1.30352900	-2.75184600
H	-3.01287000	-1.02475900	-1.20039800
H	-3.58394600	-2.34517500	-2.24096100
H	4.44754700	-0.18089900	1.17640200
H	3.00872300	0.63969000	0.52433100
H	2.89776500	-0.24422900	2.03968600
C	0.26192900	1.11347300	-1.11681300
C	-0.11615200	0.22190900	-0.00157600
H	1.33862100	1.26346100	-1.20844500
H	0.51326500	0.17106600	0.87421300
H	-1.14544400	-0.08258100	0.09326700
C	-0.41103100	0.93068300	-2.44851300
C	0.12803300	0.02421300	-3.36982200
C	-1.57809100	1.62502900	-2.77404000
C	-0.49571600	-0.18994700	-4.59824700
H	1.02516800	-0.52997000	-3.11029900
C	-2.20408500	1.41044700	-4.00422600
H	-1.98169100	2.33105100	-2.05666500
C	-1.66752800	0.50184800	-4.91832400
H	-0.07092300	-0.89945800	-5.30262600
H	-3.11122800	1.95625300	-4.24867800
H	-2.15482300	0.33596900	-5.87505700
N	-0.41120400	2.03743300	-0.20255600
S	0.58574400	2.97234900	0.75172000
O	0.04964500	4.34289600	0.72482000
O	2.02208200	2.77369800	0.44084900
C	0.31442100	2.29827400	2.39057000
C	-0.99053300	2.11825000	2.85825300
C	1.40787000	1.92165200	3.16532900
C	-1.19026100	1.53456700	4.10438500
H	-1.83339900	2.41287500	2.24250600
C	1.19055400	1.33954200	4.41614100
H	2.41042500	2.07377100	2.78370700
C	-0.10511700	1.13192200	4.90145400
H	-2.20364600	1.38391500	4.46767800
H	2.04256800	1.04129000	5.02150000
C	-3.90873200	-3.73072300	0.24043500
H	-4.09059900	-4.52553200	-0.48554600
H	-4.37098200	-4.03634800	1.18804900
H	-4.43051900	-2.83188900	-0.10120900
C	-1.74746600	-4.71974700	1.06902200
H	-0.66636400	-4.58395000	1.17777300
H	-2.16667200	-4.91667200	2.06402200
H	-1.92367600	-5.60865700	0.45669300
C	-2.27212400	-2.31848700	1.49262200
H	-2.79080100	-2.59103700	2.42084500
H	-1.22148000	-2.11998200	1.72538500
H	-2.72285200	-1.38854100	1.12835800
C	3.57717600	-2.92544700	-2.29306900
H	2.54927700	-2.71265900	-2.60583700
H	4.22188600	-2.91982700	-3.18178000
H	3.59401100	-3.93457200	-1.87347100
C	4.00645000	-0.48675800	-1.97056300
H	4.56175800	-0.52129200	-2.91615600
H	2.97550800	-0.20037600	-2.20022500
H	4.44786300	0.30000900	-1.35233700
C	5.52870800	-2.16166400	-0.90102300
H	6.16895400	-2.06331300	-1.78732900
H	5.90400500	-1.46226100	-0.14671200
H	5.65141900	-3.17899200	-0.51957300
C	3.19037700	-4.51068200	0.81327900

H 3.22541000 -5.28636300 1.58926800
H 2.27147100 -4.65142400 0.23381800
H 4.04558600 -4.67068100 0.14993700
C 4.54065600 -2.92799300 2.23715400
H 4.62432600 -3.70274100 3.01050500
H 5.41815200 -3.01091200 1.59370400
H 4.57881500 -1.95826100 2.74214300
C 2.05599600 -3.03986900 2.48052900
H 1.09593200 -3.20203500 1.98099100
H 2.18824700 -3.80844100 3.25334100
H 2.01113800 -2.06781100 2.98417900
C -0.08563500 -5.21943600 -1.88839900
H 0.10231000 -5.97437700 -2.66317600
H 0.82381600 -4.62444600 -1.75575700
H -0.28319300 -5.74488500 -0.95146300
C -2.51762500 -5.18021000 -2.53404800
H -2.34526400 -5.88235200 -3.36026800
H -2.76205300 -5.77324700 -1.64842200
H -3.39132200 -4.57515200 -2.79611900
C -0.87140200 -3.65871000 -3.65843700
H -0.01037500 -2.99480000 -3.53184300
H -0.59938600 -4.43725100 -4.38249700
H -1.68950900 -3.07352500 -4.08742400
C -0.34069500 0.50822100 6.25477200
H 0.57361100 0.05661700 6.65105800
H -0.68270400 1.25831400 6.97911600
H -1.11235300 -0.26825200 6.20624800

TS16
Pd 1.04430700 2.14056200 -0.02842500
P 0.32671600 2.86483600 2.06931300
P 2.15252900 2.11882700 -2.12679700
C 1.39751700 2.24372300 3.51324300
C -1.39042400 2.40725600 2.60116100
C 0.21459200 4.76672800 2.03956400
C 1.09660800 2.76514000 -3.57419700
C 2.82392200 0.52247100 -2.78039100
C 3.73655900 3.16882500 -1.93951800
H 2.06192100 -0.25653300 -2.71953800
H 3.17575600 0.60924900 -3.81252600
H 3.65485700 0.20593900 -2.14622600
O 3.28044800 -0.40460800 0.42500600
H 2.79101600 -1.02799100 -0.15035400
H 2.80356500 0.44205300 0.31074700
H -1.52426600 1.32418300 2.58008400
H -1.62506900 2.76162300 3.60870100
H -2.10722300 2.83854400 1.89724600
C -0.74614400 -0.37001600 -0.98801600
C -0.07200500 0.04007600 0.25878200
H -0.07385400 -0.45519500 -1.84390200
H 0.92221500 -0.32084300 0.45438500
H -0.70335300 0.28472400 1.09620000
C -2.04539000 0.29989000 -1.34464700
C -2.24131100 1.66244300 -1.09639700
C -3.05843400 -0.43722600 -1.96602500
C -3.43093500 2.28638600 -1.47549900
H -1.44550300 2.22955600 -0.61655000
C -4.24760900 0.18521100 -2.34476600
H -2.91013900 -1.49949500 -2.13709700
C -4.43691700 1.54900400 -2.10215000
H -3.57040500 3.34642300 -1.27950700
H -5.03017900 -0.39465500 -2.82605900
H -5.36507300 2.03113700 -2.39602300
N -0.91872300 -1.60081900 -0.21237300
S 0.22231900 -2.76843300 -0.51993100
O -0.36511800 -3.81183300 -1.38052800
O 1.52294700 -2.19190900 -0.97501600
C 0.46668600 -3.45220700 1.11120200
C -0.37017400 -4.48119600 1.54082600
C 1.44584700 -2.91946600 1.95260000
C -0.22703800 -4.97580000 2.83576800
H -1.11774600 -4.88500700 0.86728700
C 1.57216300 -3.42655400 3.24341500
H 2.10300800 -2.12704500 1.61165900
C 0.74189700 -4.45790700 3.70539000
H -0.87814600 -5.77646600 3.17647200
H 2.33224300 -3.01491200 3.90246900
C 0.91106100 -5.01632400 5.09654100
H 1.68199200 -5.79772900 5.11295900
H -0.01755400 -5.46451700 5.46348000
H 1.22110300 -4.23965000 5.80332600
C 1.62591300 5.37722400 2.04134500
H 2.26421600 4.92048700 1.27799200
H 1.55685600 6.45060800 1.82355100
H 2.11987300 5.27087300 3.01143100
C -0.61786900 5.37741600 3.18003000
H -0.20892000 5.15944700 4.16824700
H -0.63363600 6.46856500 3.06248800
H -1.65606800 5.03373200 3.15606200
C -0.46454200 5.12094200 0.69997100
H 0.12453800 4.76110400 -0.14812700
H -1.46807400 4.68894100 0.61723600
H -0.56590600 6.21115600 0.62179300
C 1.05849100 0.75021100 3.70407100
H 1.18806600 0.18023500 2.78020700
H 1.74257000 0.32442000 4.44835000
H 0.03872800 0.59699900 4.06890000
C 2.88283300 2.33815900 3.12037600
H 3.49797500 1.94739500 3.94103000
H 3.08917000 1.73908900 2.23024900
H 3.20230300 3.36392300 2.92287900
C 1.15979500 2.98252600 4.83803400
H 1.72296100 2.48271000 5.63650700
H 1.50434500 4.01898700 4.79519900
H 0.10464700 2.98059600 5.13004900
C 4.77079600 2.95395000 -3.05827900
H 4.39515000 3.22913200 -4.04473900
H 5.65091300 3.57706500 -2.85369000
H 5.11045600 1.91495200 -3.09893200
C 3.37143100 4.65884700 -1.83589600
H 3.02250500 5.07128300 -2.78616700
H 2.59508800 4.82682800 -1.08172400
H 4.25973900 5.22921300 -1.53564700
C 4.38124000 2.74479200 -0.60438900
H 3.71818100 2.96474100 0.23779100
H 4.61914600 1.67735000 -0.57461100
H 5.31729200 3.30121900 -0.46374700
C 0.14782000 1.62026000 -3.97558700
H 0.68451300 0.76197100 -4.39018800
H -0.45614200 1.28531400 -3.13131400

H	-0.54392600	1.98327200	-4.74595600
C	0.23468500	3.94143100	-3.08088200
H	-0.42588200	4.27322600	-3.89232600
H	-0.38969700	3.63782900	-2.23652500
H	0.83589300	4.79789800	-2.76729200
C	1.89880200	3.18865200	-4.81322700
H	2.51328700	4.07287700	-4.62816500
H	2.54797900	2.38640300	-5.17799500
H	1.20061200	3.43742100	-5.62273500

TS17

Pd	0.46476100	-1.96995800	-0.76317300
P	2.59213700	-1.65569600	0.18581800
P	-1.56908600	-2.69957500	-1.65737900
C	2.63184000	-1.99268600	2.05876500
C	3.32857100	0.04194400	0.09381700
C	3.88669100	-2.70755500	-0.73184200
C	-2.75776100	-3.36724000	-0.32772300
C	-1.31983800	-3.95497500	-3.06396200
C	-2.60695200	-1.40296900	-2.48173300
H	-1.97850000	-0.78574900	-3.12488900
H	-3.05088900	-0.74831200	-1.72695500
H	-3.41823000	-1.83579300	-3.07375300
H	4.38147900	0.05830500	0.39007700
H	3.24760200	0.41582300	-0.92939300
H	2.76811400	0.72050700	0.74210500
C	0.17117100	1.26926100	-0.94828200
C	-0.47228300	0.20464700	-0.15285900
H	1.21391700	1.44055100	-0.68942200
H	-0.14084900	0.02499100	0.85706000
H	-1.46511500	-0.09948400	-0.42881300
C	-0.06788800	1.25190600	-2.43058700
C	0.73728100	0.42928100	-3.22896700
C	-1.10509100	1.97805900	-3.01740600
C	0.49601100	0.32220500	-4.59763000
H	1.53227900	-0.14705200	-2.76410300
C	-1.34400500	1.87470000	-4.39038000
H	-1.71887300	2.61272500	-2.38761600
C	-0.54992400	1.04295100	-5.18215400
H	1.11954500	-0.32602200	-5.20708300
H	-2.15311600	2.44346400	-4.84033200
H	-0.73945200	0.95999000	-6.24870100
N	-0.79600900	2.03828400	-0.16663900
S	-0.21836800	2.71541600	1.23646000
O	-0.48470000	4.16360200	1.19274800
O	1.16362500	2.27840100	1.56212100
C	-1.29320500	1.96426200	2.45756900
C	-2.66707600	1.87228700	2.21718400
C	-0.73682000	1.44582000	3.62366700
C	-3.47754400	1.23490900	3.15053500
H	-3.08485200	2.27092000	1.29956000
C	-1.56500000	0.81327800	4.55230600
H	0.33126000	1.52211600	3.78907700
C	-2.94101700	0.69357100	4.33047300
H	-4.54485900	1.15067700	2.96199500
H	-1.13135700	0.40035200	5.45924500
C	-4.22074700	-3.49419200	-0.78451000
H	-4.34637900	-4.20257300	-1.60534400
H	-4.82948400	-3.85093800	0.05649700
H	-4.63343200	-2.53114600	-1.09984100
C	-2.24740900	-4.72208000	0.18821700
H	-1.18269100	-4.67358100	0.44268400
H	-2.80138700	-5.00131900	1.09354500
H	-2.39313600	-5.52079500	-0.54451600
C	-2.70026200	-2.35946200	0.84011700
H	-3.35193200	-2.70917000	1.65099000
H	-1.68257100	-2.26337800	1.23030500
H	-3.04923100	-1.36224300	0.55124200
C	3.34730300	-4.13623700	-0.91487600
H	2.36548700	-4.11652700	-1.39530600
H	4.03415700	-4.70778500	-1.55265300
H	3.24608600	-4.67140000	0.03130700
C	4.01867400	-2.07438600	-2.13278500
H	4.63221200	-2.72616200	-2.76730500
H	3.03808400	-1.96437600	-2.61047800
H	4.50190100	-1.09345500	-2.10124000
C	5.26978300	-2.75937700	-0.06784800
H	5.97603700	-3.26620900	-0.73841200
H	5.67111100	-1.76206700	0.13762600
H	5.24964200	-3.32212100	0.86964000
C	2.48046400	-3.49752300	2.32986600
H	2.29503000	-3.65619700	3.39999400
H	1.63652300	-3.92113700	1.77388000
H	3.38385400	-4.05521000	2.06764400
C	3.88242600	-1.45149400	2.77241400
H	3.80830200	-1.67909700	3.84377600
H	4.80757800	-1.89924500	2.40456600
H	3.96542100	-0.36514400	2.67610200
C	1.39838700	-1.26883500	2.63409200
H	0.47321600	-1.67876100	2.21718900
H	1.37262100	-1.40220100	3.72350600
H	1.42185100	-0.19372200	2.43319800
C	-0.30958500	-5.02695300	-2.62265600
H	-0.07562400	-5.67912400	-3.47419800
H	0.61890900	-4.56454700	-2.27887300
H	-0.69075100	-5.65580300	-1.81548700
C	-2.60239300	-4.63383800	-3.56512000
H	-2.37234200	-5.22247200	-4.46267600
H	-3.01746300	-5.32162800	-2.82301800
H	-3.37817700	-3.91179300	-3.83887200
C	-0.68263400	-3.15406600	-4.21892600
H	0.20841600	-2.61420200	-3.88224100
H	-0.38330000	-3.84813200	-5.01464100
H	-1.37353000	-2.42599200	-4.65299500
C	-3.83645700	0.01674600	5.33843600
H	-4.56258800	-0.64080300	4.84791200
H	-3.25784000	-0.58180100	6.04839300
H	-4.40744100	0.75565200	5.91520100

TS18

Pd	0.49223200	-1.91735800	-0.77883000
P	2.36278300	-1.83678100	0.61891100
P	-1.33165100	-2.62795800	-2.04573100
C	2.20815700	-3.20291900	1.93700200
C	2.65066200	-0.34607600	1.67410200
C	3.98397600	-1.97671500	-0.36235400
C	-2.70175500	-3.37898200	-0.95227400
C	-0.75584900	-3.85582500	-3.37766500
C	-2.27068500	-1.36522800	-3.02036400
H	-1.57143400	-0.75313700	-3.59031000

H -2.80080000 -0.70144100 -2.33202300
H -3.00252200 -1.81128300 -3.70053100
H 3.64233700 -0.34543300 2.13659200
H 2.52763900 0.57219300 1.09814400
H 1.89947600 -0.32831300 2.46638200
C 0.24452800 1.30301300 -0.93811600
C -0.42123000 0.24986500 -0.14728100
H 1.29106800 1.45724500 -0.67110100
H -0.08200000 0.04875200 0.85687800
H -1.41752900 -0.05580300 -0.41900500
C 0.01916400 1.34379400 -2.42337600
C 0.83708500 0.57169100 -3.25739800
C -1.00563900 2.10813600 -2.98404100
C 0.62858500 0.55956100 -4.63584300
H 1.61987800 -0.03856500 -2.81665200
C -1.21577800 2.09607000 -4.36510000
H -1.62751000 2.70664200 -2.32741300
C -0.40250400 1.32110000 -5.19407500
H 1.26539300 -0.04743000 -5.27313600
H -2.01512500 2.69473000 -4.79307800
H -0.56669900 1.31225800 -6.26797800
N -0.71278000 2.07154000 -0.13996900
S -0.09549700 2.84612000 1.20234300
O -0.78188900 4.14463300 1.29334700
O 1.38599100 2.82534800 1.23715200
C -0.64850200 1.82291000 2.57099000
C -1.96804100 1.36501000 2.61513900
C 0.26500700 1.45484800 3.55552100
C -2.35474600 0.50247300 3.63579100
H -2.67116100 1.65996700 1.84383500
C -0.13988000 0.59443100 4.57772000
H 1.28142100 1.82772600 3.50685100
C -1.44576900 0.09258400 4.62488600
H -3.37742400 0.13569300 3.66638500
H 0.57327900 0.30415900 5.34496500
C -4.05113000 -3.57710300 -1.66270300
H -3.98751300 -4.27290200 -2.50127800
H -4.77624500 -3.98640400 -0.94719500
H -4.45832600 -2.63172500 -2.03320900
C -2.22414300 -4.71294600 -0.35649600
H -1.23089000 -4.61731700 0.09496800
H -2.92296100 -5.03413100 0.42672900
H -2.18744300 -5.50719200 -1.10749200
C -2.90696300 -2.37959000 0.20547400
H -3.67164200 -2.76884700 0.89015900
H -1.98184200 -2.22551300 0.76858800
H -3.25679700 -1.40427100 -0.15096900
C 3.78375200 -2.98780600 -1.50597100
H 2.93754000 -2.69639500 -2.13748700
H 4.68772100 -3.02259700 -2.12823500
H 3.59189000 -3.99905700 -1.13732900
C 4.21897600 -0.58570400 -0.98583500
H 5.03662900 -0.64927700 -1.71460600
H 3.32941900 -0.22401600 -1.51174400
H 4.49848600 0.16076300 -0.23679100
C 5.21555300 -2.36358900 0.46758100
H 6.11437400 -2.29129600 -0.15843700
H 5.35857500 -1.69840000 1.32529200
H 5.15386200 -3.39178900 0.83445000
C 2.31042700 -4.57957800 1.26095500
H 2.03565700 -5.36336300 1.97872100
H 1.63121400 -4.64886000 0.40393500
H 3.32603600 -4.79330000 0.91447100
C 3.21522200 -3.10253100 3.09385400
H 3.00252000 -3.89397500 3.82446300
H 4.24875900 -3.22748100 2.76671100
H 3.13635400 -2.14597200 3.61900000
C 0.78465300 -3.05076500 2.51581200
H 0.03142900 -3.14942500 1.72808000
H 0.61334200 -3.83159100 3.26850000
H 0.63513100 -2.08147400 3.00472100
C 0.23215100 -4.85468400 -2.74676100
H 0.67521000 -5.47656800 -3.53569500
H 1.03720900 -4.32692400 -2.22565000
H -0.25101200 -5.52093000 -2.02879600
C -1.88195100 -4.61625000 -4.09011800
H -1.46048300 -5.19658700 -4.92130800
H -2.38398700 -5.32078500 -3.42166700
H -2.63471400 -3.94182600 -4.51068900
C 0.01978700 -3.01250300 -4.40981700
H 0.79853200 -2.41383300 -3.92657200
H 0.50103400 -3.68320100 -5.13285700
H -0.63263500 -2.33299000 -4.96494200
C -1.86148600 -0.88665500 5.69424000
H -1.75737100 -1.91994300 5.33779900
H -1.24271800 -0.78531900 6.59117900
H -2.90852900 -0.74535200 5.98122200

TS19

Pd -0.60104500 1.75753900 -0.81638200
P -2.05217200 2.72205900 0.74971700
P 1.15383800 1.56813700 -2.34725600
C -1.03593300 3.44426200 2.19072000
C -3.31127600 1.69037900 1.63461300
C -3.14370800 4.04509300 -0.07567600
C 1.43769800 3.24677300 -3.08727800
C 0.85479300 0.50864600 -3.90133100
C 2.84127300 1.17154700 -1.55456600
H 2.36367700 3.31388700 -3.66706500
H 1.45803800 3.99131000 -2.28893900
H 0.59347200 3.48386300 -3.74056100
H -2.83582100 0.81163100 2.07506100
H -3.82714900 2.24861100 2.42072500
H -4.05644500 1.34444300 0.91205400
C -2.02767600 -1.12658900 -0.14359100
C -0.68622300 -0.51215700 -0.04656000
H -2.75742700 -0.70430600 0.54388300
H -0.35705500 -0.13102900 0.90834800
H 0.05616200 -0.80286100 -0.76725000
C -2.59084500 -1.34132400 -1.51853500
C -3.48936600 -0.40100600 -2.03432500
C -2.19791500 -2.42025000 -2.31442000
C -3.98289200 -0.52896500 -3.33179900
H -3.78815800 0.44046200 -1.41839500
C -2.69120400 -2.54966600 -3.61449200
H -1.51533500 -3.15515400 -1.90142200
C -3.58192200 -1.60508200 -4.12772400
H -4.67882200 0.20958800 -3.72009500
H -2.38186100 -3.39349800 -4.22515100
H -3.96562800 -1.70855100 -5.13877500

N	-1.24329700	-2.24169800	0.38962000
S	-1.22267300	-2.39355200	2.04620700
O	-1.58909000	-3.77964200	2.38119600
O	-1.94507600	-1.29287500	2.73174400
C	0.52137500	-2.16284700	2.39081700
C	1.46544000	-2.94944200	1.72687900
C	0.91943800	-1.14598400	3.25497000
C	2.81871600	-2.69059900	1.91868300
H	1.14138400	-3.73793600	1.05655500
C	2.28111900	-0.90070800	3.43823400
H	0.16874000	-0.55224000	3.76268500
C	3.24814000	-1.65594400	2.76514600
H	3.55730700	-3.29497900	1.39846400
H	2.59409400	-0.10422800	4.10838700
C	4.71668700	-1.34537000	2.91375700
H	5.32541600	-2.25414500	2.86541100
H	5.05736400	-0.68211000	2.10725600
H	4.92601900	-0.84131500	3.86230100
C	1.73660200	0.88719600	-5.10343700
H	2.80083800	0.74971900	-4.90449400
H	1.47242000	0.24673700	-5.95480800
H	1.57827000	1.92366100	-5.41541600
C	1.03491600	-0.98416800	-3.58132700
H	0.40418600	-1.30188700	-2.74728500
H	0.73188500	-1.57827600	-4.45261500
H	2.07399300	-1.23799600	-3.35373300
C	-0.62110200	0.75145500	-4.27575500
H	-1.29542000	0.45242400	-3.47071600
H	-0.81489300	1.80547400	-4.50531200
H	-0.87265800	0.16395300	-5.16770000
C	2.70092000	-0.05681500	-0.63705600
H	3.66972900	-0.27422400	-0.16977900
H	1.98221400	0.12878900	0.16506200
H	2.38850500	-0.95653600	-1.17309000
C	3.19143300	2.37788700	-0.66032500
H	2.36614800	2.62030500	0.01726900
H	4.06930800	2.12996600	-0.05029700
H	3.43437500	3.27015800	-1.24480800
C	3.98470800	0.94564000	-2.55406100
H	4.93050200	0.84911500	-2.00501600
H	3.84985100	0.02883500	-3.13417200
H	4.09556300	1.78180200	-3.25173700
C	-4.31927500	4.53454900	0.78580700
H	-4.89471600	5.27870000	0.21986200
H	-5.00330300	3.71995300	1.04156300
H	-3.99254000	5.00725700	1.71398200
C	-2.28156000	5.23978000	-0.51263100
H	-1.97072500	5.85187400	0.33850500
H	-1.38557000	4.90932500	-1.05013500
H	-2.86373100	5.88291500	-1.18496000
C	-3.71465600	3.37602400	-1.34291100
H	-2.91361800	3.02110300	-1.99992100
H	-4.35764700	2.52369300	-1.09927900
H	-4.32741300	4.10231100	-1.89254800
C	-0.43333700	2.22151300	2.91470800
H	-1.19419000	1.56556900	3.34563600
H	0.18429300	1.62820800	2.23298800
H	0.21158700	2.57001400	3.73164800
C	0.13673900	4.27596000	1.64056100
H	0.83047300	4.51014400	2.45868400
H	0.67982500	3.71521600	0.87316400
H	-0.18869500	5.22150700	1.20215100
C	-1.83939800	4.28450600	3.19373500
H	-1.20097200	4.53396800	4.05123200
H	-2.17809500	5.22827500	2.75656400
H	-2.71228100	3.75017400	3.58168600

TS20

Pd	-0.43039600	1.53724400	-1.14144100
P	-2.13286300	2.56204000	0.07475600
P	1.26694500	1.30636300	-2.80851900
C	-1.53453200	3.62745600	1.53333200
C	-3.45657800	1.52830000	0.84747200
C	-3.15335200	3.60415700	-1.15916500
C	1.82098700	3.05617600	-3.32684400
C	0.75037300	0.28865400	-4.33381400
C	2.87343300	0.51900100	-2.34423700
O	2.23896700	1.44671200	0.74291500
H	1.44093600	1.68423900	0.21936000
H	2.37547100	0.50726200	0.50154100
H	3.52730800	0.36806400	-3.20789800
H	2.68694300	-0.43664200	-1.85204300
H	3.37941400	1.16162500	-1.62060300
H	-3.00888000	0.77493900	1.49494400
H	-4.16526500	2.12336600	1.43010400
H	-3.99893700	0.99729000	0.06178200
C	-0.70936800	-0.77549600	-0.08225400
C	-0.29681100	-0.67814400	1.33424800
H	0.08969800	-0.81588000	-0.80963800
H	0.53816600	-0.00614200	1.52635000
H	-1.10827400	-0.48320100	2.03692900
C	-2.05636200	-1.20704000	-0.46429000
C	-2.94533900	-1.78003000	0.46336400
C	-2.50612600	-0.99877900	-1.77886400
C	-4.24889900	-2.09746000	0.09184700
H	-2.59967900	-2.00848200	1.46472400
C	-3.81403600	-1.30070700	-2.14623400
H	-1.82962200	-0.55378300	-2.49533500
C	-4.69499900	-1.84628700	-1.20862800
H	-4.91916900	-2.54522700	0.81969800
H	-4.14411400	-1.11098500	-3.16354000
H	-5.71549100	-2.08646200	-1.49226600
N	-0.00420300	-2.10822000	1.24079500
S	1.58936100	-2.43691300	0.98716700
O	1.70218100	-3.81495100	0.49000300
O	2.27870800	-1.36790300	0.20136300
C	2.29256000	-2.37674700	2.63238000
C	2.30959900	-3.54370700	3.39965400
C	2.75180100	-1.16674600	3.15872100
C	2.79753600	-3.49481200	4.70216100
H	1.95242100	-4.47274200	2.96938700
C	3.23122300	-1.13696300	4.46720500
H	2.74306100	-0.26250600	2.56115500
C	3.26508000	-2.29402200	5.25633700
H	2.81738000	-4.40242400	5.29999500
H	3.59115900	-0.19794800	4.87950700
C	3.81890800	-2.25840500	6.65906900
H	3.28114900	-2.94778400	7.31838500
H	3.75688700	-1.25341600	7.08806000
H	4.87570700	-2.55571500	6.67023700

C	0.73219000	-1.18494300	-3.87381100
H	0.12055600	-1.33683400	-2.98073000
H	0.30828200	-1.80633900	-4.67229200
H	1.73653700	-1.55958000	-3.65880300
C	-0.66830300	0.69195400	-4.77511300
H	-1.03484600	-0.01993800	-5.52562100
H	-1.36875600	0.69645700	-3.93664200
H	-0.69052600	1.68744700	-5.22323600
C	1.70024200	0.40573100	-5.53609700
H	2.73548800	0.16876900	-5.27201700
H	1.38934500	-0.30731500	-6.31067300
H	1.67600100	1.40324200	-5.98228500
C	0.74534300	3.72265400	-4.20009400
H	0.96369800	4.79449400	-4.29036000
H	0.72051600	3.30837000	-5.21205100
H	-0.25253300	3.62003000	-3.76112800
C	3.17792900	3.09438700	-4.05008700
H	3.18251700	2.51543500	-4.97511100
H	3.41422000	4.13455900	-4.30927700
H	3.98628800	2.72598600	-3.41225700
C	1.95117500	3.86027900	-2.01752200
H	2.32213200	4.86811400	-2.24577600
H	0.98437300	3.95126900	-1.51523700
H	2.64740200	3.39574400	-1.31189700
C	-3.36219000	2.70343800	-2.39440000
H	-3.96396200	3.24301700	-3.13785700
H	-2.40385200	2.43479700	-2.84894500
H	-3.88792700	1.77551700	-2.14700100
C	-4.53607200	4.03337000	-0.64001900
H	-5.04180700	4.61816000	-1.41920900
H	-5.17149000	3.17139800	-0.41720400
H	-4.47880700	4.65634800	0.25380500
C	-2.35297200	4.84068700	-1.59674800
H	-2.27802000	5.58862100	-0.80251300
H	-1.34116700	4.56900400	-1.91311700
H	-2.85567900	5.31614400	-2.44887900
C	-1.12659800	2.64726300	2.65178500
H	-0.37892900	1.92993600	2.30276100
H	-0.67834100	3.21593400	3.47591700
H	-1.98114700	2.09690200	3.05690700
C	-2.58942900	4.59957500	2.08289500
H	-2.20248100	5.07261900	2.99447300
H	-2.81857600	5.39866500	1.37301200
H	-3.52371400	4.09443100	2.34792400
C	-0.27524500	4.41033800	1.12133500
H	-0.46665500	5.11632800	0.31065900
H	0.09200900	4.98186300	1.98337100
H	0.52099700	3.73363200	0.80215600

TS21

Pd	0.48339600	-1.91392800	-0.77556000
P	2.65250600	-1.58865100	0.14898300
P	-1.46133800	-2.56801700	-1.95136700
C	2.86620100	-1.73106400	2.04042600
C	3.35997100	0.09580600	-0.16121800
C	3.88936900	-2.70850100	-0.76533200
C	-2.95270900	-3.16881300	-0.92087300
C	-1.01715500	-3.81837900	-3.31031200
C	-2.21996100	-1.18927600	-2.92472700
H	-1.44213600	-0.67235500	-3.48287700
H	-2.67527100	-0.46187600	-2.24798000
H	-2.98772000	-1.54703400	-3.61501800
H	4.43410700	0.13112600	0.03627200
H	3.18838800	0.38683300	-1.19892800
H	2.86265300	0.82236000	0.48588400
C	0.22597600	1.30573900	-0.94537500
C	-0.44015400	0.22283800	-0.17844000
H	1.26184900	1.45002300	-0.65065900
H	-0.13725100	0.06066600	0.84488400
H	-1.44591700	-0.04075600	-0.45565400
C	0.07098700	1.33599600	-2.43980000
C	0.88859700	0.51093000	-3.22289800
C	-0.87700500	2.14983200	-3.06136000
C	0.75020900	0.48998000	-4.60984800
H	1.60961300	-0.14011500	-2.73842800
C	-1.01671500	2.12964500	-4.45135600
H	-1.49878500	2.78956500	-2.44479900
C	-0.20773400	1.29866200	-5.22877500
H	1.38312600	-0.16157700	-5.20576100
H	-1.75905100	2.76469900	-4.92687600
H	-0.31908400	1.28250300	-6.30927600
N	-0.73562600	2.10314000	-0.19295700
S	-0.11260700	2.83187000	1.16454800
O	-0.11073800	4.29169300	0.95772800
O	1.15490400	2.20213100	1.61945500
C	-1.36573200	2.44097200	2.37553800
C	-2.64971700	2.97199700	2.22631100
C	-1.05919700	1.59618100	3.43887300
C	-3.63364500	2.63378300	3.14857300
H	-2.86956800	3.63614300	1.39754800
C	-2.05793200	1.27021600	4.35794500
H	-0.05329900	1.20773000	3.54470200
C	-3.35511700	1.77793500	4.22716200
H	-4.63519700	3.04089000	3.03525200
H	-1.82248600	0.61345400	5.19122300
C	-4.29940700	-3.04384200	-1.65623300
H	-4.33482300	-3.63955400	-2.57081200
H	-5.09727700	-3.40561100	-0.99510700
H	-4.53495200	-2.00748000	-1.91348100
C	-2.76685000	-4.62239300	-0.45539600
H	-1.80622100	-4.78149800	0.04167800
H	-3.56297900	-4.86812500	0.25988800
H	-2.85236100	-5.32857500	-1.28616600
C	-2.99641400	-2.25368600	0.31999500
H	-3.80735500	-2.57854300	0.98411800
H	-2.05494700	-2.28558700	0.87695700
H	-3.19468300	-1.20984000	0.05294900
C	3.39878100	-4.16565500	-0.72361100
H	2.37834600	-4.24254600	-1.10742500
H	4.04771100	-4.78511600	-1.35608800
H	3.40929100	-4.58699400	0.28258400
C	3.86447300	-2.24631000	-2.23736400
H	4.46563000	-2.93640700	-2.84248300
H	2.84263200	-2.25114600	-2.63374400
H	4.28178400	-1.24317300	-2.36669000
C	5.32928300	-2.63069300	-0.23743600
H	5.98281900	-3.23323800	-0.88135400
H	5.72013200	-1.60847500	-0.24292600
H	5.41600300	-3.02628400	0.77761300
C	2.95160600	-3.19746400	2.49246700

H	2.89940700	-3.23044700	3.58883400
H	2.13248600	-3.80337900	2.09668300
H	3.89993200	-3.65813500	2.20186600
C	4.09056400	-0.96530100	2.57562100
H	4.14831600	-1.11134800	3.66177700
H	5.02995200	-1.32157100	2.14578100
H	4.01568500	0.11023800	2.39528300
C	1.59834700	-1.09517200	2.64471900
H	0.69519000	-1.63033500	2.33156700
H	1.65947800	-1.13606700	3.74031300
H	1.49544900	-0.04518500	2.35559500
C	-0.21549700	-4.97722500	-2.69271800
H	0.10836800	-5.65981700	-3.48894000
H	0.67619300	-4.60209200	-2.18352200
H	-0.79434900	-5.55443100	-1.96970300
C	-2.22313500	-4.37252400	-4.08346400
H	-1.86445200	-4.99299400	-4.91474400
H	-2.85828800	-5.00303600	-3.45555000
H	-2.84214400	-3.57793000	-4.51194200
C	-0.08852800	-3.06944700	-4.28765900
H	0.74431000	-2.59630300	-3.75685100
H	0.32759000	-3.78601900	-5.00705500
H	-0.61280500	-2.29420000	-4.85310100
C	-4.43259700	1.43246200	5.22505500
H	-4.09330400	0.66964900	5.93206000
H	-4.73167200	2.31562600	5.80315100
H	-5.33216300	1.05643600	4.72402400

TS22

Pd	-0.97943200	-0.38490400	-0.00785400
P	-2.07774600	0.79798100	1.69871100
P	-0.49241100	-1.90459500	-1.75447900
C	-3.66706800	1.56464900	0.98200700
C	-1.21832600	2.27137300	2.41996700
C	-2.47604300	-0.26550500	3.22718900
C	-2.10254800	-2.15598700	-2.74302800
C	0.21742800	-3.56882100	-1.16585700
C	0.69913400	-1.41505400	-3.08248500
H	1.63141300	-1.07435400	-2.62837500
H	0.27441600	-0.58925200	-3.66019700
H	0.91234200	-2.24194600	-3.76641200
H	-1.76475700	2.70916400	3.26037900
H	-0.22407000	1.97956900	2.76767200
H	-1.09509400	3.02782600	1.64021900
C	1.06344300	1.74882000	-1.38512500
C	1.01799800	0.95913800	-0.13452900
H	1.13613200	1.12379400	-2.27550300
H	1.58035500	0.03753900	-0.12359400
H	0.79990000	1.46948500	0.79054300
C	0.11634800	2.89033500	-1.60494400
C	-0.76967300	2.85786800	-2.68452000
C	0.11904600	4.00392000	-0.75709500
C	-1.65503900	3.91293400	-2.90977400
H	-0.76258300	2.00201300	-3.35398200
C	-0.76572900	5.05924600	-0.97854800
H	0.83843800	4.04698600	0.05426300
C	-1.65723700	5.01567300	-2.05447200
H	-2.33784100	3.87486100	-3.75388400
H	-0.75317800	5.92122300	-0.31723000
H	-2.34362900	5.83952200	-2.22782700
N	2.34074400	2.13360000	-0.76971400
S	3.64843900	1.34408700	-1.41976100
O	3.41034500	-0.11233100	-1.59060300
O	4.82899400	1.76812800	-0.65413500
C	3.73078000	2.04678500	-3.06455900
C	3.46001300	1.25582400	-4.17822700
C	4.00546000	3.41093700	-3.20143800
C	3.46504500	1.84128900	-5.44578500
H	3.25980700	0.19839600	-4.04937900
C	4.00654100	3.97759300	-4.47033800
H	4.21187400	4.01247800	-2.32282600
C	3.73372200	3.20416700	-5.61122400
H	3.25820400	1.22756800	-6.31854600
H	4.22014900	5.03743900	-4.58275700
C	-1.92275400	-2.84096500	-4.10752900
H	-1.52952500	-3.85479300	-4.02620700
H	-2.89876200	-2.90413700	-4.60591700
H	-1.25948100	-2.26932700	-4.76374400
C	-3.11293600	-2.93399700	-1.88411100
H	-3.19431100	-2.49892600	-0.88140700
H	-4.10379500	-2.89362500	-2.35452700
H	-2.84028900	-3.98798600	-1.78039100
C	-2.66832500	-0.74320900	-2.98751300
H	-3.59501800	-0.81666500	-3.57142300
H	-2.89241000	-0.24256800	-2.04279900
H	-1.97296300	-0.10846600	-3.54692100
C	-2.84286900	-1.68379000	2.75126600
H	-2.03925300	-2.11461600	2.14738100
H	-3.00370400	-2.32999700	3.62394100
H	-3.75712600	-1.69845500	2.15136300
C	-1.19008900	-0.35319200	4.07363700
H	-1.35573800	-1.07165500	4.88667900
H	-0.32882900	-0.70184900	3.49726700
H	-0.94200900	0.60805800	4.53487500
C	-3.59741000	0.28530000	4.12105700
H	-3.66579500	-0.33315000	5.02514400
H	-3.39987000	1.31300400	4.44230800
H	-4.57436500	0.25603900	3.63207400
C	-4.65871200	0.44167400	0.63560200
H	-5.49294100	0.85821700	0.05672400
H	-4.18044900	-0.33401800	0.02759300
H	-5.07962200	-0.03302300	1.52612700
C	-4.34172200	2.61905700	1.87491200
H	-5.19769600	3.04285600	1.33390000
H	-4.71678000	2.21191500	2.81382300
H	-3.66301100	3.44608400	2.10480800
C	-3.24244900	2.25898700	-0.32391600
H	-2.75173100	1.56477400	-1.00821600
H	-4.13008300	2.66945500	-0.82246900
H	-2.55345700	3.08740300	-0.14629300
C	-0.50022400	-4.00118400	0.12525200
H	-0.04720800	-4.92927900	0.49679900
H	-0.40137900	-3.24430100	0.90615700
H	-1.56500300	-4.18886900	-0.03228400
C	0.13142500	-4.70149200	-2.20000400
H	0.68337800	-5.57128700	-1.82132500
H	-0.89889900	-5.02191600	-2.37712800
H	0.58000900	-4.42487700	-3.15957300
C	1.70409600	-3.31588800	-0.83765300
H	1.83734100	-2.49874500	-0.12499300

H	2.11900100	-4.22058500	-0.37584500
H	2.29403200	-3.09902900	-1.73224300
C	3.73342500	3.83994900	-6.97926800
H	2.98990400	4.64379500	-7.04065100
H	4.70871900	4.28612200	-7.20789400
H	3.50569000	3.10883600	-7.76048200
O	1.25557400	-1.52480700	2.08897500
H	0.52969600	-1.24001600	1.48588800
H	1.90584300	-0.80740100	2.02575100

TS23

Pd	1.21673300	0.16730500	-0.19363000
P	1.36786600	-2.15286400	-0.57747700
P	1.75521200	2.43563700	-0.39128000
C	2.44796300	-2.47632100	-2.12064200
C	-0.19774100	-3.02072900	-1.04833400
C	2.01042900	-3.18093500	0.89338800
C	1.24768600	3.68065200	0.87864100
C	1.07128700	3.21566500	-1.99173000
C	3.65542700	2.59037600	-0.30716500
H	1.52436400	4.70080900	0.59651200
H	1.71840500	3.43297800	1.83242900
H	0.17031700	3.63229700	1.03098800
H	-0.52593900	-2.62964500	-2.01642900
H	-0.07215000	-4.10433900	-1.13455000
H	-0.97339300	-2.80036300	-0.31422300
C	-0.71579100	0.28191200	1.53766100
C	-1.84791500	0.70203700	0.69809600
H	-0.49218400	-0.77331200	1.51540900
H	-1.96301700	0.13904700	-0.23162200
H	-1.94674100	1.77375600	0.51834300
C	-0.12402700	1.09915700	2.60135900
C	1.11673300	0.72185000	3.14277800
C	-0.75502500	2.25043500	3.10213200
C	1.72995300	1.49395800	4.12579300
H	1.60955800	-0.16446600	2.76033600
C	-0.14872800	3.01247100	4.09747000
H	-1.73999800	2.51859900	2.73742800
C	1.10037900	2.64612300	4.60552600
H	2.69590100	1.19328200	4.52132500
H	-0.65410700	3.89454000	4.47989100
H	1.57318500	3.24550300	5.37810300
N	-2.63407400	0.27373000	1.85612700
S	-3.29321700	-1.23853700	1.73982100
O	-3.93662400	-1.52314700	3.03010500
O	-2.34722200	-2.24349700	1.19413900
C	-4.57245800	-1.03304100	0.50391400
C	-5.75663200	-0.37964000	0.85535200
C	-4.35734600	-1.46880000	-0.80328400
C	-6.72957700	-0.16800400	-0.11571900
H	-5.90792800	-0.05220900	1.87828500
C	-5.34268000	-1.24596200	-1.76572000
H	-3.43935300	-1.98956800	-1.05240600
C	-6.53910900	-0.59629600	-1.43918600
H	-7.65425600	0.33581800	0.15396300
H	-5.18142300	-1.58764300	-2.78489000
C	-7.61521900	-0.38437400	-2.47506900
H	-8.08984900	0.59630700	-2.36247000
H	-7.21301100	-0.45658900	-3.49017800
H	-8.40584400	-1.13972300	-2.37829400
C	-0.43295900	3.44783400	-1.74312100
H	-0.90997100	3.75407900	-2.68259600
H	-0.92552500	2.53115100	-1.40203700
H	-0.61628000	4.23598700	-1.00713000
C	1.72278400	4.54589000	-2.39345000
H	2.76252200	4.41331700	-2.70430300
H	1.17894100	4.97412900	-3.24573200
H	1.69576600	5.28351100	-1.58519600
C	1.19907500	2.20613500	-3.14444100
H	0.69759700	1.26768400	-2.89058500
H	0.72975600	2.61989000	-4.04690000
H	2.24015100	1.97777500	-3.38518600
C	4.17342700	4.02223100	-0.09299700
H	5.27035000	4.00309400	-0.05201400
H	3.88609300	4.70077400	-0.89808500
H	3.82095000	4.44488800	0.85240500
C	4.08156300	1.73518600	0.90467400
H	3.62103700	2.08194100	1.83541500
H	3.79590300	0.68819400	0.76386500
H	5.17159800	1.79191100	1.02486400
C	4.29089200	1.99038700	-1.57102700
H	4.12220100	2.61338600	-2.45428600
H	5.37632500	1.90632900	-1.42995700
H	3.89864300	0.98903200	-1.77402800
C	3.93322000	-2.25343700	-1.79489300
H	4.51036000	-2.22491000	-2.72822700
H	4.09207300	-1.30451600	-1.27231300
H	4.34457400	-3.05860900	-1.18061200
C	2.02382900	-1.42100000	-3.15994500
H	2.23847000	-0.41205300	-2.80296700
H	2.57429000	-1.58834400	-4.09535900
H	0.95344100	-1.47146900	-3.38833600
C	2.25722500	-3.86766800	-2.75000900
H	1.23175100	-4.01850600	-3.09940500
H	2.91568500	-3.95499900	-3.62415100
H	2.50626800	-4.68180100	-2.06800600
C	3.23466200	-2.47778500	1.51084800
H	3.02581300	-1.42154000	1.70718600
H	3.49085800	-2.96204400	2.46197300
H	4.11475500	-2.52643200	0.86603600
C	2.37235900	-4.63454800	0.55283700
H	3.24496600	-4.70003300	-0.10189600
H	2.61831400	-5.17065900	1.47858200
H	1.54207500	-5.16707600	0.07845200
C	0.88946100	-3.19404200	1.95225700
H	1.23267100	-3.76486400	2.82435500
H	0.64800900	-2.18762900	2.30087200
H	-0.03461800	-3.65144300	1.59317900

TS24

Pd	1.38275600	1.99093600	0.30404000
P	0.15431300	2.99465600	2.01768200
P	3.20022000	1.18701300	-0.94864600
C	0.77718400	2.34752800	3.70031200
C	-1.66731100	2.69632800	2.15023000
C	0.25937200	4.89322600	1.93028300
C	3.25124100	1.68835500	-2.78048800
C	3.33004000	-0.65535000	-1.05299200
C	4.81173500	1.66120500	-0.05149800
H	2.53205200	-1.04018900	-1.69298700

H	4.29612100	-0.98655700	-1.44536400
H	3.18994600	-1.08491000	-0.05803500
H	-1.84520300	1.64259100	2.38134600
H	-2.13642100	3.30511700	2.92887100
H	-2.14150900	2.90617400	1.19205500
C	-0.90197500	0.18115400	-1.11038900
C	-0.03218500	0.03016600	0.07850100
H	-0.34762600	0.18498500	-2.04746900
H	0.87465600	-0.54675900	-0.01226700
H	-0.47454500	0.13826700	1.05533400
C	-1.99228300	1.21478200	-1.08609400
C	-1.71133300	2.51781900	-1.51461100
C	-3.27539600	0.90636400	-0.62871600
C	-2.70015700	3.50037000	-1.48218300
H	-0.70933200	2.76259300	-1.85439200
C	-4.26526900	1.89087700	-0.59251800
H	-3.48435000	-0.10876400	-0.31000700
C	-3.98110700	3.19018000	-1.01696600
H	-2.47028100	4.50849500	-1.81481000
H	-5.26056300	1.64067000	-0.23566000
H	-4.75187100	3.95534900	-0.99092900
N	-1.29787800	-1.14378100	-0.63552400
S	-0.57743600	-2.42147900	-1.41848200
O	-1.64008600	-3.27101300	-1.98424100
O	0.51907100	-1.99596300	-2.32571600
C	0.18301500	-3.29764100	-0.05593200
C	-0.56806700	-3.58561200	1.08746900
C	1.52013500	-3.67441700	-0.14556800
C	0.04112000	-4.24158000	2.15115700
H	-1.60705200	-3.28062600	1.14468000
C	2.11532000	-4.33688800	0.93004900
H	2.08381300	-3.44297300	-1.04152700
C	1.39101000	-4.62670300	2.09115300
H	-0.53667700	-4.45956200	3.04580900
H	3.15990800	-4.62968900	0.86452100
C	2.03117400	-5.34889700	3.25079800
H	1.82770200	-4.83810100	4.19886700
H	3.11592900	-5.41978600	3.12778400
H	1.63825900	-6.36920600	3.34364700
C	-0.65184200	5.31782000	0.76187100
H	-0.51655000	6.39058800	0.57456300
H	-0.39891900	4.77800800	-0.15562000
H	-1.71041500	5.14628300	0.97424900
C	-0.18504500	5.62480400	3.20454400
H	0.50823500	5.45994800	4.03390200
H	-0.21312900	6.70517600	3.01133600
H	-1.18720700	5.32411100	3.52728200
C	1.69856200	5.29957900	1.56723500
H	2.41856600	5.02842500	2.34228200
H	2.01048000	4.81756000	0.63527100
H	1.74666100	6.38781600	1.42979100
C	-0.13937400	2.67149400	4.89210200
H	0.29439900	2.24197600	5.80450500
H	-0.25536700	3.74396800	5.05699800
H	-1.13521700	2.23550900	4.76952400
C	2.19351700	2.87865700	3.97063600
H	2.19083600	3.94542700	4.21172300
H	2.62594700	2.34827900	4.82885500
H	2.84972500	2.72021800	3.10877200
C	0.85216000	0.81269400	3.55565800
H	1.52261900	0.52167600	2.74073000
H	1.22517600	0.37660400	4.49138400
H	-0.13167100	0.37150800	3.35959900
C	4.74403000	3.14408400	0.35373200
H	3.85562300	3.33473600	0.96214300
H	5.63310800	3.40330300	0.94367000
H	4.70866500	3.81283700	-0.50939000
C	4.83937400	0.82072200	1.24147900
H	5.64930800	1.18084100	1.88837000
H	3.89781500	0.91184700	1.79444300
H	5.02209400	-0.23882600	1.04001300
C	6.09703600	1.39705300	-0.84726600
H	6.18961400	2.06512900	-1.70799200
H	6.96691100	1.57366400	-0.20106300
H	6.15857600	0.36367400	-1.20365900
C	1.82818700	1.42631800	-3.31230800
H	1.09393600	2.03171600	-2.77391100
H	1.78240900	1.69502300	-4.37562100
H	1.53903700	0.37452300	-3.22185600
C	3.54254400	3.19304300	-2.89663600
H	3.36599000	3.52135300	-3.92887800
H	2.88666500	3.77574200	-2.23927500
H	4.58054700	3.43198400	-2.64798700
C	4.23935500	0.88566200	-3.64387900
H	5.27791000	1.01344300	-3.33359200
H	4.00696700	-0.18341700	-3.63470600
H	4.16141600	1.22616900	-4.68448700

TS25

Pd	0.47141900	-1.95971900	-0.71677600
P	2.72780500	-1.59253200	-0.12572300
P	-1.68133800	-2.88148400	-0.99538600
C	2.92668800	-2.06582800	1.71001300
C	3.43564500	0.11740000	-0.13884300
C	3.96285600	-2.54828600	-1.21480600
C	-2.49877800	-3.21144000	0.69692200
C	-1.68803600	-4.45357600	-2.06994300
C	-2.94359600	-1.81172500	-1.83143100
H	-2.50385000	-1.37482300	-2.73019200
H	-3.23218100	-0.99410000	-1.16468300
H	-3.84696400	-2.36541200	-2.10278400
H	4.50427700	0.11952200	0.09505000
H	3.29599400	0.55463400	-1.12848400
H	2.91063400	0.74286500	0.58761400
C	0.25391100	1.34315400	-0.98927900
C	-0.37165900	0.23617900	-0.23414600
H	1.28918800	1.53258800	-0.72065500
H	-0.05212600	0.06563000	0.78130300
H	-1.36518400	-0.06068900	-0.51808500
C	0.03640200	1.39040100	-2.47222600
C	1.13400800	1.46326700	-3.33489700
C	-1.25249100	1.31942300	-3.01537900
C	0.95288300	1.43770700	-4.72129900
H	2.13668300	1.53240300	-2.92244000
C	-1.43694600	1.28991000	-4.39644100
H	-2.10859000	1.29992800	-2.34902100
C	-0.33394900	1.34088900	-5.25464900
H	1.81536300	1.48887800	-5.37923000
H	-2.44137200	1.23043900	-4.80570500
H	-0.47737000	1.31384600	-6.33062600

N	-0.73528300	2.08855400	-0.20001400
S	-0.17982300	2.69978700	1.24538700
O	-0.43426700	4.15028000	1.25859600
O	1.18934800	2.22876200	1.57836900
C	-1.29645300	1.91597000	2.40591000
C	-2.67612000	2.05661300	2.23380700
C	-0.78119300	1.13232500	3.43463300
C	-3.53841700	1.37945800	3.08935100
H	-3.06312200	2.67657900	1.43250200
C	-1.66052100	0.46229700	4.28675800
H	0.29165200	1.04661300	3.55821900
C	-3.04577900	0.56294300	4.12048800
H	-4.61250200	1.48038500	2.95598000
H	-1.26127400	-0.15402100	5.08799900
O	0.96691900	-1.81877600	-3.86755300
H	0.83083100	-1.91705500	-2.89444800
H	0.78821000	-0.87503500	-4.01858000
C	-4.00921100	-3.49708800	0.63537200
H	-4.25057500	-4.41230100	0.09503800
H	-4.39013600	-3.60813100	1.65890200
H	-4.55894500	-2.67127800	0.17324300
C	-1.76378900	-4.36619400	1.39720600
H	-0.68229000	-4.19158400	1.41789800
H	-2.11379500	-4.44665500	2.43425600
H	-1.94668900	-5.32870000	0.91227800
C	-2.29926300	-1.93808000	1.54177200
H	-2.73915000	-2.09054900	2.53478900
H	-1.24177300	-1.70710200	1.67700000
H	-2.78538400	-1.05936400	1.10707200
C	3.35954100	-3.91996000	-1.56456800
H	2.41340100	-3.80107000	-2.09830700
H	4.05183300	-4.46687600	-2.21762700
H	3.17510700	-4.53483800	-0.67986900
C	4.11993600	-1.74742700	-2.52437100
H	4.71509800	-2.34284000	-3.22851200
H	3.15631200	-1.54498300	-3.00096300
H	4.64992200	-0.80357000	-2.36503300
C	5.35358900	-2.74015500	-0.59173300
H	6.01861600	-3.19050600	-1.33981300
H	5.80400600	-1.79092800	-0.28415900
H	5.33387400	-3.41055100	0.27158300
C	2.76833600	-3.58593600	1.87311400
H	2.68466200	-3.83289000	2.93939900
H	1.86297000	-3.94401800	1.36957400
H	3.62478300	-4.13715500	1.47472900
C	4.24004700	-1.59153700	2.35464900
H	4.24445700	-1.88994500	3.41098300
H	5.12454300	-2.02461700	1.88675900
H	4.33426900	-0.50198600	2.32518300
C	1.76214100	-1.37457900	2.44708400
H	0.79889200	-1.76604900	2.10938100
H	1.84941400	-1.56520300	3.52472200
H	1.76482900	-0.29056100	2.29884700
C	-0.44246700	-5.28650500	-1.71882200
H	-0.39257900	-6.16367500	-2.37672100
H	0.46936200	-4.70061900	-1.85870900
H	-0.45666300	-5.64241200	-0.68516900
C	-2.94577300	-5.32424800	-1.93667300
H	-2.89773100	-6.13074700	-2.67951000
H	-3.02707600	-5.79251400	-0.95245000
H	-3.86277900	-4.75850700	-2.12975800
C	-1.57080200	-3.99309600	-3.53762700
H	-0.73619700	-3.30591800	-3.70312600
H	-1.40811100	-4.87634700	-4.16850900
H	-2.49027400	-3.51006500	-3.88364200
C	-3.99262000	-0.21029900	5.00412600
H	-3.50285200	-0.53681400	5.92646200
H	-4.86885900	0.38868100	5.27430300
H	-4.36045100	-1.10783700	4.48911300

1l

C	0.18445200	1.27724100	-0.54412800
C	-0.61691400	0.25858100	0.18652000
H	-1.38570500	-0.25782100	-0.38444300
N	-0.96053500	1.70375900	0.28153200
S	-0.64034900	2.61158700	1.68504100
O	0.74704000	2.44451500	2.15731200
O	-1.75542800	2.35379700	2.60542500
C	-0.80022300	4.25032000	1.00786500
C	0.34751400	4.98035800	0.70559700
C	-2.07547500	4.77355600	0.78262100
C	0.20999100	6.25747800	0.16329100
H	1.32559800	4.55580100	0.90118900
C	-2.19189200	6.04925400	0.24154800
H	-2.95396600	4.18917800	1.03263600
C	-1.05476600	6.80975300	-0.07511400
H	1.09904200	6.83461100	-0.07535600
H	-3.17954600	6.46552400	0.06239200
C	-1.19927200	8.20218500	-0.63596900
H	-0.27686700	8.53670100	-1.11975100
H	-2.01119200	8.25352400	-1.36908600
H	-1.43518400	8.91982500	0.16021400
C	-0.02618400	-0.55267400	1.32191400
H	0.79362800	0.00323400	1.78455400
H	0.41142000	-1.45063700	0.86269600
H	1.17604700	1.48769700	-0.14900700
H	0.05366700	1.44347100	-1.60989500
C	-1.03892000	-0.97376000	2.39915100
H	-0.49980200	-1.55952700	3.15609600
H	-1.41995300	-0.08090200	2.90672900
C	-2.21882500	-1.79997500	1.87501400
H	-1.84076900	-2.65738900	1.29916500
H	-2.81332500	-1.19581000	1.17730000
C	-3.12692200	-2.29673800	3.00310500
H	-3.97349600	-2.87317700	2.61332900
H	-2.57426000	-2.93967500	3.69903900
H	-3.52980100	-1.45488400	3.57870600

1l ring-opening TS (2-possition)

Pd	0.48469100	-1.91889100	-0.65243800
P	2.75494800	-1.81496100	-0.03438800
P	-1.56137200	-2.63370100	-1.49376100
C	3.17038600	-3.25280500	1.14615800
C	3.42031300	-0.37123000	0.92208700
C	3.86013800	-1.84348500	-1.58109400
C	-1.64863300	-4.53539300	-1.43213100
C	-1.90867000	-1.98233100	-3.24764000
C	-3.09646500	-2.15672800	-0.56691300
H	-3.09836900	-1.08175600	-0.37302600
H	-3.09409000	-2.66519700	0.40129000

H	-4.01581100	-2.41750800	-1.10004400
H	4.51389800	-0.35704400	0.94928100
H	3.06325300	0.57192200	0.50727900
H	3.04970600	-0.42856200	1.94840200
C	0.47689400	1.15613000	-0.90170000
C	-0.28714300	0.34283400	0.06609800
H	-1.28949400	0.08258000	-0.24348500
N	-0.43236500	2.19692900	-0.43504600
S	0.25311300	3.35309800	0.53278500
O	1.61018200	2.98263200	1.01081400
O	-0.74735400	3.77970700	1.52828200
C	0.46824200	4.70882800	-0.61672900
C	1.69745800	4.89033100	-1.24953000
C	-0.61432000	5.54449800	-0.90138200
C	1.83936900	5.92198400	-2.17715000
H	2.53024100	4.24105000	-1.00283100
C	-0.45536700	6.56937300	-1.82852400
H	-1.55921900	5.39148900	-0.39177600
C	0.77073700	6.77483900	-2.48017000
H	2.79698700	6.07067900	-2.66944800
H	-1.29398400	7.22453200	-2.05075500
C	-3.04794600	-5.13176100	-1.66038400
H	-3.43984200	-4.92647900	-2.65719500
H	-2.99274200	-6.22208900	-1.54572800
H	-3.77161900	-4.76462700	-0.92621600
C	-0.64924500	-5.12426300	-2.44178900
H	0.34293700	-4.67433000	-2.32471400
H	-0.55492900	-6.20498400	-2.27485600
H	-0.97389800	-4.97973200	-3.47627100
C	-1.18647200	-4.92740800	-0.01313900
H	-1.19354800	-6.02107400	0.08110300
H	-0.17450100	-4.56435200	0.18520700
H	-1.84686400	-4.52365900	0.76184600
C	3.29473600	-2.86656900	-2.58281100
H	2.23653400	-2.66815600	-2.78096700
H	3.84874300	-2.79660100	-3.52818000
H	3.38167500	-3.89315600	-2.21980400
C	3.72499900	-0.44206100	-2.21227100
H	4.24415700	-0.43106000	-3.17877000
H	2.67523400	-0.18655100	-2.39197700
H	4.16952400	0.33873000	-1.58761600
C	5.34331600	-2.13660300	-1.31875000
H	5.91271200	-1.98989000	-2.24582100
H	5.77182400	-1.47100400	-0.56272300
H	5.50379200	-3.16972600	-0.99888000
C	3.07235400	-4.59029600	0.39456300
H	3.10548700	-5.41763700	1.11505600
H	2.13435100	-4.66703000	-0.16559000
H	3.90296600	-4.72965500	-0.30347100
C	4.53982400	-3.14399000	1.83735400
H	4.66535800	-3.99493500	2.51950200
H	5.37132700	-3.16455700	1.13056200
H	4.62404400	-2.23270500	2.43649000
C	2.07361400	-3.22249600	2.23157800
H	1.07719900	-3.33047100	1.79183700
H	2.23682100	-4.04728200	2.93737100
H	2.08858000	-2.28990300	2.80616100
C	-0.59690100	-2.01864100	-4.05315600
H	-0.76392200	-1.57573900	-5.04368300
H	0.18745900	-1.45022500	-3.54359700
H	-0.22746600	-3.03687200	-4.19914600
C	-3.00920300	-2.72924000	-4.01420300
H	-3.21927100	-2.19950000	-4.95241200
H	-2.70964600	-3.74763300	-4.27502400
H	-3.94543100	-2.77761600	-3.44904400
C	-2.33583100	-0.50765800	-3.09406000
H	-1.61657400	0.07181700	-2.50899300
H	-2.39662800	-0.04830500	-4.08841500
H	-3.31900000	-0.40951900	-2.62466300
C	0.93501600	7.90776800	-3.46306300
H	0.05825000	8.00164400	-4.11308500
H	1.05469300	8.86616500	-2.94131800
H	1.81643700	7.76413000	-4.09542700
C	0.01175200	0.21121600	1.53711200
H	0.84901900	0.85935800	1.80691000
H	0.33675600	-0.82165200	1.71707100
H	1.52982500	1.29514500	-0.66241400
H	0.33992000	0.90523300	-1.95496100
C	-1.20584400	0.49565500	2.42539100
H	-1.52790400	1.53048700	2.27063000
H	-2.03758800	-0.15371400	2.11286800
C	-0.90534000	0.25848600	3.90745200
H	-0.07141800	0.90628000	4.21057800
H	-0.56177000	-0.77696500	4.04997500
C	-2.11441400	0.52732000	4.80654500
H	-1.87681200	0.35460700	5.86248100
H	-2.45420300	1.56458600	4.70343700
H	-2.95531800	-0.12564800	4.54083700

1l ring-opening TS (2-possition)

Pd	1.25597500	-1.59892200	-1.55079800
P	3.43601200	-0.78788600	-1.25731600
P	-0.56735600	-2.97439000	-2.01587200
C	4.16714400	-1.26254800	0.43018000
C	3.64965700	1.05387000	-1.26821100
C	4.55343800	-1.34606400	-2.68947300
C	-0.28307000	-4.65549200	-1.16427900
C	-0.81324200	-3.17168300	-3.89263100
C	-2.27856800	-2.55484600	-1.44080500
H	-2.53732600	-1.54791500	-1.77083400
H	-2.30351800	-2.56709700	-0.34813900
H	-3.03233700	-3.25206000	-1.81761200
H	4.69895200	1.35456100	-1.33573400
H	3.10635000	1.47343400	-2.11904700
H	3.21854400	1.47496700	-0.35571600
C	0.19769900	0.49459200	-1.00592400
C	-0.88340800	0.33879900	-0.00921900
H	-1.82149300	-0.00234000	-0.44899500
N	-0.85187700	1.80639800	-0.12630500
S	0.03890400	2.65780000	0.98134400
O	1.42332400	2.14222000	1.17618600
O	-0.75013900	2.89143900	2.21089400
C	0.18427300	4.21724400	0.12037900
C	1.19873400	4.38839800	-0.82180900
C	-0.71281300	5.24429600	0.40335700
C	1.30394900	5.60397600	-1.49214400
H	1.89747200	3.58279500	-1.01868000
C	-0.59302000	6.45634100	-0.27506300
H	-1.48612000	5.08951600	1.14745400
C	0.41339800	6.65542800	-1.22902500

H	2.09202400	5.74339100	-2.22788200
H	-1.29100400	7.26097900	-0.05897700
C	-1.50953600	-5.58267500	-1.14109900
H	-1.85066800	-5.85782300	-2.14060900
H	-1.24817400	-6.50926000	-0.61390700
H	-2.35009300	-5.13088900	-0.60572700
C	0.90747800	-5.37138500	-1.82249800
H	1.77423800	-4.70631200	-1.90305400
H	1.19797500	-6.23557600	-1.21131700
H	0.66308100	-5.74384800	-2.82163900
C	0.10013500	-4.32327800	0.29332900
H	0.29306000	-5.25611600	0.83910300
H	1.00008800	-3.70126100	0.33402100
H	-0.70059100	-3.79313000	0.82061400
C	4.30996000	-2.84072600	-2.96943600
H	3.24708300	-3.03531500	-3.14121300
H	4.86643900	-3.13819600	-3.86802200
H	4.64093100	-3.47823100	-2.14668800
C	4.07812400	-0.55035800	-3.92275900
H	4.56065100	-0.95403100	-4.82166300
H	2.99291200	-0.63149100	-4.05415600
H	4.33983700	0.50986900	-3.85434000
C	6.05258200	-1.09398500	-2.47638100
H	6.59568300	-1.32327500	-3.40252100
H	6.26417300	-0.05020100	-2.22293300
H	6.46741700	-1.72974300	-1.68926700
C	4.49757800	-2.76339100	0.45833700
H	4.72815300	-3.06359200	1.48842600
H	3.65032600	-3.36689600	0.11451600
H	5.36795600	-3.00693800	-0.15759400
C	5.40489300	-0.44518000	0.83730400
H	5.74296700	-0.77913100	1.82671700
H	6.24079700	-0.56844900	0.14534600
H	5.17756000	0.62186700	0.91604100
C	3.04317500	-0.99322900	1.45222700
H	2.17263400	-1.62439600	1.25094800
H	3.41159000	-1.22264600	2.46098100
H	2.70878500	0.04848700	1.44746300
C	0.56357000	-3.28866500	-4.57053600
H	0.43242800	-3.31441800	-5.66025300
H	1.19399800	-2.43112800	-4.31461400
H	1.09610100	-4.19591500	-4.27615600
C	-1.70037300	-4.35232300	-4.31151700
H	-1.88799600	-4.30071800	-5.39189800
H	-1.22117500	-5.31425400	-4.10946300
H	-2.67317300	-4.33945600	-3.80935400
C	-1.46368600	-1.85699800	-4.37309200
H	-0.90264500	-0.98166600	-4.02616900
H	-1.46929100	-1.83844700	-5.47004400
H	-2.50003800	-1.76016400	-4.03636700
C	0.55796300	7.98102700	-1.93507900
H	1.25991700	8.63480200	-1.40100000
H	0.94284500	7.85240700	-2.95200800
H	-0.39917200	8.50854500	-1.99452400
C	-0.62220300	-0.39700500	1.29524500
H	0.28243400	-0.01030100	1.77102300
H	-0.40930200	-1.43983100	1.02848100
H	1.15848200	0.80955300	-0.64037200
H	-0.05700800	0.68949300	-2.03936200
C	-1.80252700	-0.33655500	2.26681700
H	-2.00897900	0.70960300	2.51885300
H	-2.70425400	-0.72975300	1.77235100
C	-1.53968100	-1.13223200	3.54894900
H	-0.64096700	-0.73212600	4.03782900
H	-1.30904800	-2.17625200	3.28921300
C	-2.71639800	-1.09301800	4.52676600
H	-2.50345600	-1.66498300	5.43720300
H	-2.94321800	-0.06187700	4.82281100
H	-3.62177500	-1.51316800	4.07086900
